# Direct alkylation of heteroarenes with unactivated bromoalkanes using photoredox gold catalysis†

**DOI:** 10.1039/c6sc00807k

**Published:** 2016-04-18

**Authors:** T. McCallum, L. Barriault

**Affiliations:** a Centre for Catalysis, Research and Innovation, Department of Chemistry and Biomolecular Sciences, University of Ottawa 10 Marie Curie Ottawa ON K1N 6N5 Canada lbarriau@uottawa.ca

## Abstract

Although visible light photoredox catalysis has emerged as a powerful tool for the construction of C–C bonds, common catalysts and/or their photoexcited states suffer from low redox potentials, limiting their applicability to alkyl radical generation from substrates with activated carbon–halogen bonds. Radicals derived from these activated compounds, being highly electrophilic or stabilized, do not undergo efficient addition to heteroarenes. Herein we describe the photocatalytic generation of nucleophilic alkyl radicals from unactivated bromoalkanes as part of a universal and efficient cross-coupling strategy for the direct alkylation of heteroarenes using a dimeric gold(i) photoredox catalyst, [Au_2_(bis(diphenylphosphino)methane)_2_]Cl_2_. The method proves to be efficient for alkylation of arenes under mild conditions in the absence of directing groups.

## Abstract 2

A protocol for direct C–H alkylation of heteroarenes was developed using photoredox catalysis as a mode of activation for unactivated bromoalkanes. Dimeric Au(i) complex, [Au_2_(dppm)_2_]Cl_2_, served as an efficient photocatalyst for this transformation.

## Graphical abstract

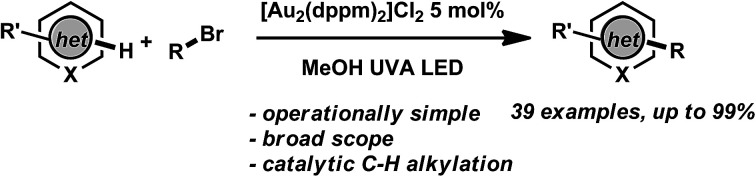

Over the past few decades, we have seen a dramatic increase in photochemical research, leading to great progress in the fields of energy storage, water splitting, photovoltaic devices, and more recently, transformations of organic molecules in the synthesis of high-value products and therapeutics.^1–5^ For over a century, chemists have found inspiration in the sophistication of light-harvesting biomolecules, owing the development of photoexcitable complexes to furthering our knowledge of the “well-kept secrets of the plants”.^6,7^ A fresh take on classical radical chemistry through the eyes of photoredox catalysis has produced unconventional reactivity in organic synthesis, unlocking mild and environmentally sound methodology for the construction of C–C bonds.^2–5^ Photoredox catalysis methods are advantageous to classical methods of alkyl and aryl radical generation from which the use of hazardous radical initiators and/or harsh conditions are often employed.^8^

The synthesis and diversification of heteroaromatic compounds *via* C–H functionalization has had a tremendous impact in organic materials and drug discovery.^9,10^ Although important advances have been made, C–H functionalization of heteroarenes without directing groups remains a standing challenge in organic synthesis.^11^ The addition of carbon centered radicals derived from carboxylic acids to heteroarenes, known as the Minisci reaction, has been instrumental in the functionalization of heteroaromatics.^12^ This seminal work describes an expedient synthesis of alkyl functionalized heteroarenes but suffers from harsh conditions that often lead to byproduct formation. Contemporary C–H alkylation protocols have been developed from a variety of functional groups but necessitate the use of stoichiometric oxidants/reagents and are often accompanied by multi-step syntheses of the starting materials (Fig. 1).^13–21^ In combination, these drawbacks limit functional group tolerance and the scope of the parent radicals. Keeping this in mind, we wondered if a practical Csp^3^–Csp^2^ coupling strategy for direct C–H alkylation of heteroarenes of broader applicability could be conceived using photoredox catalysis and simple bromoalkanes. This remains a challenge in organic synthesis as no photocatalytic Minisci-type methodologies have been described involving carbon-centered radicals from simple bromoalkanes. To this effect, photoexcited [Au_2_(dppm)_2_]Cl_2_ has been shown to undergo reduction chemistry of both unactivated bromoalkanes and arenes (and other functional groups),^22^ while recently luminescent platinum complexes^23^ and organic dyes^24^ have been reported to reduce unactivated bromoalkanes and arenes.

**Fig. 1:**
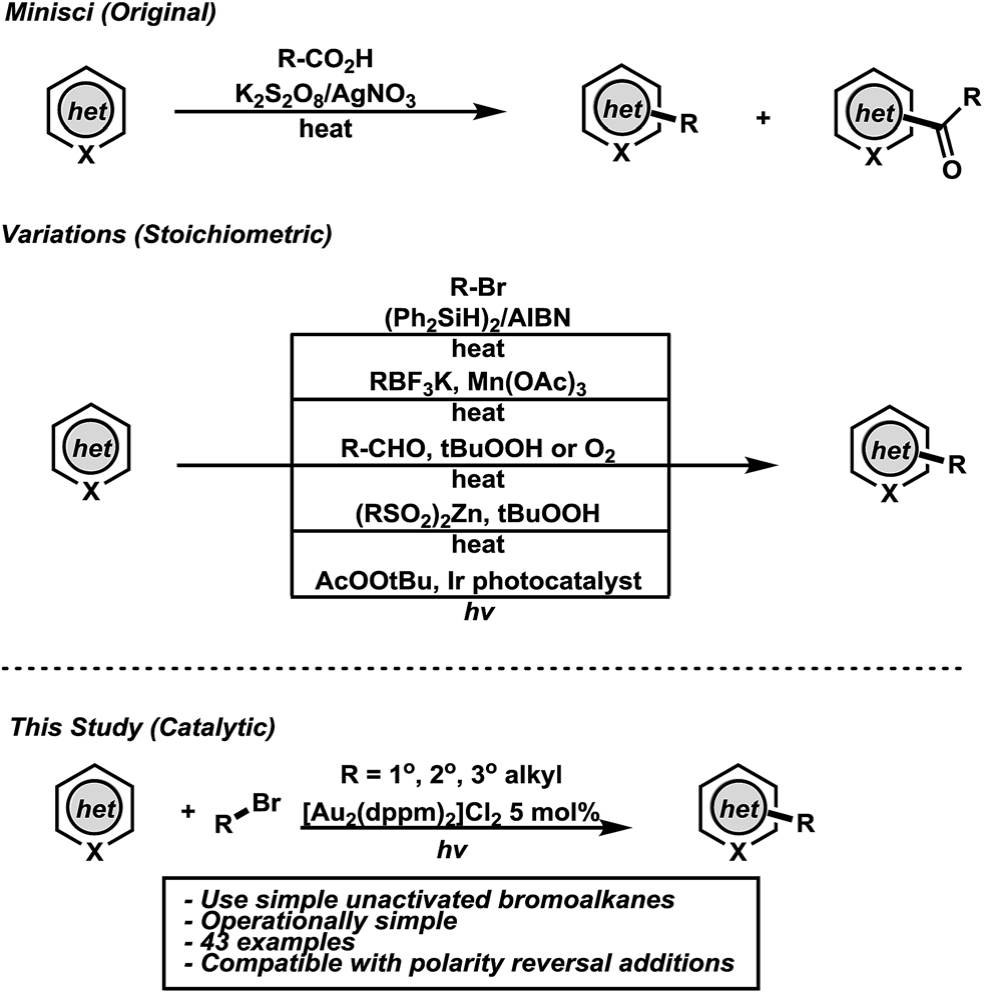
Previous and present work in direct C–H alkylations.

We hypothesized that a redox neutral C–H functionalization protocol employing a photoredox catalyst as reductant and oxidant would mitigate the need for stoichiometric additives. Based on previous studies in our laboratory, we envisioned the genesis of alkyl radicals derived from broadly available bromoalkanes (1, *ca*. −2.0 V *vs.* SCE)^25^ through an oxidative quenching mechanism with photoexcited [Au_2_(dppm)_2_]Cl_2_ (−1.63 V *vs.* SCE) (Fig. 2).^26^ These nucleophilic alkyl radicals would then be uniquely reactive towards heteroaromatics 2 prone to addition at electron deficient locations. The resulting aminyl radical intermediate 3 would be subsequently oxidized by [Au_2_(dppm)_2_]^3+^, thus completing the catalytic cycle while providing the directly alkylated heteroarene products 4. It is conceivable that intermediate 3 could undergo chain propagating electron transfer with 1, but this is likely to be a minor pathway considering the reduction potential of bromoalkanes. Ru and Ir based polypyridyl complexes operate through an outer sphere mechanism of metal-to-ligand charge transfer (MLCT) for activation of carbon–halogen bonds. Complexes like [Ru(bpy)_3_]Cl_2_ and *fac*-Ir(ppy)_3_ have excited state reduction potentials of −0.81 V and −1.73 V *vs.* SCE, respectively. Substrate engagement is, therefore, limited to compounds with reduction potentials lower than the excited states of the Ru and Ir based polypyridyl complexes. These complexes are unable to undergo efficient excited state quenching with unactivated bromoalkanes.

**Fig. 2:**
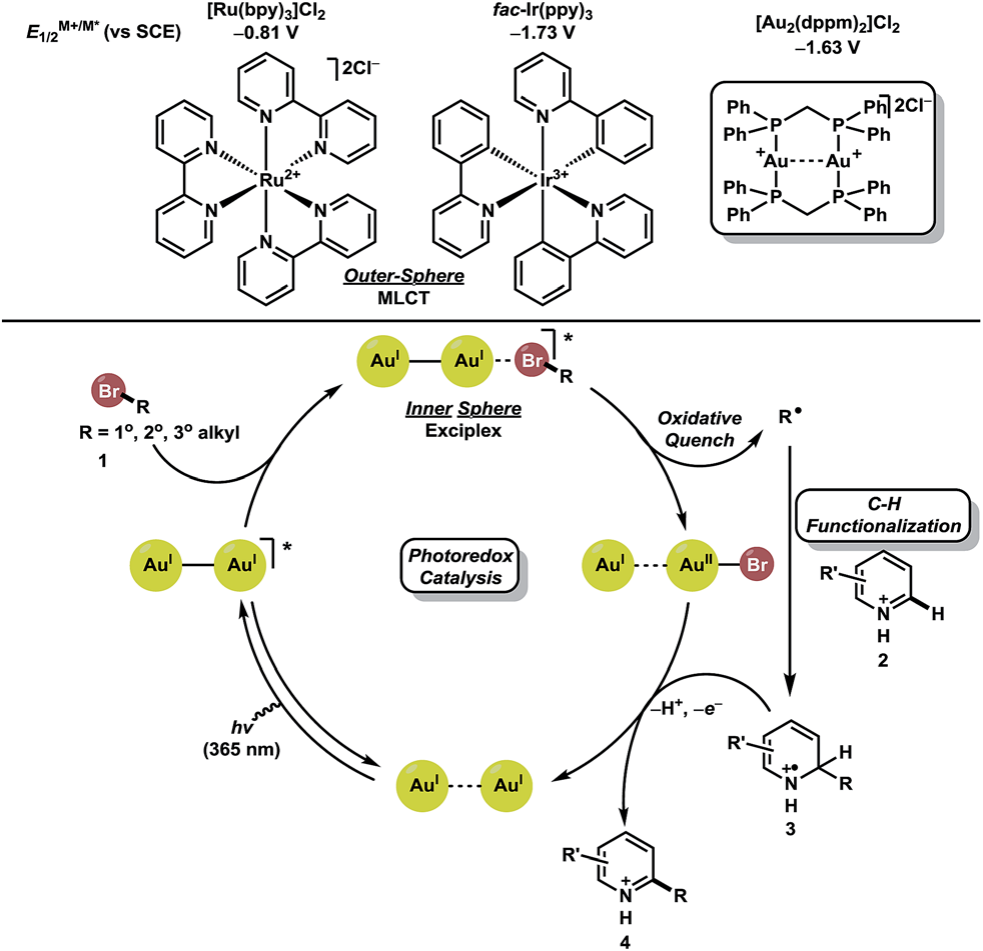
Proposed mechanism of the direct C–H alkylation of heteroarenes.

Recently, it has been shown that binuclear Au(i) phosphine complexes such as [Au_2_(dppm)_2_]Cl_2_ bear little aurophilic interaction at ground state but upon excitation, an Au–Au interaction is formed.^27^ This interaction provides an open coordination site where association of haloalkanes may occur, leading to inner sphere photoinduced electron transfer (PET) upon generation of an exciplex between the photocatalyst and bromoalkane.^25^ This phenomena allows the photocatalyst to activate bromoalkanes that have a larger reduction potential than the dimeric Au(i) complex, *vide supra*.

Initial screening of the reaction conditions were carried out with [Au_2_(dppm)_2_]Cl_2_ (2 mol%) as photocatalyst with a lepidine–TFA salt 5 along with bromocyclohexane in an array of solvents. To our delight, methanol was the optimal solvent, which upon concentrating to 0.5 M, allowed for quantitative conversion to the alkyl functionalized heteroarene product 6g with irradiation from a UVA LED (365 nm). Further optimization increased the amount of catalyst to 5 mol% which allowed shorter irradiation times to reach reaction completion and showed promise for generality towards other substrates (see ESI† for details). Blank and control experiments show the reaction does not proceed in the absence of catalyst when irradiated or in the absence of light when catalyst is present. *fac*-Ir(ppy)_3_ was evaluated as an alternative photocatalyst for this transformation under similar conditions using the lepidine–TFA salt and 2-iodopropane. Upon application of this photocatalyst, limited success was observed as an *Ir^III^/Ir^IV^ pathway may not have a sufficient redox potential to undergo quenching with iodoalkanes. Tertiary amines may be used to access an Ir^II^/Ir^III^ pathway with a larger redox potential but are not compatible under the described conditions (byproduct formation and reduction of alkyl radical intermediates).^28^ However, with use of the binuclear gold(i) photocatalyst, 2-iodopropane was converted to 6j quantitatively (see ESI† for details). This demonstrates that [Au_2_(dppm)_2_]Cl_2_ is the superior choice for this activation mode of unactivated haloalkanes in the direct alkylation of heteroarenes.

After the establishment of optimized conditions, a variety of bromoalkanes were screened (Table 1A). Direct alkylation of lepidine–TFA salt 5 with 1° bromoalkanes 1a–e gave the target compounds 6a–e in 32–77% yields. Using 2° and 3° bromoalkanes, the substituted lepidine compounds 6f–j and 6k–o were obtained in good to excellent yields. Remarkably, the addition of 1-bromoadamantane 1m proceeded in 98% yield. The robustness of this C–H functionalization was further demonstrated in the gram scale preparation of 6g in 90% yield.

**Table 1:** Substrate scope in the direct alkylation of lepidine^a^

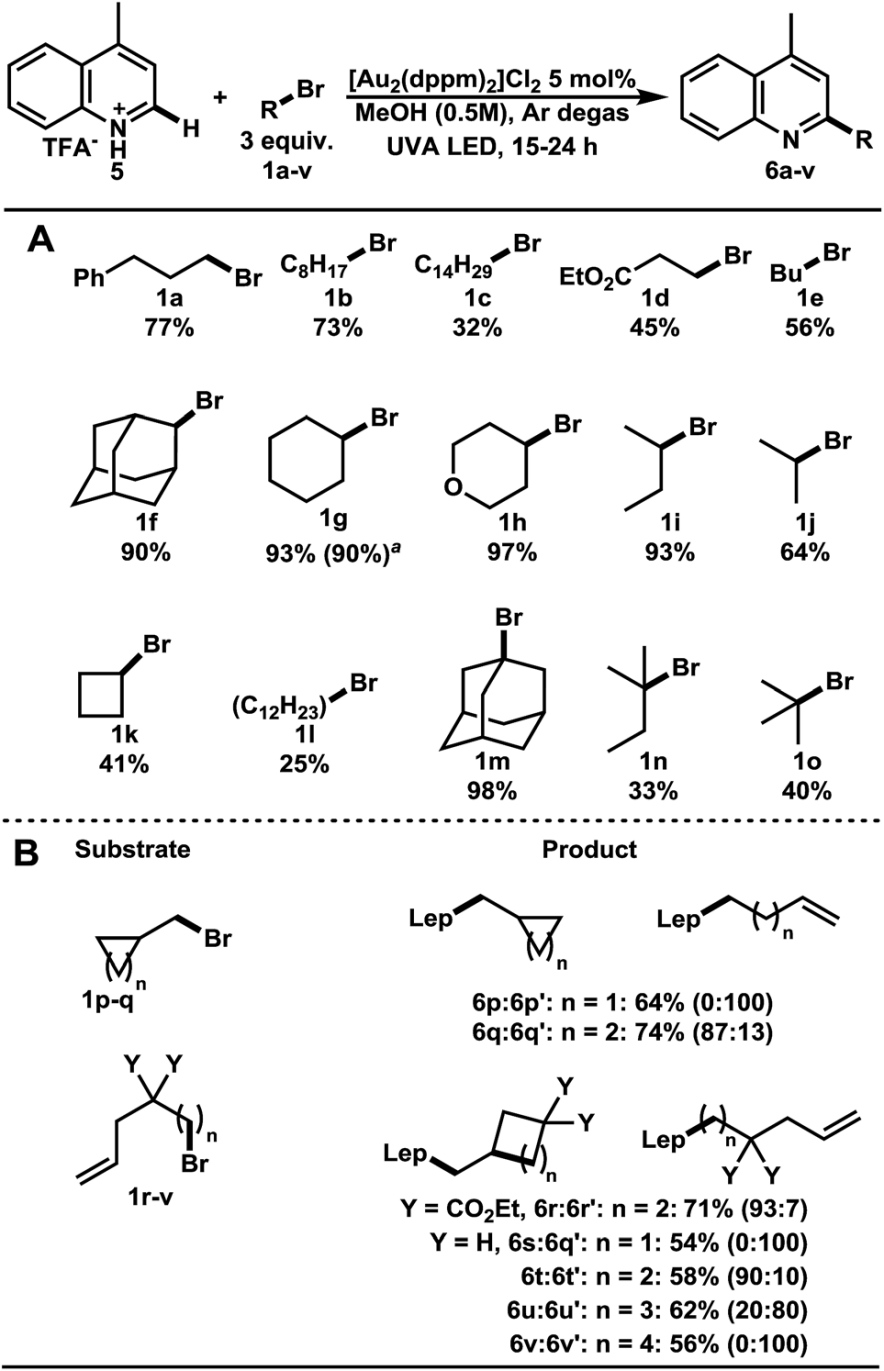

a 1 gram scale of lepidine–TFA salt, 4 equiv. CyBr, 4 UVA LEDs, 48 h. TFA = trifluoroacetic acid; Lep = lepidinyl or (4-methylquinolin-2-yl).

We then focused our efforts upon investigating ring opening and ring forming reactions characteristic of radicals (Table 1B). As expected, the (bromomethyl)cyclopropane 1p proceeded with a radical ring opening before heteroarene addition, giving 6p′ in 64% yield. (Bromomethyl)cyclobutane 1q remained mostly unopened (87 : 13, 74%). 4-Bromopentene 1s remained completely uncyclized while bromoalkenes 1r and 1t produced primary alkyl radicals that underwent 5-*exo trig* cyclization before heteroarene addition. As the chain length grew to 6 and 7 carbon spacing, 1u and 1v, the ratio of cyclized to uncyclized product diminished, 20 : 80 and 0 : 100 respectively. Each of these additions proceeded in 54–71% yields. Bromoalkanes thus proved to have broad generality in the C–H alkylation of lepidine.

As outlined in Table 2, we evaluated the generality of the heterocyclic coupling partner under the optimized reaction conditions. Alkylation of quinaldine with bromocyclohexane and 1-bromo-3-phenylpropane afforded the corresponding products in a high level of efficacy, 8 and 9 in 86% and 84% yields, respectively (Table 2A). The scope of the reaction was further extended to 7-chloroquinaldine (10, 99%), isoquinoline (11, 66%) 2,4-lutidine (12, 60%), 2,6-lutidine (13, 66%), and 2,6-diphenylpyridine (14, 98%). 3-Methylbenzoxazole underwent smooth addition (15, 98%) with bromocyclohexane under basic condition (K_2_HPO_4_). These conditions were extended to methyl indole-3-carboxylate (16, 76%), benzoxazole (19, 55%), and benzothiazole (20, 90%). Under standard conditions, bromocyclohexane also coupled well with *N*-methylbenzimidazole (17, 80%) and benzimidazole (18, 75%). Alkylation of caffeine with 1-bromoadamantane and bromocyclohexane gave C–H functionalized products in excellent yields, 21 and 22 in 95% and 92% yield, respectively. Thus, a wide variety of heterocycles were shown to undergo high yielding C–H functionalization reactions with bromoalkanes, demonstrating the potential for late-stage functionalization of many scaffolds found in nature and drug design.

**Table 2:** Substrate scope in the direct alkylation of heteroarenes and polarity reversal radical additions

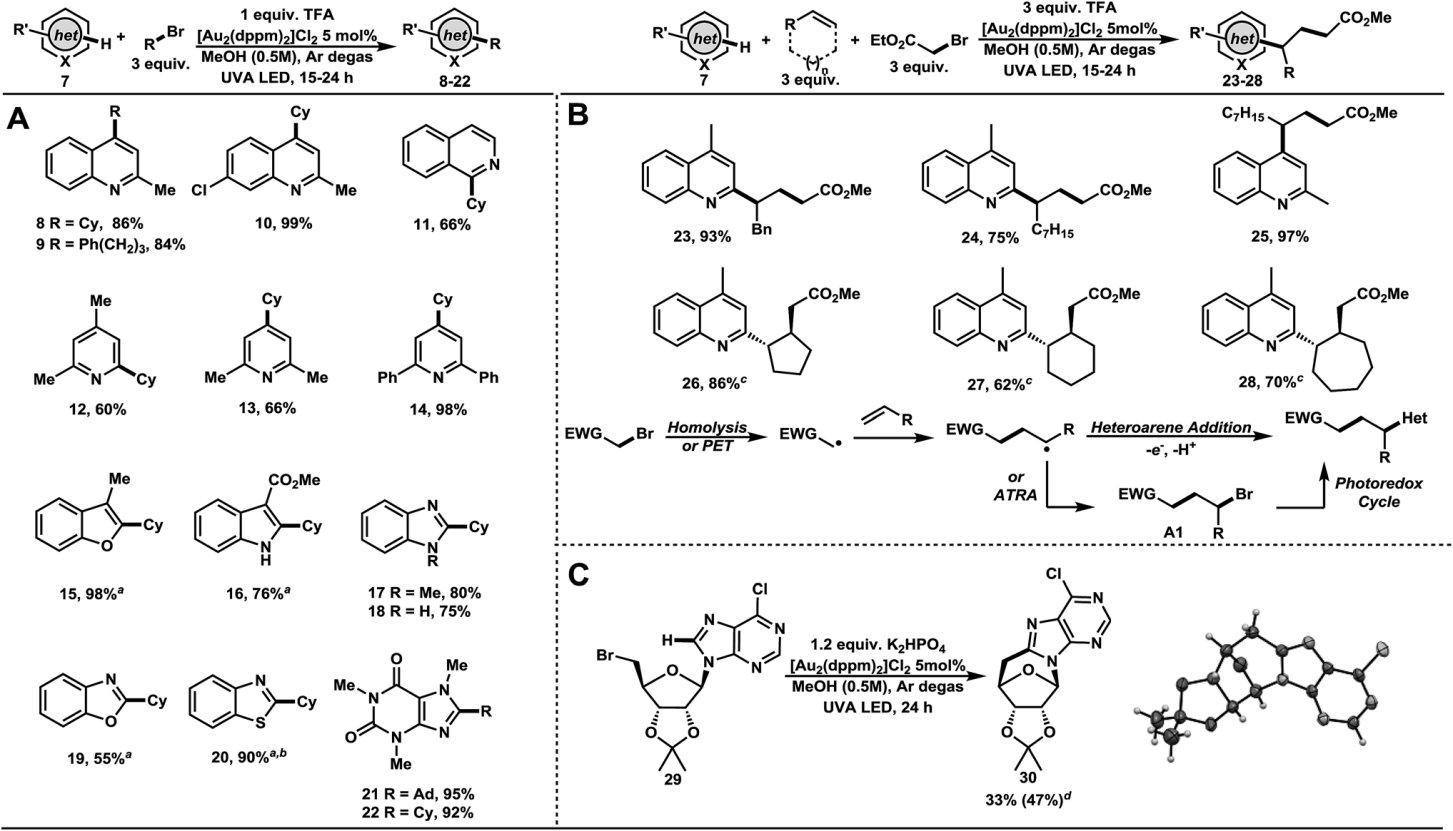

a 1.2 equiv. K_2_HPO_4_ instead of TFA.

b 0.6 mmol scale.

c d.r. > 20 : 1.

d Based on recovered starting material (31% recovered). TFA = trifluoroacetic acid; het = heteroaromatic; Ad = adamantyl; Cy = cyclohexyl.

Next, we turned our attention to the generation of electrophilic radicals from activated α-bromoesters and their application to complex motif construction (Table 2B). Harnessing the electrophilicity of radicals generated from α-bromoester 7, it was reasoned that a polarity reversal radical addition strategy as introduced by Minisci would allow this idea to be realized.^29^ Addition of an electrophilic radical to electron deficient heteroarenes is not feasible on an electronic basis. Upon addition of an acceptor alkene, the electrophilic radical becomes nucleophilic; gaining access to the C–H functionalization chemistry developed herein. Controlling the chemoselectivity of 3-component reactions is notoriously difficult, especially when considering radical transformations. Using only ethyl bromoacetate with lepidine or quinaldine furnished no functionalized products. With added alkenes, lepidine underwent smooth C–H alkylation. Thus, allylbenzene (23, 93%) 1-nonene (24, 75%), cyclopentene (26, 86%), cyclohexene (27, 62%), and cycloheptene (28, 70%) gave addition products in good to excellent yields. Addition of 1-nonene with quinaldine provided the three-component adduct 25 in 97% yield. Atom transfer radical addition reactions (ATRA) are known to be fast and efficient chain processes with activated C–Br bonds.^30^ UVA irradiation in the absence of the dimeric Au(i) photocatalyst did not produce the desired product. However, a small amount of product resulting from an ATRA pathway was observed (<10%). Also, an intramolecular example of a modified bromonucleoside 29 was amenable to addition under the described conditions, forming a surrogate version of a DNA lesion (30, 33% or 47% b.r.s.m.) (Table 2C). One can imagine the application of such a compound in the evaluation of its pharmacological properties in future studies. The successful cyclization of this sensitive and richly functionalized precursor is a testament to the mildness of these newly developed reaction conditions.

Finally, the product distribution obtained from the addition of bromoalkane 1q to lepidine was utilized to determine the absolute rate constant of 1° radical addition to lepidine (5) (Fig. 3). In this experiment, the ratios between C–H functionalization products that remained unopened *versus* those that ring opened were measured under increasing concentrations of lepidine. The results show that the absolute rate constant of addition of primary alkyl radicals to lepidine is 8.0 ± 1.3 × 10^4^ M^−1^ s^−1^. This is indicative of a free radical addition process and is comparable in rate to other known clocking studies used in heteroarene functionalization.^31^ This method could offer an alternative to photochemical radical clocking experiments using the pyridine-2-thione-*N*-oxycarbonyl (PTOC)-thiol method. The present method represents a significant improvement, since fewer synthetic steps are needed for substrate construction than PTOC esters. Furthermore, this new method produces clean results, whereas PTOC esters form many byproducts.^32^

**Fig. 3:**
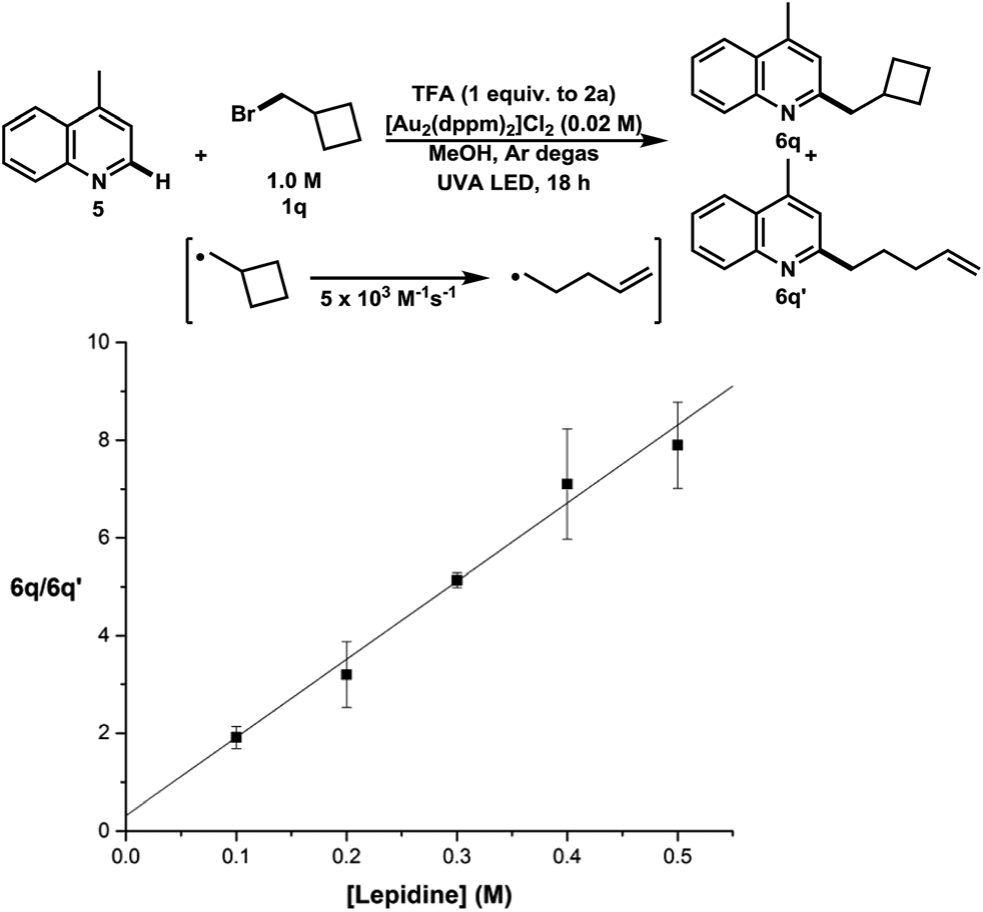
Kinetic study of the absolute rate of alkyl radical addition to lepidine, ratio of unopened to opened methylcyclobutane addition *vs.* [lepidine] (M).

## Conclusions

In summary, a protocol for direct C–H alkylation of heteroarenes was achieved using photoredox catalysis as a mode of activation for unactivated bromoalkanes. Dimeric Au(i) complex, [Au_2_(dppm)_2_]Cl_2_, served as an efficient photocatalyst for this transformation. The reaction showed robust generality in relation to the bromoalkanes and heteroarenes that were coupled in this operationally facile process. Alkyl radical polarity was also shown to play a vital role in coupling to electron deficient heteroaromatics, exemplified by polarity reversal radical addition reactions. Further studies for the development of this process in the context of late-stage functionalization of medicinally important molecules are currently underway.†

## Supplementary Material

SC-007-C6SC00807K-s001

SC-007-C6SC00807K-s002
