# Supplementary material for: Direct alkylation of heteroarenes with unactivated bromoalkanes using photoredox gold catalysis
Source: Chem Sci. 2016 Apr 18;7(7):4754–8. doi: 10.1039/c6sc00807k (PMC6016576; doi:10.1039/c6sc00807k)
Supplement: SC-007-C6SC00807K-s001 [file SC-007-C6SC00807K-s001.pdf]

Electronic Supplementary Material (ESI) for Chemical Science.
This journal is © The Royal Society of Chemistry 2016

Direct Alkylation of Heteroarenes with Unactivated Bromoalkanes using Photoredox Gold Catalysis

Terry McCallum and Louis Barriault*

Centre for Catalysis, Research and Innovation, Department of Chemistry and Biomolecular Sciences, University of Ottawa, Ottawa, Ontario, Canada.
*Correspondence to: lbarriau@uottawa.ca

Table of Contents

1. General Information 2

2. General Procedures 3

GP1- Preparation of Alkylated Heteroarenes from Bromoalkanes 3

GP2- Preparation of Alkylated Heteroarenes via Polarity Reversal Radical Addition . . . 3

GP3- Kinetic Study using (bromomethyl)cyclobutane 3

3. Optimization and Comparative Catalyst Studies 4

4. Product Characterization 6

5. Spectra 23

6. X-Ray Diffraction Data 113

1. General Information

All reactions were performed under argon atmosphere Pyrex glassware equipped with a magnetic stir bar, capped with a septum, unless otherwise indicated. All commercial reagents were used without further purification, unless otherwise noted. Reactions were monitored by thin layer chromatography (TLC) analysis. TLC plates were viewed under UV light and stained with potassium permanganate or p-anisaldehyde staining solution. Yields refer to products isolated after purification, unless otherwise stated. Proton nuclear magnetic resonance (1H NMR) spectra were recorded on a Bruker AMX 400 MHz. NMR samples were dissolved in chloroform-d (unless specified otherwise) and chemical shifts are reported in ppm referenced to residual undeuterated solvent. Data are reported as follows: chemical shift, multiplicity, coupling, integration, where multiplicity is as follows : s = singlet, d = doublet, dd = doublet of doublets, ddd = doublet of doublets of doublets, dt = doublet of triplets, ddt = doublet of doublets of triplets, dq = doublet of quartets, dquin = doublet of quintets, br = broad signal, t = triplet, td = triplet of doublets, tt = triplet of triplets, tq = triplet of quartets, tquin = triplet of quintets, q = quartet, qd = quartet of doublets, quin = quintet, spt = septet, m = multiplet, or otherwise noted. Carbon nuclear magnetic resonance (13C NMR) spectra were recorded on the same Bruker instruments as in proton NMR using 75 MHz or 101 MHz. IR spectra were recorded with an Agilent Technologies Cary 630 FTIR Spectrometer equipped with a diamond ATR module. HRMS were obtained on a Kratos Analytical Concept instrument (University of Ottawa Mass Spectrum Centre).

2. General Procedures

General Procedure 1 (GP1). *Preparation of alkylated heteroarenes from bromoalkanes.* To an 8 mL pyrex screw-top reaction vessel was added the TFA salt of heterocycle (0.2-0.3 mmol, 1.0 eq, isolated by adding 1.0 eq of TFA to heterocycle in DCM and concentrating *in vacuo*), then bromoalkane (0.6-0.9 mmol, 3.0 eq), $[Au_2(dppm)_2]Cl_2$ (0.010-0.015 mmol, 0.05 eq) and methanol (0.4-0.6 mL, 0.5 M). If heterocycle was added without being a TFA salt, TFA was added last (0.2-0.3 mmol, 1.0 eq).

*In certain cases, reaction was run under basic condition by adding K_2HPO_4 (0.24-0.36 mmol, 1.2 eq) instead of TFA.

The reaction mixture was degassed with argon by sparging, and irradiated with a UVA (365 nm) LED at a distance of a centimetre, for 15 to 24 hours. The resulting mixture was concentrated, dissolved in methylene chloride and poured into a seperatory funnel under basic aqueous work-up (saturated sodium bicarbonate or 1 M sodium hydroxide), extracted 3 times with methylene chloride, where the organic fractions were combined, dried over sodium sulfate, and concentrated *in vacuo*. Crude product was further purified by flash chromatography, where relevant fractions were combined, concentrated and characterized by proton and carbon NMR (400 and 101 MHz, respectively), HR-MS, and IR.

General Procedure 2 (GP2). *Preparation of alkylated heteroarenes via polarity reversal radical addition.* To an 8 mL pyrex screw-top reaction vessel was added the heterocycle (0.2-0.3 mmol, 1.0 eq), methanol (0.4-0.6 mL, 0.5 M), TFA (0.6-0.9 mmol, 3.0 eq), $[Au_2(dppm)_2]Cl_2$ (0.010-0.015 mmol, 0.05 eq), then alkene (0.6-0.9 mmol, 3.0 eq) and ethyl bromoacetate (0.6-0.9 mmol, 3.0 eq). The reaction mixture was degassed with argon by sparging and irradiated with a UVA (365 nm) LED at a distance of a centimetre for 15 to 24 hours.

*Alkenes with very low boiling points (e.g. cyclohexene) were added after sparging the mixture. The resulting mixture was concentrated, dissolved in methylene chloride and poured into a seperatory funnel under basic aqueous work-up (saturated sodium bicarbonate), extracted 3 times with methylene chloride, where the organic fractions were combined, dried over sodium sulfate, and concentrated *in vacuo*. Crude product was further purified by flash chromatography, where relevant fractions were combined, concentrated and characterized by proton and carbon NMR (400 and 101 MHz, respectively), HR-MS, and IR.

General Procedure 3 (GP3). *Kinetic study using (bromomethyl)cyclobutane*. Reactions were prepared from stock solutions of lepidine (1.0 M), TFA (2.0 M), $[Au_2(dppm)_2]Cl_2$ (0.2 M) in MeOH and (bromomethyl)cyclobutane (neat). In a given run, 5 samples were prepared using the stock solutions of lepidine (100-500 μL, 0.1-0.5 M), TFA (50-250 μL, 0.1-0.5 M), $[Au_2(dppm)_2]Cl_2$ (100 μL, 0.02 M each), and reactions were degassed lightly with argon for 5 minutes. (bromomethyl)cyclobutane (113 μL, 1.0 M each) was then added and each solution was added the remaining MeOH to make a 1 mL volume (637μL, 487 μL, 337 μL, 187 μL, 37 μL, respectively). The reactions were then irradiated for 18 hours with a UVA LED. The resulting mixtures were then subjected to the work-up portion of GP1. This was done because the crude products remained protonated after irradiation, however, the ^{1}H NMR after irradiation showed the same ratio of products as the ^{1}H NMR of material that had been worked up (also having the same ratio after flash chromatography). The worked up products could then be analyzed by ^{1}H NMR reliably for product ratios (GC studies were not reliable for this experiment at present). Reactions ranged from 40% to complete conversion.

3. Optimization and Comparative Catalyst Studies

Table S1. Optimization of direct C-H alkylation of heteroarenes from bromoalkanes.

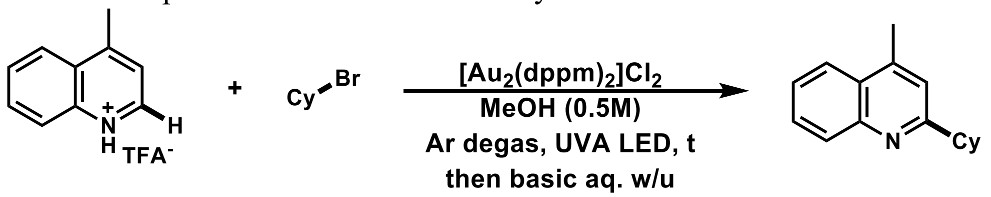

Entry	Photocatalyst [mol%]	Bromo [eq]	Time [h]	Yield [%][a]
1	2	3	13	79
2	2	3	26	99
3	2	1	48	60
4	1	3	13	64
**5**	**5**	**3**	**13**	**99(93)**
6	---	3	13	<5
7	5	3	13	0[b]
8	5	3	6.5	71
9	5	3	18	99[c]
10	5	3	18	21[d]
11	5	2	18	78
12	5	1	18	46
13	5	3	24	99[e]
14	5	4	48	99(90)[f]

* Optimization conducted on 0.2 mmol scale of heteroaromatic salt following GP1. NMR conversion (isolated yield). [b] Not irradiated with light and heated to 80°C. [c] Lepidine used instead of TFA salt. [d] Lepidine with 1.2 equivalents of potassium phosphate dibasic added. [e] 1.0 mmol scale. [f] 1.0 g scale of TFA salt, 4 X UVA LEDs.

Table S2. Comparison of photoredox catalysts and haloalkanes.

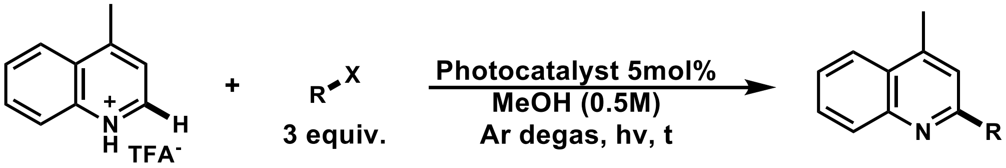

Entry	Photocatalyst	RX	K_2HPO_4 [eq]	hv [nm]	t	Yield [%][a]
1	$[Au_2(dppm)_2]Cl_2$	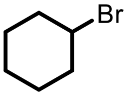	---	365	20	99(93)
2	*fac*-Ir(ppy)$_3$		2.0	410	20	0
3	*fac*-Ir(ppy)$_3$		---	410	20	0
4	$[Ir(dtbbpy)_2(ppy)](PF_6)_2$		2.0	410	20	0
**5**	**$[Au_2(dppm)_2]Cl_2$**	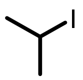	**---**	**365**	**20**	**99**
6	None		2.0	410	20	0
7	None		---	365	20	0

[a] Experiments conducted on 0.2 mmol scale of heteroaromatic salt following GP1. Reactions irradiated with 365 or 410 nm LEDs. For reactions containing K_2HPO_4, lepidine was used instead of the TFA salt. NMR conversion (isolated yield).

4. Product Characterization

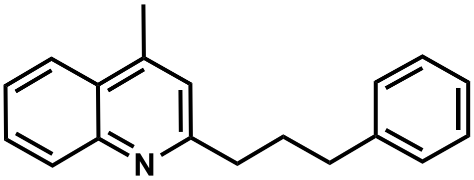

4-methyl-2-(3-phenylpropyl)quinoline (6a)

Synthesized according to GP1.

IR (neat, cm^{-1}): 2925(m), 2857(m), 1603(s), 1449(s), 758(vs), 700(s); **^{1}H NMR** (400 MHz, $CDCl_3$) δ 8.07 (d, *J*=8.3 Hz, 1H), 7.96 (d, *J* = 8.3 Hz, 1H), 7.69 (ddd, *J* = 8.2, 6.7, 1.2 Hz, 1H), 7.52 (ddd, *J* = 8.0, 6.7, 1.0 Hz, 1H), 7.33-7.27 (m, 2H), 7.26-7.17 (m, 3H), 7.14 (s, 1H), 2.99 (t, *J* = 7.7 Hz, 2H), 2.76 (t, *J* = 7.7 Hz, 2H), 2.68 (s, 3H), 2.17 (quin, *J* = 7.9 Hz, 2H) ppm; **^{13}C NMR** (101 MHz, $CDCl_3$) δ 162.1 (C), 147.7 (C), 144.2 (C), 142.1 (C), 129.3 (CH), 129.0 (CH), 128.5 (2 X CH), 128.3 (2 X CH), 126.8 (C), 125.7 (CH), 125.4 (CH), 123.6 (CH), 122.0 (CH), 38.7 (CH_2), 35.7 (CH_2), 31.6 (CH_2), 18.6 (CH_3) ppm; **HRMS (EI)** m/z calc'd for $C_{19}H_{19}N$ [M^+] 261.1517, found 261.1515.

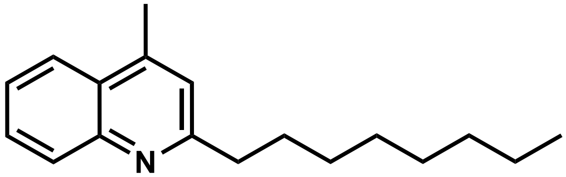

4-methyl-2-octylquinoline (6b)

Synthesized according to GP1.

IR (neat, cm^{-1}): 2922(vs), 2853(m), 1603(s), 1449(m), 756(vs); **^{1}H NMR** (400 MHz, $CDCl_3$) δ 8.05 (d, *J* = 8.4 Hz, 1H), 7.95 (dd, *J* = 8.2, 0.8 Hz, 1H), 7.67 (ddd, *J* = 8.4, 6.9, 1.3 Hz, 1H), 7.50 (ddd, *J* = 8.2, 7.0, 1.2 Hz, 1H), 7.14 (s, 1H), 2.94-2.90 (m, 2H), 2.68 (d, *J* = 0.6 Hz, 3H), 1.80 (quin, *J* = 7.7 Hz, 2H), 1.42 (quin, *J* = 7.7 Hz, 2H), 1.38-1.25 (m, 8H), 0.88 (t, *J* = 7.0 Hz, 3H) ppm; **^{13}C NMR** (101 MHz, $CDCl_3$) δ 162.8 (C), 147.7 (C), 144.1 (C), 129.3 (CH), 128.9 (CH), 126.7 (C), 125.3 (CH), 123.5 (CH), 122.0 (CH), 39.3 (CH_2), 31.8 (CH_2), 30.1 (CH_2), 29.6 (CH_2), 29.5 (CH_2), 29.2 (CH_2), 22.6 (CH_2), 18.6 (CH_3), 14.1 (CH_3) ppm; **HRMS (EI)** m/z calc'd for $C_{18}H_{25}N$ [M^+] 255.1987 found 255.1966.

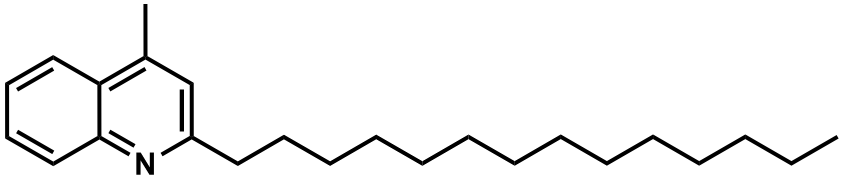

4-methyl-2-tetradecylquinoline (6c)

Synthesized according to GP1.

IR (neat, cm^{-1}): 2922(s), 2852(s) 1604(m), 1449(m), 756(s); **^{1}H NMR** (400 MHz, $CDCl_3$) δ 8.06 (d, *J* = 8.2 Hz, 1H), 7.96 (dd, *J* = 8.2, 0.9 Hz, 1H), 7.68 (ddd, *J* = 8.4, 6.9, 1.4 Hz, 1H), 7.51 (ddd, *J* = 8.2, 7.0, 1.2 Hz, 1H), 7.15 (s, 1H), 2.93 (t, *J* = 7.9 Hz, 2H), 2.69 (d, *J* = 0.8 Hz, 3H), 1.80 (quin, *J* = 7.7 Hz, 2H), 1.42 (quin, *J* = 7.7 Hz, 2H), 1.34-1.20 (m, 20H), 0.89 (t, *J* = 6.7 Hz, 3H) ppm; **^{13}C NMR** (101 MHz, $CDCl_3$) δ 162.8 (C), 147.7 (C), 144.1 (C), 129.3 (CH), 129.0 (CH), 126.8 (C), 125.4 (CH), 123.6 (CH), 122.1 (CH), 39.3 (CH_2), 31.9 (CH_2), 30.1 (CH_2), 29.7 (2 X CH_2), 29.6 (4 X CH_2), 29.5 (2 X CH_2), 29.3 (CH_2), 22.7 (CH_2), 18.7 (CH_3), 14.1 (CH_3) ppm; **HRMS (EI)** m/z calc'd for $C_{24}H_{37}N$ [M^+] 339.2926, found 339.2931.

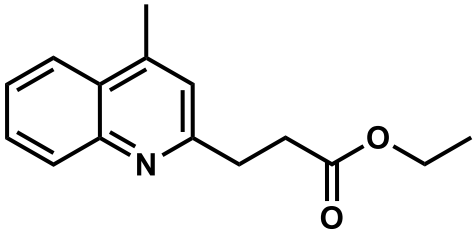

ethyl 3-(4-methylquinolin-2-yl)propanoate (6d)
Synthesized according to GP1 and characterized according to NMR comparison (*14*).
^{1}H NMR (400 MHz, $CDCl_3$) δ 8.02 (d, *J* = 8.4 Hz, 1H), 7.96 (dd, *J* = 8.3, 1.0 Hz, 1H), 7.68 (ddd, *J* = 8.4, 6.9, 1.4 Hz, 1H), 7.51 (ddd, *J* = 8.3, 7.0, 1.2 Hz, 1H), 7.18 (s, 1H), 4.15 (q, *J* = 7.2 Hz, 2H), 3.26 (t, *J* = 7.5 Hz, 2H), 2.91 (t, *J* = 7.5 Hz, 2H), 2.68 (d, *J* = 0.9 Hz, 3H), 1.25 (t, *J* = 7.1 Hz, 3H) ppm; **^{13}C NMR** (101 MHz, $CDCl_3$) δ 173.2 (C), 160.2 (C), 147.6 (C), 144.4 (C), 129.4 (CH), 129.1 (CH), 126.9 (C), 125.6 (CH), 123.6 (CH), 122.2 (CH), 60.4 (CH_2), 33.4 (CH_2), 33.3 (CH_2), 18.6 (CH_3), 14.2 (CH_3) ppm.

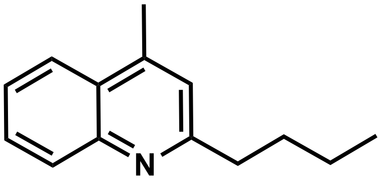

2-butyl-4-methylquinoline (6e)
Synthesized according to GP1 and characterized according to NMR comparison (*32*).
^{1}H NMR (400 MHz, $CDCl_3$) δ 8.06 (d, *J* = 8.3 Hz, 1H), 7.96 (dd, *J* = 8.3, 1.1 Hz, 1H), 7.68 (ddd, *J* = 8.4, 6.9, 1.4 Hz, 1H), 7.51 (ddd, *J* = 8.2, 7.0, 1.2 Hz, 1H), 7.16 (s, 1H), 2.98-2.89 (m, 2H), 2.69 (d, *J* = 0.7 Hz, 3H), 1.85-1.75 (m, 2H), 1.46 (sxt, *J* = 7.4 Hz, 2H), 0.97 (t, *J* = 7.3 Hz, 3H) ppm; **^{13}C NMR** (101 MHz, $CDCl_3$) δ 162.7 (C), 147.6 (C), 144.1 (C), 129.2 (CH), 129.1 (CH), 126.8 (C), 125.4 (CH), 123.6 (CH), 122.1 (CH), 38.9 (CH_2), 32.2 (CH_2), 22.7 (CH_2), 18.7 (CH_3), 14.0 (CH_3) ppm.

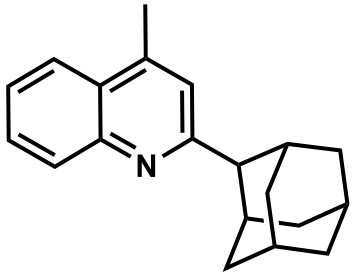

2-((1R,3S,5r,7r)-adamantan-2-yl)-4-methylquinoline (6f)
Synthesized according to GP1.
IR (neat, cm^{-1}): 2902(s), 2848(m), 1602(m), 1449(m), 755(m); **^{1}H NMR** (400 MHz, $CDCl_3$) δ 8.07 (d, *J* = 8.4 Hz, 1H), 7.96 (d, *J* = 8.1 Hz, 1H), 7.67 (t, *J* = 7.4 Hz, 1H), 7.50 (t, *J* = 7.5 Hz, 1H), 7.29 (s, 1H), 3.25-3.16 (m, 1H), 2.83 - 2.75 (m, 2H), 2.74-2.65 (m, 3H), 2.10-1.93 (m, 7H), 1.82 (s, 3H), 1.63 (d, *J* = 12.2 Hz, 2H) ppm; **^{13}C NMR** (101 MHz, $CDCl_3$) δ 164.1 (C), 147.7 (C), 143.3 (C), 129.8 (CH), 128.6 (CH), 126.4 (C), 125.3 (CH), 123.4 (CH), 120.5 (CH), 50.4 (CH), 39.1 (2 X CH_2), 37.9 (CH_2), 32.6 (2 X CH_2), 30.9 (2 X CH), 28.0 (CH), 27.9 (CH), 18.9 (CH_3) ppm; **HRMS (EI)** m/z calc'd for $C_{20}H_{23}N$ [M^+] 277.1830, found 277.1841.

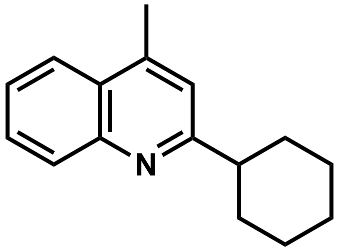

2-cyclohexyl-4-methylquinoline (6g)
Synthesized according to GP1 and characterized according to NMR comparison (*16*).
^{1}H NMR (400 MHz, $CDCl_3$) δ 8.05 (d, J = 8.3 Hz, 1H), 7.95 (dd, J = 8.3, 1.0 Hz, 1H), 7.67 (ddd, J = 8.4, 6.9, 1.4 Hz, 1H), 7.50 (ddd, J = 8.3, 6.9, 1.2 Hz, 1H), 7.18 (s, 1H), 2.88 (tt, J = 12.0, 3.3 Hz, 1H), 2.69 (d, J = 0.8 Hz, 3H), 2.05-1.99 (m, 2H), 1.90 (dt, J = 12.9, 3.1 Hz, 2H), 1.83-1.77 (m, 1H), 1.63 (qd, J = 12.5, 2.9 Hz, 2H (+1H from H_2O in spectrum)), 1.48 (qt, J = 12.7, 3.1 Hz, 2H), 1.36 (tt, J = 12.5, 3.2 Hz, 1H) ppm; **^{13}C NMR** (101 MHz, $CDCl_3$) δ 166.5 (C), 147.6 (C), 144.3 (C), 129.4 (CH), 129.0 (CH), 127.0 (C), 125.4 (CH), 123.5 (CH), 120.2 (CH), 47.6 (CH), 32.8 (2 X CH_2), 26.6 (2 X CH_2), 26.1 (CH_2), 18.8 (CH_3) ppm.

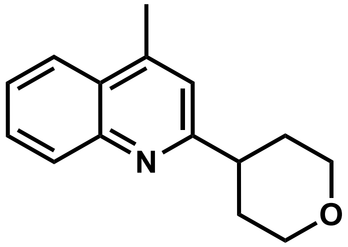

4-methyl-2-(tetrahydro-2H-pyran-4-yl)quinoline (6h)
Synthesized according to GP1 and characterized according to NMR comparison (*16*).
^{1}H NMR (400 MHz, $CDCl_3$) δ 8.05 (d, J = 8.3 Hz, 1H), 7.96 (dd, J = 8.4, 0.8 Hz, 1H), 7.69 (ddd, J = 8.3, 7.0, 1.3 Hz, 1H), 7.52 (ddd, J = 8.2, 7.6, 1.1 Hz, 1H), 7.18 (s, 1H), 4.14 (dd, J = 11.2, 4.3 Hz, 2H), 3.60 (td, J = 11.7, 2.2 Hz, 2H), 3.13 (tt, J = 11.8, 4.0 Hz, 1H), 2.70 (s, 3H), 2.03 (qd, J = 12.7, 4.5 Hz, 2H), 1.95 - 1.89 (m, 2H) ppm; **^{13}C NMR** (101 MHz, $CDCl_3$) δ 164.2 (C), 147.6 (C), 144.6 (C), 129.5 (CH), 129.1 (CH), 127.1 (C), 125.6 (CH), 123.6 (CH), 119.9 (CH), 68.1 (2 X CH_2), 44.4 (CH), 32.3 (2 X CH_2), 18.8 (CH_3) ppm.

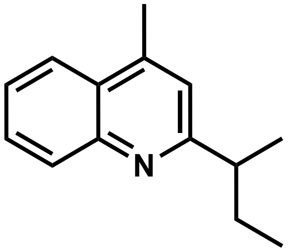

2-(sec-butyl)-4-methylquinoline (6i)
Synthesized according to GP1 and characterized according to NMR comparison (*16*).
^{1}H NMR (400 MHz, $CDCl_3$) δ 8.07 (d, J = 8.4 Hz, 1H), 7.95 (dd, J = 8.2, 0.9 Hz, 1H), 7.67 (ddd, J = 8.4, 7.0, 1.3 Hz, 1H), 7.50 (ddd, J = 8.2, 7.0, 1.2 Hz, 1H), 7.14 (s, 1H), 2.97 (sxt, J = 7.1 Hz, 1H), 2.69 (d, J = 0.7 Hz, 3H), 1.86 (dquin, J = 13.9, 7.2 Hz, 1H), 1.72 (dquin, J = 13.9, 7.2 Hz, 1H), 1.37 (d, J = 7.0 Hz, 3H), 0.91 (t, J = 7.4 Hz, 3H) ppm; **^{13}C NMR** (101 MHz, $CDCl_3$) δ 166.7 (C), 147.6 (C), 144.1 (C), 129.5 (CH), 128.8 (CH), 127.0 (C), 125.3 (CH), 123.5 (CH), 120.1 (CH), 44.5 (CH), 29.9 (CH_2), 20.4 (CH_3), 18.8 (CH_3), 12.2 (CH_3) ppm.

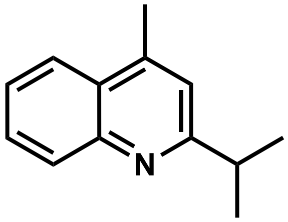

2-isopropyl-4-methylquinoline (6j)
Synthesized according to GP1 and characterized according to NMR comparison (*16*).
^{1}H NMR (400 MHz, $CDCl_3$) δ 8.06 (dd, *J* = 8.5, 0.7 Hz, 1H), 7.95 (dd, *J* = 8.3, 0.9 Hz, 1H), 7.67 (ddd, *J* = 8.4, 6.9, 1.4 Hz, 1H), 7.50 (ddd, *J* = 8.3, 6.9, 1.3 Hz, 1H), 7.18 (d, *J* = 0.8 Hz, 1H), 3.23 (spt, *J* = 7.0 Hz, 1H), 2.69 (d, *J* = 0.9 Hz, 3H), 1.40 (d, *J* = 7.0 Hz, 6H) ppm; **^{13}C NMR** (101 MHz, $CDCl_3$) δ 167.3 (C), 147.5 (C), 144.3 (C), 129.5 (CH), 128.9 (CH), 127.0 (C), 125.4 (CH), 123.5 (CH), 119.7 (CH), 37.2 (CH), 22.5 (2 X CH_3), 18.8 (CH_3) ppm.

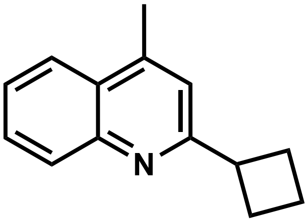

2-cyclobutyl-4-methylquinoline (6k)
Synthesized according to GP1 and characterized according to NMR comparison (*16*).
^{1}H NMR (400 MHz, $CDCl_3$) δ 8.07 (d, *J* = 8.4 Hz, 1H), 7.95 (dd, *J* = 8.2, 0.7 Hz, 1H), 7.68 (ddd, *J* = 8.2, 6.9, 1.3 Hz, 1H), 7.50 (ddd, *J* = 8.1, 7.0, 1.1 Hz, 1H), 7.21 (s, 1H), 3.84 (quin, *J* = 8.7 Hz, 1H), 2.70 (s, 3H), 2.50 - 2.42 (m, 4H), 2.19 - 2.07 (m, 1H), 2.00-1.91 (m, 1H) ppm; **^{13}C NMR** (101 MHz, $CDCl_3$) δ 164.7 (C), 147.5 (C), 144.1 (C), 129.5 (CH), 128.9 (CH), 126.9 (C), 125.4 (CH), 123.5 (CH), 120.2 (CH), 42.6 (CH), 28.2 (2 X CH_2), 18.8 (CH_3), 18.3 (CH_2) ppm.

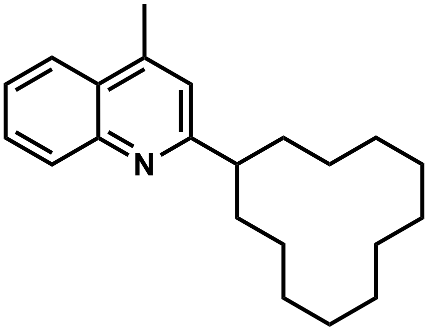

2-cyclododecyl-4-methylquinoline (6l)
Synthesized according to GP1.
IR (neat, cm^{-1}): 2929(vs), 2861(m), 1603(m), 1445(m), 757(s); **^{1}H NMR** (400 MHz, $CDCl_3$) δ 8.23 (m, 1H), 7.97 (d, *J* = 8.2 Hz, 1H), 7.71 (t, *J* = 7.6 Hz, 1H), 7.54 (t, *J* = 7.3 Hz, 1H), 7.19 (s, 1H), 3.23 (s, 1H), 2.72 (s, 3H), 2.01-1.88 (m, 2H), 1.74 (td, *J* = 12.8, 5.7 Hz, 2H), 1.65-1.32 (m, 18H) ppm; **^{13}C NMR** (101 MHz, $CDCl_3$) δ 166.4 (C), 148.0 (C), 143.2 (C), 129.5 (CH), 128.8 (CH), 126.9 (C), 125.8 (CH), 123.6 (CH), 121.3 (CH), 42.6 (CH), 30.1 (2 X CH_2), 23.9 (2 X CH_2), 23.8 (2 X CH_2), 23.7 (2 X CH_2), 23.4 (CH_2), 22.8 (2 X CH_2), 19.0 (CH_3) ppm; **HRMS (EI)** m/z calc'd for $C_{22}H_{31}N$ [M^+] 309.2457, found 309.2444.

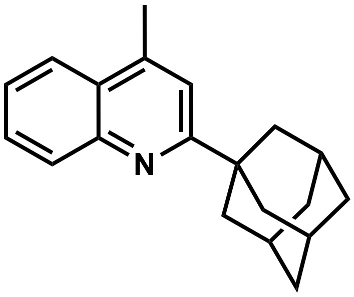

2-((1s,3s)-adamantan-1-yl)-4-methylquinoline (6m)
Synthesized according to GP1 and characterized according to NMR comparison (*20d*).
^{1}H NMR (400 MHz, $CDCl_3$) δ 8.07 (d, *J* = 8.4 Hz, 1H), 7.95 (dd, *J* = 8.3, 1.0 Hz, 1H), 7.66 (ddd, *J* = 8.4, 6.9, 1.4 Hz, 1H), 7.49 (ddd, *J* = 8.2, 6.9, 1.2 Hz, 1H), 7.34 (s, 1H), 2.70 (d, *J* = 0.8 Hz, 3H), 2.16 (s, 3H), 2.13 (d, *J* = 2.3 Hz, 6H), 1.84 (t, *J* = 2.8 Hz, 6H) ppm; **^{13}C NMR** (101 MHz, $CDCl_3$) δ 168.7 (C), 147.5 (C), 143.5 (C), 130.0 (CH), 128.6 (CH), 126.7 (C), 125.3 (CH), 123.4 (CH), 118.5 (CH), 41.8 (3 X CH_2), 39.5 (C), 36.9 (3 X CH_2), 28.8 (3 X CH), 19.0 (CH_3) ppm.

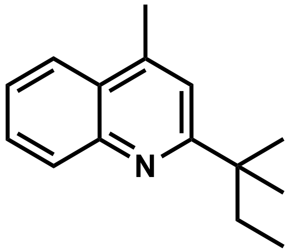

4-methyl-2-(tert-pentyl)quinoline (6n)
Synthesized according to GP1.
IR (neat, cm^{-1}): 2964(s), 2924(m), 1602(m), 1448(m), 757(vs); **^{1}H NMR** (400 MHz, $CDCl_3$) δ 8.07 (d, *J* = 8.3 Hz, 1H), 7.95 (dd, *J* = 8.2, 1.0 Hz, 1H), 7.67 (ddd, *J* = 7.6, 1.3 Hz, 1H), 7.53-7.47 (m, 1H), 7.31 (s, 1H), 2.70 (d, *J* = 0.6 Hz, 3H), 1.86 (q, *J* = 7.4 Hz, 2H), 1.44 (s, 6H), 0.75 (t, *J* = 7.4 Hz, 3H) ppm; **^{13}C NMR** (101 MHz, $CDCl_3$) δ 168.0 (C), 147.4 (C), 143.3 (C), 130.0 (CH), 128.6 (CH), 126.5 (C), 125.3 (CH), 123.4 (CH), 119.4 (CH), 41.2 (C), 35.8 (CH_2), 27.3 (2 X CH_3), 19.0 (CH_3), 9.2 (CH_3) ppm; **HRMS (EI)** m/z calc'd for $C_{15}H_{19}N$ [M^+] 213.1517, found 213.1520.

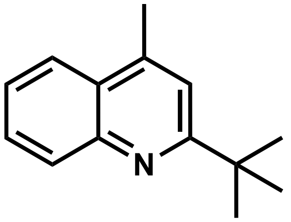

2-(tert-butyl)-4-methylquinoline (6o)
Synthesized according to GP1 and characterized according to NMR comparison (*32*).
^{1}H NMR (400 MHz, $CDCl_3$) δ 8.06 (d, *J* = 8.3 Hz, 1H), 7.95 (dd, *J* = 8.2, 0.8 Hz, 1H), 7.66 (ddd, *J* = 8.3, 7.0, 1.3 Hz, 1H), 7.50 (ddd, *J* = 8.2, 6.9, 1.2 Hz, 1H), 7.36 (s, 1H), 2.70 (d, *J* = 0.7 Hz, 3H), 1.47 (s, 9H) ppm; **^{13}C NMR** (101 MHz, $CDCl_3$) δ 168.9 (C), 147.3 (C), 143.6 (C), 130.0 (CH), 128.7 (CH), 126.5 (C), 125.4 (CH), 123.4 (CH), 118.9 (CH), 37.9 (C), 30.1 (3 X CH_3), 18.9 (CH_3) ppm.

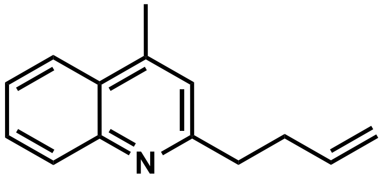

2-(but-3-en-1-yl)-4-methylquinoline (6p')

Synthesized according to GP1.

IR (neat, cm^{-1}): 3068(m), 2923(m), 2854(m), 1641(m), 1604(s), 1448(m), 997(m), 913(m), 758(vs); **^{1}H NMR** (400 MHz, $CDCl_3$) δ 8.05 (d, *J* = 8.4 Hz, 1H), 7.96 (dd, *J* = 8.3, 0.9 Hz, 1H), 7.68 (ddd, *J* = 8.4, 7.0, 1.3 Hz, 1H), 7.51 (ddd, *J* = 8.2, 7.0, 1.1 Hz, 1H), 7.15 (s, 1H), 5.94 (ddt, *J* = 17.0, 10.3, 6.6 Hz, 1H), 5.10 (dq, *J* = 17.0, 1.6 Hz, 1H), 5.00 (dq, *J* = 10.2, 1.6 Hz, 1H), 3.03 (dd, *J* = 9.3, 7.6 Hz, 2H), 2.68 (d, *J* = 0.6 Hz, 3H), 2.63-2.55 (m, 2H) ppm; **^{13}C NMR** (101 MHz, $CDCl_3$) δ 161.7 (C), 147.7 (C), 144.2 (C), 137.8 (CH), 129.3 (CH), 129.0 (CH), 126.8 (C), 125.5 (CH), 123.6 (CH), 122.1 (CH), 115.1 (CH_2), 38.4 (CH_2), 33.8 (CH_2), 18.7 (CH_3) ppm; **HRMS (EI)** m/z calc'd for $C_{14}H_{15}N$ [M^+] 197.1204, found 197.1190.

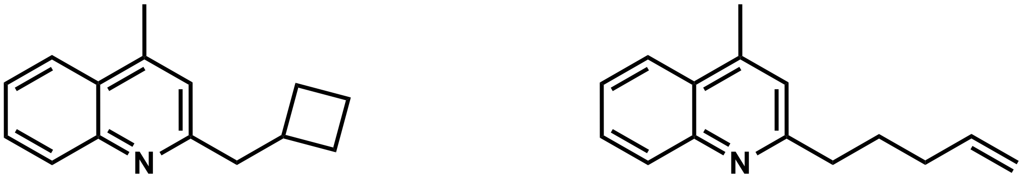

2-(cyclobutylmethyl)-4-methylquinoline/4-methyl-2-(pent-4-en-1-yl)quinoline (87:13) (6q:6q')

Synthesized according to GP1.

IR (neat, cm^{-1}): 2933(m), 2857(m), 1603(s), 1447(m), 757(vs);

6q: ^{1}H NMR (400 MHz, $CDCl_3$) δ 8.05 (d, *J* = 8.3 Hz, 1H), 7.95 (d, *J* = 8.3 Hz, 1H), 7.67 (ddd, *J* = 8.3, 7.0, 1.3 Hz, 1H), 7.50 (ddd, *J* = 8.1, 6.9, 1.1 Hz, 1H), 7.09 (s, 1H), 3.03 (d, *J* = 7.6 Hz, 2H), 2.82 (spt, *J* = 7.9 Hz, 1H), 2.67 (s, 3H), 2.13-2.01 (m, 2H), 1.97-1.79 (m, 4H) ppm; **^{13}C NMR** (101 MHz, $CDCl_3$) δ 161.3 (C), 147.7 (C), 144.0 (C), 129.4 (CH), 128.9 (CH), 126.8 (C), 125.3 (CH), 123.5 (CH), 122.1 (CH), 46.0 (CH_2), 36.2 (CH), 28.3 (2 X CH_2), 18.7 (CH_3), 18.5 (CH_2) ppm;

6q': ^{1}H NMR (400 MHz, $CDCl_3$) δ 8.05 (d, *J* = 8.3 Hz, 1H), 7.95 (d, *J* = 8.3 Hz, 1H), 7.67 (ddd, *J* = 8.3, 7.0, 1.3 Hz, 1H), 7.50 (ddd, *J* = 8.1, 6.9, 1.1 Hz, 1H), 7.15 (s, 1H), 5.88 (ddt, *J* = 17.0, 10.3, 6.7 Hz, 1H), 5.13-4.93 (m, 2H), 2.94 (dd, *J* = 7.8, 7.1 Hz, 2H), 2.68 (s, 3H), 2.19 (q, *J* = 7.3 Hz, 2H), 1.97-1.79 (m, 2H) ppm; **^{13}C NMR** (101 MHz, $CDCl_3$) δ 162.3 (C), 147.7 (C), 144.0 (C), 138.4 (CH), 129.4 (CH), 129.0 (CH), 126.8 (C), 125.4 (CH), 123.5 (CH), 122.1 (CH), 114.8 (CH_2), 38.6 (CH_2), 33.6 (CH_2), 29.2 (CH_2), 18.7 (CH_3) ppm;

HRMS (EI) m/z calc'd for $C_{15}H_{17}N$ [M^+] 211.1361, found 211.1354.

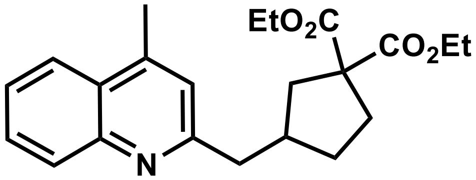

diethyl 3-((4-methylquinolin-2-yl)methyl)cyclopentane-1,1-dicarboxylate (6r)
Synthesized according to GP1.
IR (neat, cm^{-1}): 2978(m), 2939(m), 1725(s), 1251(s), 1176(m), 1158(m), 761(m); **^{1}H NMR** (400 MHz, $CDCl_3$) δ 8.05 (d, *J* = 8.6 Hz, 1H), 7.96 (dd, *J* = 8.3, 0.8 Hz, 1H), 7.69 (t, *J* = 7.3 Hz, 1H), 7.52 (t, *J* = 7.2 Hz, 1H), 7.14 (s, 1H), 4.17 (dq, *J* = 14.0, 7.1 Hz, 4H), 3.00 (d, *J* = 7.2 Hz, 2H), 2.69 (s, 3H), 2.62-2.52 (m, 1H), 2.46 (dd, *J* = 12.9, 6.6 Hz, 1H), 2.35 (ddd, *J* = 13.4, 8.4, 3.8 Hz, 1H), 2.17 (ddd, *J* = 13.6, 9.4, 7.5 Hz, 1H), 1.95 (dd, *J* = 13.4, 9.9 Hz, 1H), 1.90-1.82 (m, 1H), 1.49 (dq, *J* = 12.5, 9.2 Hz, 1H), 1.23 (dt, *J* = 11.3, 7.1 Hz, 6H) ppm; **^{13}C NMR** (101 MHz, $CDCl_3$) δ 172.6 (C), 172.5 (C), 161.0 (C), 147.4 (C), 144.3 (C), 129.2 (2 X CH), 126.8 (C), 125.6 (CH), 123.6 (CH), 122.3 (CH), 61.3 (CH_2), 61.3 (CH_2), 60.0 (C), 44.0 (CH_2), 40.4 (CH_2), 40.2 (CH), 33.7 (CH_2), 32.0 (CH_2), 18.7 (CH_3), 14.0 (CH_3), 14.0 (CH_3) ppm; **HRMS (EI)** m/z calc'd for $C_{22}H_{27}NO_4$ [M^+] 369.1940, found 369.1942.
***6r'** too little to fully characterized but ratio could be estimated by allyl peaks and a C-H aryl peaks by proton NMR.

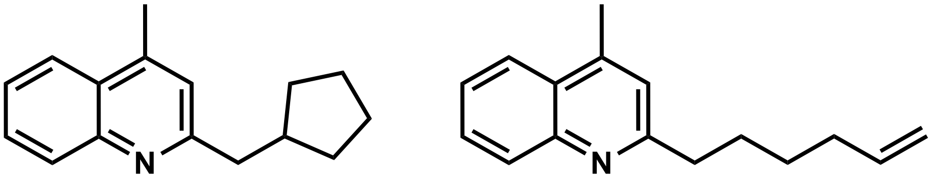

2-(cyclopentylmethyl)-4-methylquinoline/2-(hex-5-en-1-yl)-4-methylquinoline (90:10) (6t:6t')
Synthesized according to GP1 and characterized according to NMR comparison (*30*).
6t: ^{1}H NMR (400 MHz, $CDCl_3$) δ 8.08 (d, *J* = 8.2 Hz, 1H), 7.96 (dd, *J* = 8.3, 0.8 Hz, 1H), 7.68 (ddd, *J* = 8.4, 7.0, 1.4 Hz, 1H), 7.51 (ddd, *J* = 8.3, 7.0, 1.2 Hz, 1H), 7.15 (d, *J* = 0.7 Hz, 1H), 2.95 (d, *J* = 7.5 Hz, 2H), 2.69 (d, *J* = 0.9 Hz, 3H), 2.37 (quin, *J* = 7.8 Hz, 1H), 1.89-1.61 (m, 4H), 1.61-1.47 (m, 2H), 1.36-1.25 (m, 2H) ppm; **^{13}C NMR** (101 MHz, $CDCl_3$) δ 162.2 (C), 147.4 (C), 144.2 (C), 129.2 (CH), 129.1 (CH), 126.8 (C), 125.4 (CH), 123.6 (CH), 122.4 (CH), 44.9 (CH_2), 40.7 (CH), 32.5 (2 X CH_2), 25.0 (2 X CH_2), 18.7 (CH_3) ppm.
6t': ^{1}H NMR (400 MHz, $CDCl_3$) δ 8.08 (d, *J* = 8.2 Hz, 1H), 7.96 (dd, *J* = 8.3, 0.8 Hz, 1H), 7.68 (ddd, *J* = 8.4, 7.0, 1.4 Hz, 1H), 7.51 (ddd, *J* = 8.3, 7.0, 1.2 Hz, 1H), 7.10 (s, 1H), 5.82 (ddt, *J* = 17.0, 10.3, 6.7 Hz, 1H), 5.10-4.87 (m, 2H), 2.95 (m, 2H), 2.70 (d, *J* = 0.9 Hz, 3H), 2.18-2.07 (m, 2H), 1.89-1.25 (m, 4H) ppm; **^{13}C NMR** (101 MHz, $CDCl_3$) δ 162.4 (C), 147.4 (C), 144.2 (C), 138.8 (CH), 129.2 (CH), 129.1 (CH), 126.8 (C), 125.4 (CH), 123.6 (CH), 122.0 (CH), 114.4 (CH_2), 39.0 (CH_2), 33.6 (CH_2), 29.5 (CH_2), 28.8 (CH_2), 18.7 (CH_3) ppm.

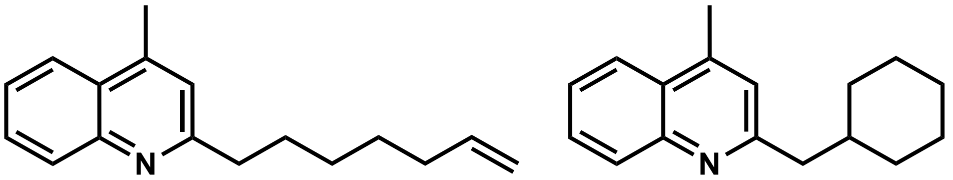

2-(hept-6-en-1-yl)-4-methylquinoline/2-(cyclohexylmethyl)-4-methylquinoline (80:20) (6u':6u)

Synthesized according to GP1.

IR (neat, cm^{-1}): 3065(m), 2924(s), 2853(m), 1641(m), 1603(s), 1448(m), 994(m), 910(m), 758(vs);

6u': ^{1}H NMR (400 MHz, $CDCl_3$) δ 8.09 (d, *J* = 8.3 Hz, 1H), 7.96 (d, *J* = 8.3 Hz, 1H), 7.69 (ddd, *J* = 8.3, 7.4, 1.3 Hz, 1H), 7.57-7.48 (m, 1H), 7.16 (s, 1H), 5.82 (ddt, *J* = 17.0, 10.3, 6.7 Hz, 1H), 5.08-4.86 (m, 2H), 2.99-2.88 (m, 2H), 2.70 (s, 3H), 2.12-2.02 (m, 2H), 1.90-1.77 (m, 2H), 1.52-1.38 (m, 4H) ppm; **^{13}C NMR** (101 MHz, $CDCl_3$) δ 162.5 (C), 147.3 (C), 144.6 (C), 139.0 (CH), 129.2 (CH), 129.0 (CH), 126.8 (C), 125.5 (CH), 123.6 (CH), 122.0 (CH), 114.2 (CH_2), 38.9 (CH_2), 33.6 (CH_2), 29.9 (CH_2), 29.0 (CH_2), 28.8 (CH_2), 18.7 (CH_3) ppm;

6u: ^{1}H NMR (400 MHz, $CDCl_3$) δ 8.09 (d, *J* = 8.3 Hz, 1H), 7.96 (d, *J* = 8.3 Hz, 1H), 7.69 (ddd, *J* = 8.3, 7.4, 1.3 Hz, 1H), 7.57-7.48 (m, 1H), 7.13 (s, 1H), 2.84 (d, *J* = 7.3, 2H), 2.70 (s, 3H), 1.76-1.62 (m, 1H), 1.62-1.00 (m, 10H); **^{13}C NMR** (101 MHz, $CDCl_3$) δ 161.5 (C), 147.3 (C), 144.6 (C), 129.2 (CH), 129.0 (CH), 126.8 (C), 125.5 (CH), 123.6 (CH), 122.9 (CH), 46.6 (CH_2), 38.9 (CH), 33.3 (2 X CH_2), 26.4 (CH_2), 26.2 (2 X CH_2), 18.8 (CH_3) ppm;

HRMS (EI) m/z calc'd for $C_{17}H_{21}N$ [M^+] 239.1674, found 239.1676.

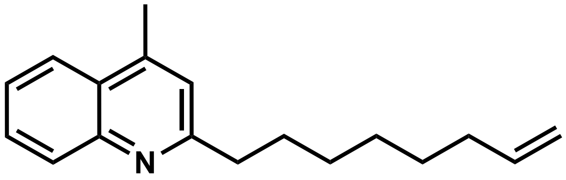

4-methyl-2-(oct-7-en-1-yl)quinoline (6v')

Synthesized according to GP1.

IR (neat, cm^{-1}): 3065(m), 2924(s), 2853(m), 1640(m), 1603(s), 1448(m), 994(m), 908(m), 756(vs); **^{1}H NMR** (400 MHz, $CDCl_3$) δ 8.08 (d, *J* = 8.4 Hz, 1H), 7.96 (d, *J* = 8.3 Hz, 1H), 7.73-7.61 (m, 1H), 7.58-7.45 (m, 1H), 7.15 (s, 1H), 5.81 (ddt, *J* = 17.0, 10.2, 6.7 Hz, 1H), 5.11-4.87 (m, 2H), 3.02-2.86 (m, 2H), 2.69 (s, 3H), 2.05 (q, *J* = 6.7 Hz, 2H), 1.81 (quin, *J* = 7.5 Hz, 2H), 1.40 (m, 6H) ppm; **^{13}C NMR** (101 MHz, $CDCl_3$) δ 162.6 (C), 147.4 (C), 144.4 (C), 139.1 (CH), 129.1 (2 X CH), 126.7 (C), 125.5 (CH), 123.6 (CH), 122.0 (CH), 114.2 (CH_2), 39.1 (CH_2), 33.7 (CH_2), 30.0 (CH_2), 29.4 (CH_2), 29.0 (CH_2), 28.8 (CH_2), 18.7 (CH_3) ppm; **HRMS (EI)** m/z calc'd for $C_{18}H_{23}N$ [M^+] 253.1830, found 253.1797.

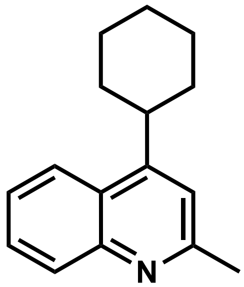

4-cyclohexyl-2-methylquinoline (8)
Synthesized according to GP1 and characterized according to NMR comparison (*17*).
^{1}H NMR (400 MHz, $CDCl_3$) δ 8.05 (t, *J* = 7.7 Hz, 2H), 7.73-7.58 (m, 1H), 7.56-7.42 (m, 1H), 7.18 (s, 1H), 3.33-3.27 (m, 1H), 2.73 (s, 3H), 2.08-1.80 (m, 5H), 1.62-1.44 (m, 4H), 1.37-1.32 (m, 1H) ppm; **^{13}C NMR** (101 MHz, $CDCl_3$) δ 158.7 (C), 153.5 (C), 147.9 (C), 129.3 (CH), 128.8 (CH), 125.3 (CH), 125.1 (C), 122.8 (CH), 118.3 (CH), 38.8 (CH), 33.5 (2 X CH_2), 26.9 (2 X CH_2), 26.3 (CH_2), 25.4 (CH_3) ppm.

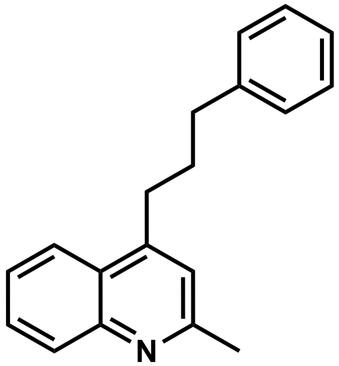

2-methyl-4-(3-phenylpropyl)quinoline (9)
Synthesized according to GP1.
IR (neat, cm^{-1}): 2936(m), 2860(m), 1602(vs), 1454(m), 763(s), 700(s); **^{1}H NMR** (400 MHz, $CDCl_3$) δ 8.05 (d, *J* = 8.3 Hz, 1H), 7.90 (d, *J* = 8.3 Hz, 1H), 7.66 (t, *J* = 7.6 Hz, 1H), 7.50-7.45 (m, 1H), 7.34-7.31 (m, 2H), 7.24-7.22 (m, 3H), 7.13 (s, 1H), 3.13-2.99 (m, 2H), 2.77 (t, *J* = 7.6 Hz, 2H), 2.72 (s, 3H), 2.10 (quin, *J* = 7.7 Hz, 2H) ppm; **^{13}C NMR** (101 MHz, $CDCl_3$) δ 158.5 (C), 148.0 (C), 148.0 (C), 141.5 (C), 129.3 (CH), 128.9 (CH), 128.4 (2 X CH), 128.4 (2 X CH), 125.9 (CH), 125.7 (C), 125.3 (CH), 123.2 (CH), 121.5 (CH), 35.7 (CH_2), 31.4 (2 X CH_2), 25.2 (CH_3) ppm; **HRMS (EI)** m/z calc'd for $C_{16}H_{18}ClN$ [M^+] 261.1517, found 261.1551.

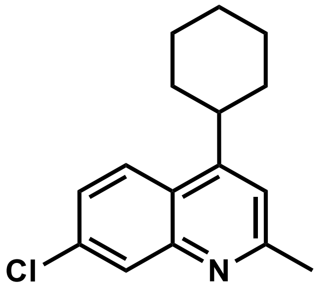

7-chloro-4-cyclohexyl-2-methylquinoline (10)
Synthesized according to GP1.
IR (neat, cm^{-1}): 2924(s), 2851(m), 1599(vs), 1447(m), 820 (vs), 769(s); **^{1}H NMR** (400 MHz, $CDCl_3$) δ 8.02 (d, *J* = 2.3 Hz, 1H), 7.96 (d, *J* = 9.0 Hz, 1H), 7.44 (dd, *J* = 9.0, 2.2 Hz, 1H), 7.16 (s, 1H), 3.32-3.09 (m, 1H), 2.71 (s, 3H), 2.01-1.84 (m, 5H), 1.60-1.48 (m, 4H), 1.41-1.31 (m, 1H) ppm; **^{13}C NMR** (101 MHz, $CDCl_3$) δ 160.1 (C), 153.3 (C), 148.7 (C), 134.6 (C), 128.5 (CH), 126.1 (CH), 124.3 (CH), 123.6 (C), 118.5 (CH), 38.9 (CH), 33.5 (2 X CH_2), 26.9 (2 X CH_2), 26.2 (CH_2), 25.5 (CH_3) ppm; **HRMS (EI)** m/z calc'd for $C_{16}H_{18}ClN$ [M^+] 259.1128, found 259.1101.

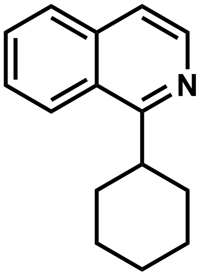

1-cyclohexylisoquinoline (11)
Synthesized according to GP1 and characterized according to NMR comparison (*17*).
^{1}H NMR (400 MHz, $CDCl_3$) δ 8.49 (d, *J* = 5.7 Hz, 1H), 8.23 (d, *J* = 8.4 Hz, 1H), 7.81 (d, *J* = 8.0 Hz, 1H), 7.65 (ddd, *J* = 8.0, 6.9, 1.2 Hz, 1H), 7.59 (ddd, *J* = 8.3, 7.0, 1.5 Hz, 1H), 7.48 (d, *J* = 5.7 Hz, 1H), 3.57 (tt, *J* = 11.6, 3.2 Hz, 1H), 2.05-1.91 (m, 4H), 1.88-1.76 (m, 3H), 1.55 (qt, *J* = 12.7, 3.1 Hz, 2H), 1.42 (tt, *J* = 12.6, 3.3 Hz, 1H) ppm; **^{13}C NMR** (101 MHz, $CDCl_3$) δ 165.7 (C), 141.9 (CH), 136.4 (C), 129.5 (CH), 127.5 (CH), 126.8 (CH), 126.3 (C), 124.7 (CH), 118.8 (CH), 41.5 (CH), 32.6 (2 X CH_2), 26.9 (2 X CH_2), 26.2 (CH_2) ppm.

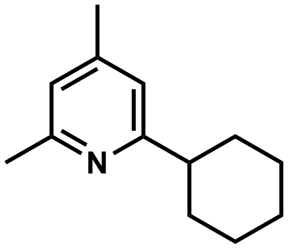

2-cyclohexyl-4,6-dimethylpyridine (12)
Synthesized according to GP1.
IR (neat, cm^{-1}): 2923(vs), 2851(s), 1607(s), 1571(m), 1449(m), 843(s); **^{1}H NMR** (400 MHz, $CDCl_3$) δ 6.79 (d, *J* = 3.4 Hz, 2H), 2.65 (tt, *J* = 11.6, 3.3 Hz, 1H), 2.49 (s, 3H), 2.28 (s, 3H), 2.00-1.92 (m, 2H), 1.88-1.81 (m, 2H), 1.78-1.72 (m, 1H), 1.48-1.38 (m, 4H), 1.33-1.28 (m, 1H) ppm; **^{13}C NMR** (101 MHz, $CDCl_3$) δ 165.9 (C), 157.1 (C), 147.4 (C), 121.6 (CH), 118.3 (CH), 46.6 (CH), 33.2 (2 X CH_2), 26.6 (2 X CH_2), 26.1 (CH_2), 24.4 (CH_3), 21.0 (CH_3) ppm; **HRMS (EI)** m/z calc'd for $C_{13}H_{19}N$ [M^+] 189.1517, found 189.1523.

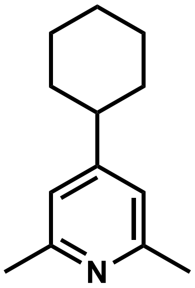

4-cyclohexyl-2,6-dimethylpyridine (13)
Synthesized according to GP1.
IR (neat, cm^{-1}): 2924(vs), 2852(s), 1605(vs), 1565(s), 1448(m), 850(s); **^{1}H NMR** (400 MHz, $CDCl_3$) δ 6.80 (s, 2H), 2.50 (s, 6H), 2.46-2.36 (m, 1H), 1.85-1.74 (m, 5H), 1.42-1.24 (m, 5H) ppm; **^{13}C NMR** (101 MHz, $CDCl_3$) δ 157.4 (2 X C), 157.2 (C), 118.9 (2 X CH), 43.8 (CH), 33.5 (2 X CH_2), 26.6 (2 X CH_2), 26.0 (CH_2), 24.4 (2 X CH_3) ppm; **HRMS (EI)** m/z calc'd for $C_{13}H_{19}N$ [M^+] 189.1517, found 189.1534.

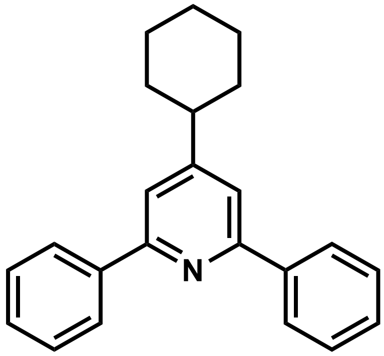

4-cyclohexyl-2,6-diphenylpyridine (14)
Synthesized according to GP1 and characterized according to NMR comparison (*33*).
^{1}H NMR (400 MHz, $CDCl_3$) δ 8.22-8.12 (m, 4H), 7.55 (s, 2H), 7.53-7.47 (m, 4H), 7.46-7.40 (m, 2H), 2.66 (tt, *J* = 11.7, 3.3 Hz, 1H), 2.04-1.88 (m, 4H), 1.85-1.79 (m, 1H), 1.62-1.26 (m, 5H and grease) ppm; **^{13}C NMR** (101 MHz, $CDCl_3$) δ 158.0 (C), 156.9 (C), 139.9 (C), 128.7 (CH), 128.6 (2 X CH), 127.0 (2 X CH), 117.6 (CH), 44.4 (CH), 33.7 (2 X CH_2), 26.6 (2 X CH_2), 26.0 (CH_2) ppm.

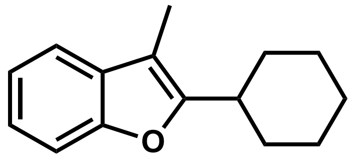

2-cyclohexyl-3-methylbenzofuran (15)
Synthesized according to GP1.
IR (neat, cm^{-1}): 2925(s), 2853(m), 1453(s), 743(vs); **^{1}H NMR** (400 MHz, $CDCl_3$) δ 7.46-7.41 (m, 1H), 7.41-7.36 (m, 1H), 7.24-7.16 (m, 2H), 2.82 (tt, *J* = 11.8, 3.5 Hz, 1H), 2.19 (s, 3H), 1.91-1.82 (m, 4H), 1.80-1.69 (m, 3H), 1.48-1.33 (m, 3H) ppm; **^{13}C NMR** (101 MHz, $CDCl_3$) δ 158.1 (C), 153.6 (C), 130.6 (C), 122.8 (CH), 121.8 (CH), 118.6 (CH), 110.5 (CH), 107.7 (C), 36.3 (CH), 31.3 (2 X CH_2), 26.5 (2 X CH_2), 25.9 (CH_2), 7.8 (CH_3) ppm; **HRMS (EI)** m/z calc'd for $C_{15}H_{18}O$ [M^+] 214.1358, found 214.1346.

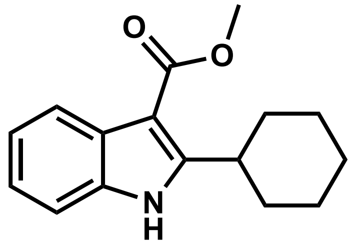

methyl 2-cyclohexyl-1H-indole-3-carboxylate (16)
Synthesized according to GP1.
IR (neat, cm^{-1}): 3311 (br), 2925(s), 2851(m), 1664 (vs), 1449(vs), 1196(vs), 1081(s), 793 (m), 744(s); **^{1}H NMR** (400 MHz, $CDCl_3$) δ 8.58 (br. s., 1H), 8.16-8.07 (m, 1H), 7.39-7.32 (m, 1H), 7.26-7.18 (m, 2H), 3.95 (s, 3H), 3.81 (tt, *J* = 11.8, 3.1 Hz, 1H), 2.14-2.04 (m, 2H), 1.93-1.77 (m, 3H), 1.58-1.39 (m, 4H), 1.33-1.22 (m, 1H) ppm; **^{13}C NMR** (101 MHz, $CDCl_3$) δ 166.4 (C), 153.0 (C), 134.4 (C), 127.0 (C), 122.3 (CH), 121.7 (CH), 121.5 (CH), 110.7 (CH), 102.8 (C), 50.7 (CH_3), 36.3 (CH), 32.4 (2 X CH_2), 26.4 (2 X CH_2), 26.1 (CH_2) ppm; **HRMS (EI)** m/z calc'd for $C_{16}H_{19}NO_2$ [M^+] 257.1416, found 257.1650.

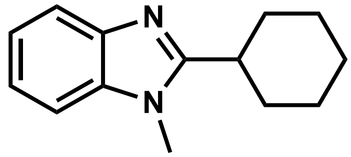

2-cyclohexyl-1-methyl-1H-benzo[d]imidazole (17)
Synthesized according to GP1 and characterized according to NMR comparison (*34*).
^{1}H NMR (400 MHz, $CDCl_3$) δ = 7.81-7.69 (m, 1 H), 7.33-7.28 (m, 1 H), 7.25-7.19 (m, 2 H), 3.75 (s, 3 H), 2.86 (tt, *J* = 11.7, 3.3 Hz, 1 H), 2.05-1.89 (m, 4 H), 1.88-1.75 (m, 3 H), 1.50-1.34 (m, 3 H) ppm; **^{13}C NMR** (101 MHz, $CDCl_3$) δ 159.0 (C), 142.5 (C), 135.6 (C), 121.9 (CH), 121.7 (CH), 119.3 (CH), 108.8 (CH), 36.3 (CH), 31.4 (2 X CH_2), 29.5 (CH_3), 26.3 (2 X CH_2), 25.8 (CH_2) ppm.

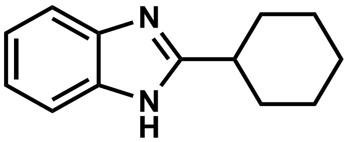

2-cyclohexyl-1H-benzo[d]imidazole (18)
Synthesized according to GP1 and characterized according to NMR comparison (*35*).
^{1}H NMR (400 MHz, DMSO-d_6) δ 12.09 (s, 1H), 7.50-7.40 (m, 2H), 7.10 (s, 2H), 2.83 (t, *J* = 11.3 Hz, 1H), 2.01 (d, *J* = 12.3 Hz, 2H), 1.81-1.56 (m, 5H), 1.46-1.21 (m, 3H) ppm; **^{13}C NMR** (101 MHz, DMSO-d_6) δ 158.9 (C), 143.1 (C), 134.2 (C), 121.3 (CH), 120.7 (CH), 118.2 (CH), 110.7 (CH), 37.7 (CH), 31.2 (2 X CH_2), 25.6 (CH_2), 25.5 (2 X CH_2) ppm.

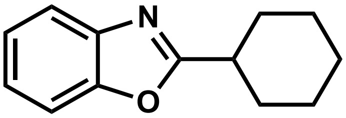

2-cyclohexylbenzo[d]oxazole (19)
Synthesized according to GP1 and characterized according to NMR comparison (*20g*).
^{1}H NMR (400 MHz, $CDCl_3$) δ 7.74-7.66 (m, 1H), 7.52-7.45 (m, 1H), 7.34-7.28 (m, 2H), 2.97 (tt, *J* = 11.4, 3.7 Hz, 1H), 2.22-2.15 (m, 2H), 1.88 (dt, *J* = 12.9, 3.3 Hz, 2H and grease), 1.80-1.66 (m, 2H and grease), 1.51-1.36 (m, 4H) ppm; **^{13}C NMR** (101 MHz, $CDCl_3$) δ 170.4 (C), 150.6 (C), 141.3 (C), 124.3 (CH), 124.0 (CH), 119.6 (CH), 110.3 (CH), 37.9 (CH), 30.5 (2 X CH_2), 25.8 (CH_2), 25.6 (2 X CH_2) ppm.

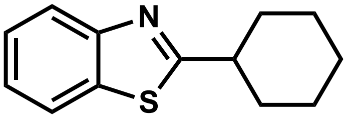

2-cyclohexylbenzo[d]thiazole (20)
Synthesized according to GP1 and characterized according to NMR comparison (*36*).
^{1}H NMR (400 MHz, $CDCl_3$) δ 8.01-7.95 (m, 1H), 7.88-7.83 (m, 1H), 7.48-7.42 (m, 1H), 7.37-7.31 (m, 1H), 3.12 (tt, *J* = 11.7, 3.6 Hz, 1H), 2.25-2.19 (m, 2H), 1.90 (dt, *J* = 13.1, 3.3 Hz, 2H), 1.81-1.75 (m, 1H), 1.71-1.60 (m, 2H), 1.46 (qt, *J* = 12.6, 3.2 Hz, 2H), 1.35 (tt, *J* = 12.3, 3.2 Hz, 1 H) ppm; **^{13}C NMR** (101 MHz, $CDCl_3$) δ 177.6 (C), 153.1 (C), 134.5 (C), 125.8 (CH), 124.5 (CH), 122.5 (CH), 121.5 (CH), 43.4 (CH), 33.4 (2 X CH_2), 26.1 (2 X CH_2), 25.8 (CH_2) ppm.

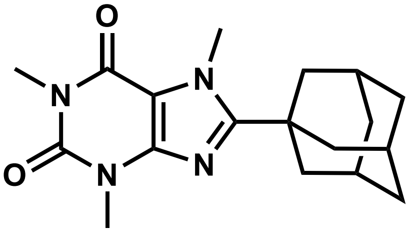

8-((3r,5r,7r)-adamantan-1-yl)-1,3,7-trimethyl-3,7-dihydro-1H-purine-2,6-dione (21)
Synthesized according to GP1 and characterized according to NMR comparison (*20g*).
^{1}H NMR (400 MHz, $CDCl_3$) δ 4.17 (s, 3H), 3.57 (s, 3H), 3.40 (s, 3H), 2.20-2.09 (m, 9H), 1.88-1.75 (m, 6 H) ppm; **^{13}C NMR** (101 MHz, $CDCl_3$) δ 159.5 (C), 155.7 (C), 151.8 (C), 147.1 (C), 108.1 (C), 39.8 (3 X CH_2), 36.8 (C), 36.4 (3 X CH_2), 34.4 (CH_3), 29.6 (CH_3), 28.2 (3 X CH), 27.9 (CH_3) ppm.

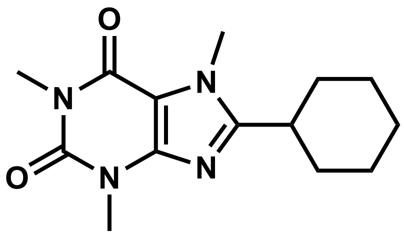

8-cyclohexyl-1,3,7-trimethyl-3,7-dihydro-1H-purine-2,6-dione (22)
Synthesized according to GP1 and characterized according to NMR comparison (*20g*).
^{1}H NMR (400 MHz, $CDCl_3$) δ 3.93 (s, 3H), 3.57 (s, 3H), 3.40 (s, 3H), 2.71 (tt, J = 11.7, 3.4 Hz, 1H), 1.93-1.83 (m, 4H), 1.79-1.63 (m, 3H and grease), 1.45-1.31 (m, 3H) ppm; **^{13}C NMR** (101 MHz, $CDCl_3$) δ 158.0 (C), 155.5 (C), 151.8 (C), 148.1 (C), 107.0 (C), 35.8 (CH), 31.4 (CH_3), 30.9 (2 X CH_2), 29.7 (CH_3), 27.8 (CH_3), 26.0 (2 X CH_2), 25.5 (CH_2) ppm.

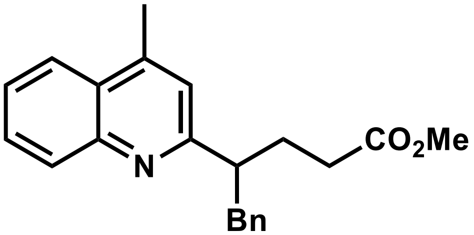

methyl 4-(4-methylquinolin-2-yl)-5-phenylpentanoate (23)
Synthesized according to GP2.
IR (neat, cm^{-1}): 2982(s), 2953(m), 1737(vs), 1602(m), 1451(m), 1159(s), 759(m), 702(m); **^{1}H NMR** (400 MHz, $CDCl_3$) δ 8.08 (d, J = 8.4 Hz, 1 H), 7.95 (dd, J = 8.3, 0.9 Hz, 1 H), 7.69 (ddd, J = 8.4, 6.9, 1.4 Hz, 1 H), 7.52 (ddd, J = 8.2, 7.0, 1.3 Hz, 1 H), 7.24-7.19 (m, 2 H), 7.17-7.11 (m, 3 H), 7.02 (s, 1 H), 3.56 (s, 3 H), 3.28-3.17 (m, 2 H), 3.05-2.95 (m, 1 H), 2.65 (d, J = 0.7 Hz, 3 H), 2.26-2.08 (m, 4 H) ppm; **^{13}C NMR** (101 MHz, $CDCl_3$) δ 173.9 (C), 163.4 (C), 147.8 (C), 144.2 (C), 140.1 (C), 129.7 (CH), 129.2 (2 X CH), 128.9 (CH), 128.2 (2 X CH), 127.1 (C), 125.9 (CH), 125.6 (CH), 123.6 (CH), 121.7 (CH), 51.4 (CH_3), 49.5 (CH), 41.8 (CH_2), 32.1 (CH_2), 29.4 (CH_2), 18.7 (CH_3) ppm; **HRMS (EI)** m/z calc'd for $C_{22}H_{23}NO_2$ [M^+] 333.1729, found 333.1769.

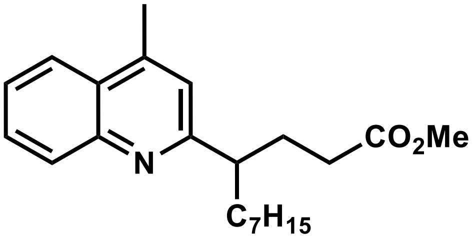

methyl 4-(4-methylquinolin-2-yl)undecanoate (24)
Synthesized according to GP2.
IR (neat, cm^{-1}): 2926(s), 2855(m), 1736(vs), 1603(m), 1450(m), 1163(m), 760(m); **^{1}H NMR** (400 MHz, $CDCl_3$) δ 8.06 (d, J = 8.4 Hz, 1H), 7.95 (d, J = 8.3 Hz, 1H), 7.67 (td, J = 7.6, 1.2 Hz, 1H), 7.55-7.47 (m, 1H), 7.11 (s, 1H), 3.59 (s, 3H), 2.97-2.83 (m, 1H), 2.69 (s, 3H), 2.33-2.21 (m, 1H), 2.20-2.07 (m, 3H), 1.86-1.67 (m, 2H), 1.30-1.16 (m, 10H), 0.84 (t, J = 6.9 Hz, 3H) ppm; **^{13}C NMR** (101 MHz, $CDCl_3$) δ 174.1 (C), 164.5 (C), 147.6 (C), 144.3 (C), 129.6 (CH), 128.9 (CH), 127.1 (C), 125.5 (CH), 123.5 (CH), 120.8 (CH), 51.4 (CH_3), 48.0 (CH), 35.6 (CH_2), 32.2 (CH_2), 31.7 (CH_2), 30.4 (CH_2), 29.6 (CH_2), 29.1 (CH_2), 27.5 (CH_2), 22.6 (CH_2), 18.8 (CH_3), 14.0 (CH_3) ppm; **HRMS (EI)** m/z calc'd for $C_{22}H_{31}NO_2$ [M^+] 341.2355, found 341.2392.

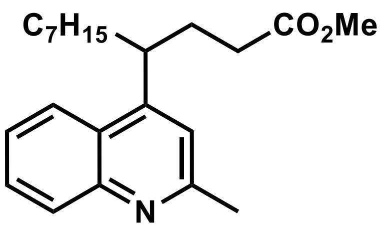

methyl 4-(2-methylquinolin-4-yl)undecanoate (25)
Synthesized according to GP2.
IR (neat, cm^{-1}): 2927(s), 2855(m), 1736(vs), 1598(m), 1169(m), 763(m); **^{1}H NMR** (400 MHz, $CDCl_3$) δ 8.05-8.01 (m, 2H), 7.66 (t, *J* = 7.4 Hz, 1H), 7.48 (t, *J* = 7.4 Hz, 1H), 7.15 (s, 1H), 3.57 (s, 3H), 3.51-3.50 (m, 1H), 2.73 (s, 3H), 2.23-2.10 (m, 3H), 2.08-1.97 (m, 1H), 1.81-1.68 (m, 2H), 1.26-1.14 (m, 10H), 0.83 (t, *J* = 6.9 Hz, 3H) ppm; **^{13}C NMR** (101 MHz, $CDCl_3$) δ 173.7 (C), 158.5 (C), 151.4 (C), 148.1 (C), 129.5 (CH), 129.0 (CH), 126.2 (C), 125.4 (CH), 122.7 (CH), 118.8 (CH), 51.4 (CH_3), 37.8 (CH), 36.1 (CH_2), 31.7 (CH_2), 31.7 (CH_2), 30.9 (CH_2), 29.6 (CH_2), 29.0 (CH_2), 27.3 (CH_2), 25.4 (CH_3), 22.5 (CH_2), 14.0 (CH_3) ppm; **HRMS (EI)** m/z calc'd for $C_{22}H_{31}NO_2$ [M^+] 341.2355, found 341.2364.

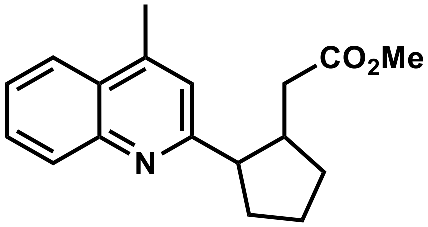

methyl 2-(2-(4-methylquinolin-2-yl)cyclopentyl)acetate (26)
Synthesized according to GP2.
IR (neat, cm^{-1}): 2949(s), 2870(m), 1735(vs), 1602(s), 1438(m), 1165(m), 760(s); **^{1}H NMR** (400 MHz, $CDCl_3$) δ 8.04 (d, *J* = 8.3 Hz, 1 H), 7.95 (dd, *J* = 8.3, 0.6 Hz, 1 H), 7.67 (ddd, *J* = 8.3, 6.9, 1.2 Hz, 1 H), 7.50 (ddd, *J* = 8.2, 7.0, 1.1 Hz, 1 H), 7.18 (s, 1 H), 3.50 (s, 3 H), 2.96 (td, *J* = 9.4, 9.2 Hz, 1 H), 2.71-2.63 (m, 4 H (CH_3 + CH), 2.49 (dd, *J* = 15.2, 4.8 Hz, 1 H), 2.31 (dd, *J* = 15.2, 9.5 Hz, 1 H), 2.33-2.15 (m, 2 H), 1.96-1.88 (m, 2 H), 1.86-1.80 (m, 1 H), 1.49-1.42 (m, 1 H) ppm; **^{13}C NMR** (101 MHz, $CDCl_3$) δ 173.5 (C), 163.9 (C), 147.6 (C), 144.3 (C), 129.5 (CH), 128.9 (CH), 127.1 (C), 125.5 (CH), 123.5 (CH), 120.7 (CH), 54.8 (CH), 51.3 (CH_3), 43.4 (CH), 38.7 (CH_2), 33.7 (CH_2), 32.6 (CH_2), 24.2 (CH_2), 18.8 (CH_3) ppm; **HRMS (EI)** m/z calc'd for $C_{18}H_{21}NO_2$ [M^+] 283.1572, found 283.1570.

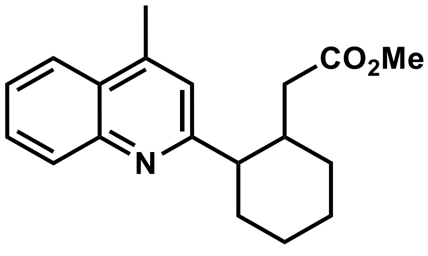

methyl 2-(2-(4-methylquinolin-2-yl)cyclohexyl)acetate (27)
Synthesized according to GP2.
IR (neat, cm^{-1}): 2925(s), 2853(m), 1735(vs), 1603(s), 1447(m), 1158(m), 759(s)**;** ^{1}**H NMR** (400 MHz, $CDCl_3$) δ 8.04 (d, *J* = 8.4 Hz, 1 H), 7.96 (dd, *J* = 8.3, 1.0 Hz, 1 H), 7.68 (ddd, *J* = 8.4, 6.9, 1.4 Hz, 1 H), 7.51 (ddd, *J* = 8.2, 6.9, 1.2 Hz, 1 H), 7.19 (s, 1 H), 3.50 (s, 3 H), 2.70 (d, *J* = 0.7 Hz, 3 H), 2.65 (dd, *J* = 11.4, 3.6 Hz, 1 H), 2.40-2.30 (m, 1 H), 2.14 (dd, *J* = 15.3, 3.9 Hz, 1 H), 2.05 (dd, *J* = 9.7, 6.1 Hz, 1 H), 2.02-1.93 (m, 2 H), 1.90-1.81 (m, 2 H), 1.66 (qd, *J* = 12.5, 3.4 Hz, 2 H), 1.46 (qt, *J* = 13.0, 3.3 Hz, 2 H), 1.22 (td, *J* = 12.1, 2.6 Hz, 1 H) ppm; ^{13}**C NMR** (101 MHz, $CDCl_3$) δ 173.5 (C), 164.7 (C), 147.6 (C), 144.6 (C), 129.6 (CH), 129.0 (CH), 127.1 (C), 125.6 (CH), 123.6 (CH), 120.5 (CH), 52.9 (CH), 51.2 (CH_3), 39.5 (CH_2), 38.6 (CH), 33.9 (CH_2), 32.6 (CH_2), 26.3 (CH_2), 26.0 (CH_2), 18.9 (CH_3) ppm; **HRMS (EI)** m/z calc'd for $C_{19}H_{23}NO_2$ [M^+] 297.1729, found 297.1701.

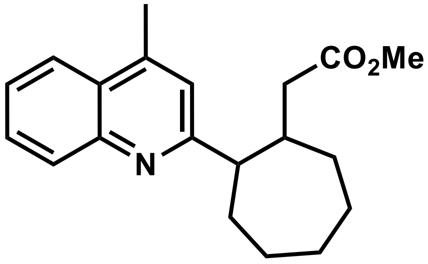

methyl 2-(2-(4-methylquinolin-2-yl)cycloheptyl)acetate (28)
Synthesized according to GP2.
IR (neat, cm^{-1}): 2922(s), 2855(m), 1734(vs), 1602(s), 1443(m), 1147(m), 759(s); ^{1}**H NMR** (400 MHz, $CDCl_3$) δ 8.04 (d, *J* = 8.4 Hz, 1 H), 7.94 (d, *J* = 8.1 Hz, 1 H), 7.67 (ddd, *J* = 8.1, 7.0, 1.1 Hz, 1 H), 7.51 (ddd, *J* = 7.9, 6.8, 0.8 Hz, 1 H), 7.17 (s, 1 H), 3.50 (s, 3 H), 2.82-2.74 (m, 1 H), 2.69 (s, 3 H), 2.61-2.51 (m, 1 H), 2.21-2.15 (m, 2 H), 1.93-1.83 (m, 4 H), 1.79-1.68 (m, 3 H), 1.64-1.56 (m, 3 H) ppm; ^{13}**C NMR** (101 MHz, $CDCl_3$) δ 173.7 (C), 166.5 (C), 147.3 (C), 144.7 (C), 129.6 (CH), 129.0 (CH), 127.0 (C), 125.5 (CH), 123.5 (CH), 120.6 (CH), 54.9 (CH), 51.2 (CH_3), 40.9 (CH), 39.9 (CH_2), 33.8 (CH_2), 32.5 (CH_2), 28.8 (CH_2), 27.3 (CH_2), 24.9 (CH_2), 18.8 (CH_3) ppm; **HRMS (EI)** m/z calc'd for $C_{20}H_{25}NO_2$ [M^+] 311.1885, found 311.1915.

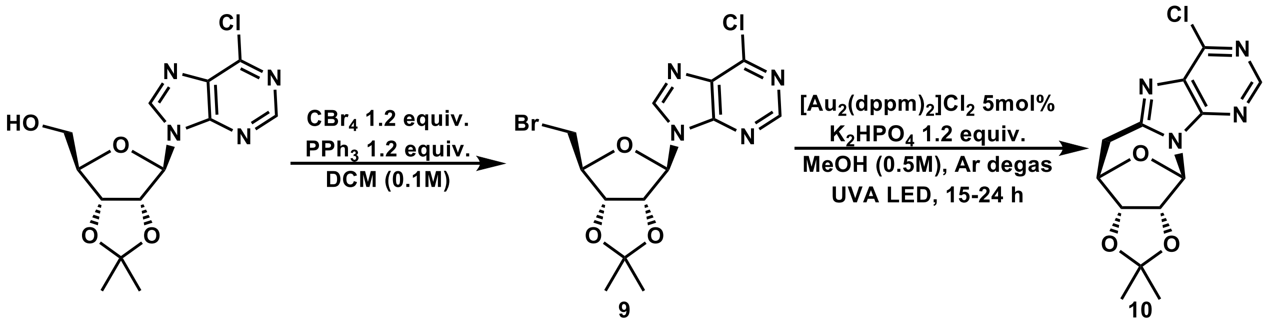

9-((3aR,4R,6S,6aS)-6-(bromomethyl)-2,2-dimethyltetrahydrofuro[3,4-d][1,3]dioxol-4-yl)-6-chloro-9H-purine (29)

To a flame dried 25 mL round bottomed flask under argon atmosphere was added triphenylphosphine (1.84 mmol, 1.2 eq.) and 15 mL of DCM. Upon cooling to 0°C, the reaction mixture was slowly added tetrabromomethane (1.84 mmol, 1.2 eq) and allowed to stir for 30 minutes, reaching room temperature. 6-Chloro-9-β-D-(2,3-isopropylidene)ribofuranosylpurine (1.53 mmol, 1.0 eq.) was then added and the mixture was heated to reflux for overnight. Upon completion as judged by TLC analysis, the mixture was added silica, concentrated under reduced pressure, and dry packed directly onto a flash chromatography column and eluted (0 to 50% EtOAc:Hex). Relevant fractions were combined affording the brominated product in 30 % yield.

IR (neat, cm^{-1}): 2970(m), 2938(w), 1592 (w), 1560(vs), 1204(s), 1085(s), 952(m), 864 (m); **^{1}H NMR** (400 MHz, $CDCl_3$) δ 8.79 (s, 1H), 8.32 (s, 1H), 6.21 (d, J = 2.5 Hz, 1H), 5.42 (dd, J = 6.4, 2.5 Hz, 1H), 5.11 (dd, J = 6.4, 3.0 Hz, 1H), 4.56 (ddd, J = 6.9, 4.8, 3.1 Hz, 1H), 3.64 (dd, J = 10.7, 6.9 Hz, 1H), 3.51 (dd, J = 10.7, 4.9 Hz, 1H), 1.65 (s, 3H), 1.41 (s, 3H) ppm; **^{13}C NMR** (101 MHz, $CDCl_3$) δ 152.2 (CH), 151.7 (C), 150.8 (C), 144.3 (CH), 132.4 (C), 115.1 (C), 91.2 (CH), 86.0 (CH), 84.1 (CH), 83.2 (CH), 31.7 (CH_2), 27.1 (CH_3), 25.3 (CH_3) ppm; **HRMS (EI)** m/z calc'd for $C_{13}H_{14}BrClN_4O_3$ [M^+] 387.9938, found [M^+-$CH_3^{\cdot}$] 372.9750.

(3aR,4R,12R,12aR)-9-chloro-2,2-dimethyl-3a,11,12,12a-tetrahydro-4H-4,12-epoxy[1,3]dioxolo[4',5':5,6]azepino[1,2-e]purine (30)

Synthesized according to GP1.

IR (neat, cm^{-1}): 2980(m), 2936(m), 1600(s), 1560(s), 1441(m), 1376(m), 1094(s), 845(s), 734(s); **^{1}H NMR** (400 MHz, $CDCl_3$) δ 8.70 (s, 1H), 6.40 (s, 1H), 4.91 (d, J = 6.5 Hz, 1H), 4.75-4.67 (dd, J = 18.5, 5.5 Hz, 2H), 3.63 (dd, J = 18.5, 6.6 Hz, 1H), 3.13 (d, J = 18.4 Hz, 1H), 1.57 (s, 3H), 1.31 (s, 3 H) ppm; **^{13}C NMR** (101 MHz, $CDCl_3$) δ 151.7 (CH), 150.0 (C), 149.7 (C), 149.4 (C), 131.1 (C), 114.1 (C), 86.4 (CH), 85.5 (CH), 83.0 (CH), 79.8 (CH), 29.4 (CH_2), 26.0 (CH_3), 24.9 (CH_3) ppm; **HRMS (EI)** m/z calc'd for $C_{13}H_{13}ClN_4O_3$ [M^+] 308.0676, found 308.0668; **[m.p.]** 208-212 °C.

References

32. B. Gabriele, R. Mancuso, G. Salerno, G. Ruffolo, P. Plastina, *J. Org. Chem.* **2007**, *72*, 6873-6877.
33. E. Wenkert, J. M. Hanna Jr., M. H. Leftin, E. L. Michelotti, K. T. Potts, D. Usifer, *J. Org. Chem.* **1985**, *50*, 1125-1126.
34. R.-J. Tang, L. Kang, L. Yang, *Adv. Synth. Catal.* **2015**, *357*, 2055-2060.
35. S. Park, J. Jung, E. J. Cho, *Eur. J. Org. Chem.* **2014**, 4148-4154.
36. Y. Yao, K. Hirano, T. Satoh, M. Miura, *Angew. Chem. Int.* Ed. **2012**, *51*, 775-779.

5. Spectra

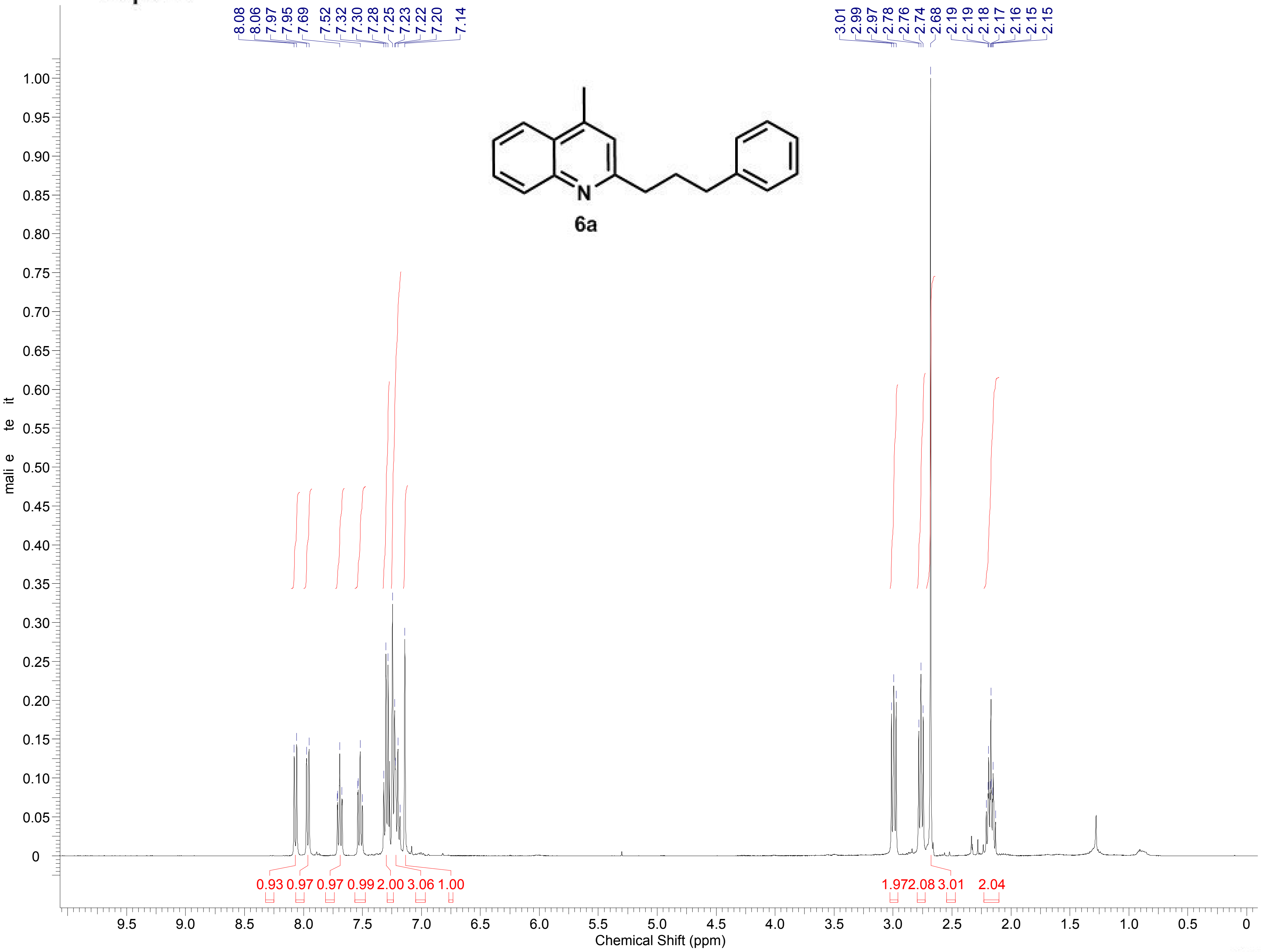
8.08 8.06 7.97 7.95 7.69 7.52 7.32 7.30 7.28 7.25 7.23 7.22 7.20 7.14
3.01 2.99 2.97 2.78 2.76 2.74 2.68 2.19 2.19 2.18 2.17 2.16 2.15 2.15
N
6a
0.93 0.97 0.97 0.99 2.00 3.06 1.00
1.97 2.08 3.01 2.04
Chemical Shift (ppm)

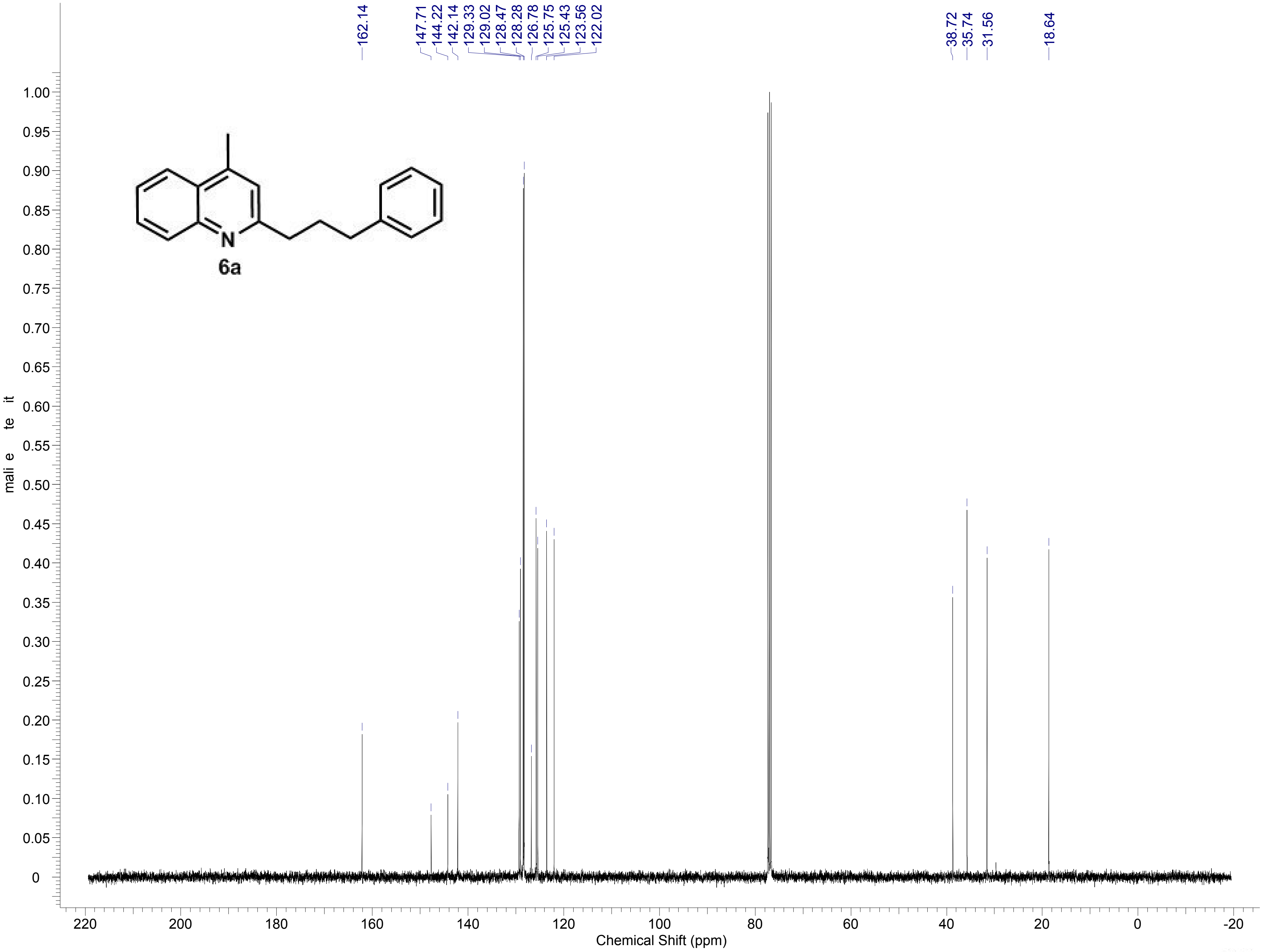

162.14
147.71
144.22
142.14
129.33
129.02
128.47
128.28
126.78
125.75
125.43
123.56
122.02
38.72
35.74
31.56
18.64
N
6a
Chemical Shift (ppm)

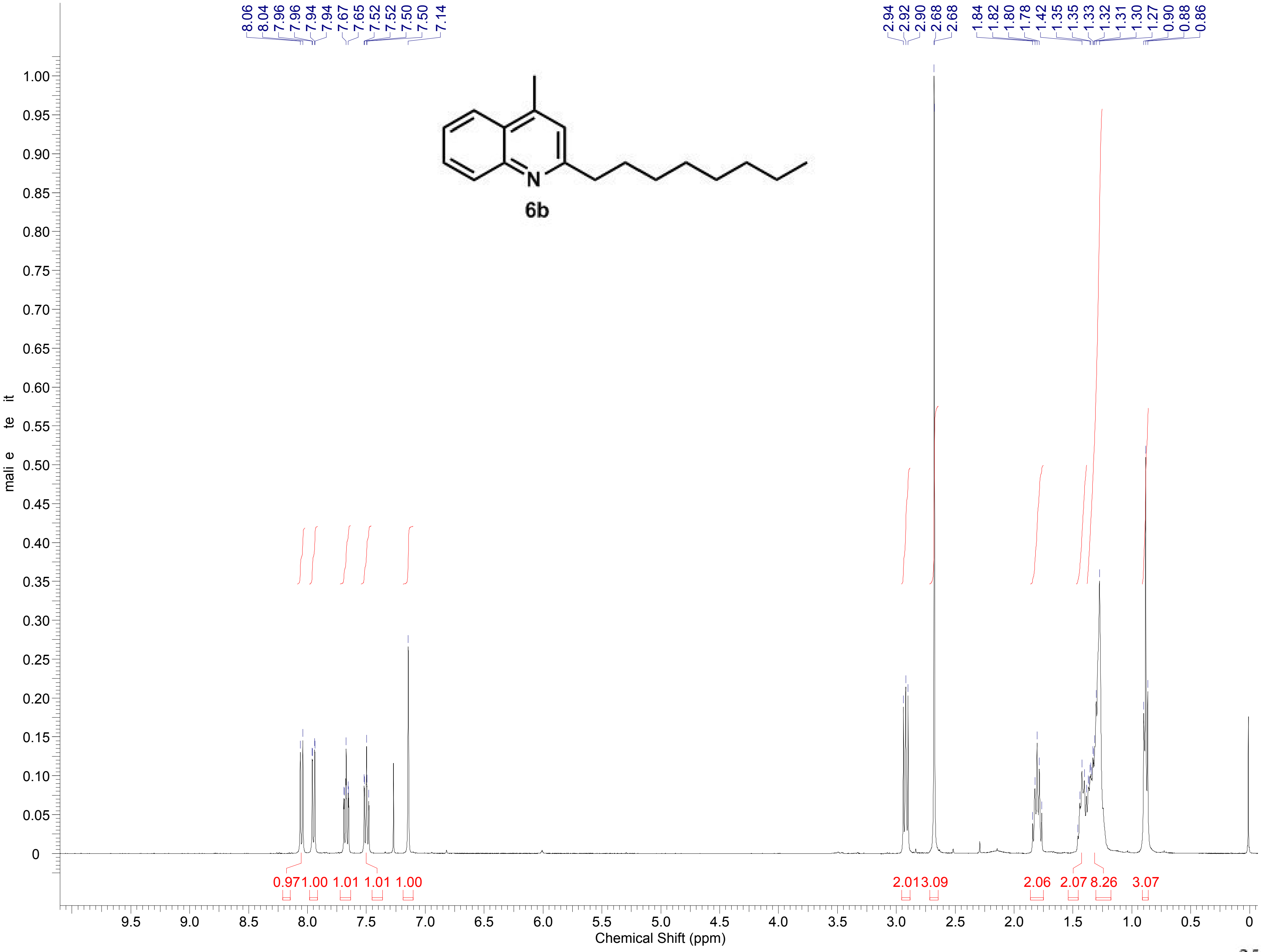
8.06
8.04
7.96
7.96
7.94
7.94
7.67
7.65
7.52
7.52
7.50
7.50
7.14
2.94
2.92
2.90
2.68
2.68
1.84
1.82
1.80
1.78
1.42
1.35
1.35
1.33
1.32
1.31
1.30
1.27
0.90
0.88
0.86
N
6b
0.97
1.00
1.01
1.01
1.00
2.01
3.09
2.06
2.07
8.26
3.07
Chemical Shift (ppm)

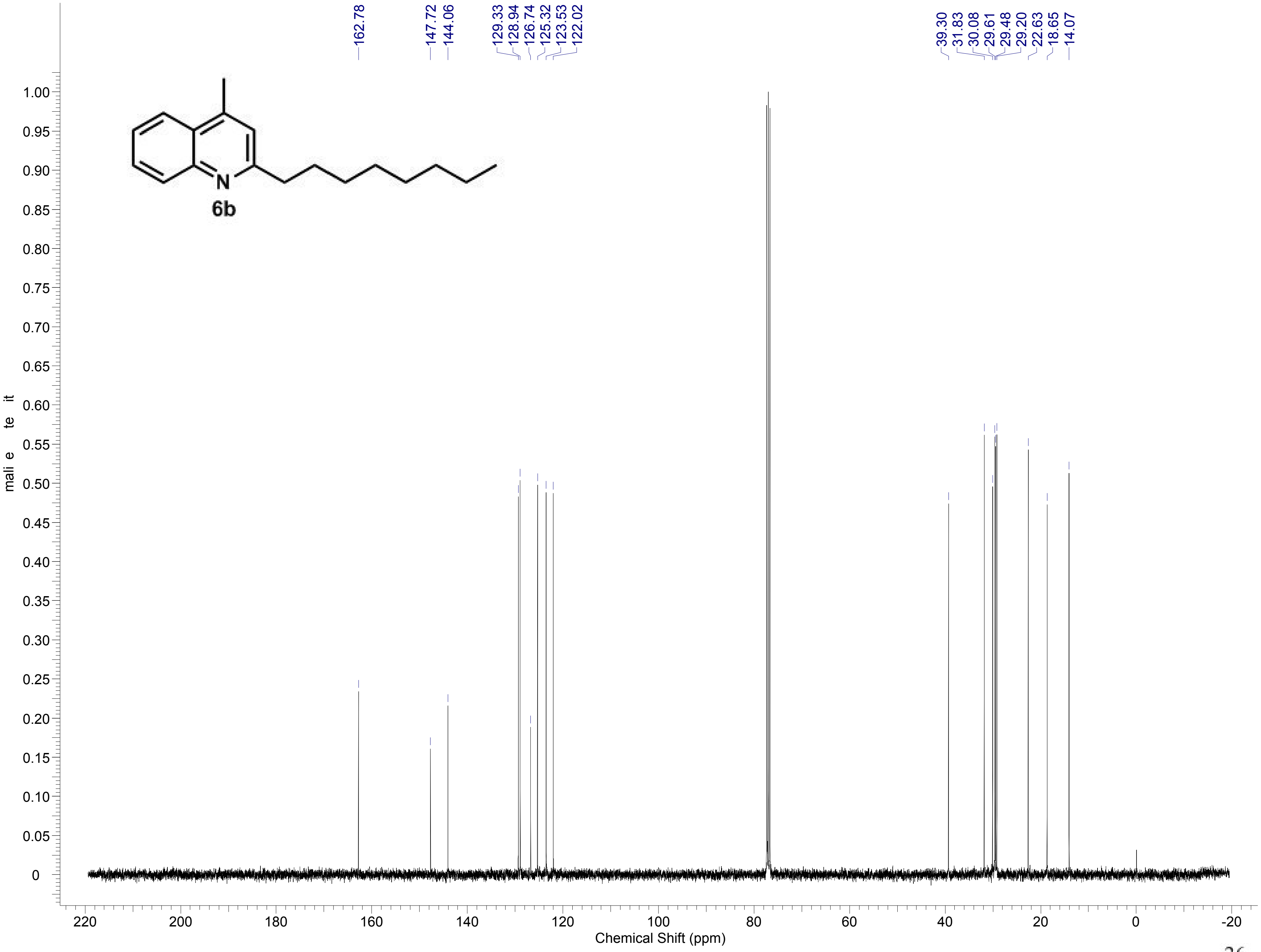
162.78
147.72
144.06
129.33
128.94
126.74
125.32
123.53
122.02
39.30
31.83
30.08
29.61
29.48
29.20
22.63
18.65
14.07
N
6b
1.00
0.95
0.90
0.85
0.80
0.75
0.70
0.65
0.60
0.55
0.50
0.45
0.40
0.35
0.30
0.25
0.20
0.15
0.10
0.05
0
220
200
180
160
140
120
100
80
60
40
20
0
-20
Chemical Shift (ppm)

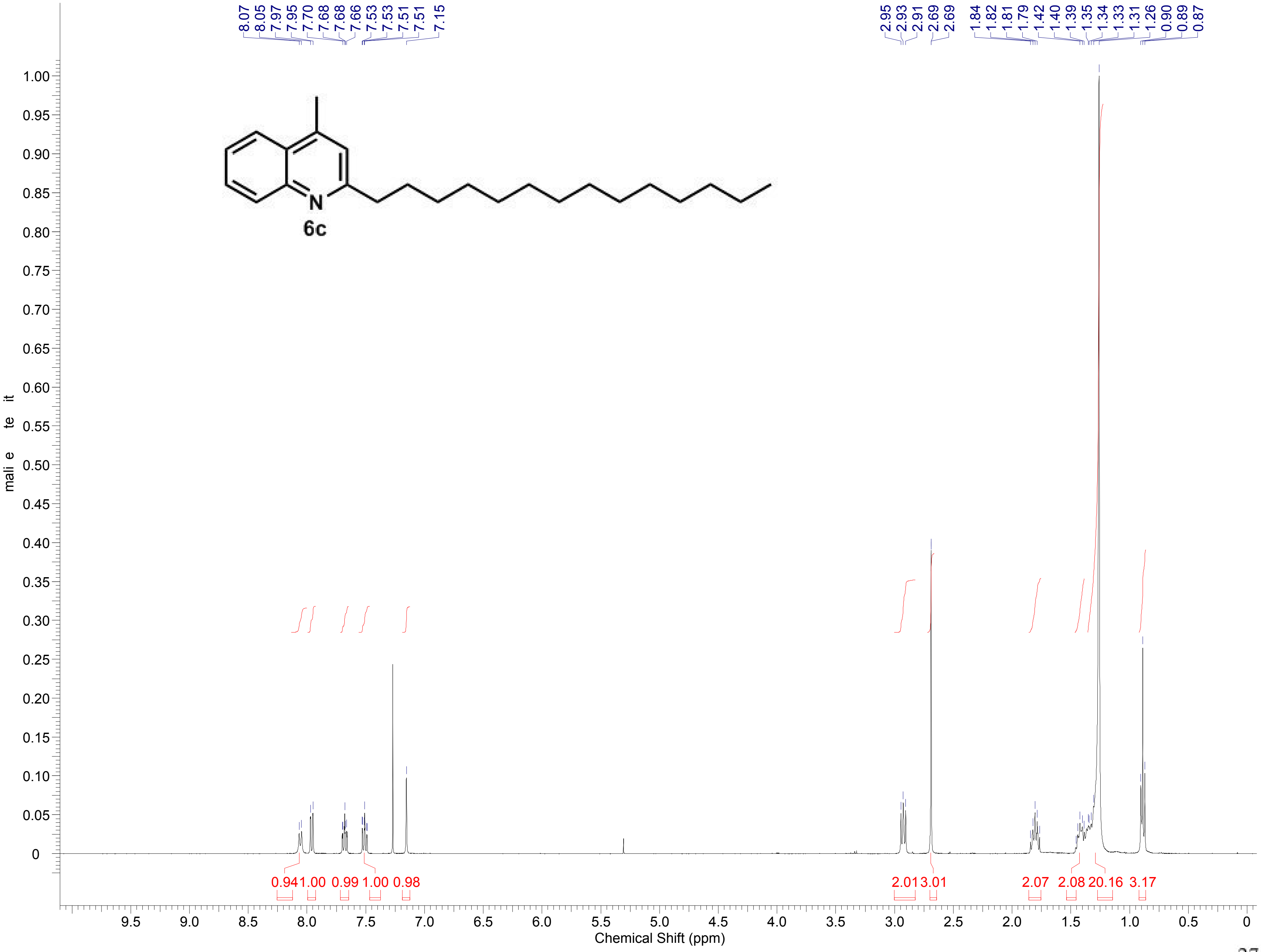

8.07 8.05 7.97 7.95 7.70 7.68 7.68 7.66 7.53 7.53 7.51 7.51 7.15
2.95 2.93 2.91 2.69 2.69
1.84 1.82 1.81 1.79 1.42 1.40 1.39 1.35 1.34 1.33 1.31 1.26 0.90 0.89 0.87
N
6c
1.00 0.95 0.90 0.85 0.80 0.75 0.70 0.65 0.60 0.55 0.50 0.45 0.40 0.35 0.30 0.25 0.20 0.15 0.10 0.05 0
0.94 1.00 0.99 1.00 0.98
2.01 3.01
2.07 2.08 20.16 3.17
9.5 9.0 8.5 8.0 7.5 7.0 6.5 6.0 5.5 5.0 4.5 4.0 3.5 3.0 2.5 2.0 1.5 1.0 0.5 0
Chemical Shift (ppm)

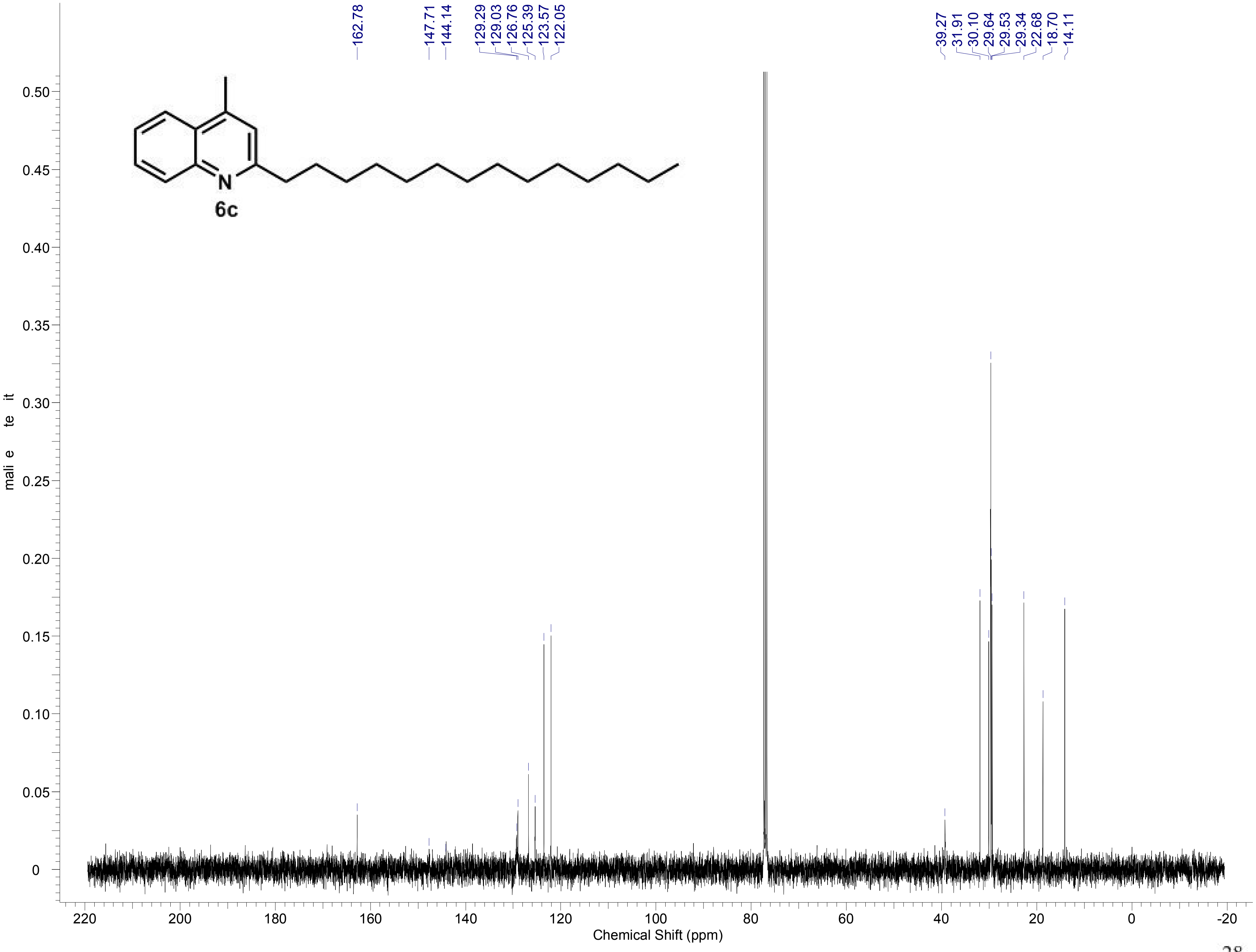

162.78
147.71
144.14
129.29
129.03
126.76
125.39
123.57
122.05
39.27
31.91
30.10
29.64
29.53
29.34
22.68
18.70
14.11
N
6c
0.50
0.45
0.40
0.35
0.30
0.25
0.20
0.15
0.10
0.05
0
mali e te it
220
200
180
160
140
120
100
80
60
40
20
0
-20
Chemical Shift (ppm)

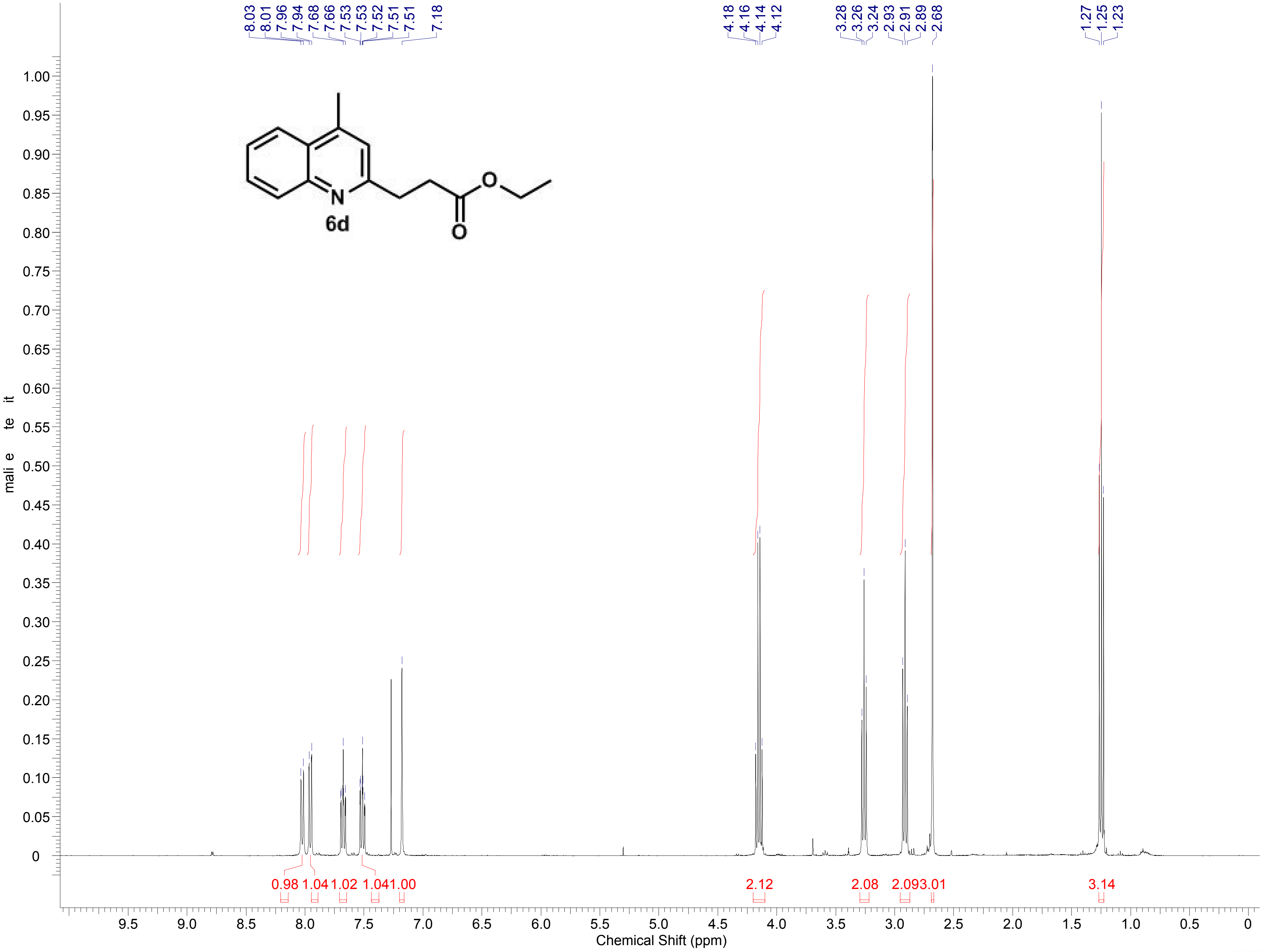
8.03 8.01 7.96 7.94 7.68 7.66 7.53 7.53 7.52 7.51 7.51 7.18
4.18 4.16 4.14 4.12
3.28 3.26 3.24 2.93 2.91 2.89 2.68
1.27 1.25 1.23
6d
0.98 1.04 1.02 1.04 1.00
2.12
2.08
2.09 3.01
3.14
Chemical Shift (ppm)

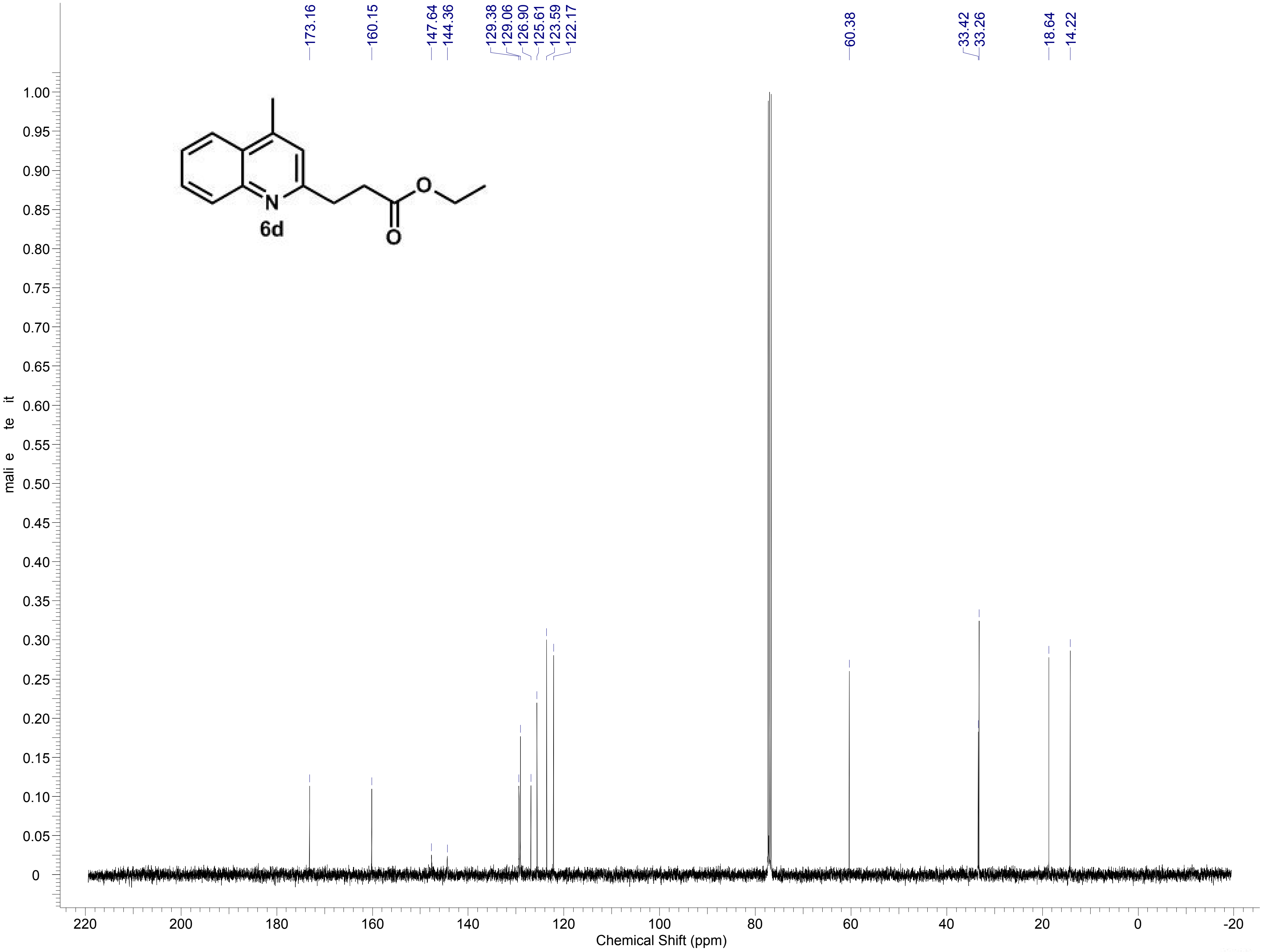

173.16
160.15
147.64
144.36
129.38
129.06
126.90
125.61
123.59
122.17
60.38
33.42
33.26
18.64
14.22
N
O
O
6d
1.00
0.95
0.90
0.85
0.80
0.75
0.70
0.65
0.60
0.55
0.50
0.45
0.40
0.35
0.30
0.25
0.20
0.15
0.10
0.05
0
220
200
180
160
140
120
100
80
60
40
20
0
-20
Chemical Shift (ppm)

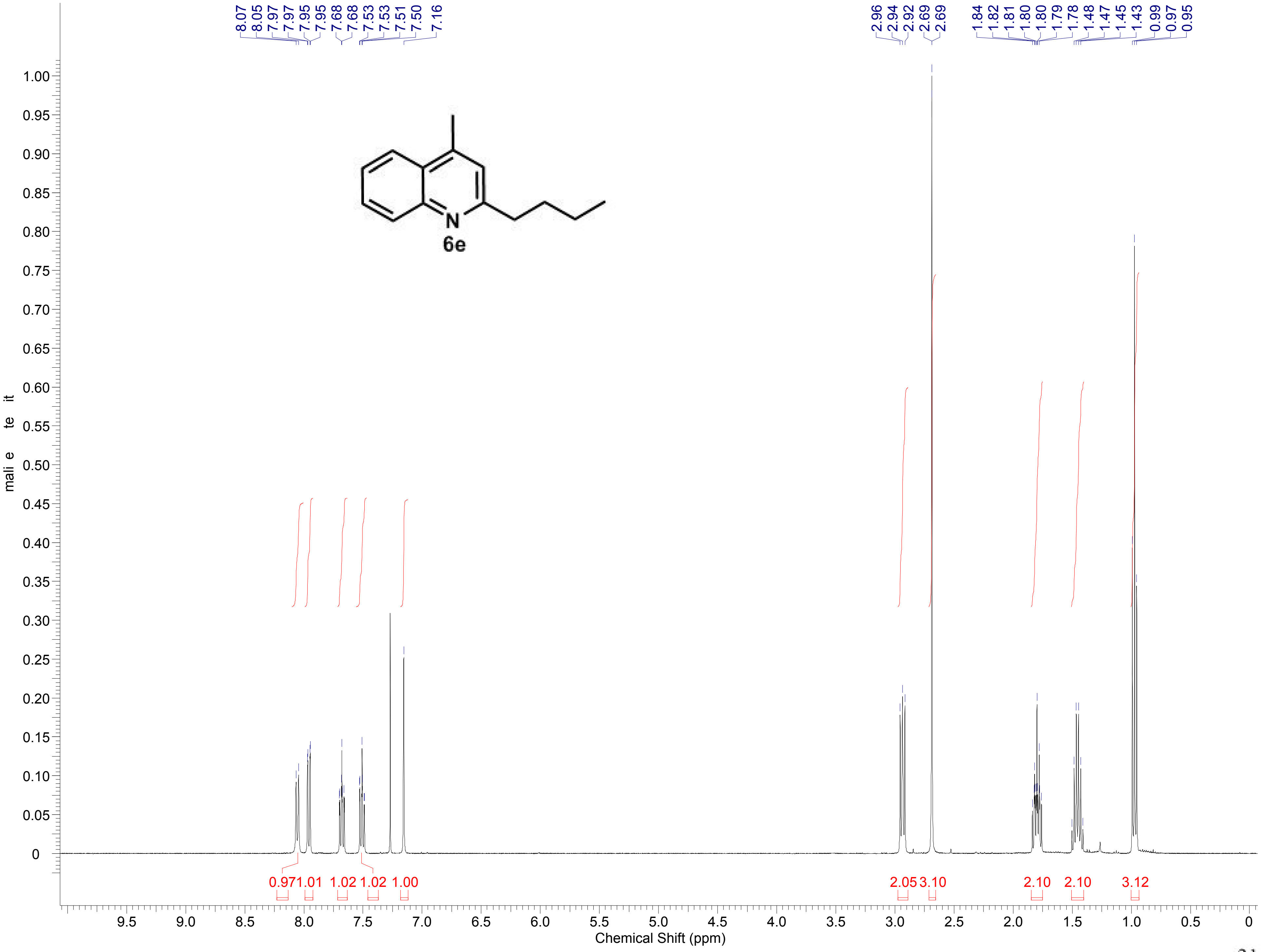
8.07 8.05 7.97 7.97 7.95 7.95 7.68 7.68 7.53 7.53 7.51 7.50 7.16
2.96 2.94 2.92 2.69 2.69
1.84 1.82 1.81 1.80 1.80 1.79 1.78 1.48 1.47 1.45 1.43 0.99 0.97 0.95
N
6e
0.97 1.01 1.02 1.02 1.00
2.05 3.10
2.10 2.10 3.12
Chemical Shift (ppm)

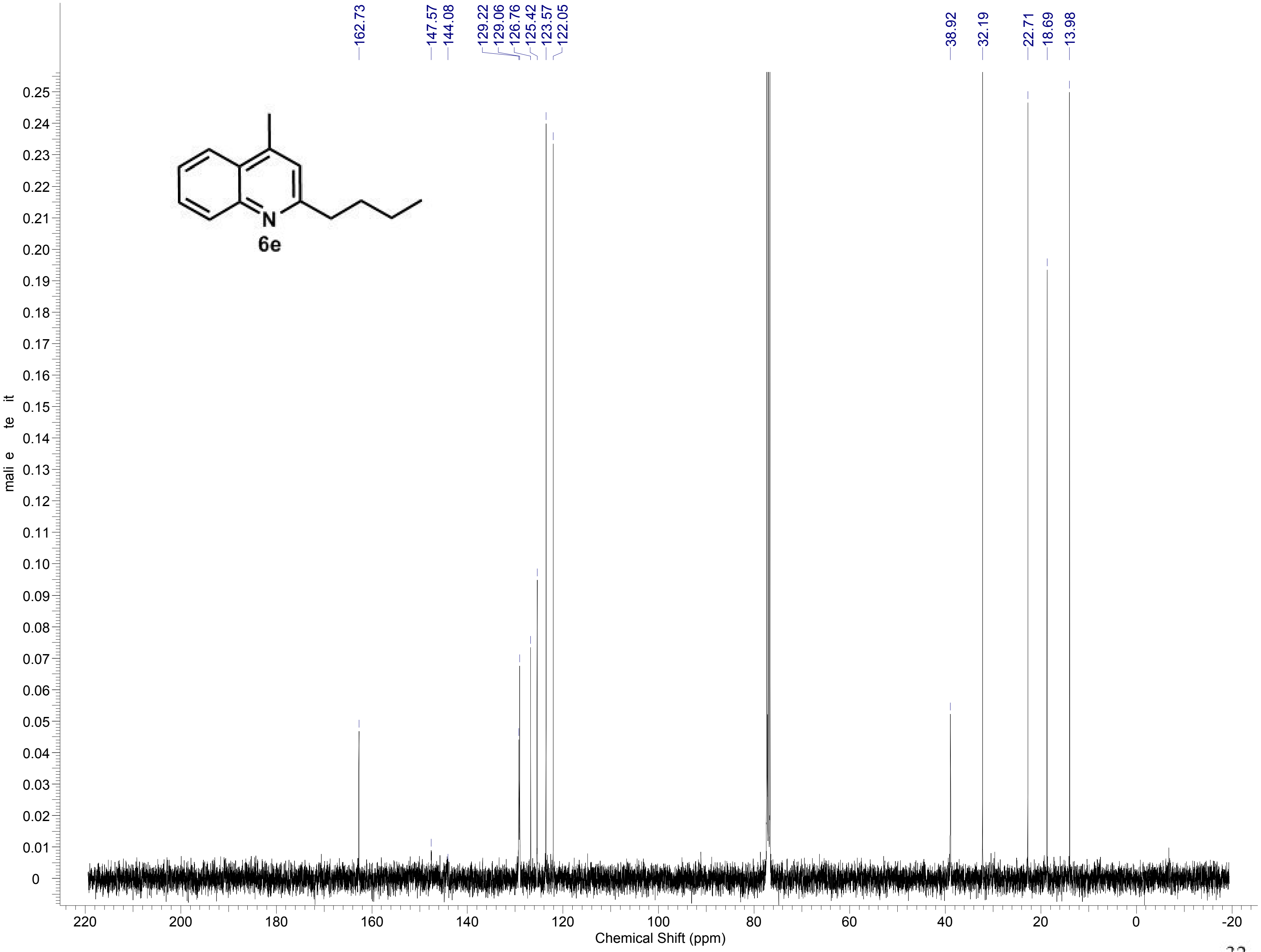
162.73
147.57
144.08
129.22
129.06
126.76
125.42
123.57
122.05
38.92
32.19
22.71
18.69
13.98
N
6e
Chemical Shift (ppm)

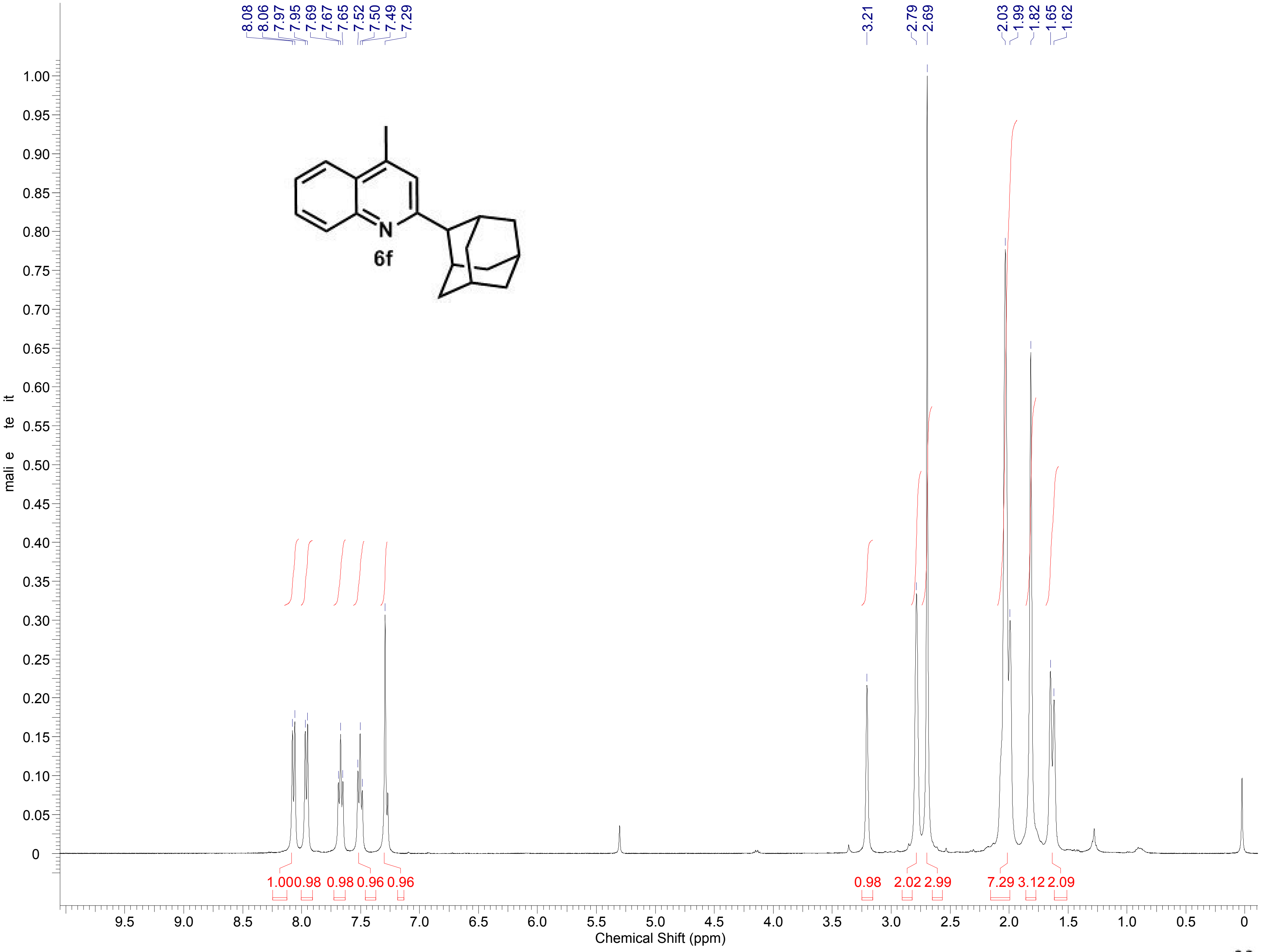

8.08
8.06
7.97
7.95
7.69
7.67
7.65
7.52
7.50
7.49
7.29
3.21
2.79
2.69
2.03
1.99
1.82
1.65
1.62
N
6f
1.00
0.95
0.90
0.85
0.80
0.75
0.70
0.65
0.60
0.55
0.50
0.45
0.40
0.35
0.30
0.25
0.20
0.15
0.10
0.05
0
1.00
0.98
0.98
0.96
0.96
0.98
2.02
2.99
7.29
3.12
2.09
9.5
9.0
8.5
8.0
7.5
7.0
6.5
6.0
5.5
5.0
4.5
4.0
3.5
3.0
2.5
2.0
1.5
1.0
0.5
0
Chemical Shift (ppm)

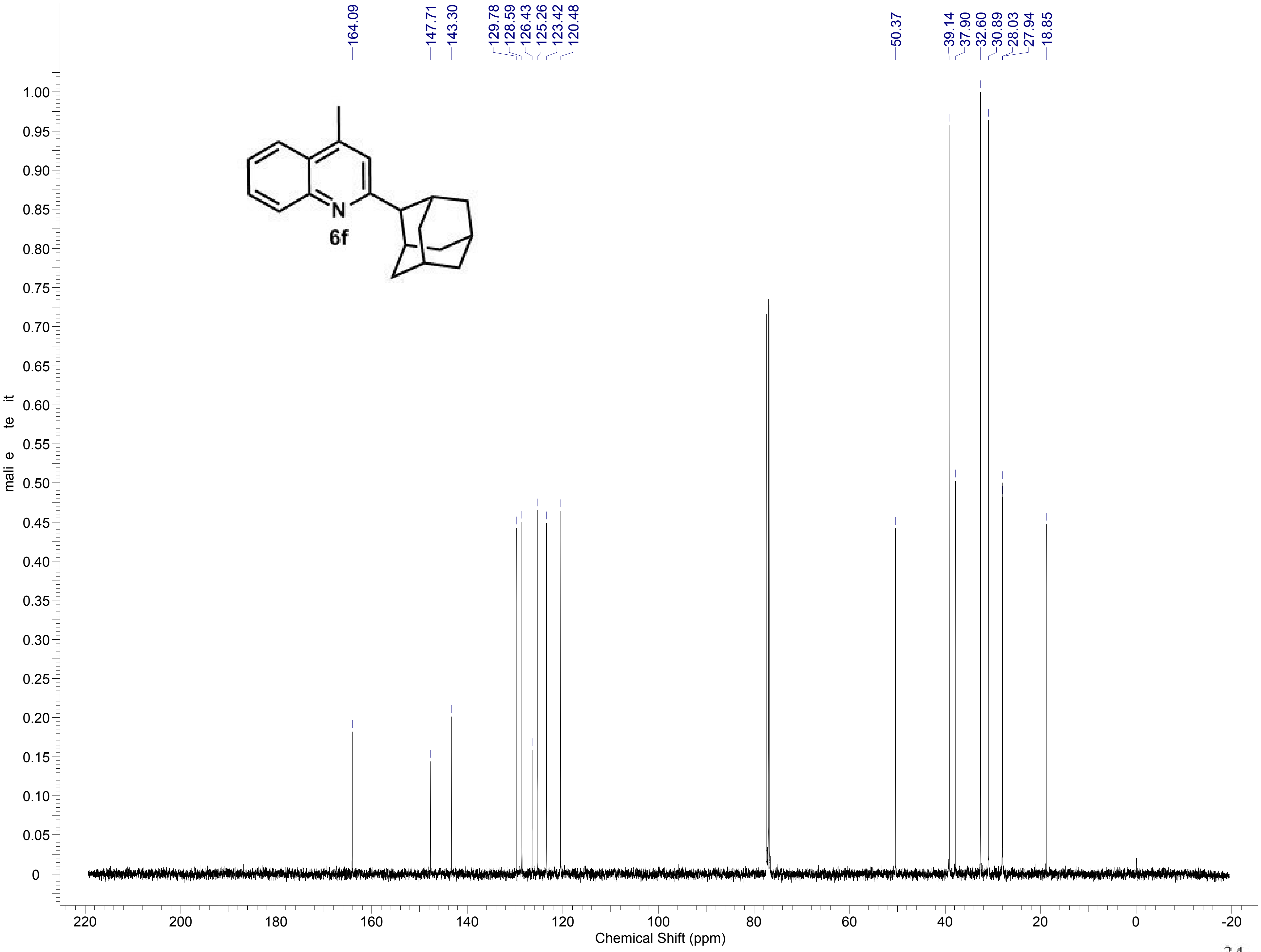
164.09
147.71
143.30
129.78
128.59
126.43
125.26
123.42
120.48
50.37
39.14
37.90
32.60
30.89
28.03
27.94
18.85
6f
Chemical Shift (ppm)

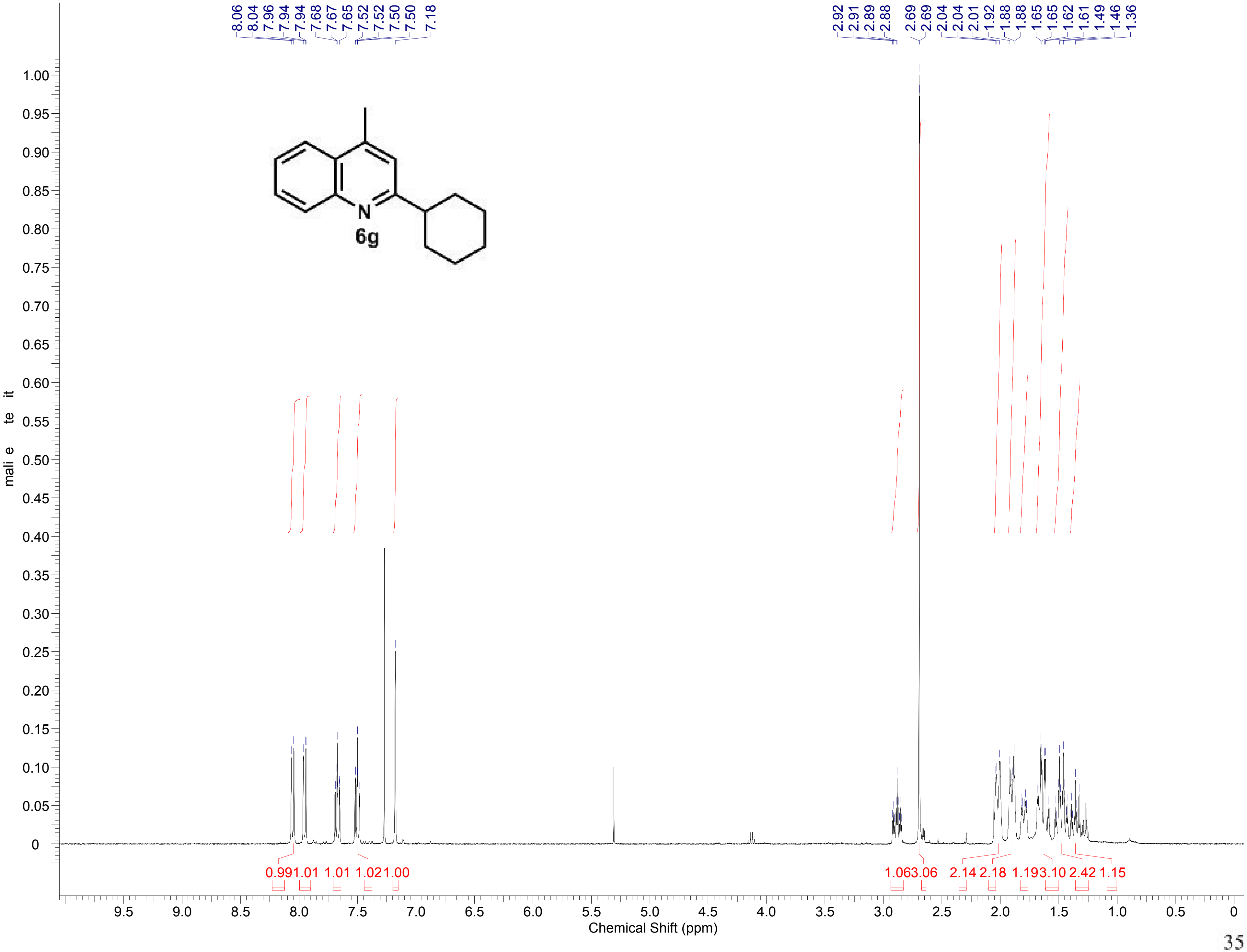

8.06
8.04
7.96
7.94
7.94
7.68
7.67
7.65
7.52
7.52
7.50
7.50
7.18
2.92
2.91
2.89
2.88
2.69
2.69
2.04
2.04
2.01
1.92
1.88
1.88
1.65
1.65
1.62
1.61
1.49
1.46
1.36
N
6g
0.99
1.01
1.01
1.02
1.00
1.06
3.06
2.14
2.18
1.19
3.10
2.42
1.15
Chemical Shift (ppm)

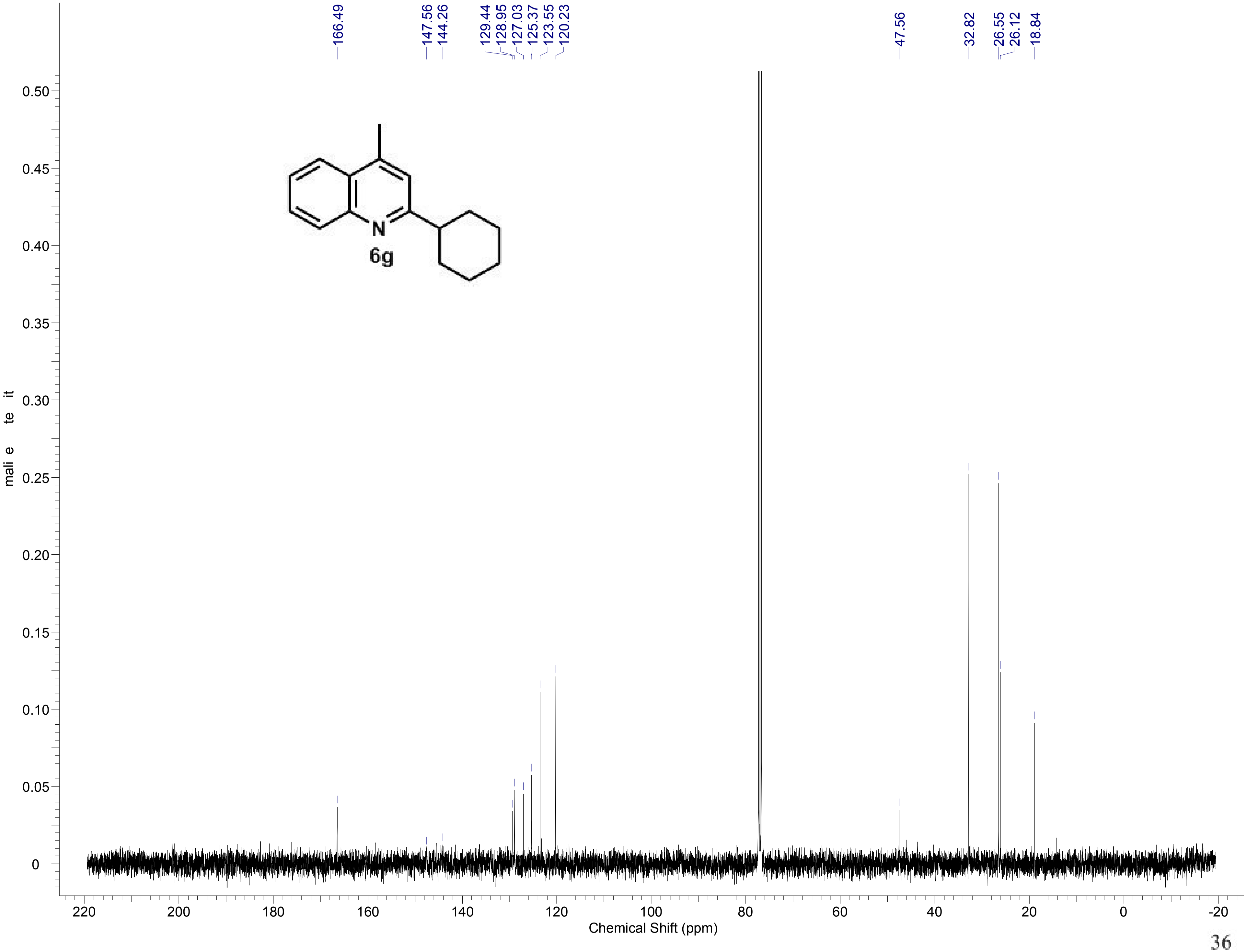

166.49
147.56
144.26
129.44
128.95
127.03
125.37
123.55
120.23
47.56
32.82
26.55
26.12
18.84
N
6g
mali e te it
Chemical Shift (ppm)

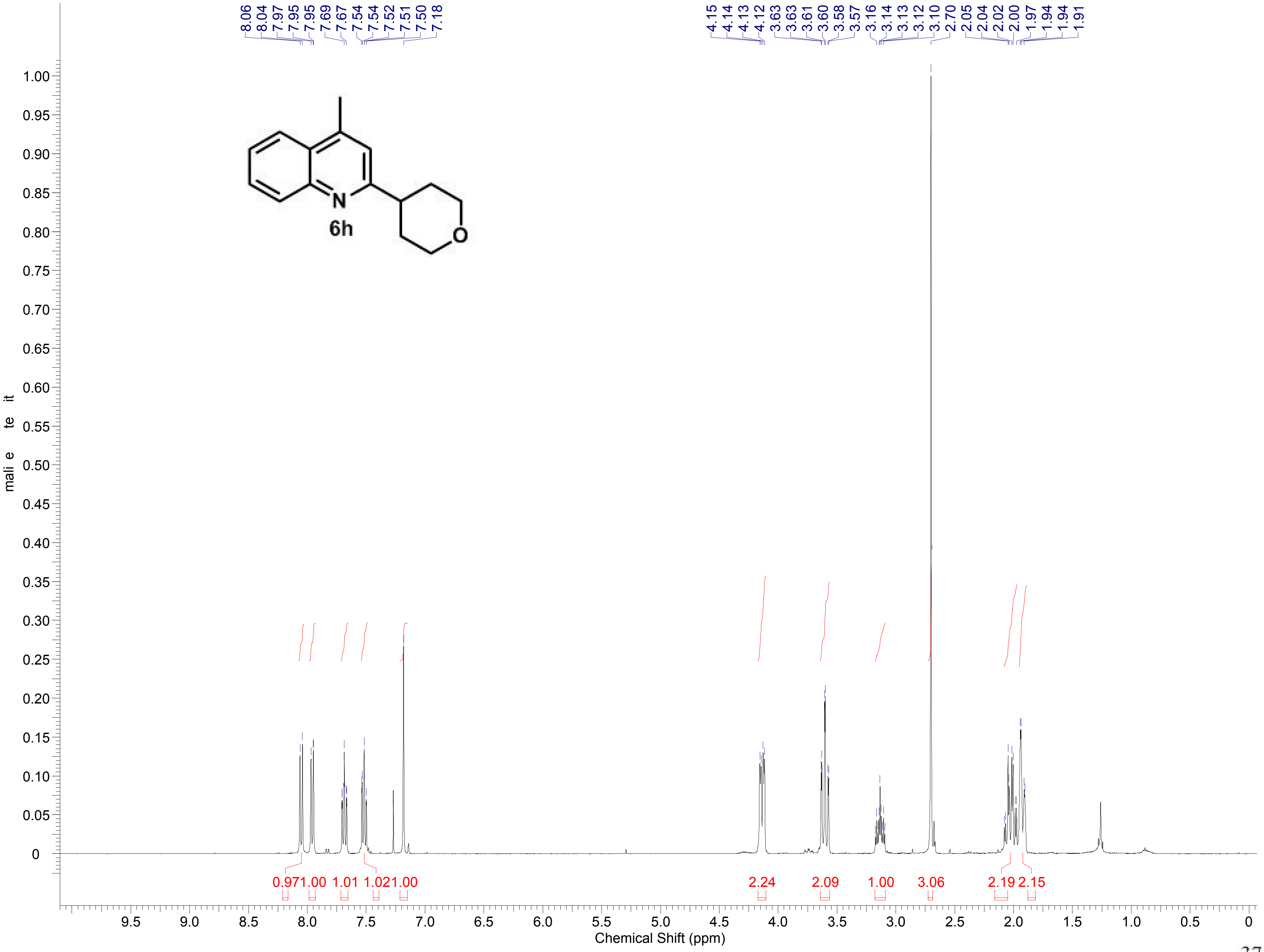
8.06 8.04 7.97 7.95 7.95 7.69 7.67 7.54 7.54 7.52 7.51 7.50 7.18
4.15 4.14 4.13 4.12 3.63 3.63 3.61 3.60 3.58 3.57 3.16 3.14 3.13 3.12 3.10 2.70 2.05 2.04 2.02 2.00 1.97 1.94 1.94 1.91
N
O
6h
0.97 1.00 1.01 1.02 1.00 2.24 2.09 1.00 3.06 2.19 2.15
Chemical Shift (ppm)

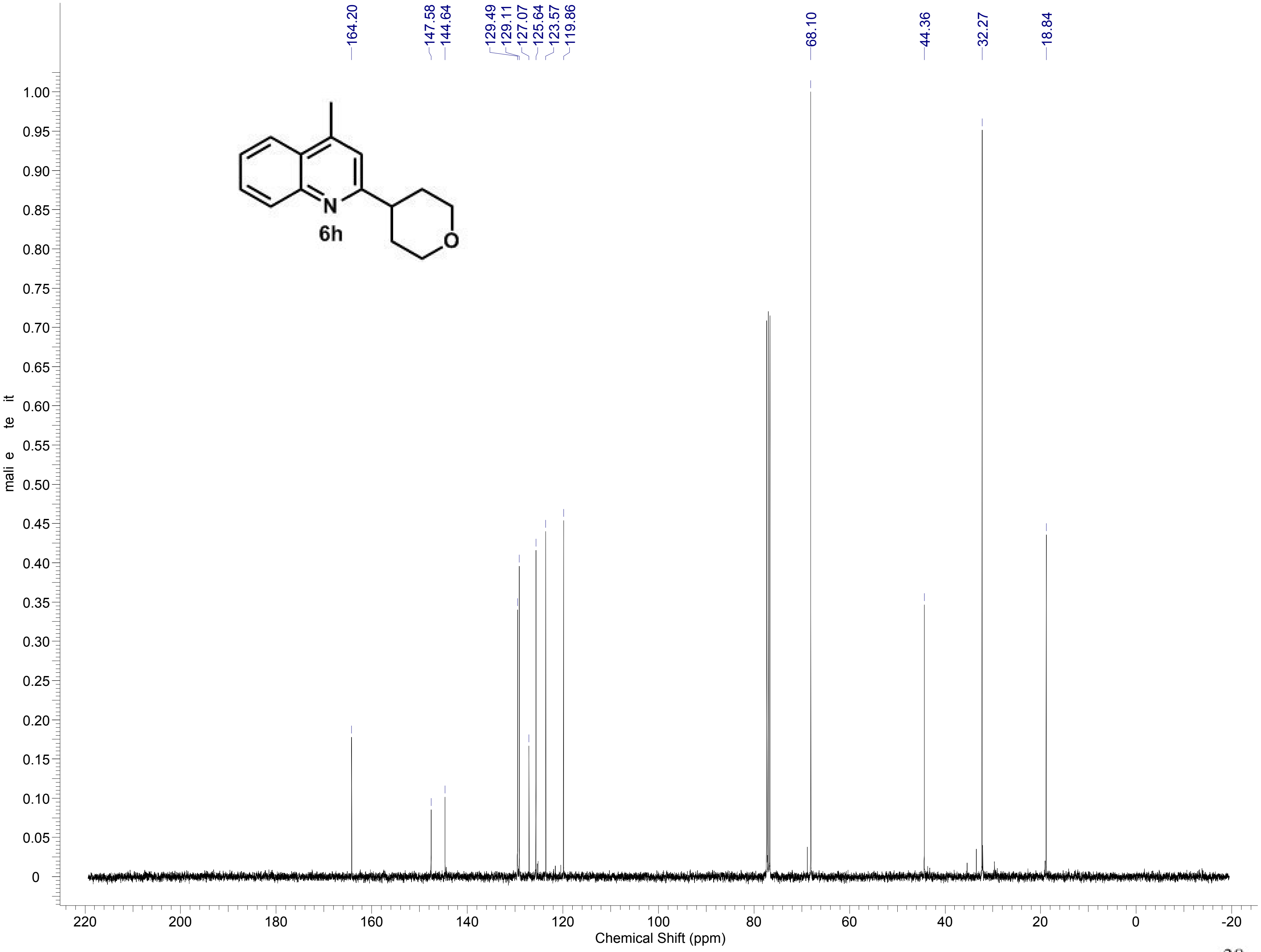
164.20
147.58
144.64
129.49
129.11
127.07
125.64
123.57
119.86
68.10
44.36
32.27
18.84
6h
N
O
Chemical Shift (ppm)

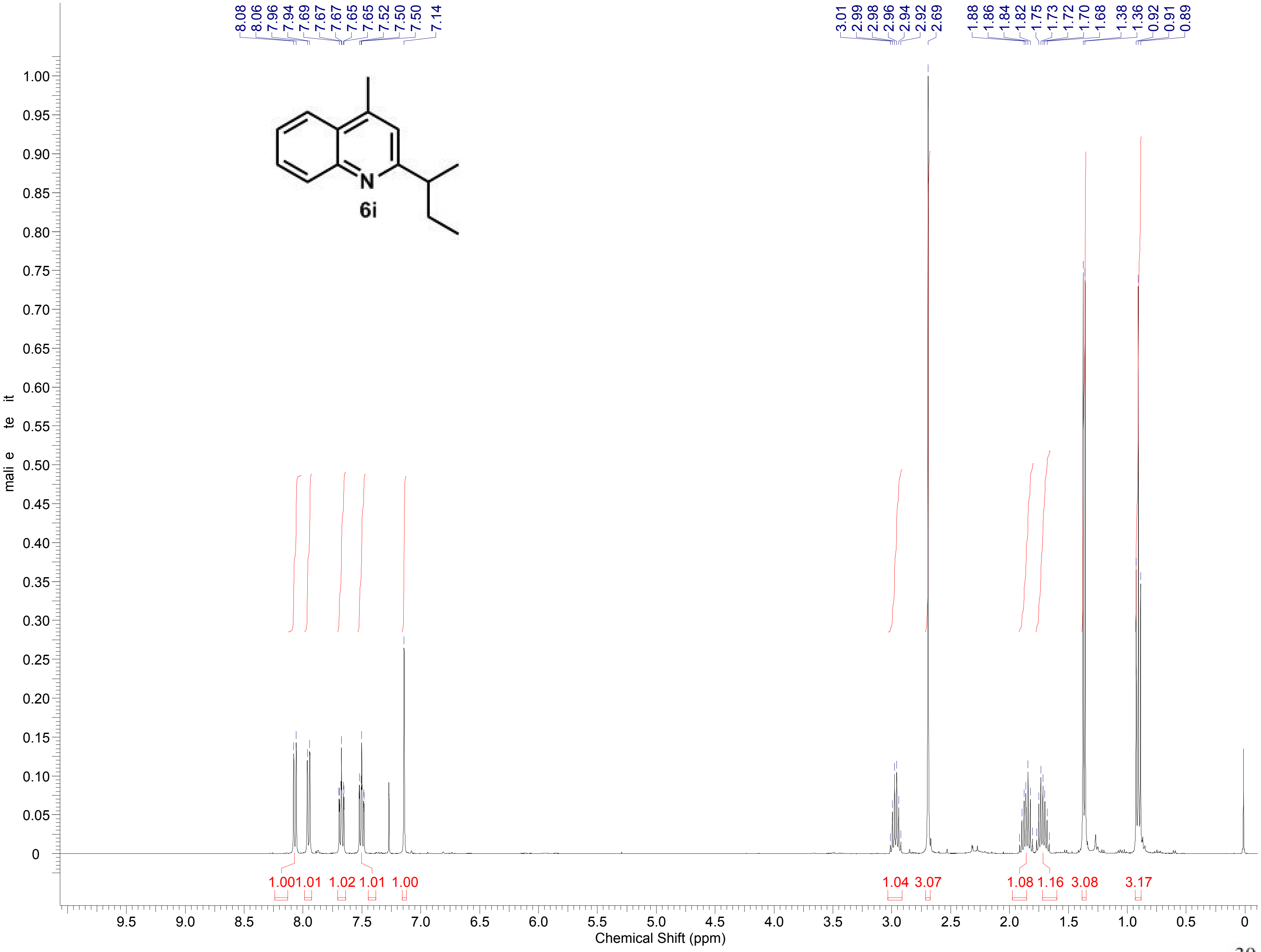
8.08 8.06 7.96 7.94 7.69 7.67 7.67 7.65 7.65 7.52 7.50 7.50 7.14
3.01 2.99 2.98 2.96 2.94 2.92 2.69
1.88 1.86 1.84 1.82 1.75 1.73 1.72 1.70 1.68 1.38 1.36 0.92 0.91 0.89
N
6i
1.00 1.01 1.02 1.01 1.00 1.04 3.07 1.08 1.16 3.08 3.17
Chemical Shift (ppm)

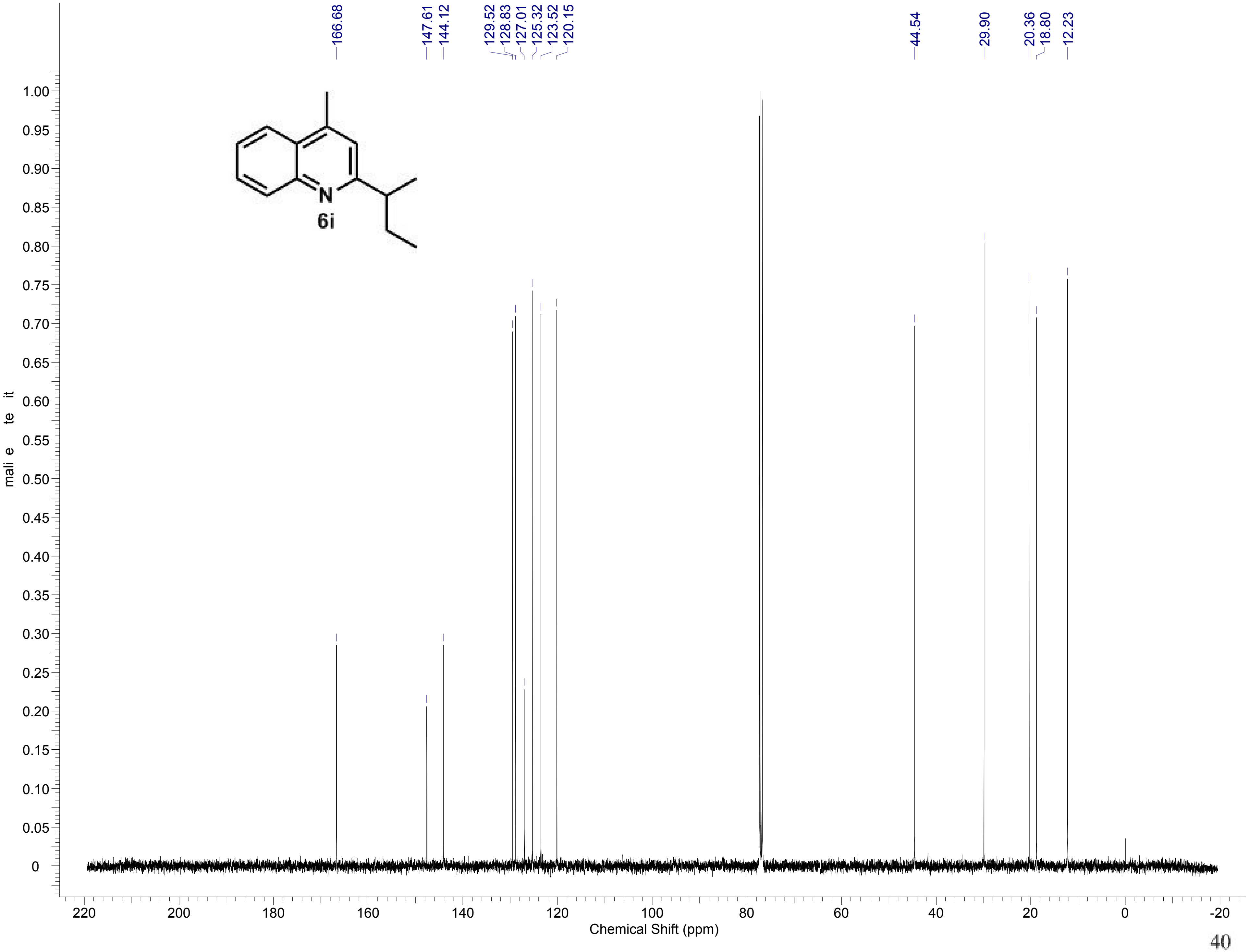
166.68
147.61
144.12
129.52
128.83
127.01
125.32
123.52
120.15
44.54
29.90
20.36
18.80
12.23
N
6i
Chemical Shift (ppm)

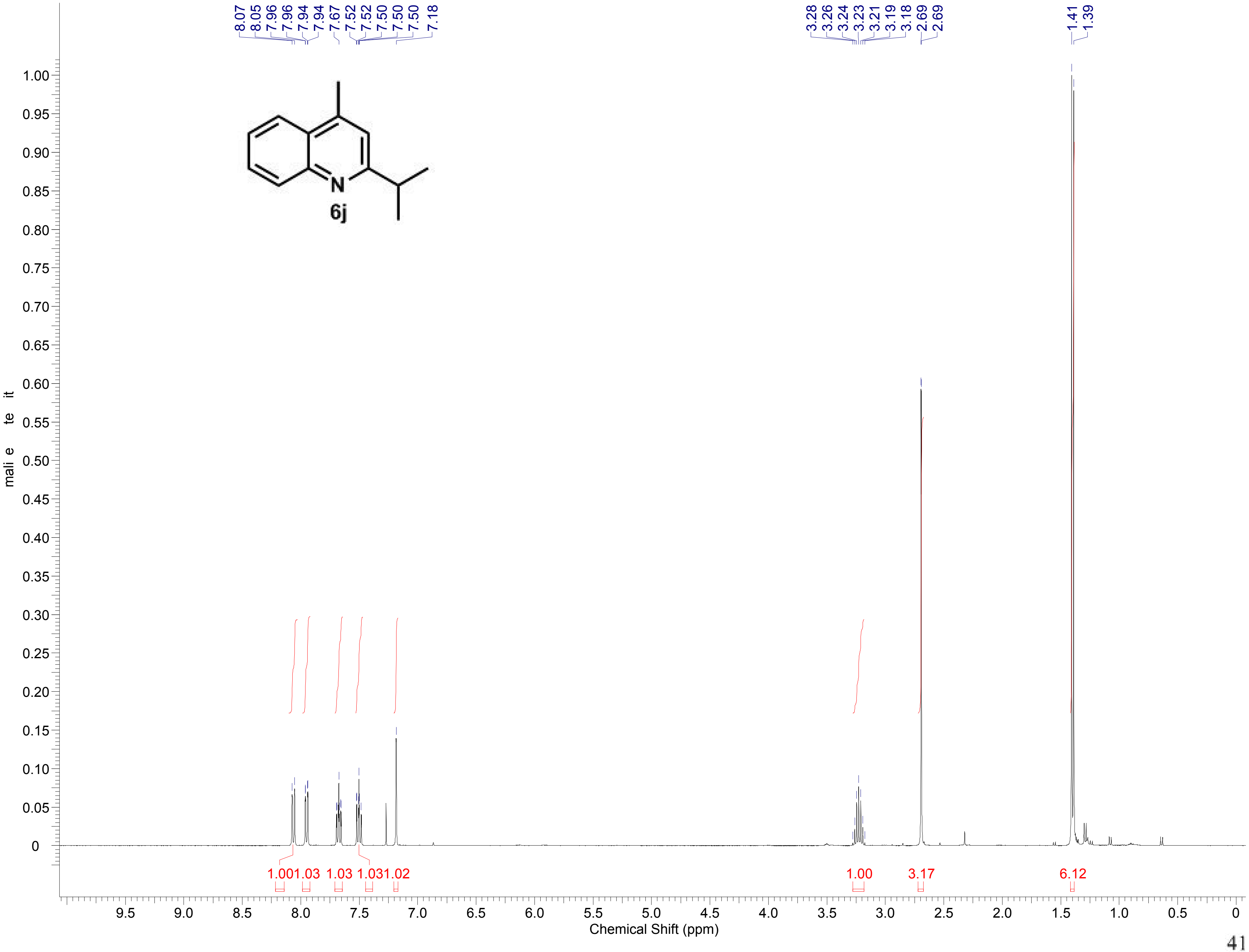
8.07 8.05 7.96 7.96 7.94 7.94 7.67 7.52 7.52 7.50 7.50 7.50 7.18
3.28 3.26 3.24 3.23 3.21 3.19 3.18 2.69 2.69
1.41 1.39
N
6j
1.00 1.03 1.03 1.03 1.02
1.00
3.17
6.12
Chemical Shift (ppm)

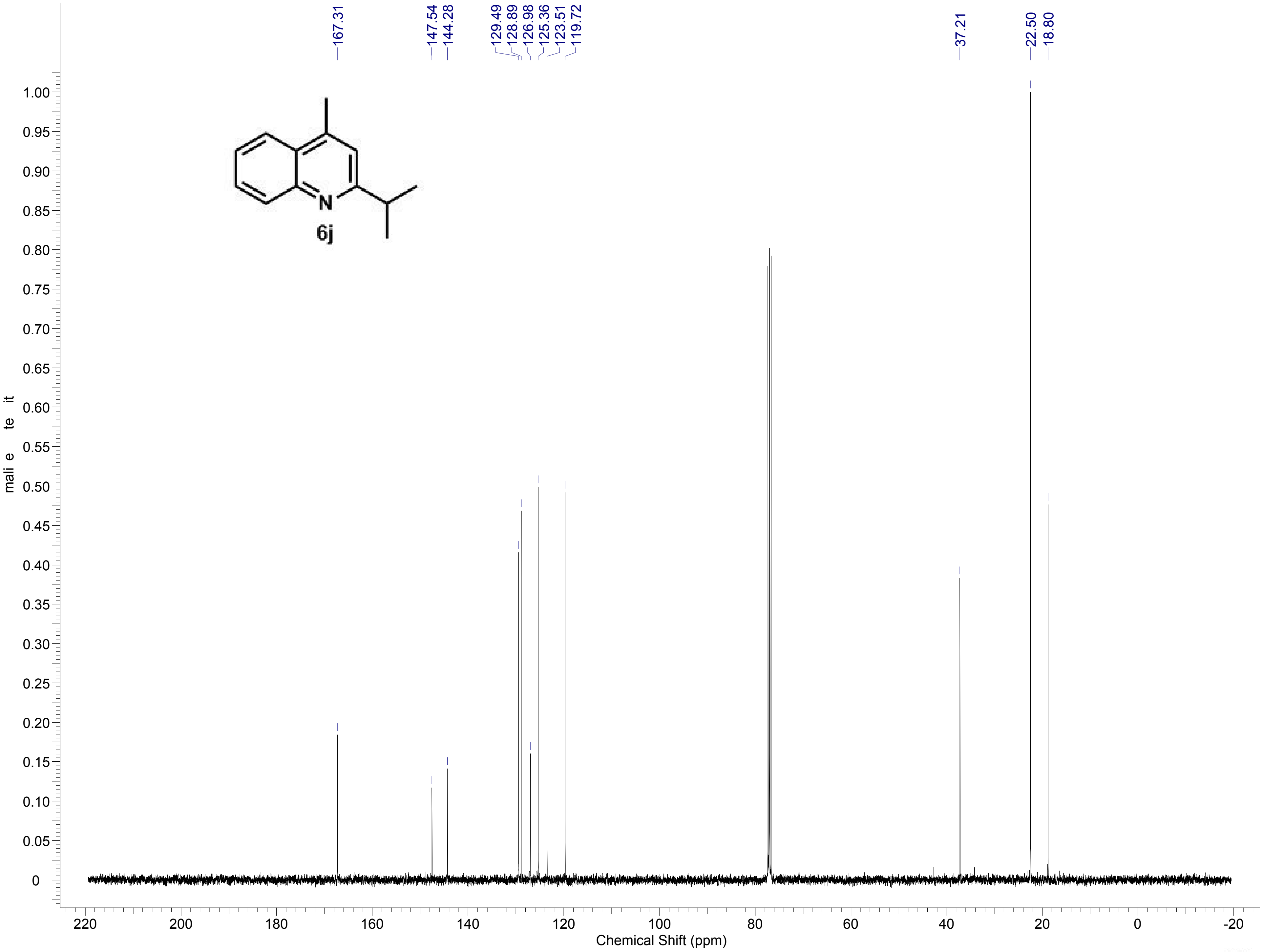
167.31
147.54
144.28
129.49
128.89
126.98
125.36
123.51
119.72
37.21
22.50
18.80
6j
N
Chemical Shift (ppm)

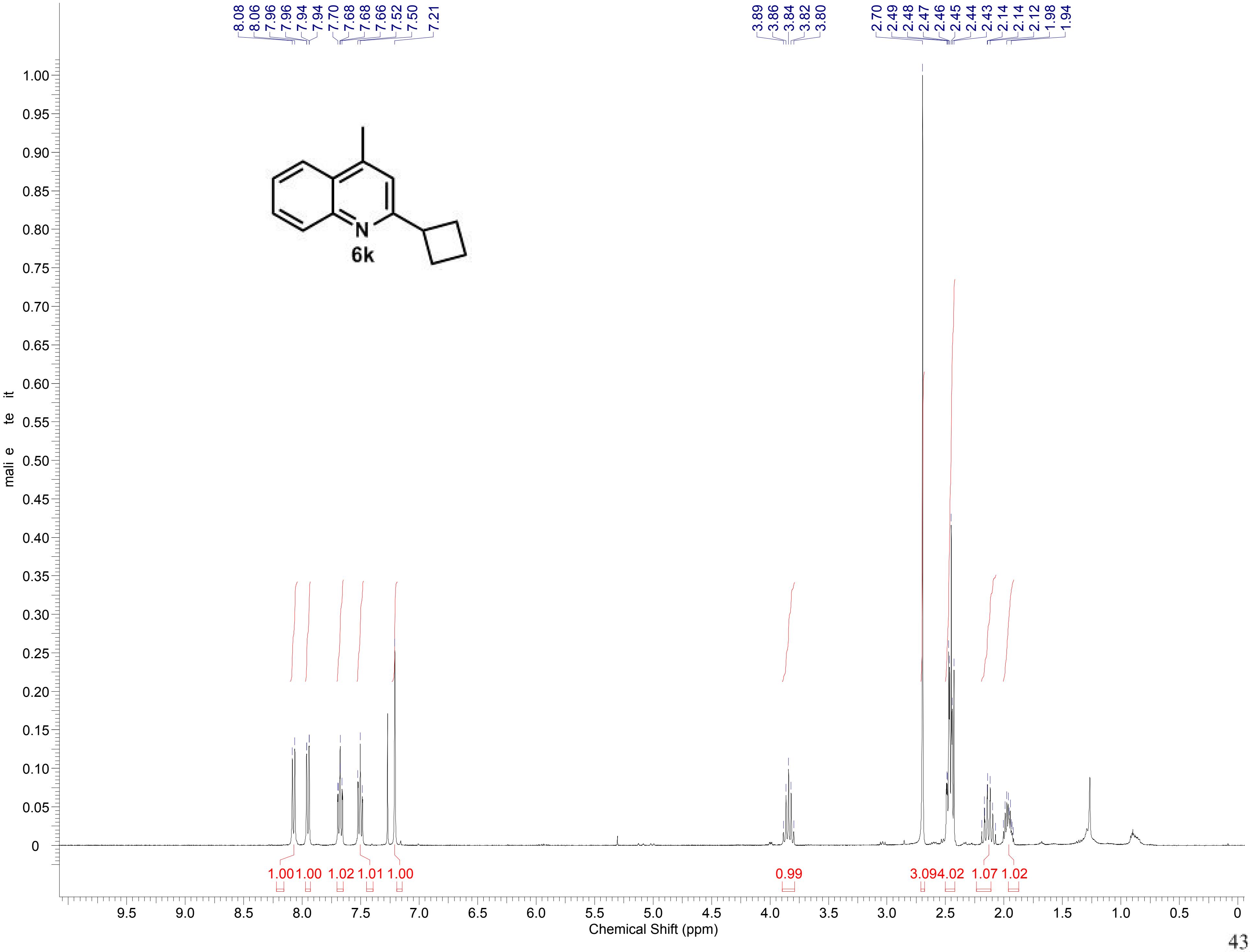
8.08 8.06 7.96 7.96 7.94 7.94 7.70 7.68 7.68 7.66 7.52 7.50 7.21
3.89 3.86 3.84 3.82 3.80
2.70 2.49 2.48 2.47 2.46 2.45 2.44 2.43 2.14 2.14 2.12 1.98 1.94
N
6k
1.00 1.00 1.02 1.01 1.00
0.99
3.09 4.02 1.07 1.02
Chemical Shift (ppm)

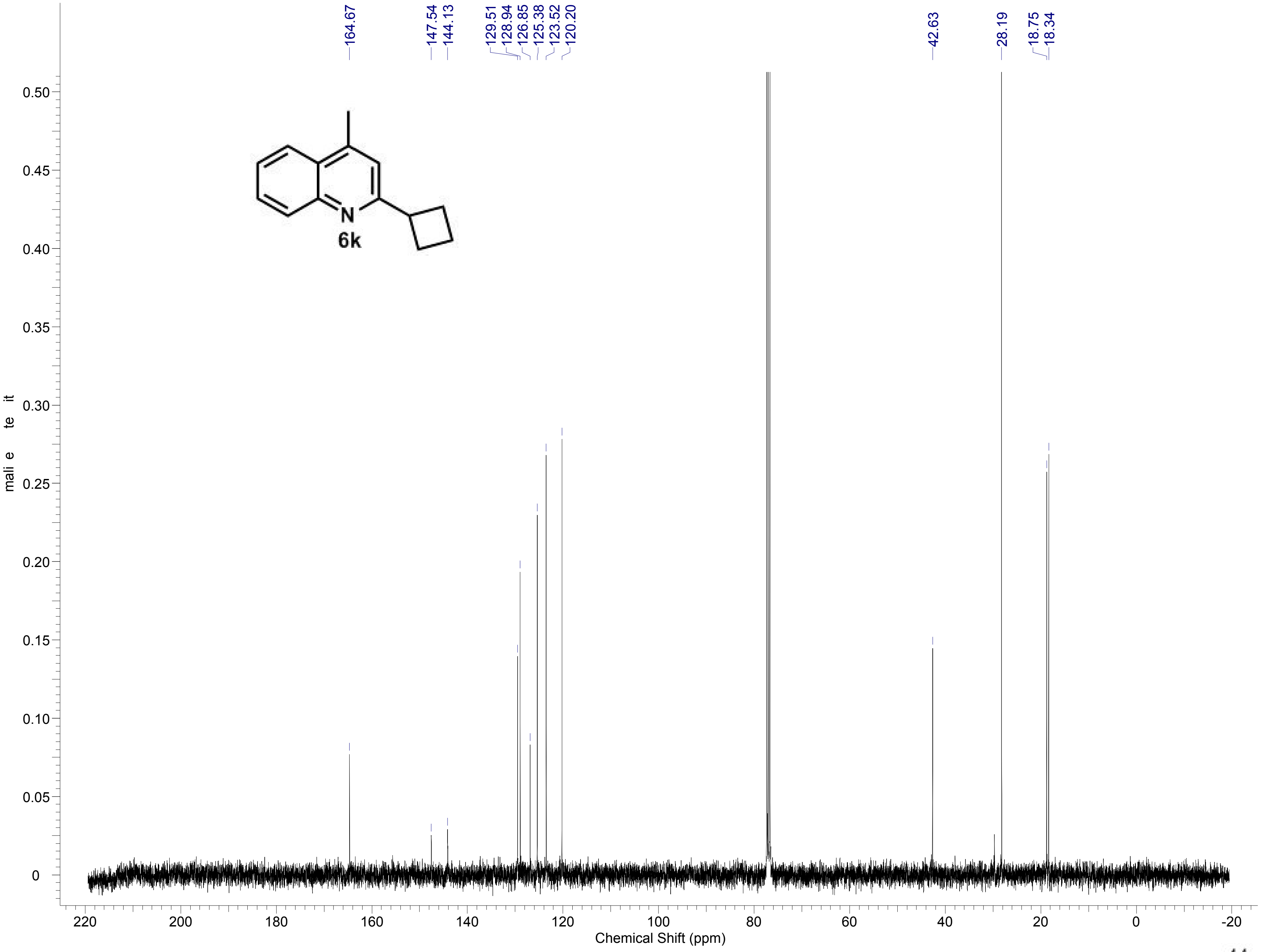
164.67
147.54
144.13
129.51
128.94
126.85
125.38
123.52
120.20
42.63
28.19
18.75
18.34
N
6k
0.50
0.45
0.40
0.35
0.30
0.25
0.20
0.15
0.10
0.05
0
220
200
180
160
140
120
100
80
60
40
20
0
-20
Chemical Shift (ppm)

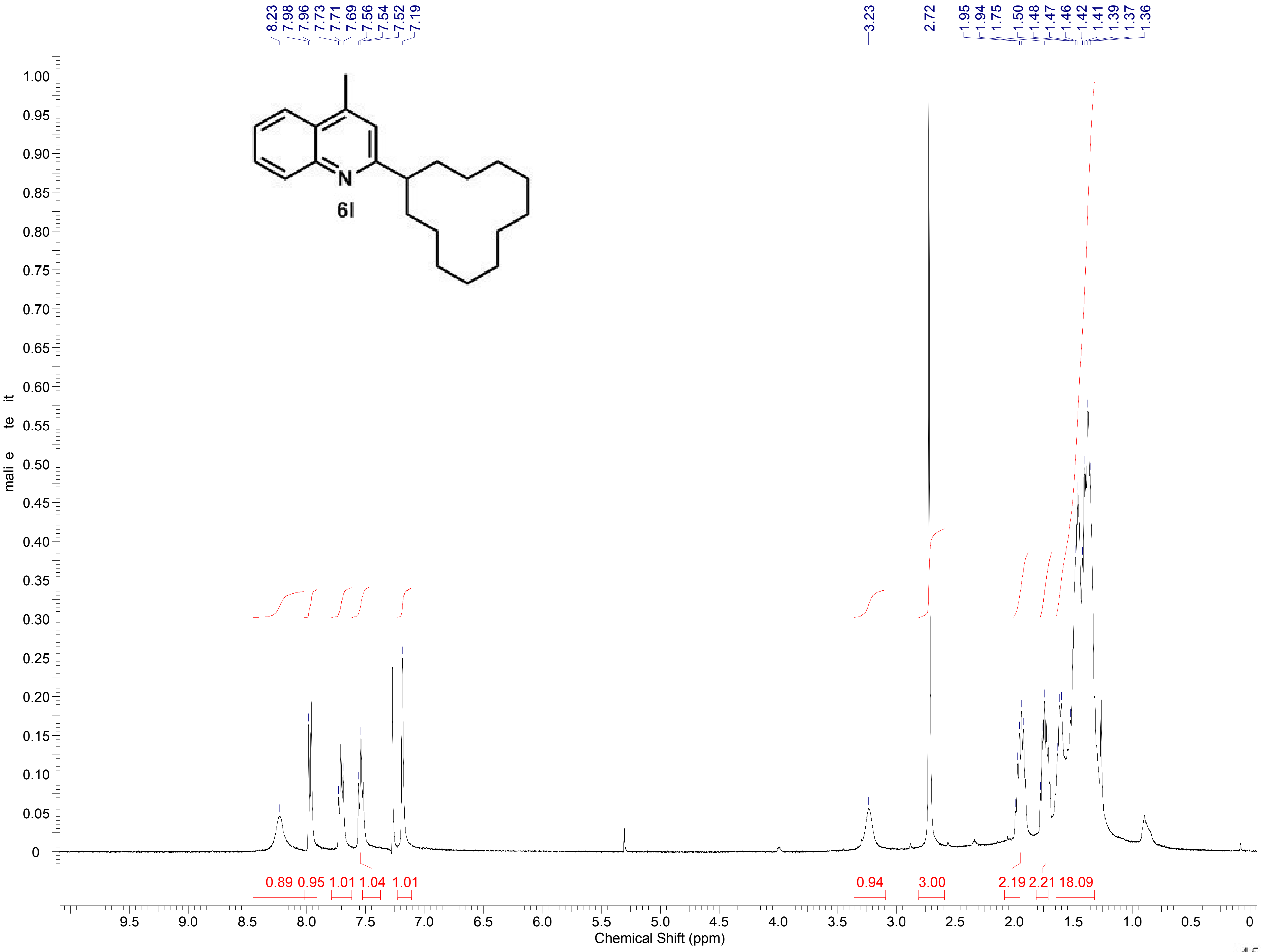
8.23
7.98
7.96
7.73
7.71
7.69
7.56
7.54
7.52
7.19
3.23
2.72
1.95
1.94
1.75
1.50
1.48
1.47
1.46
1.42
1.41
1.39
1.37
1.36
N
6l
0.89 0.95 1.01 1.04 1.01
0.94
3.00
2.19 2.21 18.09
Chemical Shift (ppm)

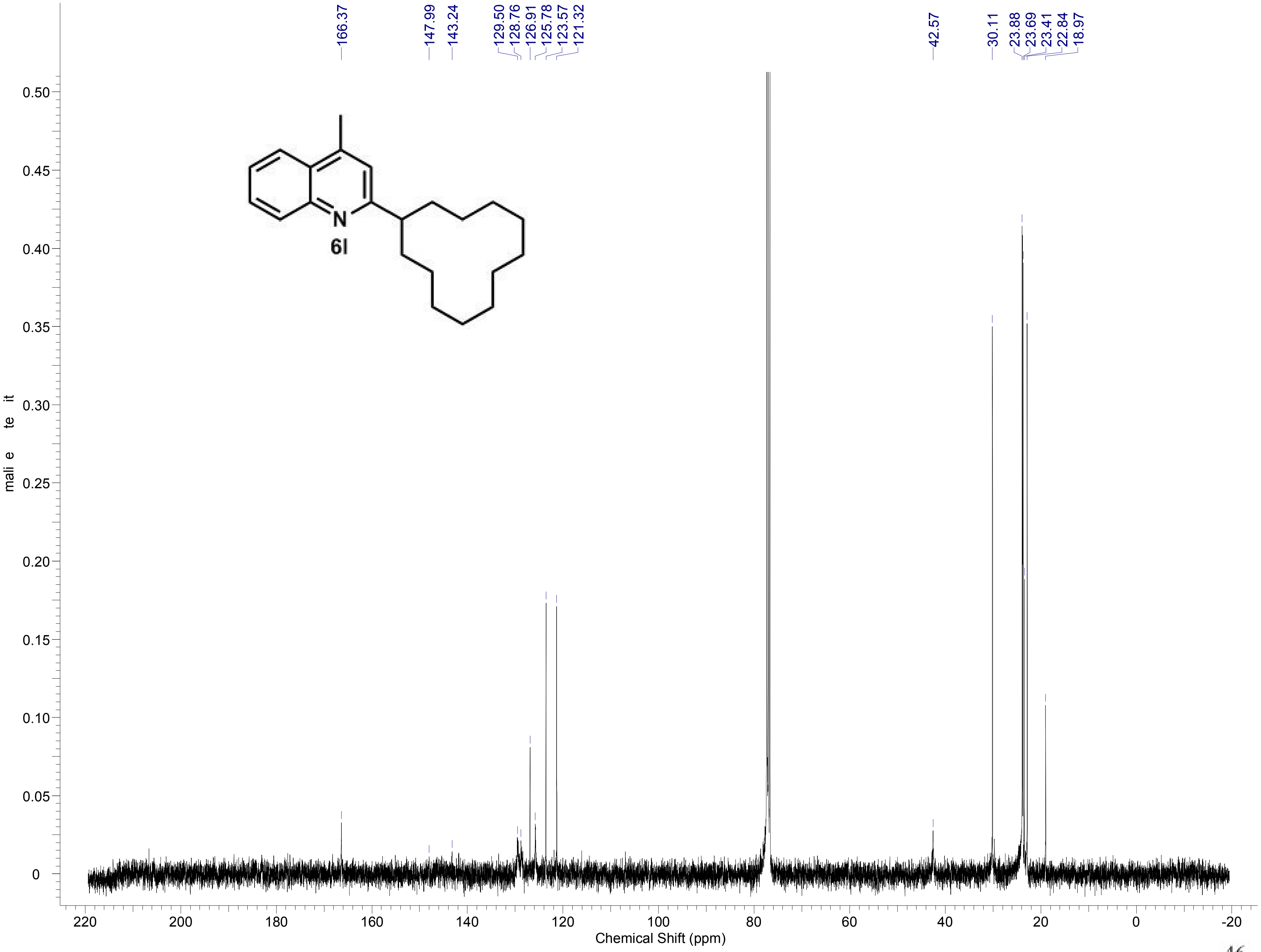

166.37
147.99
143.24
129.50
128.76
126.91
125.78
123.57
121.32
42.57
30.11
23.88
23.69
23.41
22.84
18.97
N
6l
mali e te it
Chemical Shift (ppm)

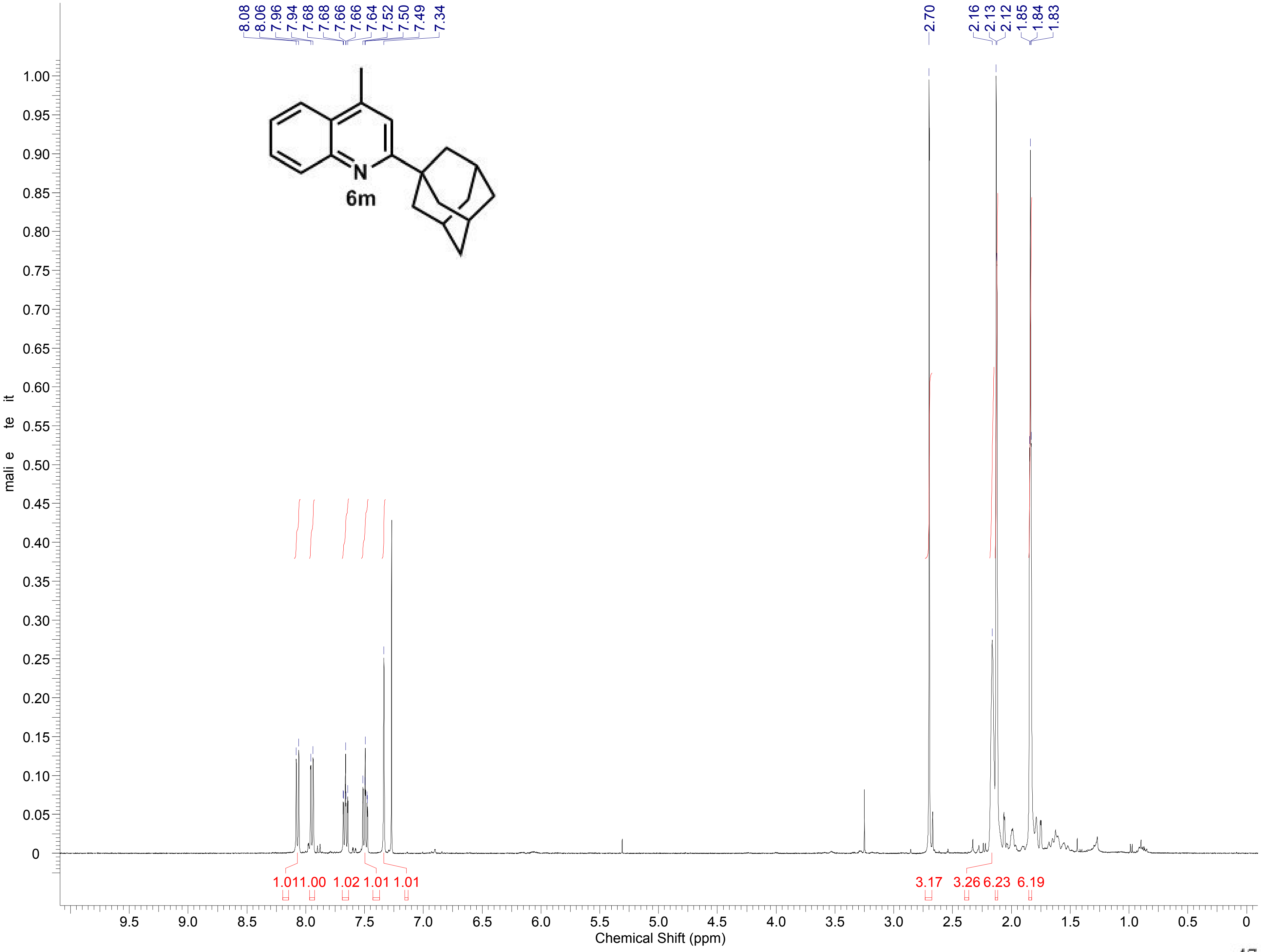

8.08
8.06
7.96
7.94
7.68
7.68
7.66
7.66
7.64
7.52
7.50
7.49
7.34
2.70
2.16
2.13
2.12
1.85
1.84
1.83
N
6m
1.01
1.00
1.02
1.01
1.01
3.17
3.26
6.23
6.19
Chemical Shift (ppm)

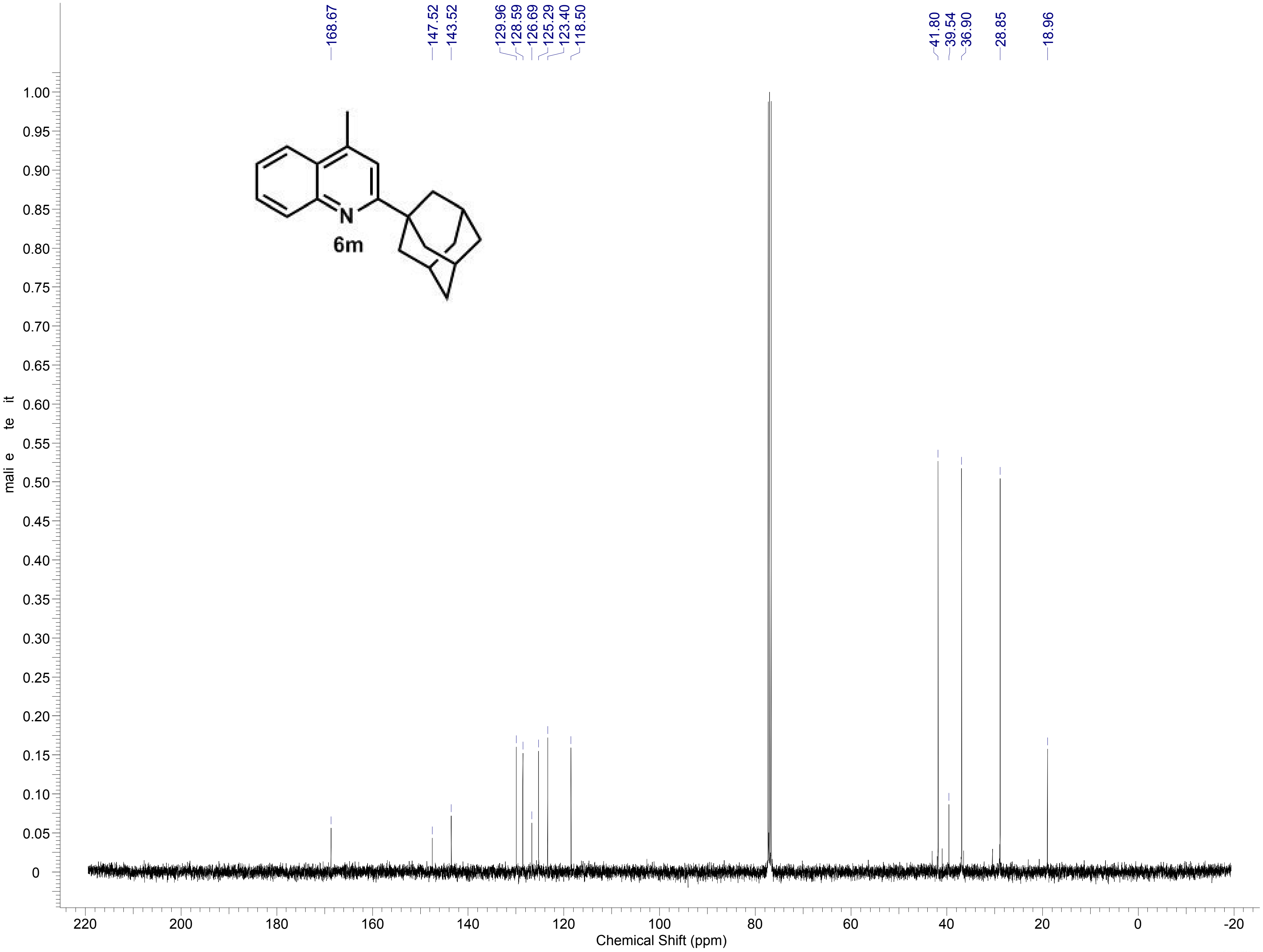
168.67
147.52
143.52
129.96
128.59
126.69
125.29
123.40
118.50
41.80
39.54
36.90
28.85
18.96
N
6m
Chemical Shift (ppm)

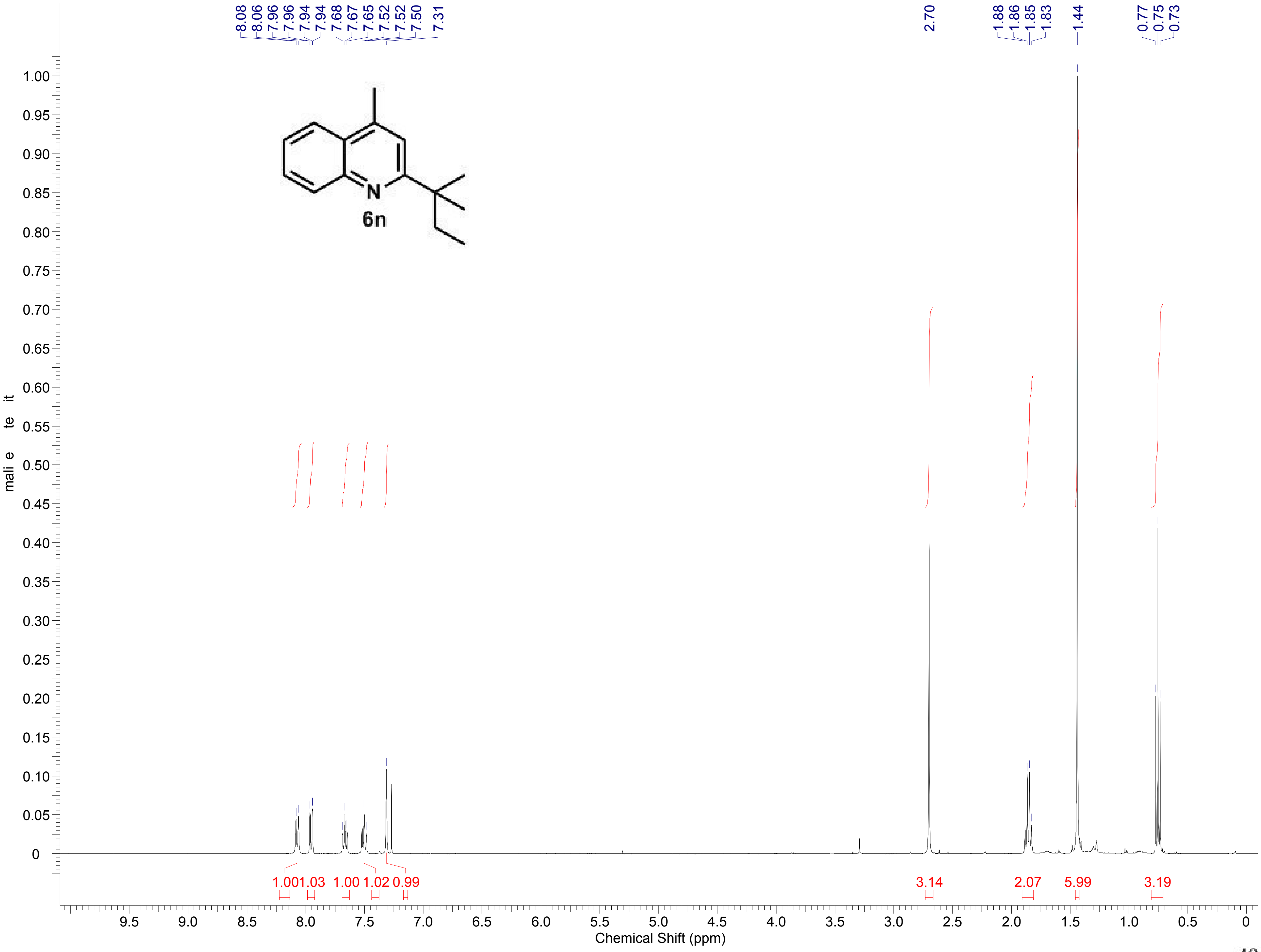
8.08
8.06
7.96
7.96
7.94
7.94
7.68
7.67
7.65
7.52
7.52
7.50
7.31
2.70
1.88
1.86
1.85
1.83
1.44
0.77
0.75
0.73
N
6n
1.00
1.03
1.00
1.02
0.99
3.14
2.07
5.99
3.19
Chemical Shift (ppm)

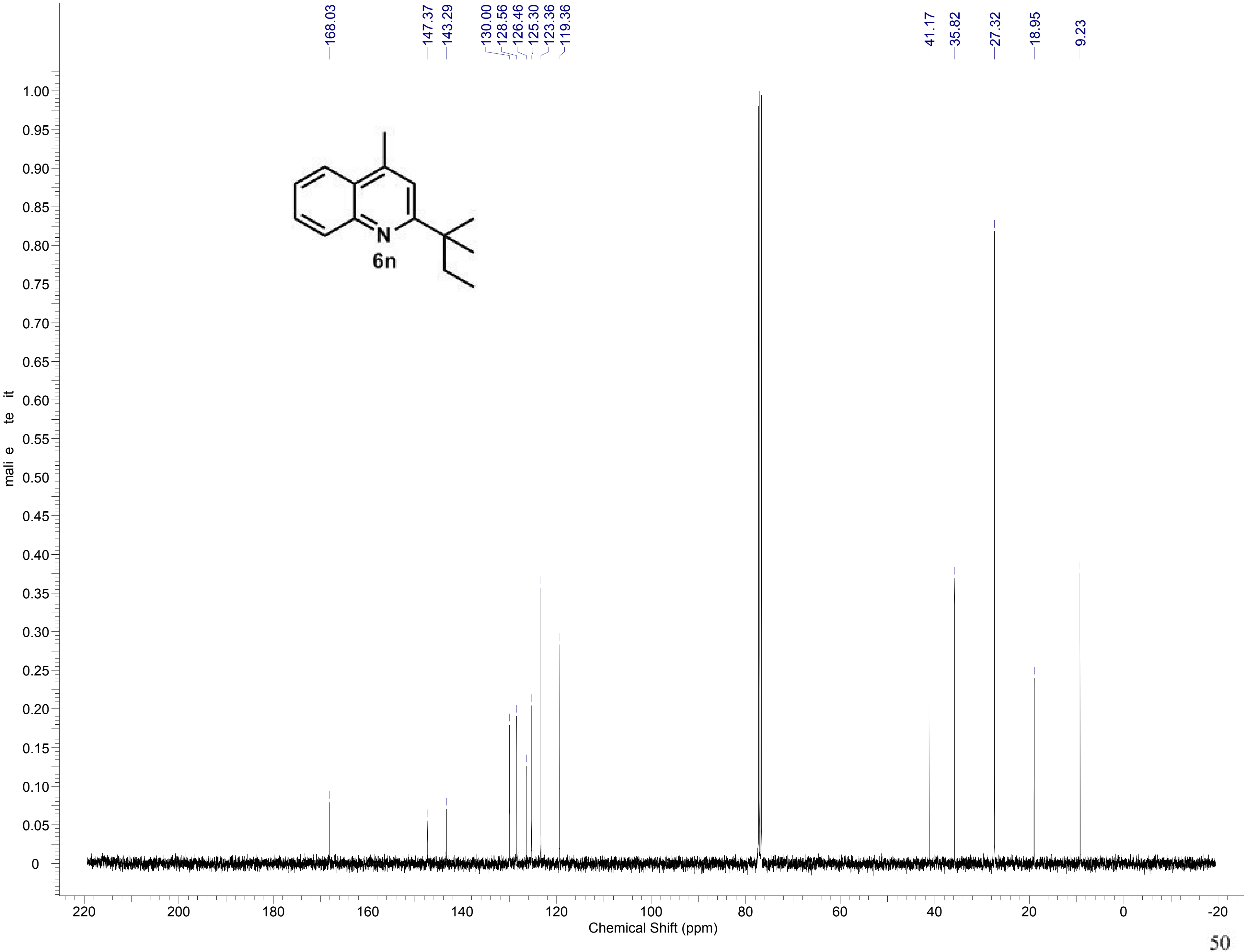
168.03
147.37
143.29
130.00
128.56
126.46
125.30
123.36
119.36
41.17
35.82
27.32
18.95
9.23
N
6n
Chemical Shift (ppm)

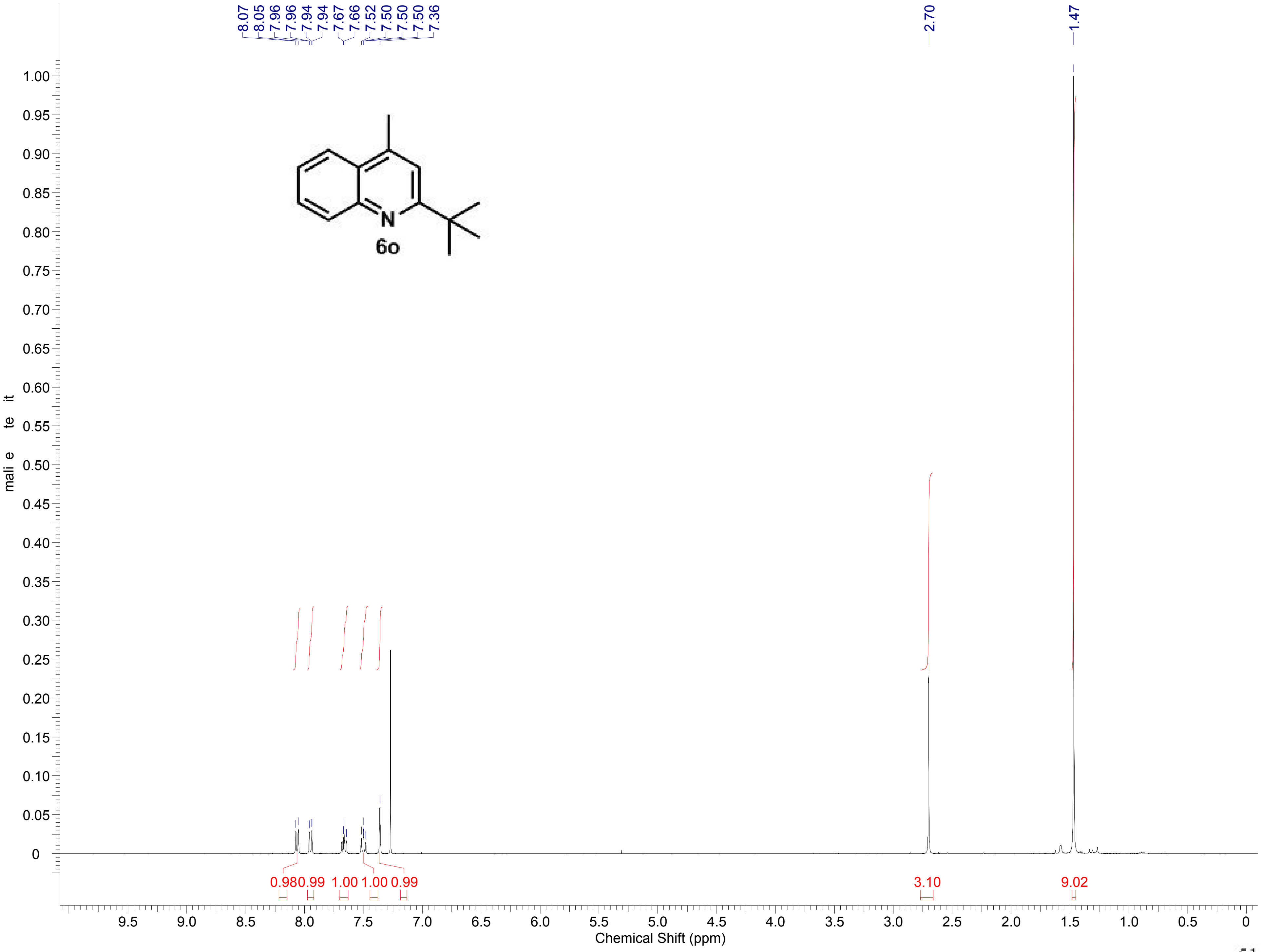
8.07
8.05
7.96
7.96
7.94
7.94
7.67
7.66
7.52
7.50
7.50
7.50
7.36
2.70
1.47
6o
N
0.98
0.99
1.00
1.00
0.99
3.10
9.02
Chemical Shift (ppm)

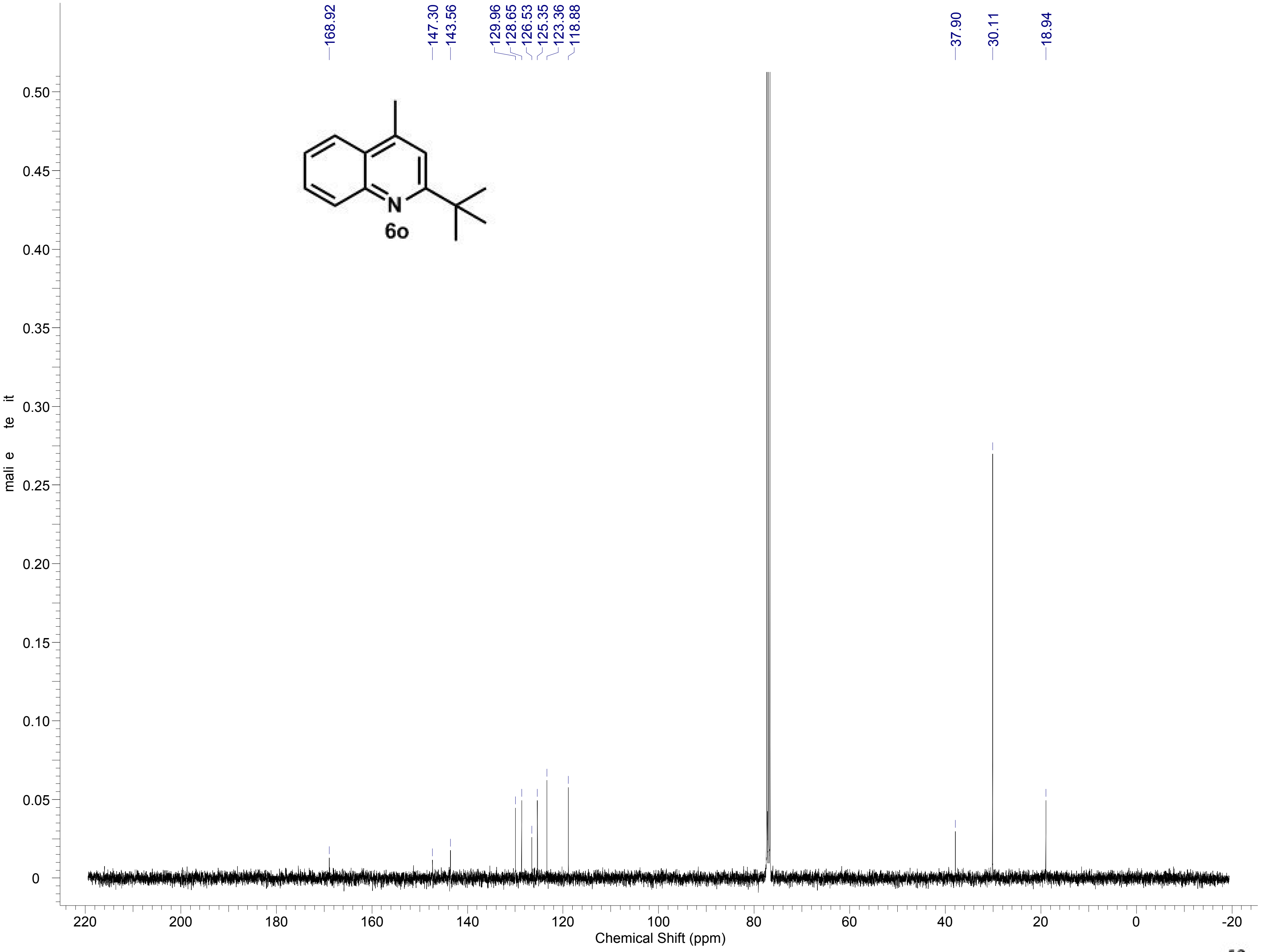

168.92
147.30
143.56
129.96
128.65
126.53
125.35
123.36
118.88
37.90
30.11
18.94
N
6o
0.50
0.45
0.40
0.35
0.30
0.25
0.20
0.15
0.10
0.05
0
220 200 180 160 140 120 100 80 60 40 20 0 -20
Chemical Shift (ppm)

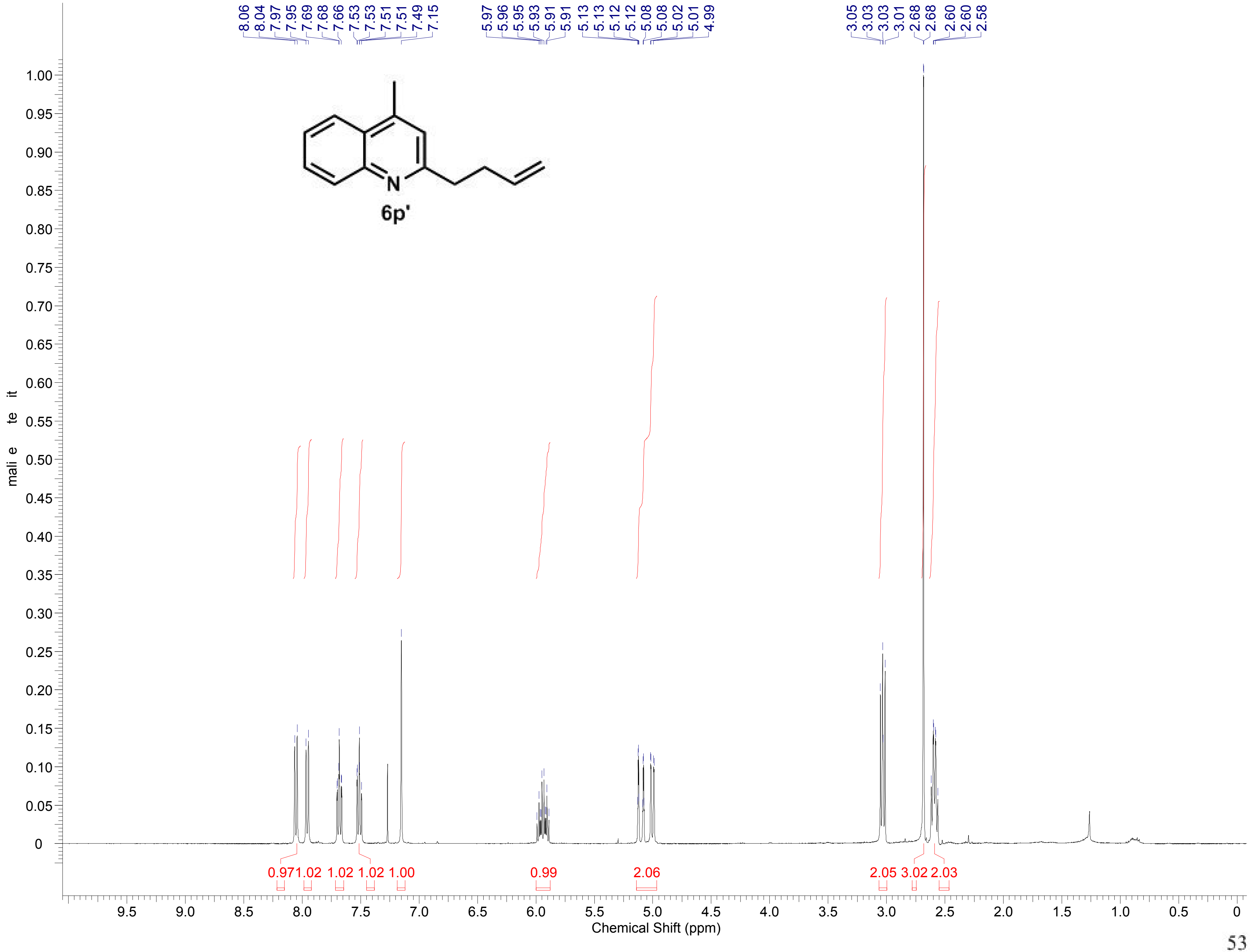
8.06 8.04 7.97 7.95 7.69 7.68 7.66 7.53 7.53 7.51 7.51 7.49 7.15
5.97 5.96 5.95 5.93 5.91 5.91 5.13 5.13 5.12 5.12 5.08 5.08 5.02 5.01 4.99
3.05 3.03 3.03 3.01 2.68 2.68 2.60 2.60 2.58
N
6p'
0.97 1.02 1.02 1.02 1.00
0.99
2.06
2.05 3.02 2.03
Chemical Shift (ppm)

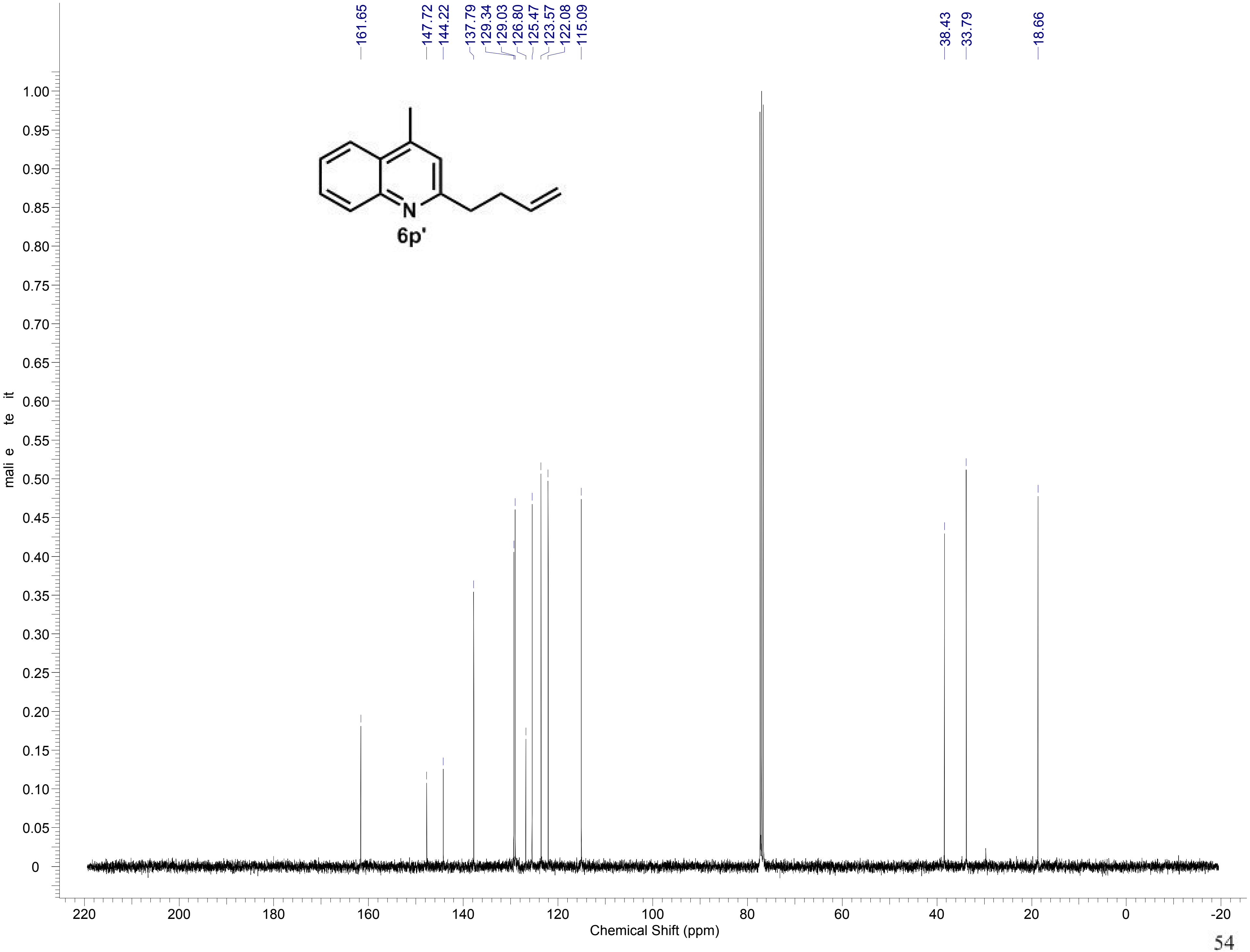
161.65
147.72
144.22
137.79
129.34
129.03
126.80
125.47
123.57
122.08
115.09
38.43
33.79
18.66
N
6p'
Chemical Shift (ppm)

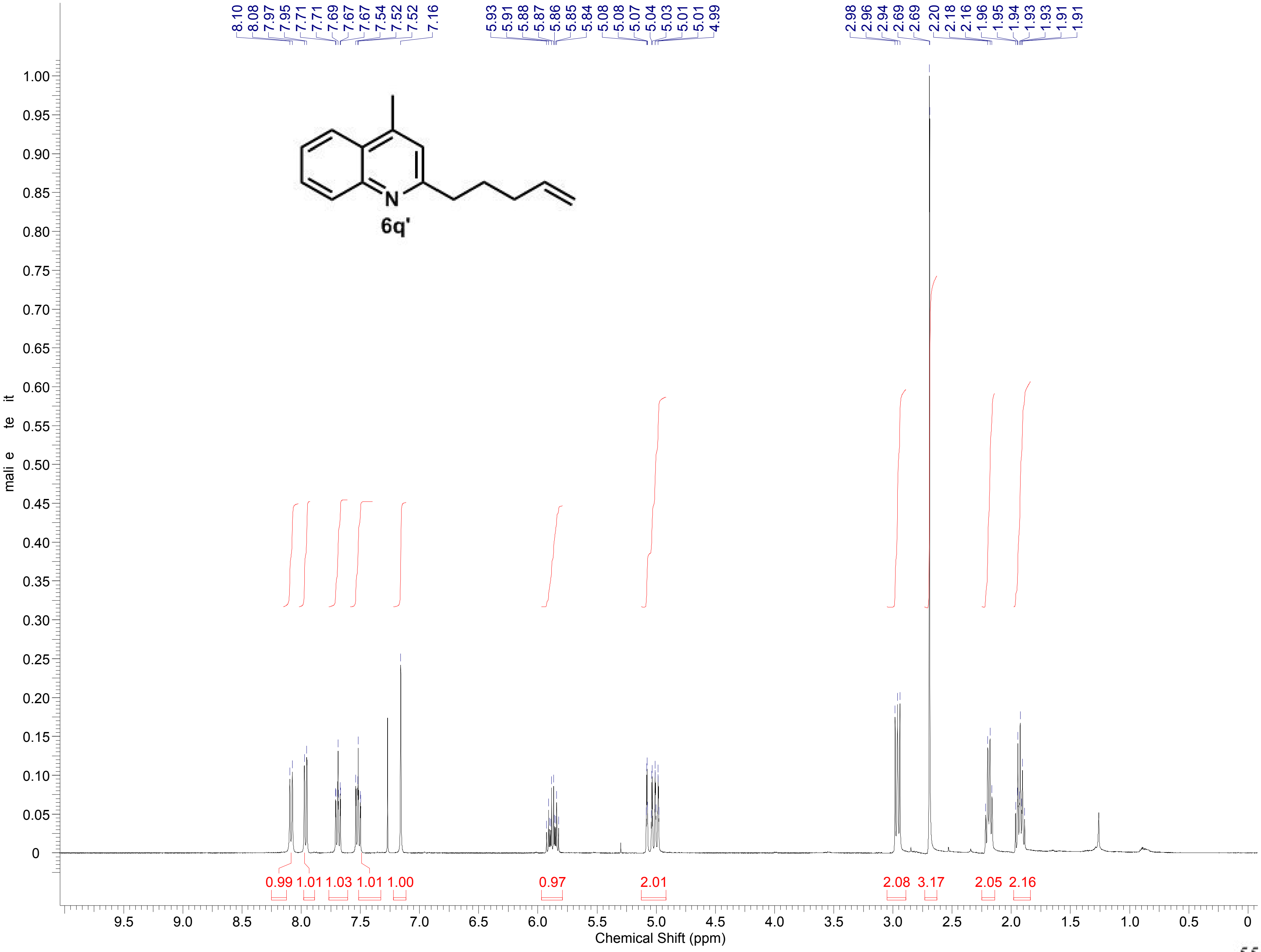
8.10 8.08 7.97 7.95 7.71 7.71 7.69 7.67 7.67 7.54 7.52 7.52 7.16
5.93 5.91 5.88 5.87 5.86 5.85 5.84 5.08 5.08 5.07 5.04 5.03 5.01 5.01 4.99
2.98 2.96 2.94 2.69 2.69 2.20 2.18 2.16 1.96 1.95 1.94 1.93 1.93 1.91 1.91
N
6q'
0.99 1.01 1.03 1.01 1.00 0.97 2.01 2.08 3.17 2.05 2.16
Chemical Shift (ppm)

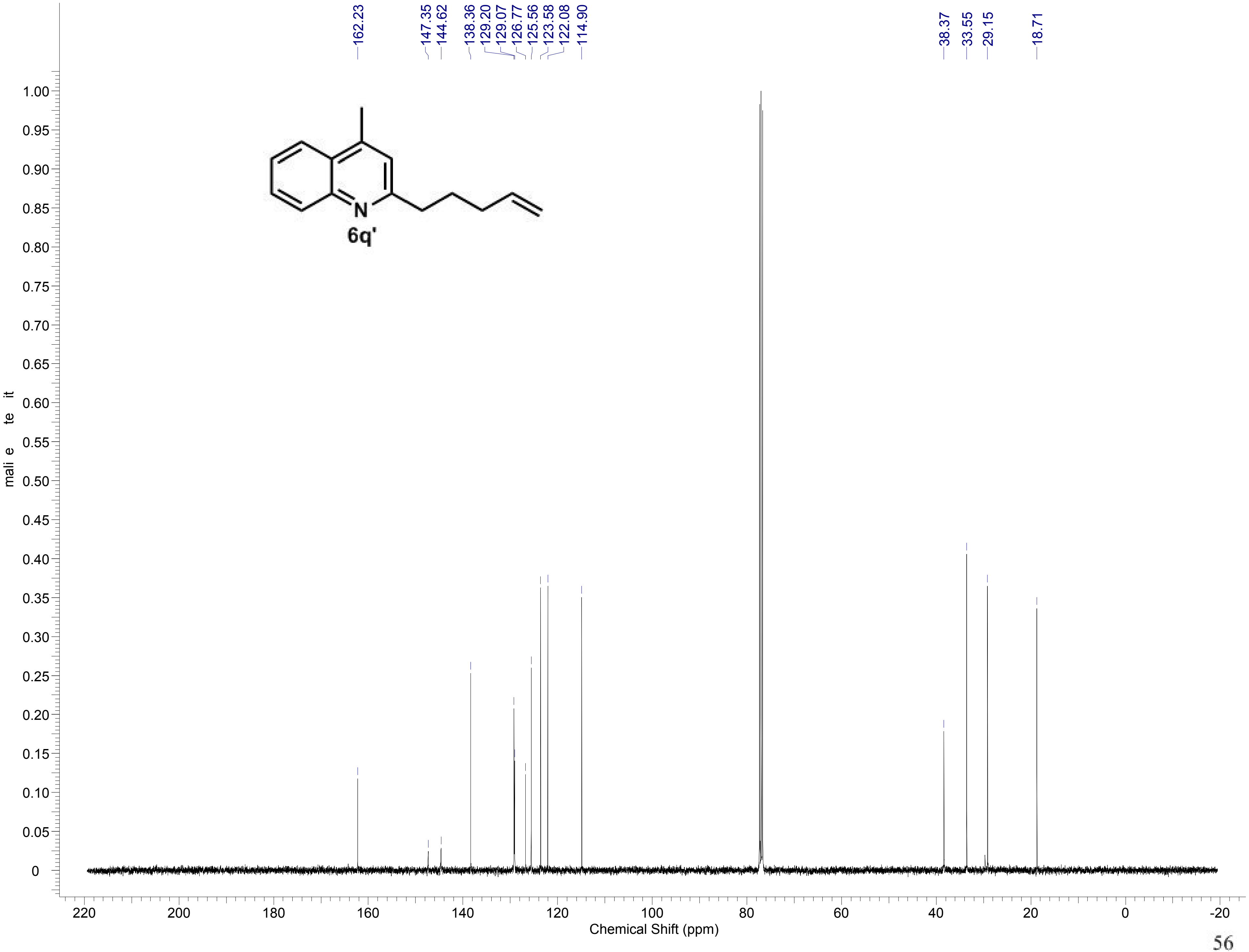
162.23
147.35
144.62
138.36
129.20
129.07
126.77
125.56
123.58
122.08
114.90
38.37
33.55
29.15
18.71
N
6q'
Chemical Shift (ppm)

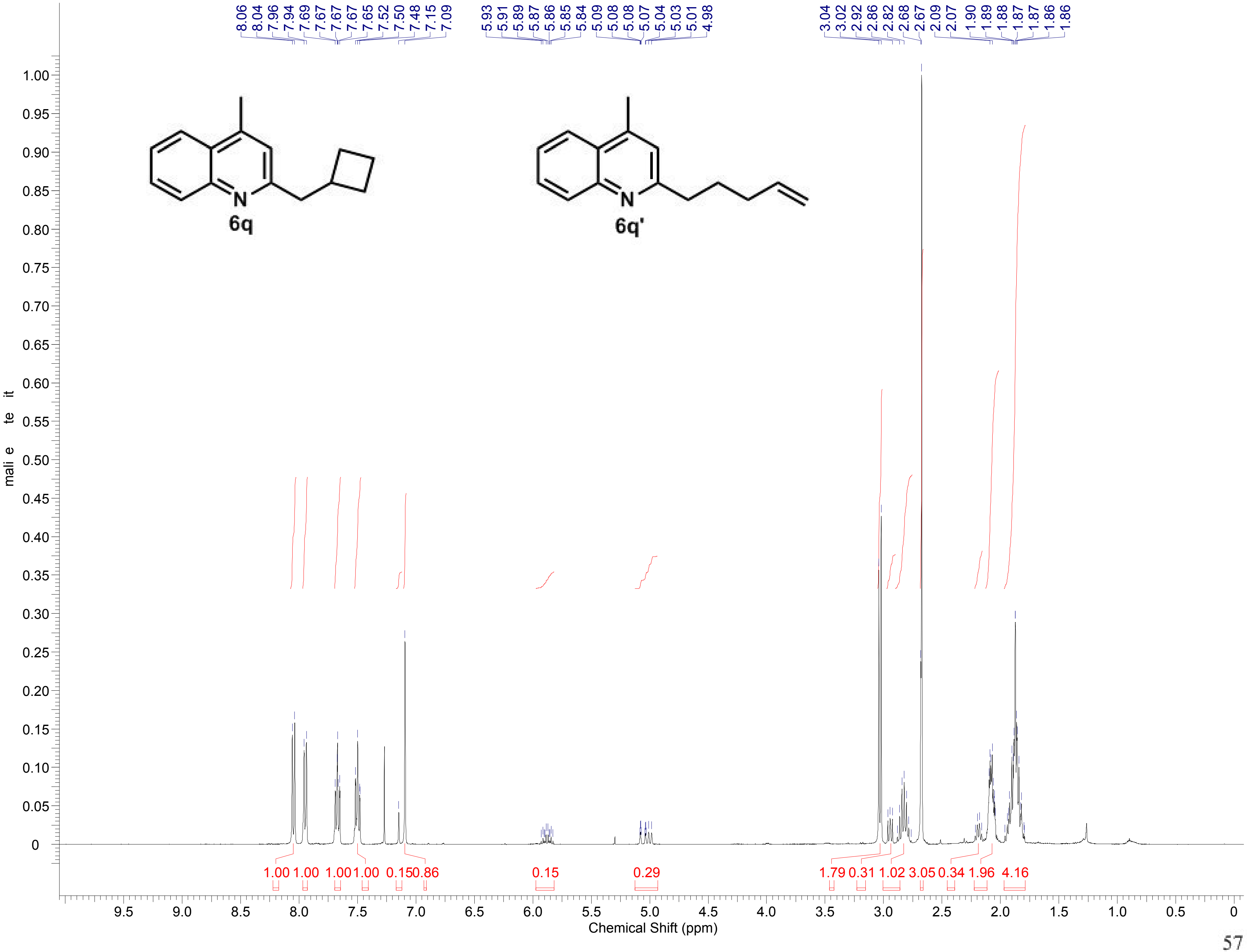

8.06 8.04 7.96 7.94 7.69 7.67 7.67 7.67 7.65 7.52 7.50 7.48 7.15 7.09
5.93 5.91 5.89 5.87 5.86 5.85 5.84 5.09 5.08 5.08 5.07 5.04 5.03 5.01 4.98
3.04 3.02 2.92 2.86 2.82 2.68 2.67 2.09 2.07 1.90 1.89 1.88 1.87 1.87 1.86 1.86
6q
6q'
1.00 1.00 1.00 1.00 0.15 0.86 0.15 0.29 1.79 0.31 1.02 3.05 0.34 1.96 4.16
Chemical Shift (ppm)

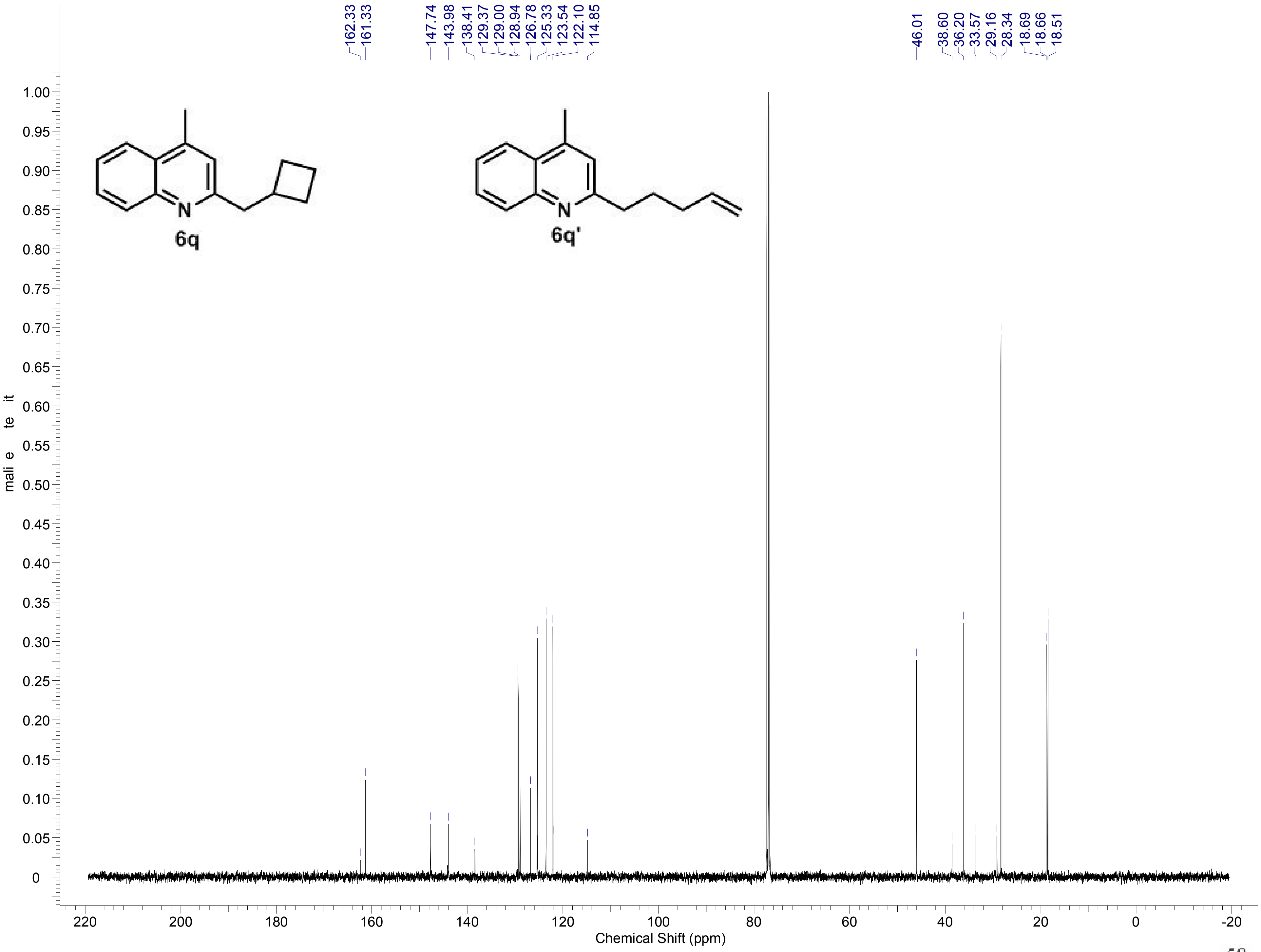

162.33
161.33
147.74
143.98
138.41
129.37
129.00
128.94
126.78
125.33
123.54
122.10
114.85
46.01
38.60
36.20
33.57
29.16
28.34
18.69
18.66
18.51
6q
6q'
N
Chemical Shift (ppm)

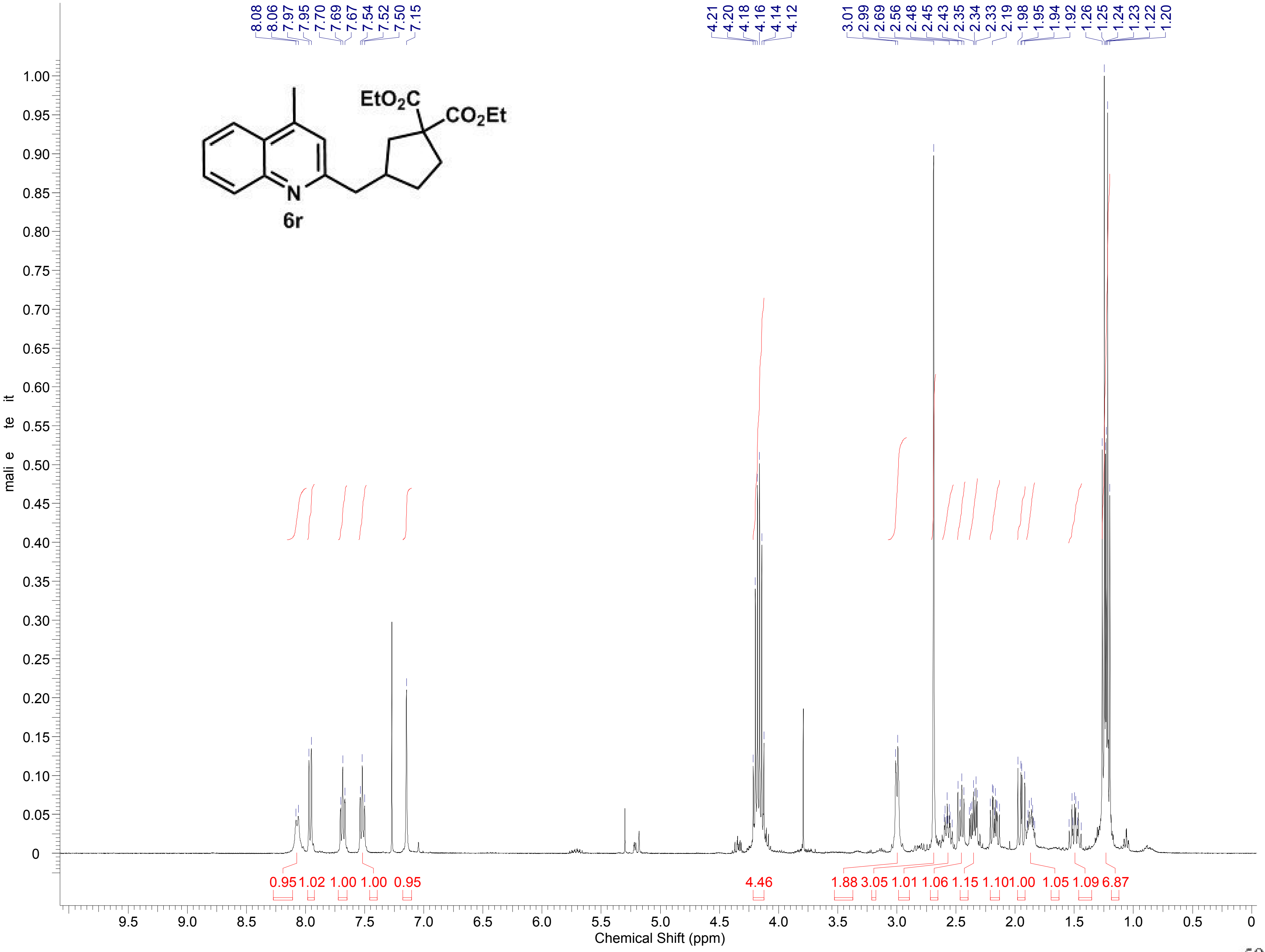

EtO2C
CO2Et
N
6r
Chemical Shift (ppm)

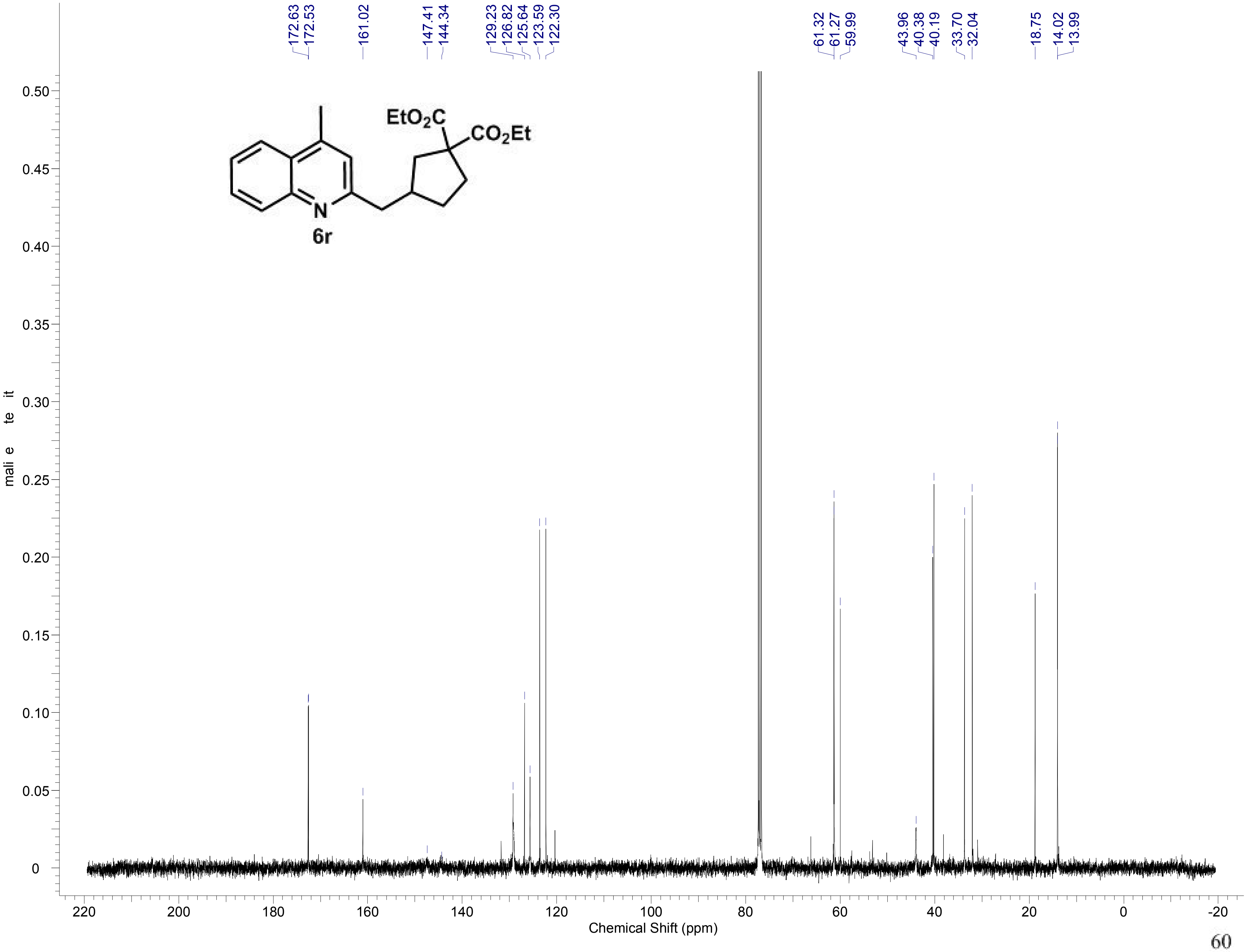
172.63
172.53
161.02
147.41
144.34
129.23
126.82
125.64
123.59
122.30
61.32
61.27
59.99
43.96
40.38
40.19
33.70
32.04
18.75
14.02
13.99
EtO2C
CO2Et
N
6r
0.50
0.45
0.40
0.35
0.30
0.25
0.20
0.15
0.10
0.05
0
mali e te it
220
200
180
160
140
120
100
80
60
40
20
0
-20
Chemical Shift (ppm)

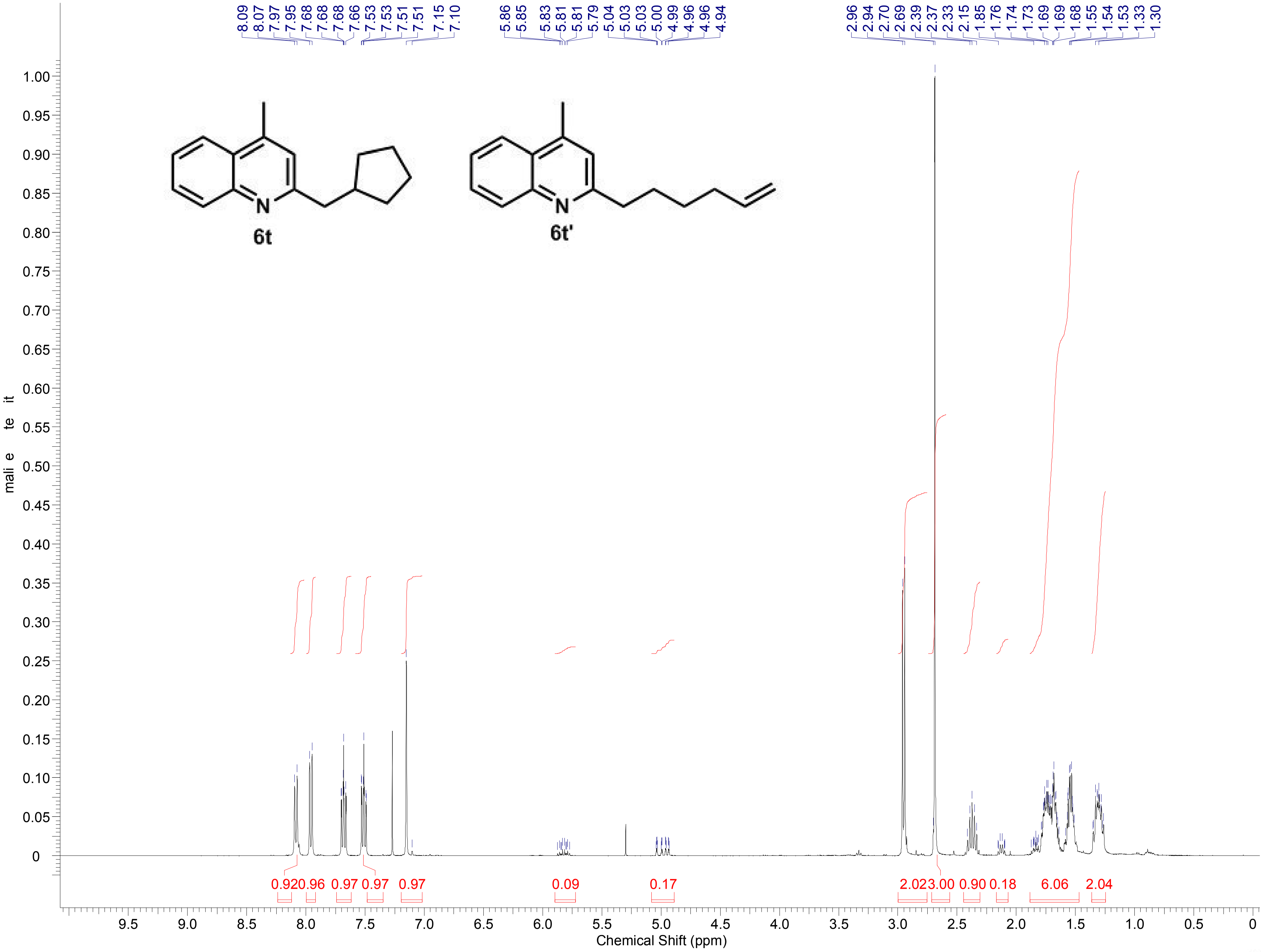
8.09
8.07
7.97
7.95
7.68
7.68
7.68
7.66
7.53
7.53
7.51
7.51
7.15
7.10
5.86
5.85
5.83
5.81
5.81
5.79
5.04
5.03
5.03
5.00
4.99
4.96
4.96
4.94
2.96
2.94
2.70
2.69
2.39
2.37
2.33
2.15
1.85
1.76
1.74
1.73
1.69
1.69
1.68
1.55
1.54
1.53
1.33
1.30
N
6t
N
6t'
mali e te it
0.92
0.96
0.97
0.97
0.97
0.09
0.17
2.02
3.00
0.90
0.18
6.06
2.04
Chemical Shift (ppm)

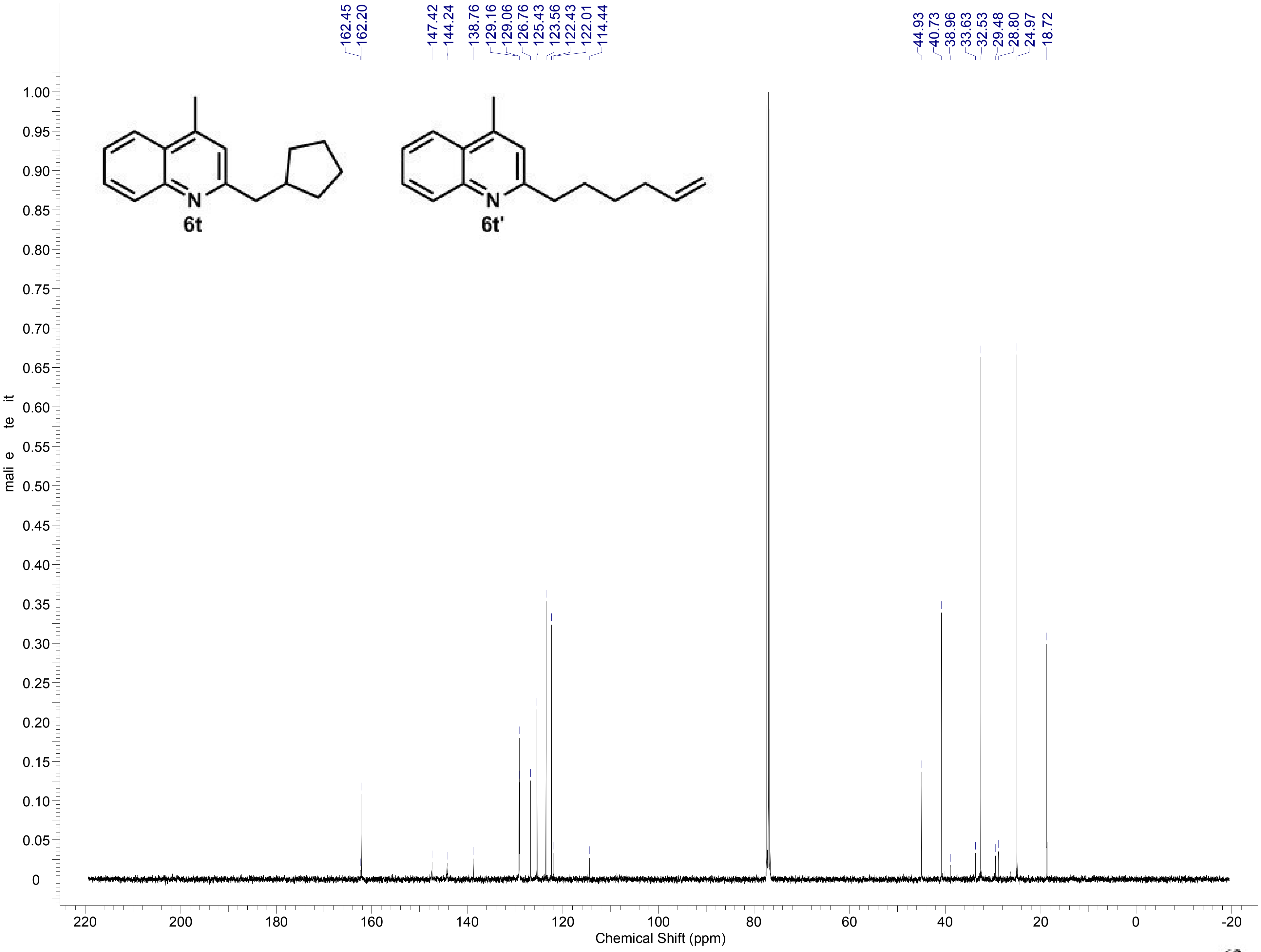
162.45
162.20
147.42
144.24
138.76
129.16
129.06
126.76
125.43
123.56
122.43
122.01
114.44
44.93
40.73
38.96
33.63
32.53
29.48
28.80
24.97
18.72
6t
6t'
Chemical Shift (ppm)

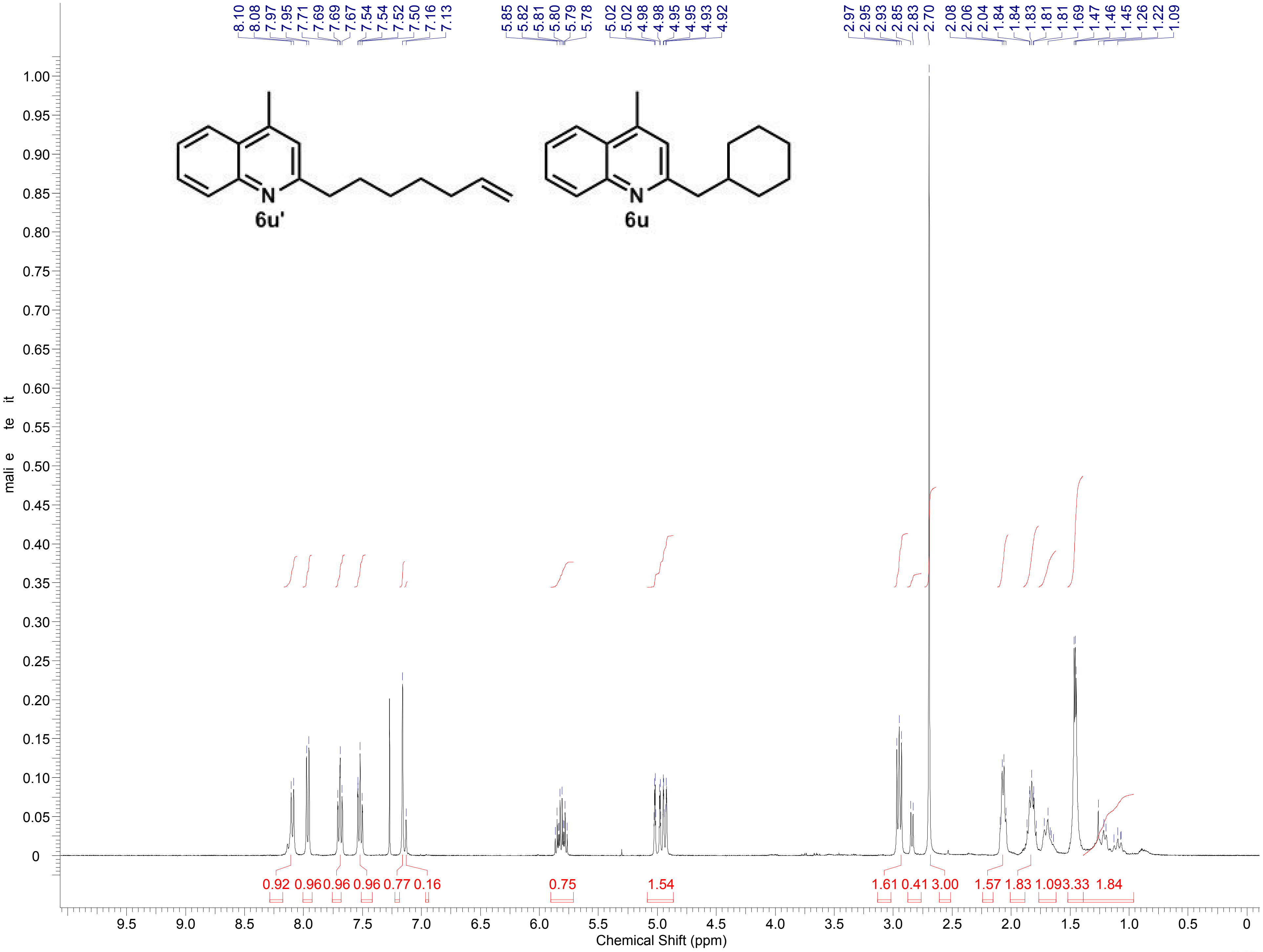

8.10
8.08
7.97
7.95
7.71
7.69
7.69
7.67
7.54
7.54
7.52
7.50
7.16
7.13
5.85
5.82
5.81
5.80
5.79
5.78
5.02
5.02
4.98
4.98
4.95
4.95
4.93
4.92
2.97
2.95
2.93
2.85
2.83
2.70
2.08
2.06
2.04
1.84
1.84
1.83
1.81
1.81
1.69
1.47
1.46
1.45
1.26
1.22
1.09
6u'
6u
N
0.92
0.96
0.96
0.96
0.77
0.16
0.75
1.54
1.61
0.41
3.00
1.57
1.83
1.09
3.33
1.84
Chemical Shift (ppm)

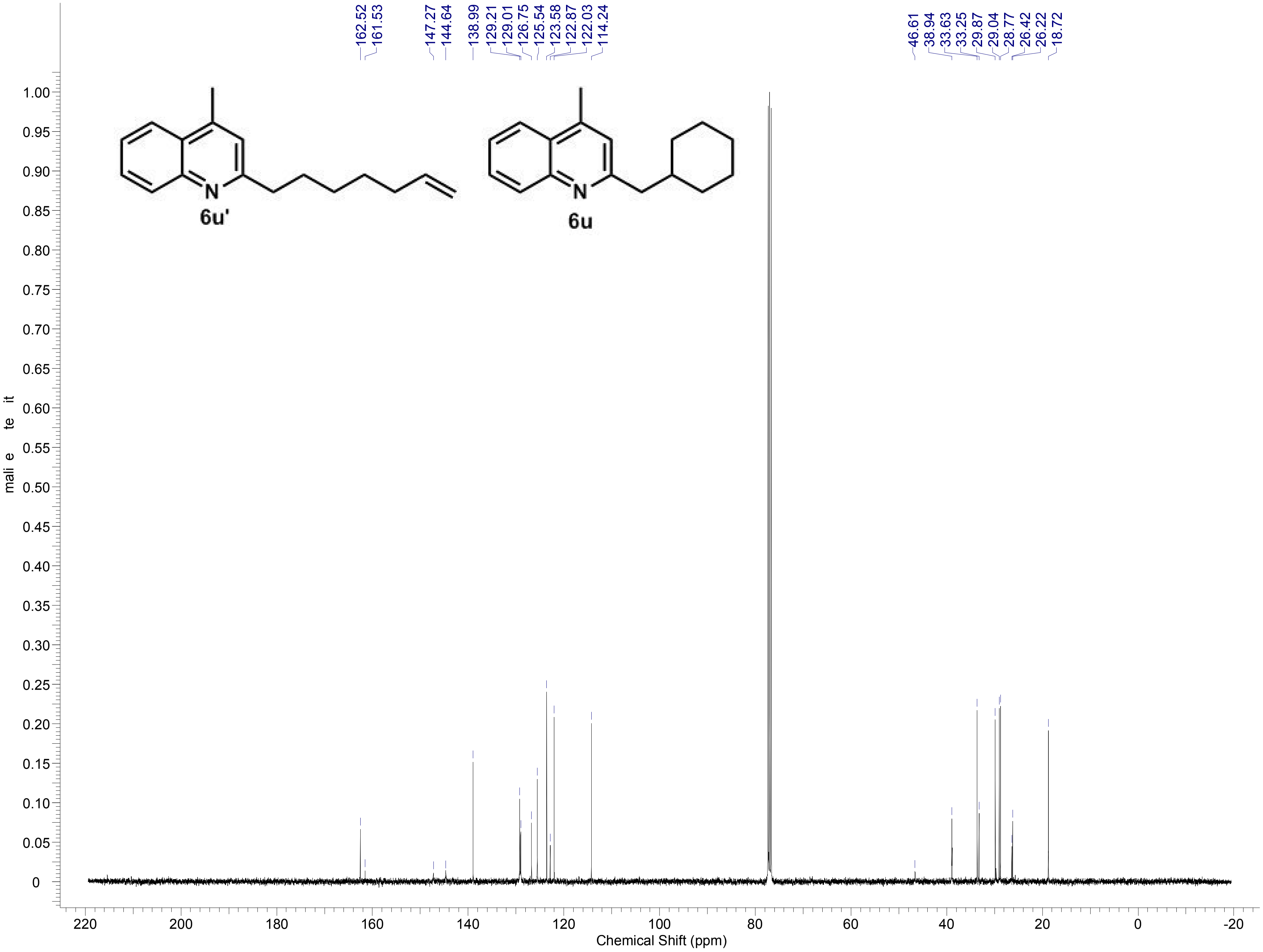

162.52
161.53
147.27
144.64
138.99
129.21
129.01
126.75
125.54
123.58
122.87
122.03
114.24
46.61
38.94
33.63
33.25
29.87
29.04
28.77
26.42
26.22
18.72
N
6u'
N
6u
mali e te it
Chemical Shift (ppm)

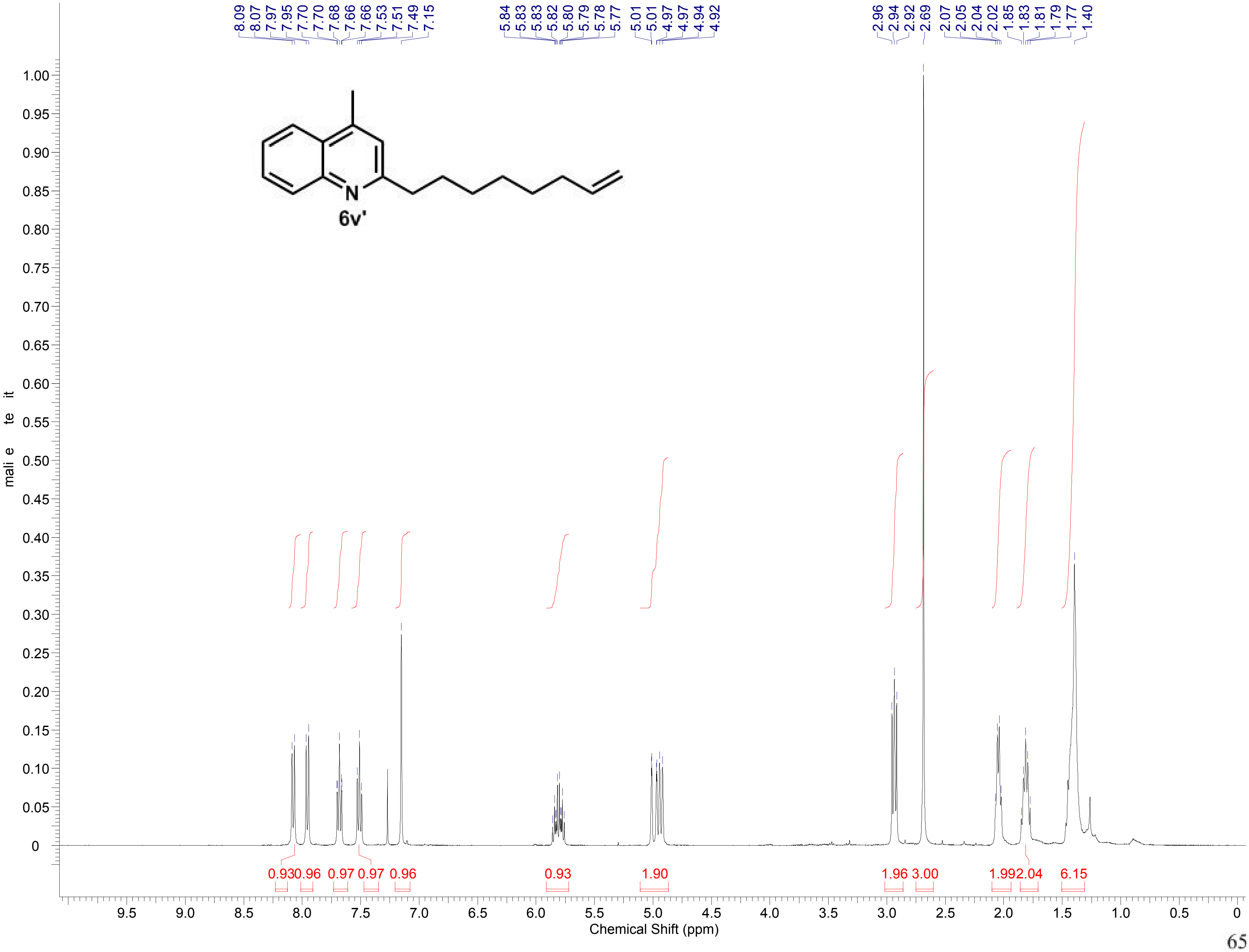
8.09
8.07
7.97
7.95
7.70
7.68
7.66
7.66
7.53
7.51
7.49
7.15
5.84
5.83
5.83
5.82
5.80
5.79
5.78
5.77
5.01
5.01
4.97
4.97
4.94
4.92
2.96
2.94
2.92
2.69
2.07
2.05
2.04
2.02
1.85
1.83
1.81
1.79
1.77
1.40
N
6v'
0.93
0.96
0.97
0.97
0.96
0.93
1.90
1.96
3.00
1.99
2.04
6.15
Chemical Shift (ppm)

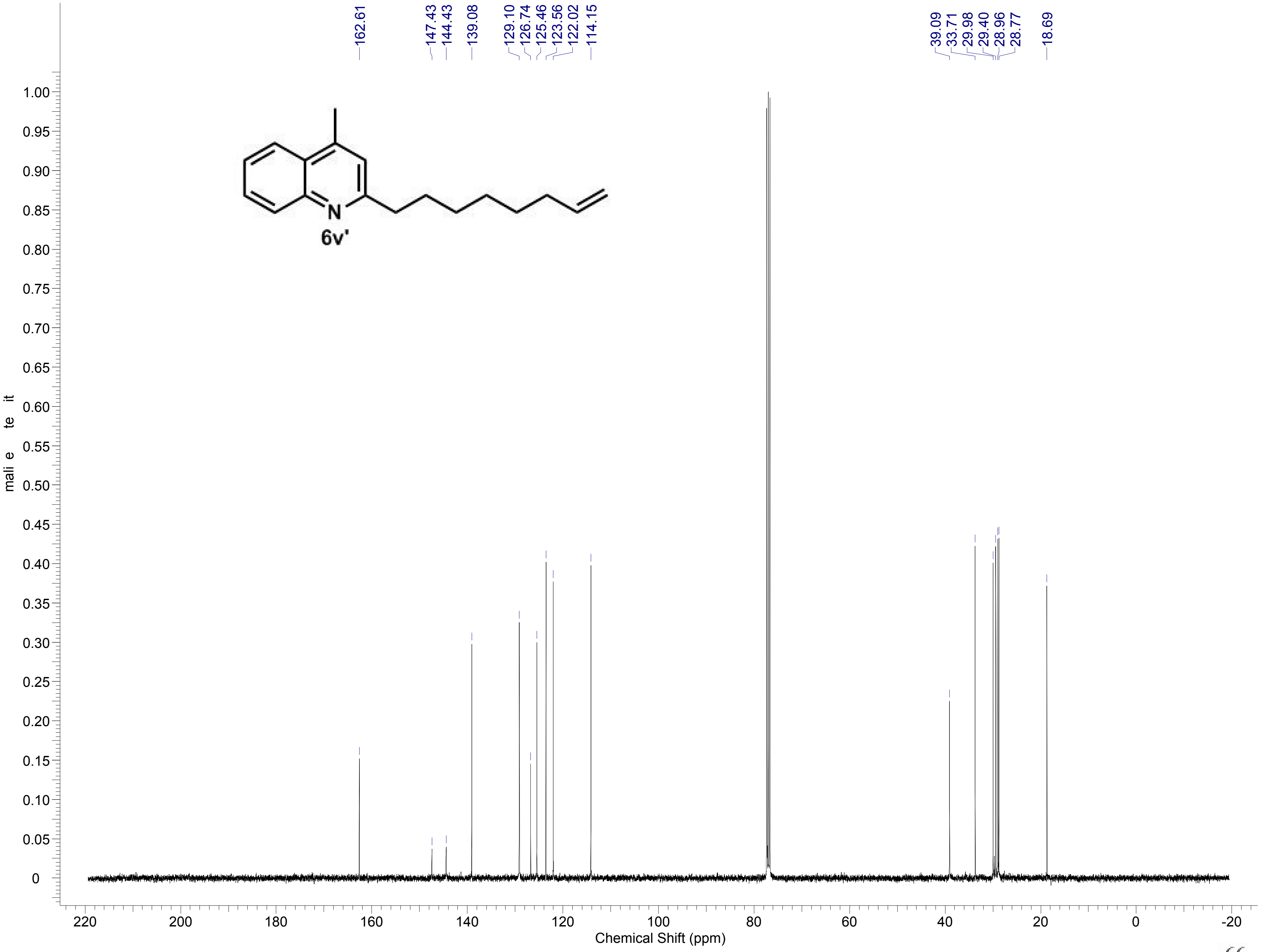
162.61
147.43
144.43
139.08
129.10
126.74
125.46
123.56
122.02
114.15
39.09
33.71
29.98
29.40
28.96
28.77
18.69
N
6v'
Chemical Shift (ppm)

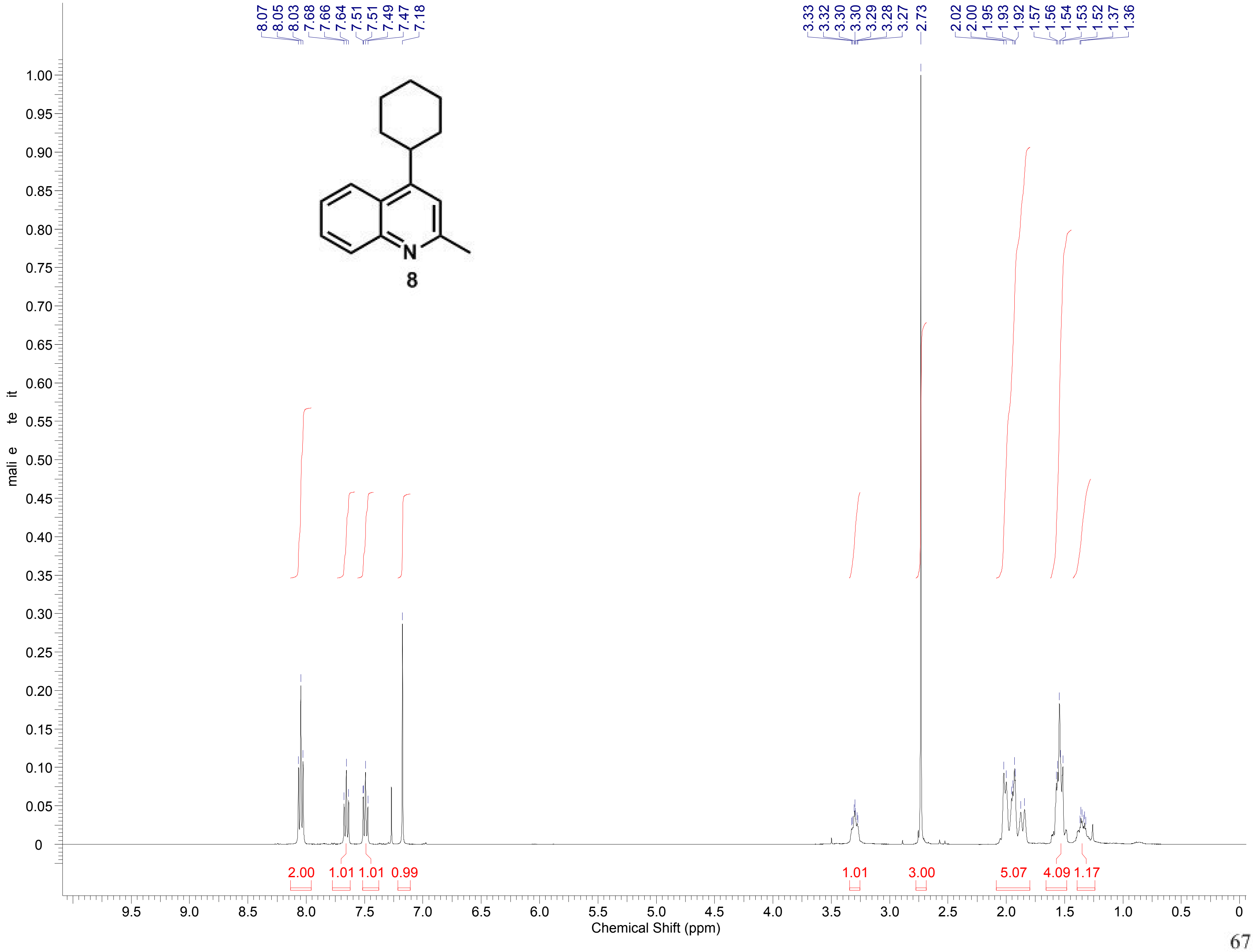
8.07
8.05
8.03
7.68
7.66
7.64
7.51
7.51
7.49
7.47
7.18
3.33
3.32
3.30
3.30
3.29
3.28
3.27
2.73
2.02
2.00
1.95
1.93
1.92
1.57
1.56
1.54
1.53
1.52
1.37
1.36
N
8
2.00
1.01
1.01
0.99
1.01
3.00
5.07
4.09
1.17
Chemical Shift (ppm)

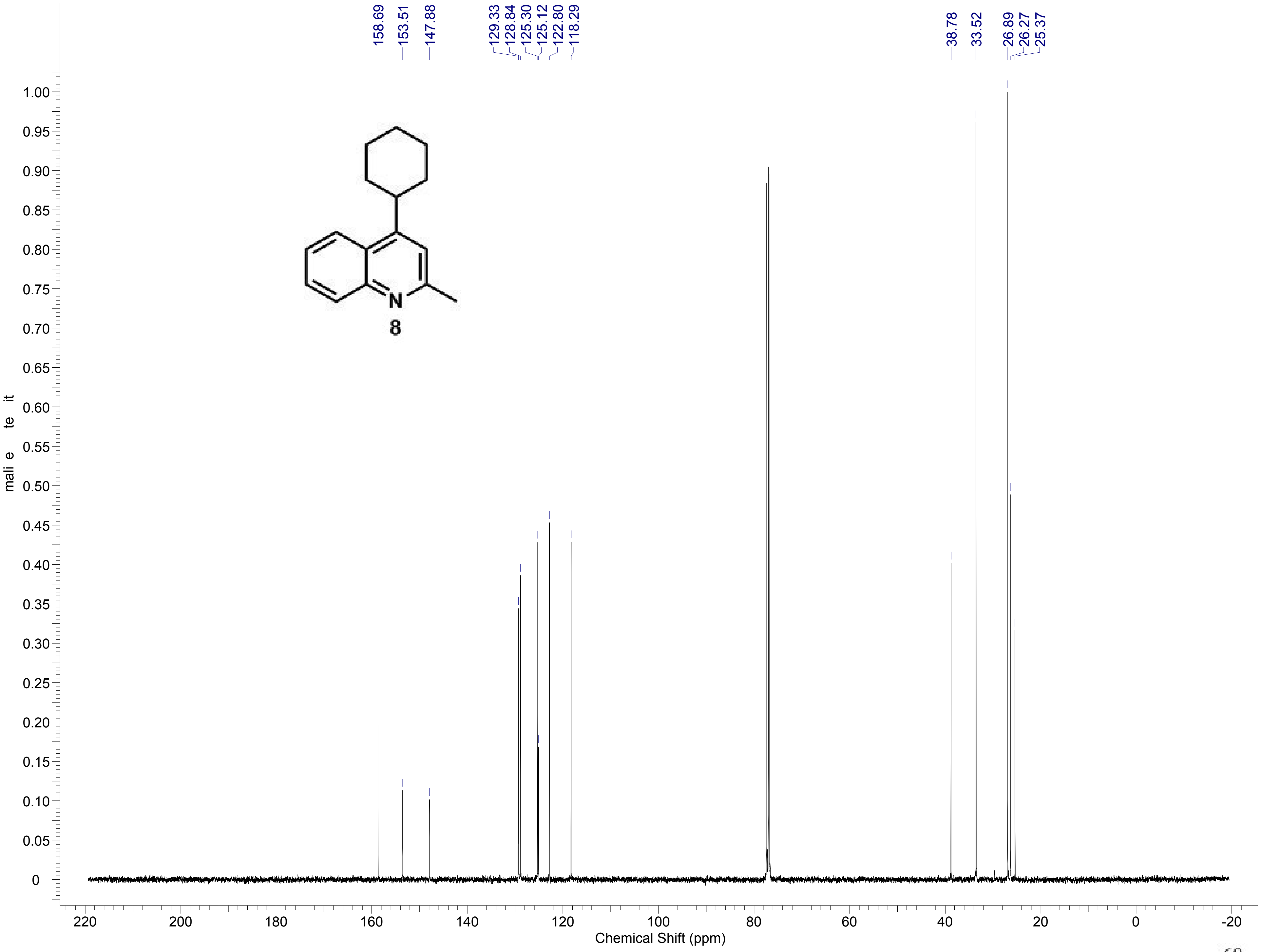

158.69
153.51
147.88
129.33
128.84
125.30
125.12
122.80
118.29
38.78
33.52
26.89
26.27
25.37
N
8
Chemical Shift (ppm)

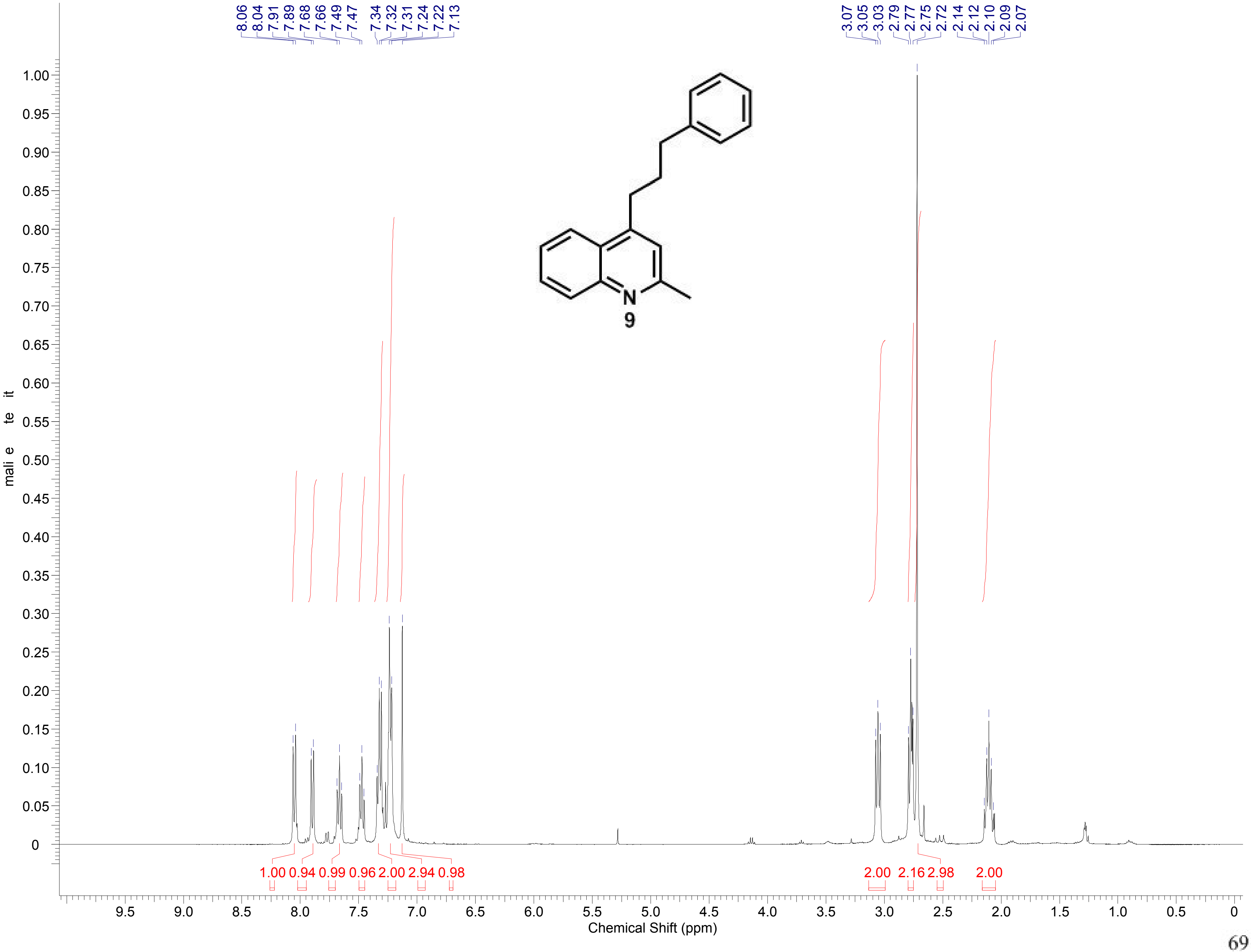

8.06 8.04 7.91 7.89 7.68 7.66 7.49 7.47 7.34 7.32 7.31 7.24 7.22 7.13
3.07 3.05 3.03 2.79 2.77 2.75 2.72 2.14 2.12 2.10 2.09 2.07
N
9
1.00 0.94 0.99 0.96 2.00 2.94 0.98
2.00 2.16 2.98 2.00
Chemical Shift (ppm)

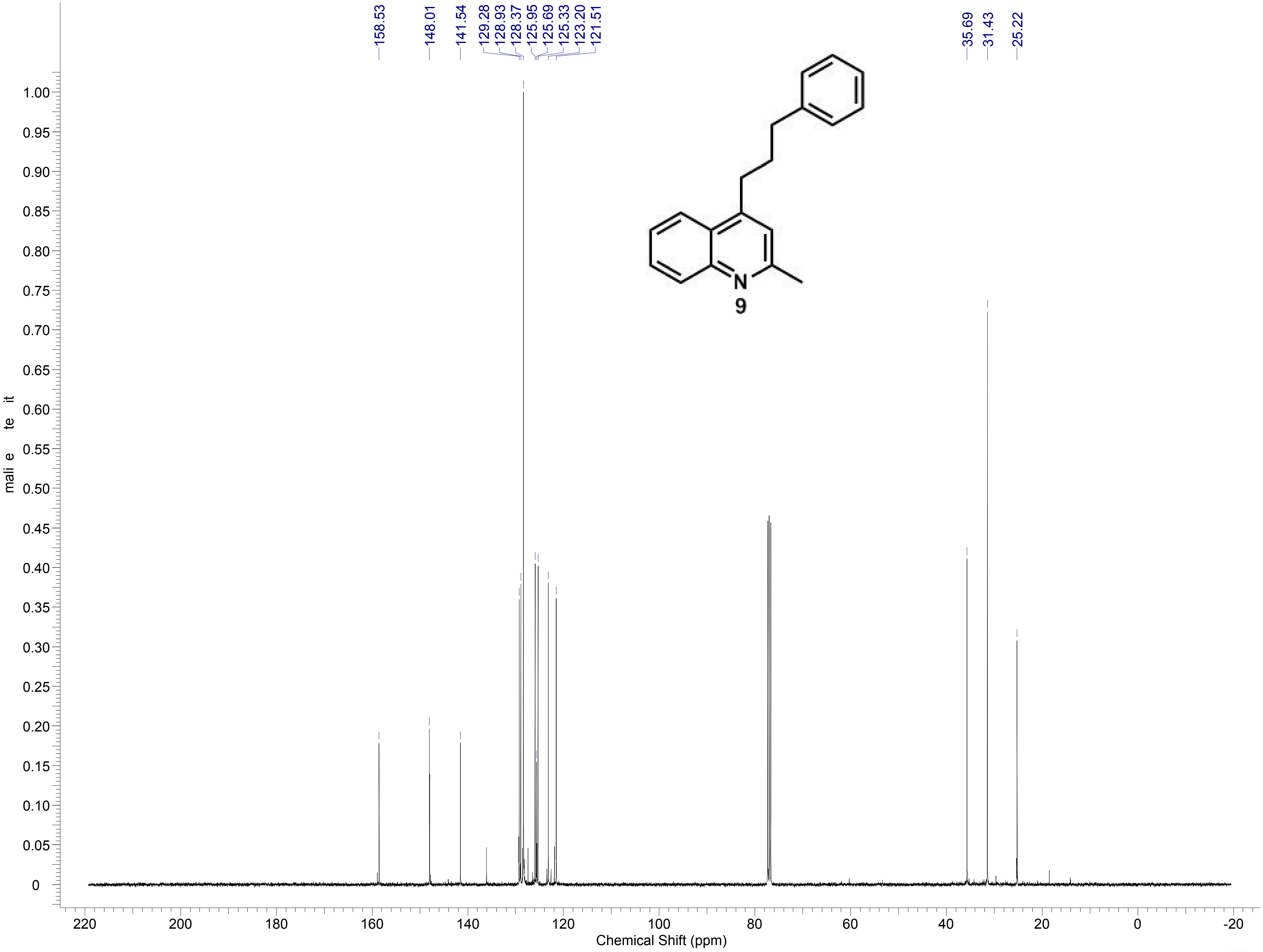

158.53
148.01
141.54
129.28
128.93
128.37
125.95
125.69
125.33
123.20
121.51
35.69
31.43
25.22
9
N
mali e te it
1.00
0.95
0.90
0.85
0.80
0.75
0.70
0.65
0.60
0.55
0.50
0.45
0.40
0.35
0.30
0.25
0.20
0.15
0.10
0.05
0
220
200
180
160
140
120
100
80
60
40
20
0
-20
Chemical Shift (ppm)

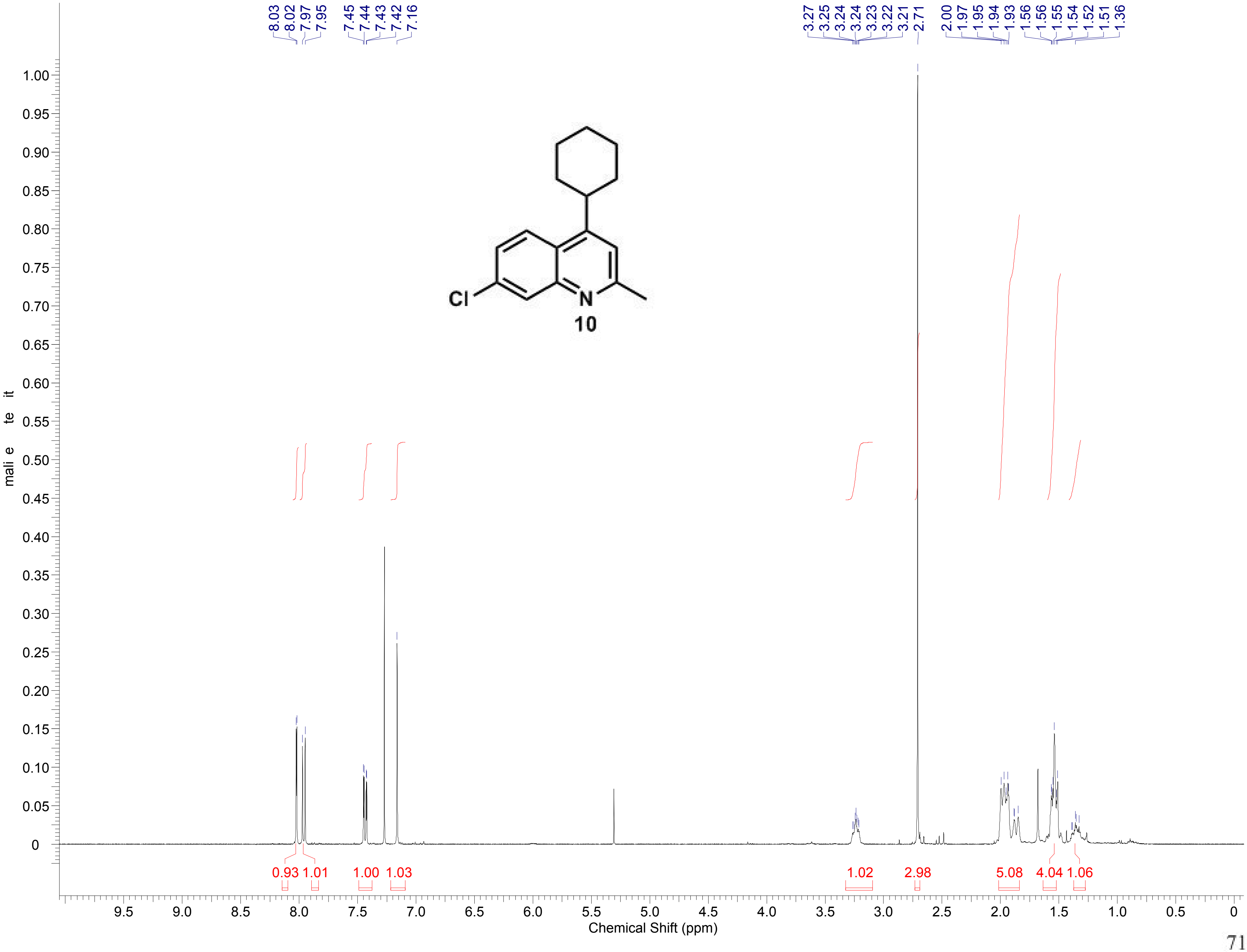
8.03
8.02
7.97
7.95
7.45
7.44
7.43
7.42
7.16
3.27
3.25
3.24
3.24
3.23
3.22
3.21
2.71
2.00
1.97
1.95
1.94
1.93
1.56
1.56
1.55
1.54
1.52
1.51
1.36
Cl
N
10
0.93
1.01
1.00
1.03
1.02
2.98
5.08
4.04
1.06
Chemical Shift (ppm)

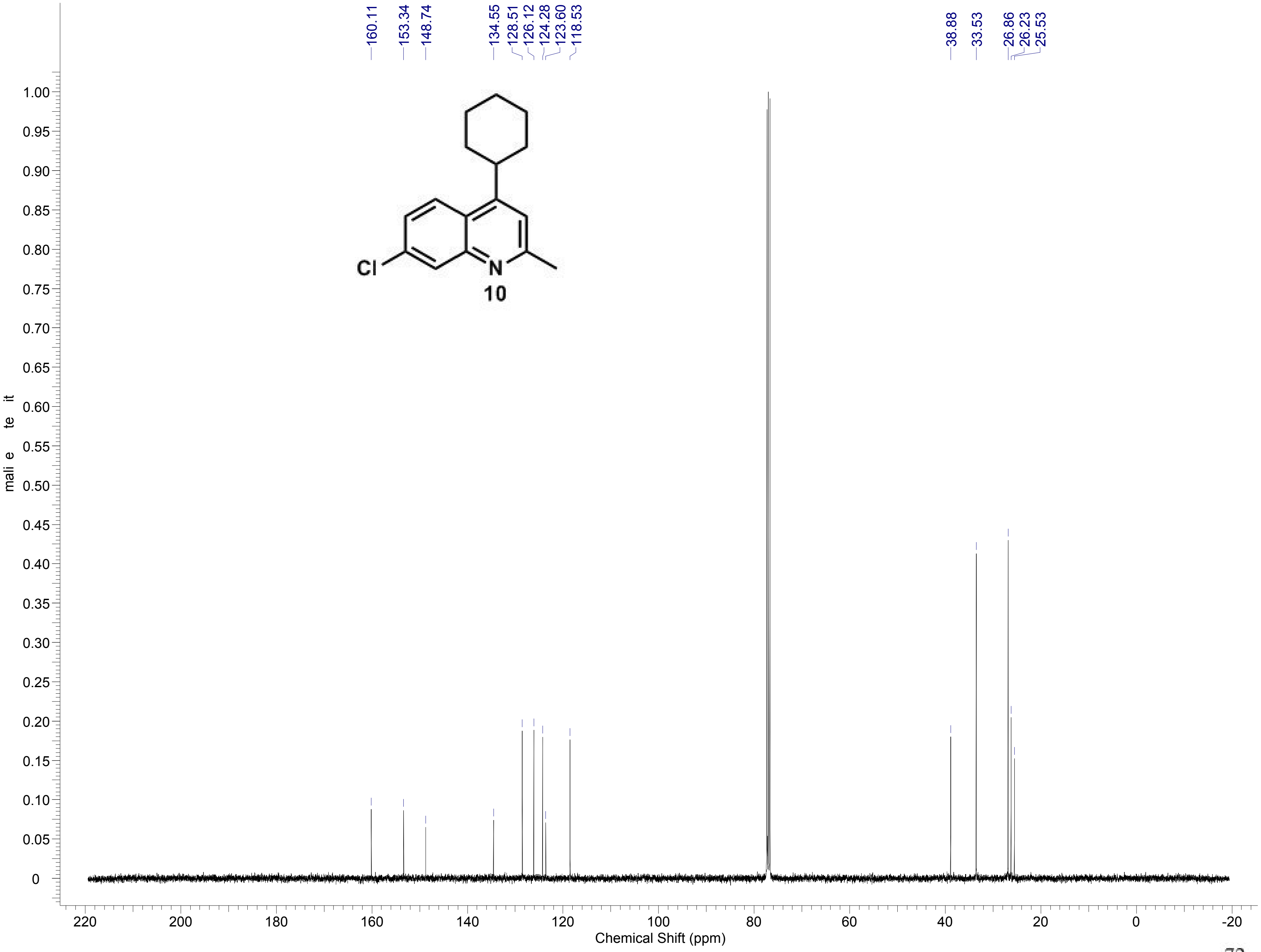
160.11
153.34
148.74
134.55
128.51
126.12
124.28
123.60
118.53
38.88
33.53
26.86
26.23
25.53
Cl
N
10
Chemical Shift (ppm)

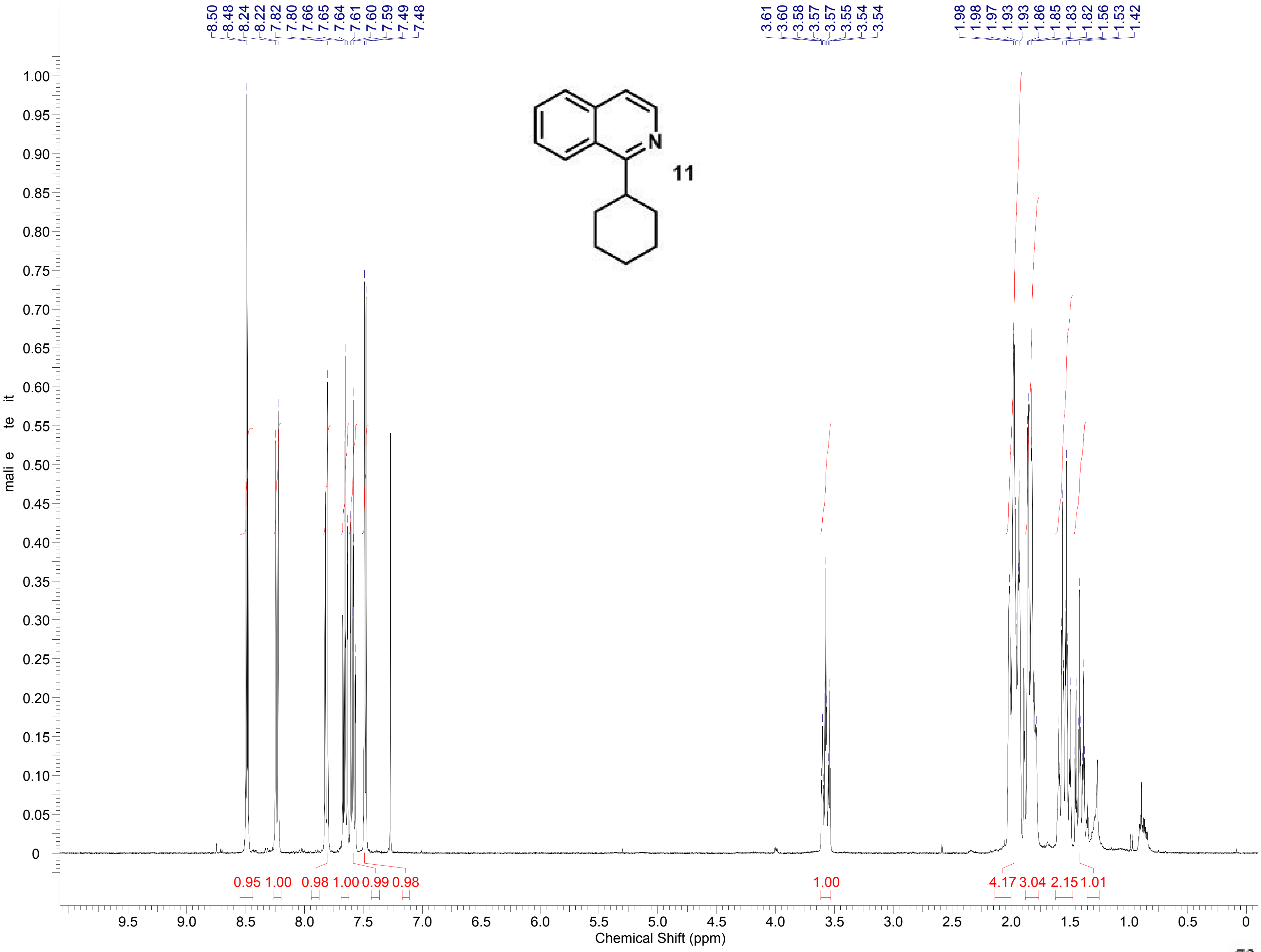

8.50
8.48
8.24
8.22
7.82
7.80
7.66
7.65
7.64
7.61
7.60
7.59
7.49
7.48
3.61
3.60
3.58
3.57
3.57
3.55
3.54
3.54
1.98
1.98
1.97
1.93
1.93
1.86
1.85
1.83
1.82
1.56
1.53
1.42
N
11
0.95 1.00 0.98 1.00 0.99 0.98
1.00
4.17 3.04 2.15 1.01
Chemical Shift (ppm)

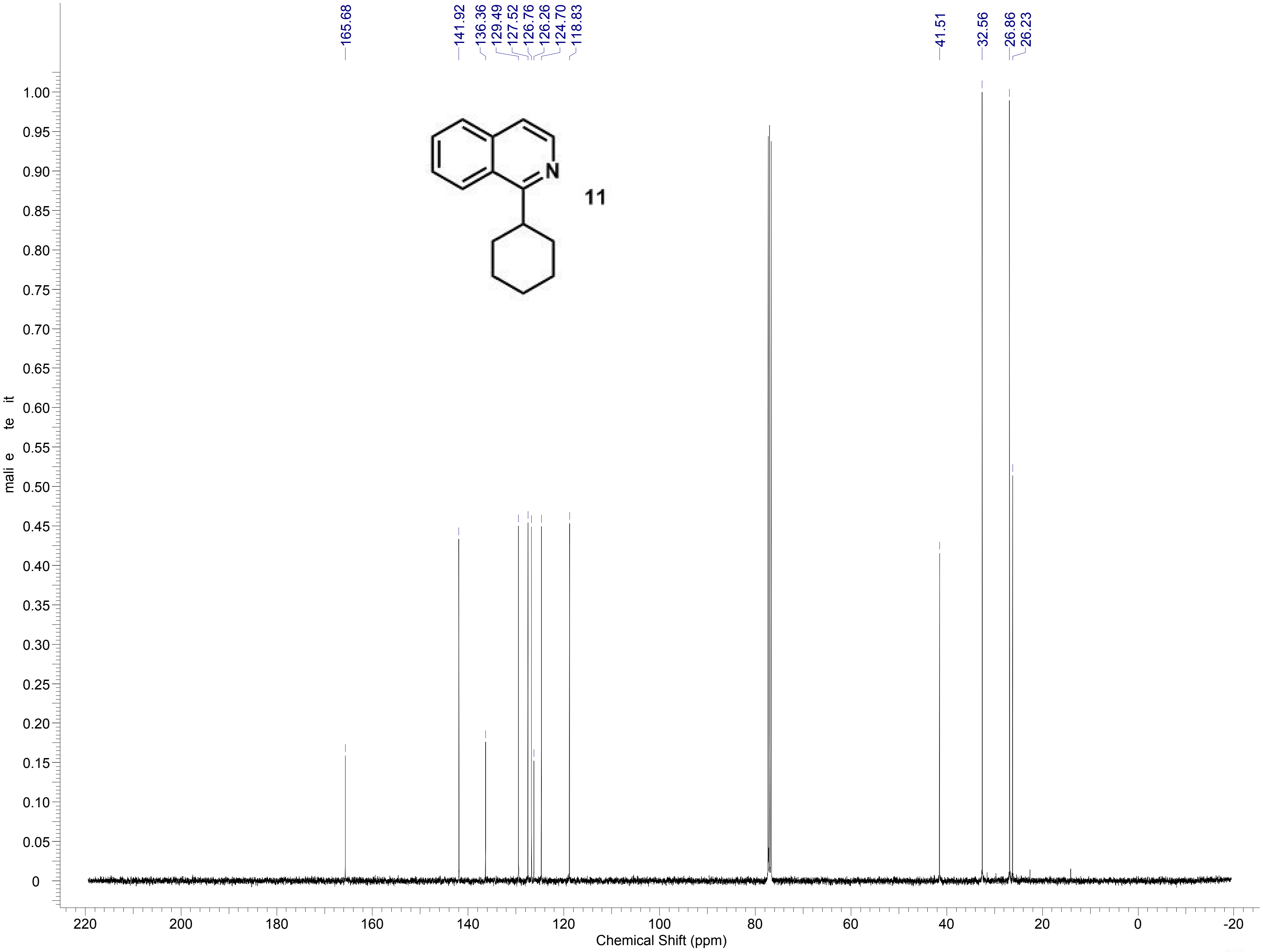

165.68
141.92
136.36
129.49
127.52
126.76
126.26
124.70
118.83
41.51
32.56
26.86
26.23
N
11
mali e te it
Chemical Shift (ppm)

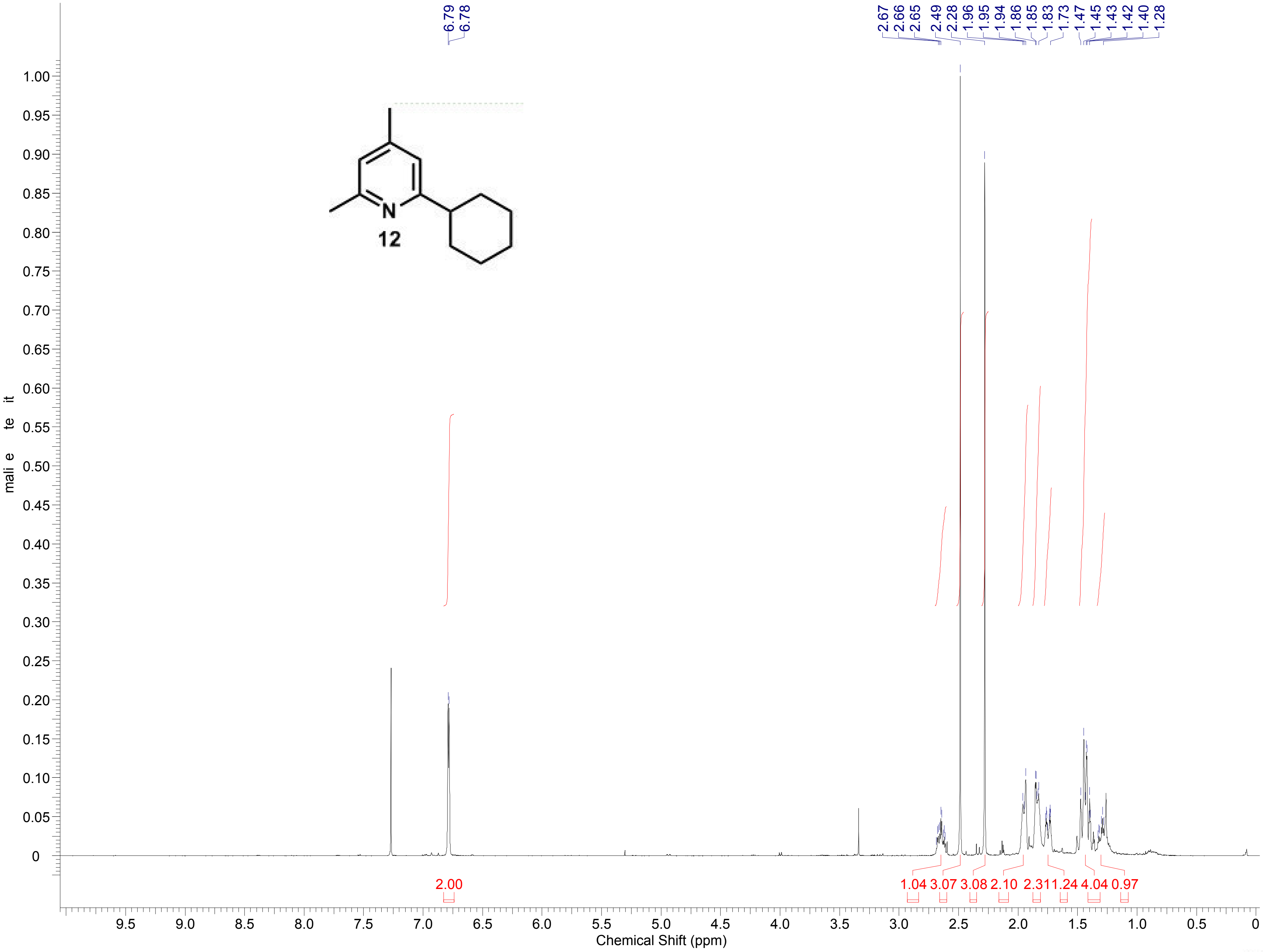

6.79
6.78
2.67
2.66
2.65
2.49
2.28
1.96
1.95
1.94
1.86
1.85
1.83
1.73
1.47
1.45
1.43
1.42
1.40
1.28
N
12
2.00
1.04 3.07 3.08 2.10 2.31 1.24 4.04 0.97
Chemical Shift (ppm)

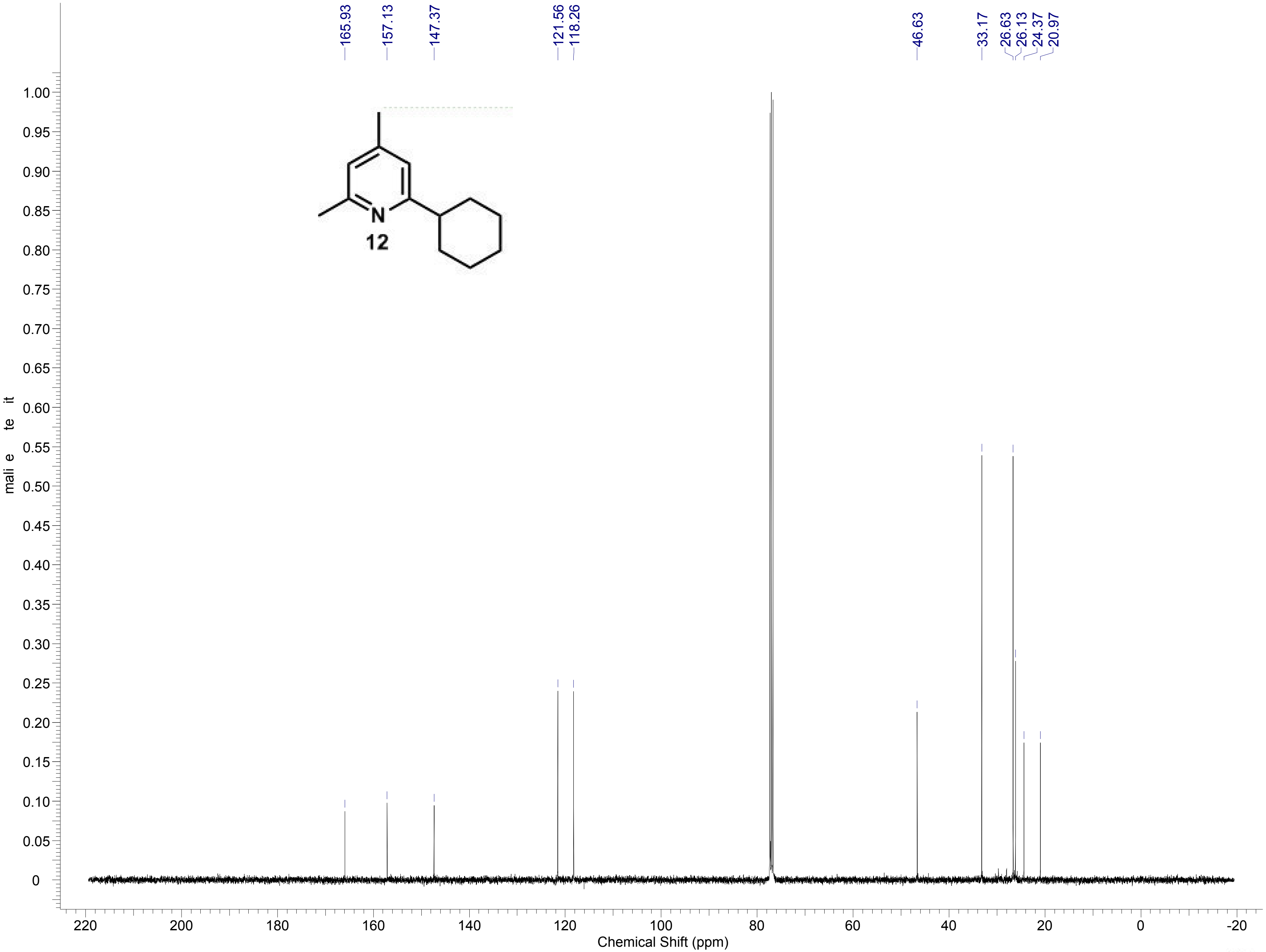
165.93
157.13
147.37
121.56
118.26
46.63
33.17
26.63
26.13
24.37
20.97
N
12
Chemical Shift (ppm)

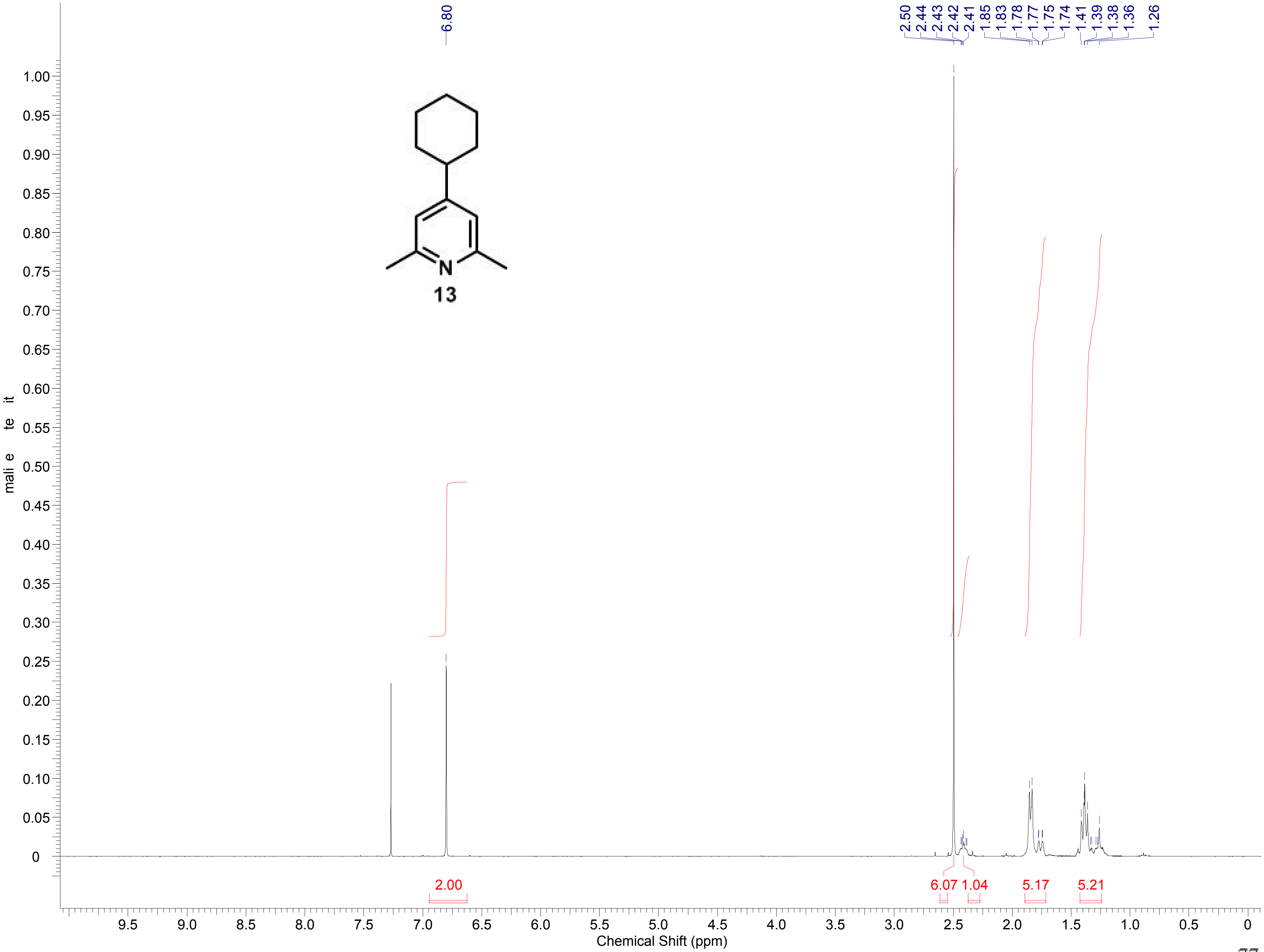
6.80
2.50 2.44 2.43 2.42 2.41 1.85 1.83 1.78 1.77 1.75 1.74 1.41 1.39 1.38 1.36 1.26
13
2.00
6.07 1.04
5.17
5.21
Chemical Shift (ppm)

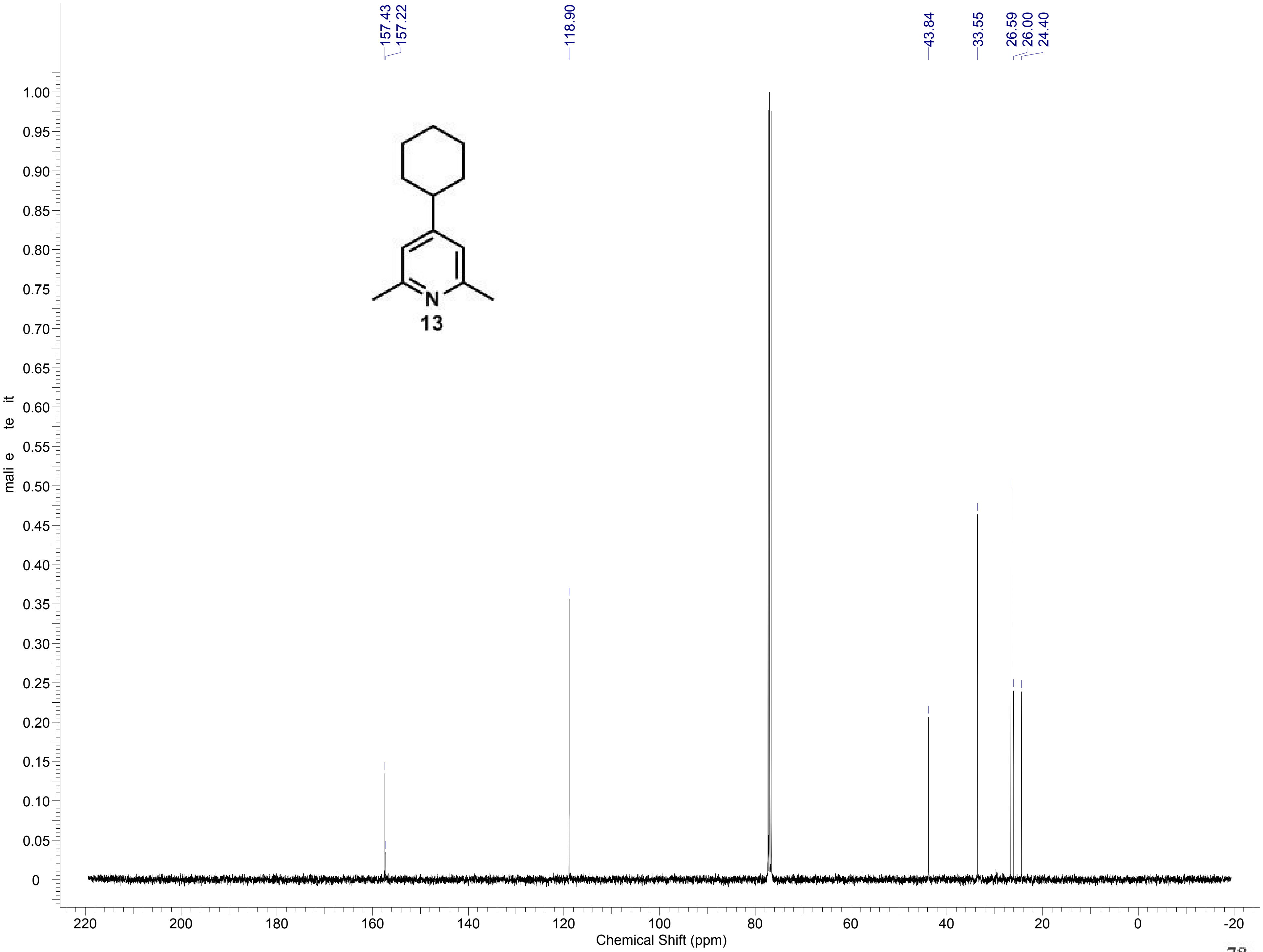

157.43
157.22
118.90
43.84
33.55
26.59
26.00
24.40
N
13
Chemical Shift (ppm)

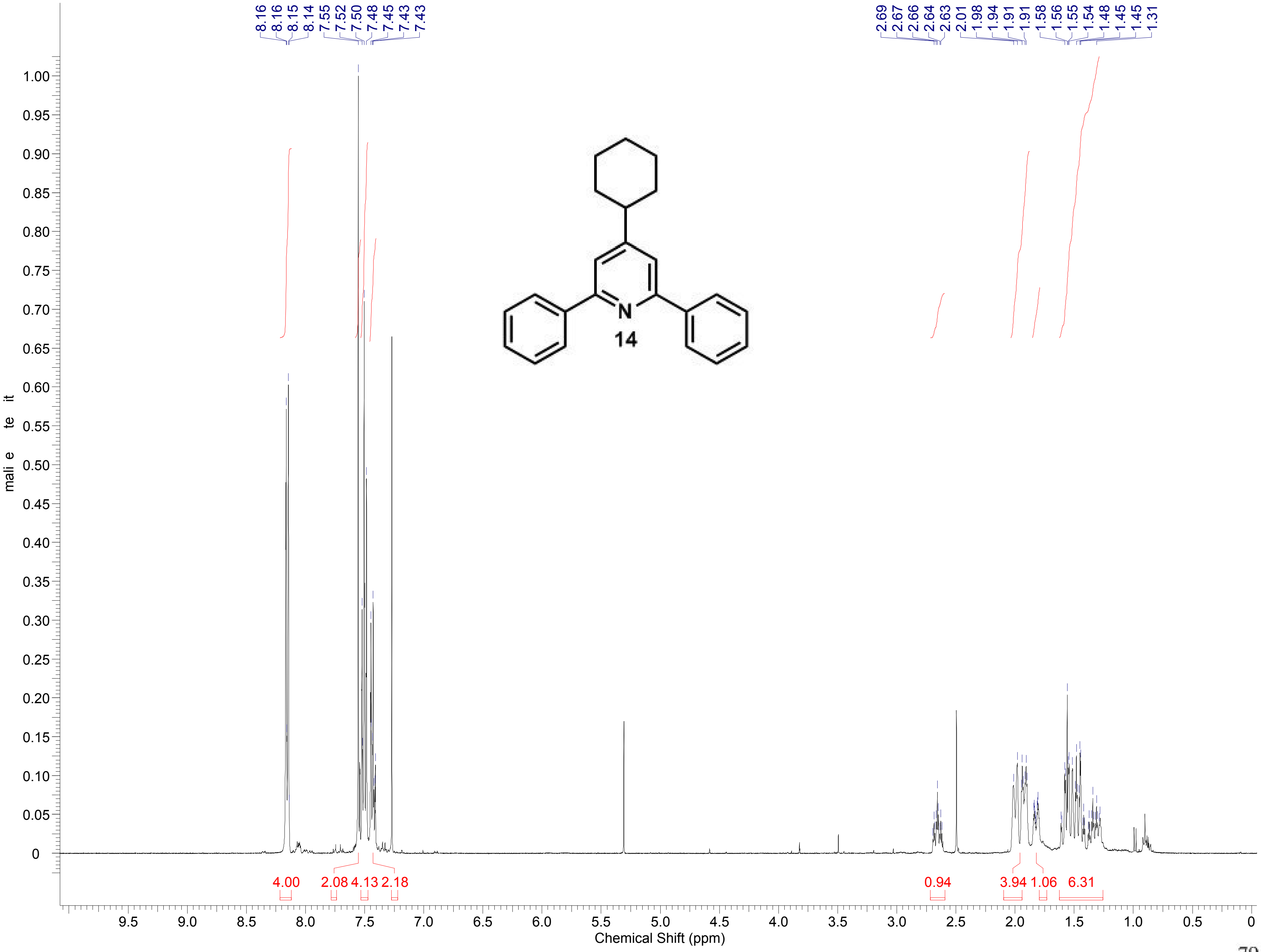
8.16
8.16
8.15
8.14
7.55
7.52
7.50
7.48
7.45
7.43
7.43
2.69
2.67
2.66
2.64
2.63
2.01
1.98
1.94
1.91
1.91
1.58
1.56
1.55
1.54
1.48
1.45
1.45
1.31
N
14
4.00
2.08
4.13
2.18
0.94
3.94
1.06
6.31
Chemical Shift (ppm)

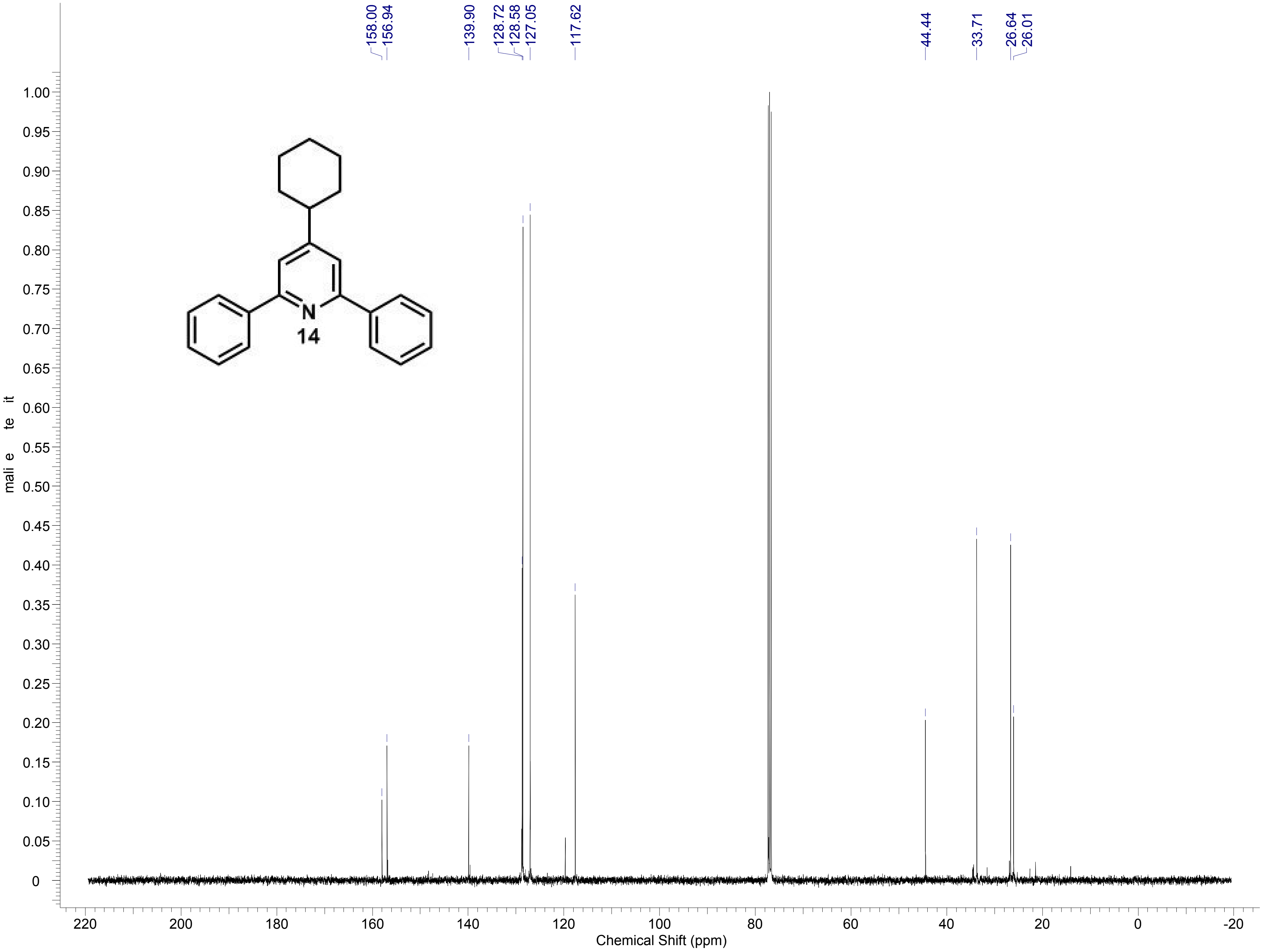
158.00
156.94
139.90
128.72
128.58
127.05
117.62
44.44
33.71
26.64
26.01
N
14
Chemical Shift (ppm)

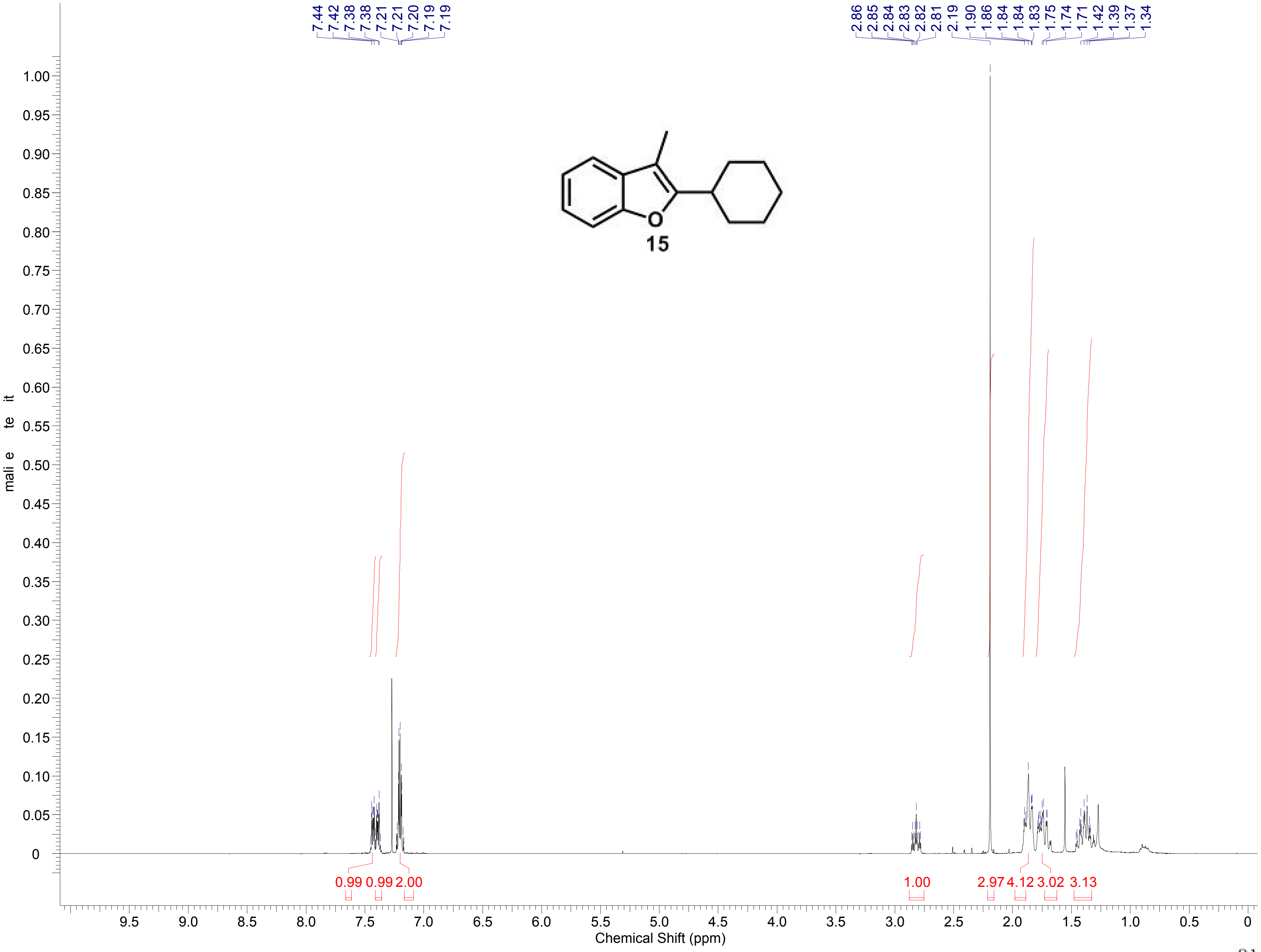
7.44
7.42
7.38
7.38
7.21
7.21
7.20
7.19
7.19
2.86
2.85
2.84
2.83
2.82
2.81
2.19
1.90
1.86
1.84
1.84
1.83
1.75
1.74
1.71
1.42
1.39
1.37
1.34
O
15
0.99
0.99
2.00
1.00
2.97
4.12
3.02
3.13
Chemical Shift (ppm)

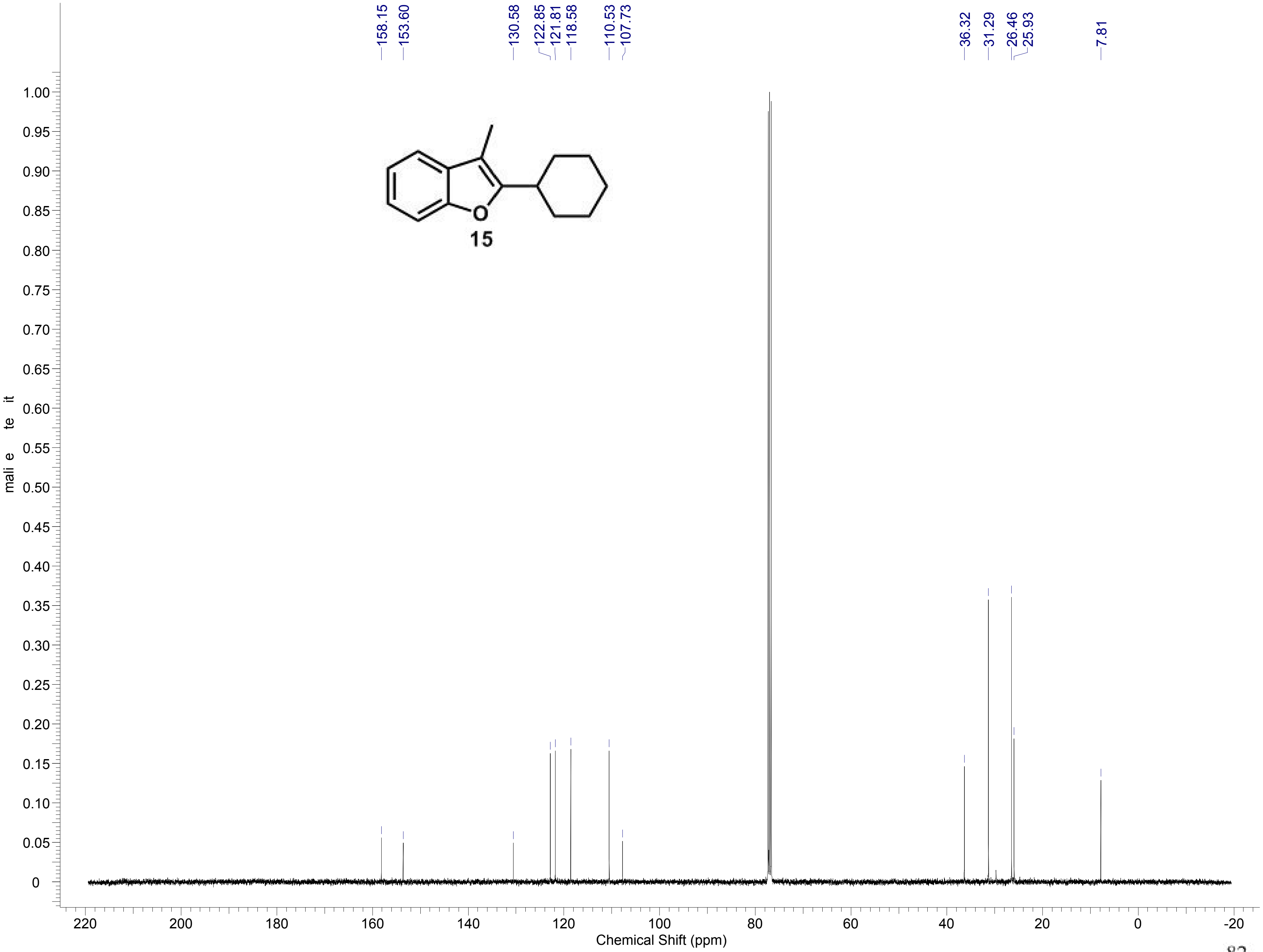

158.15
153.60
130.58
122.85
121.81
118.58
110.53
107.73
36.32
31.29
26.46
25.93
7.81
15
O
Chemical Shift (ppm)

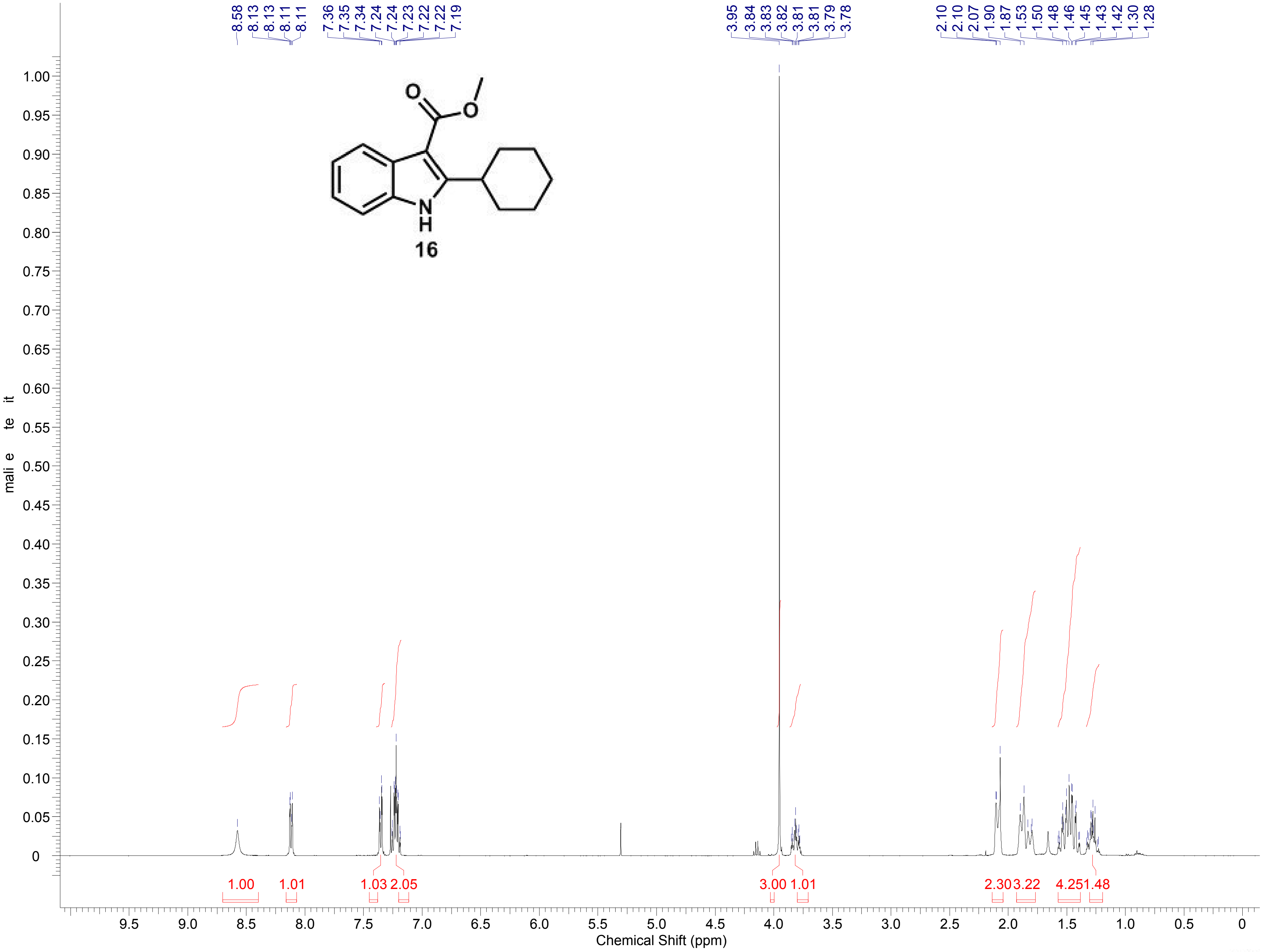
8.58
8.13
8.13
8.11
8.11
7.36
7.35
7.34
7.24
7.24
7.23
7.22
7.22
7.19
3.95
3.84
3.83
3.82
3.81
3.81
3.79
3.78
2.10
2.10
2.07
1.90
1.87
1.53
1.50
1.48
1.46
1.45
1.43
1.42
1.30
1.28
O
O
N
H
16
1.00
1.01
1.03
2.05
3.00
1.01
2.30
3.22
4.25
1.48
Chemical Shift (ppm)

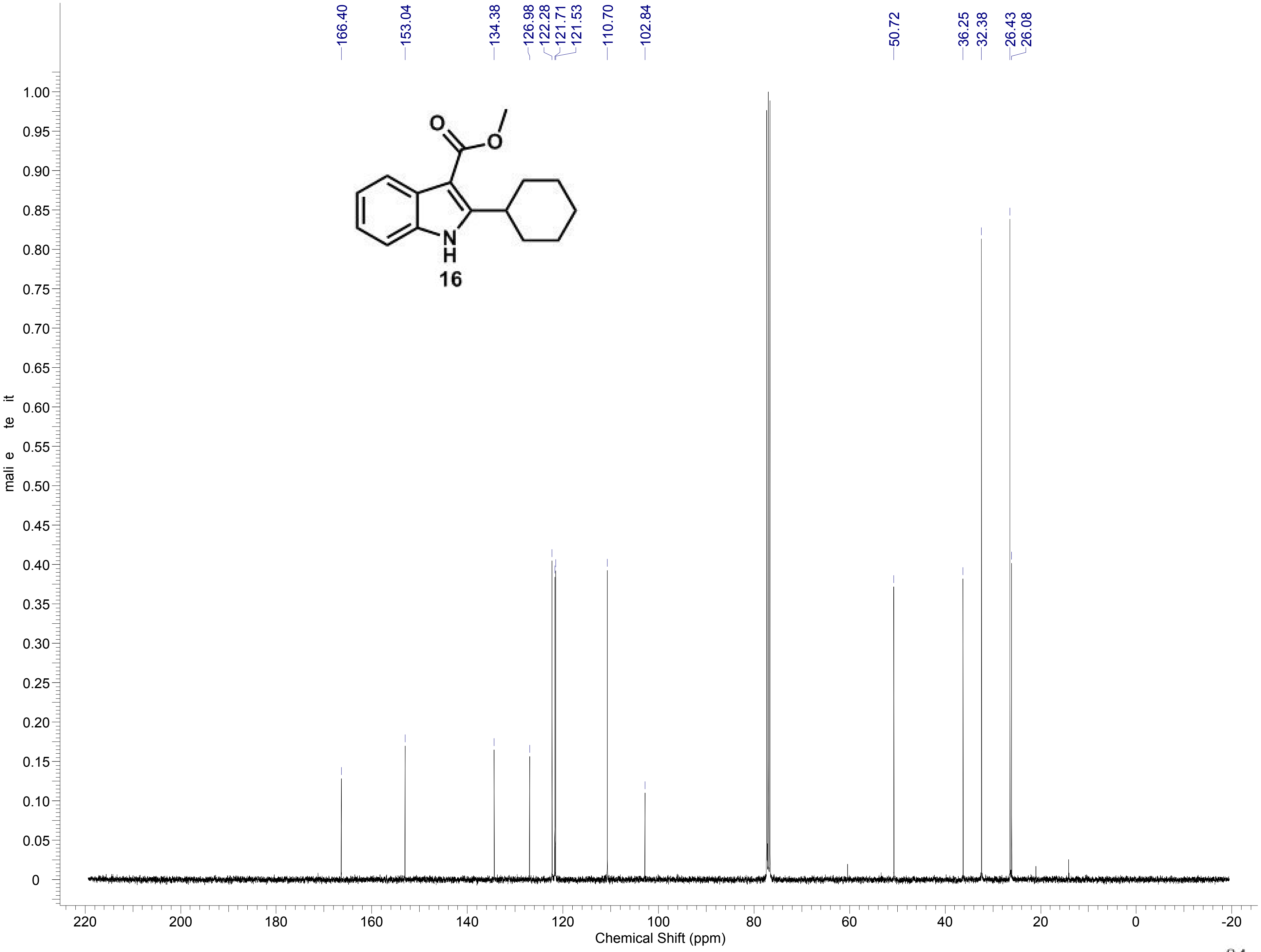

166.40
153.04
134.38
126.98
122.28
121.71
121.53
110.70
102.84
50.72
36.25
32.38
26.43
26.08
O
O
N
H
16
mali e te it
Chemical Shift (ppm)

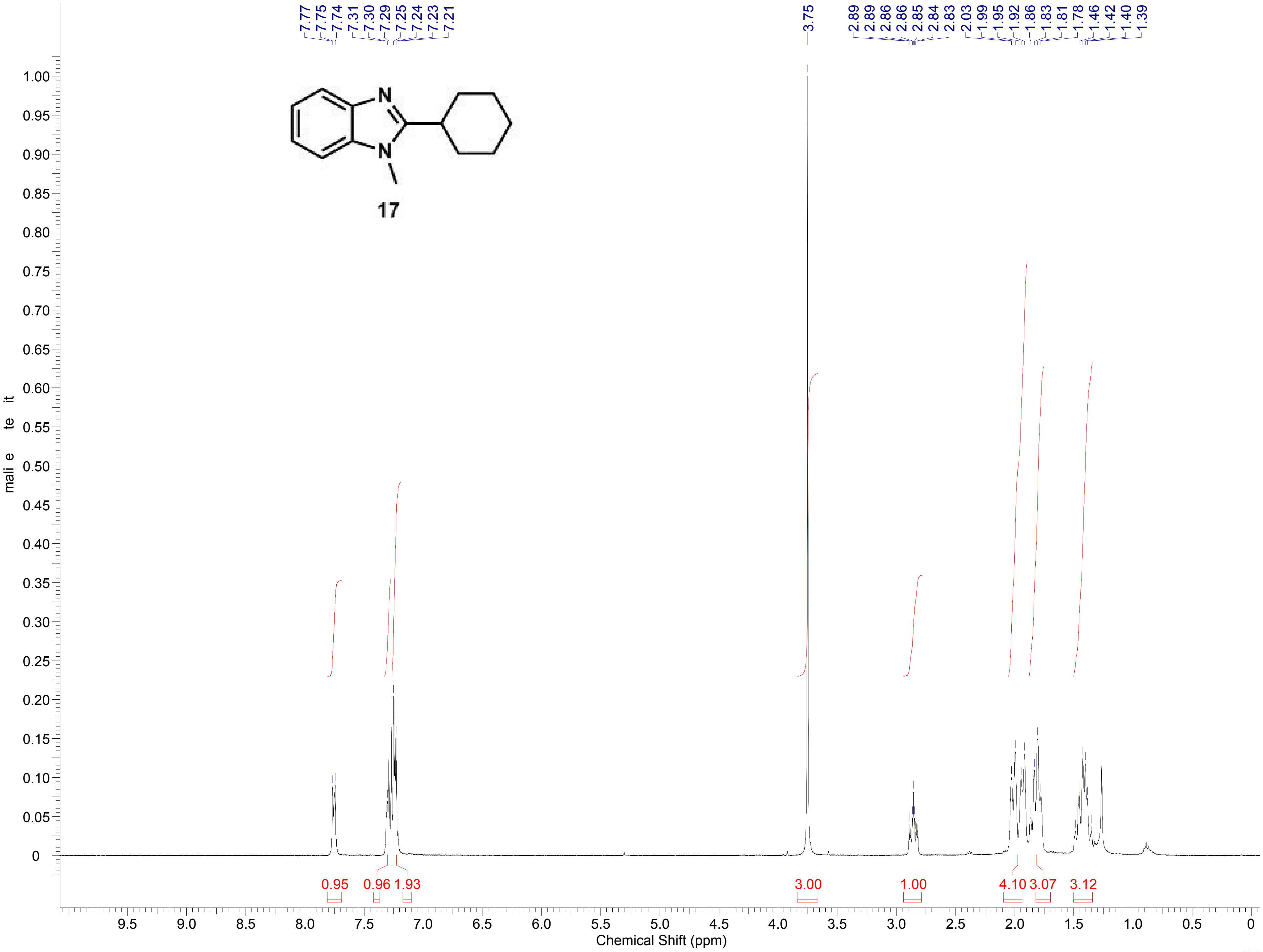

7.77 7.75 7.74 7.31 7.30 7.29 7.25 7.24 7.23 7.21
3.75
2.89 2.89 2.86 2.86 2.85 2.84 2.83 2.03 1.99 1.95 1.92 1.86 1.83 1.81 1.78 1.46 1.42 1.40 1.39
N
N
17
0.95 0.96 1.93 3.00 1.00 4.10 3.07 3.12
Chemical Shift (ppm)

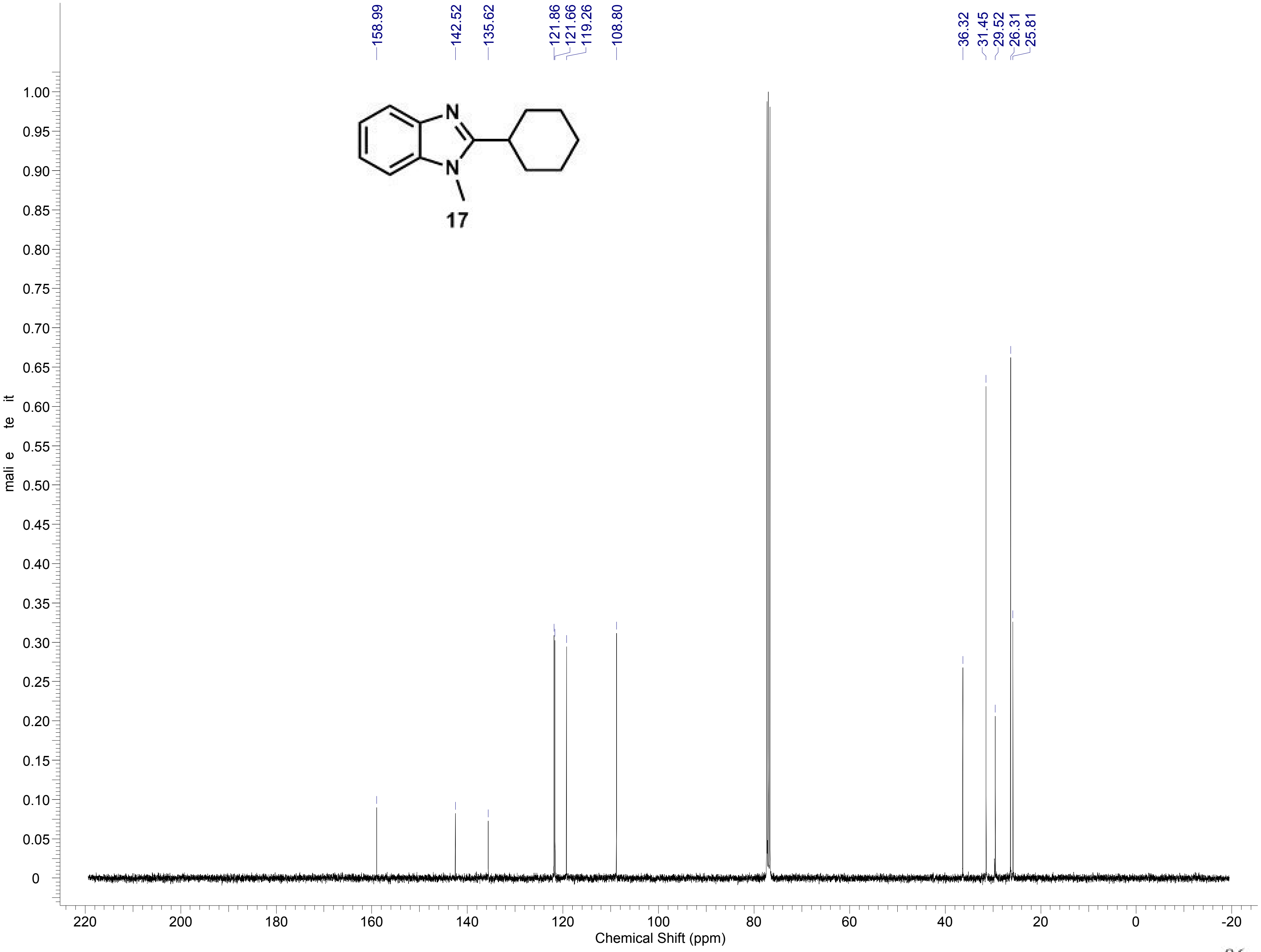

158.99
142.52
135.62
121.86
121.66
119.26
108.80
36.32
31.45
29.52
26.31
25.81
N
N
17
Chemical Shift (ppm)

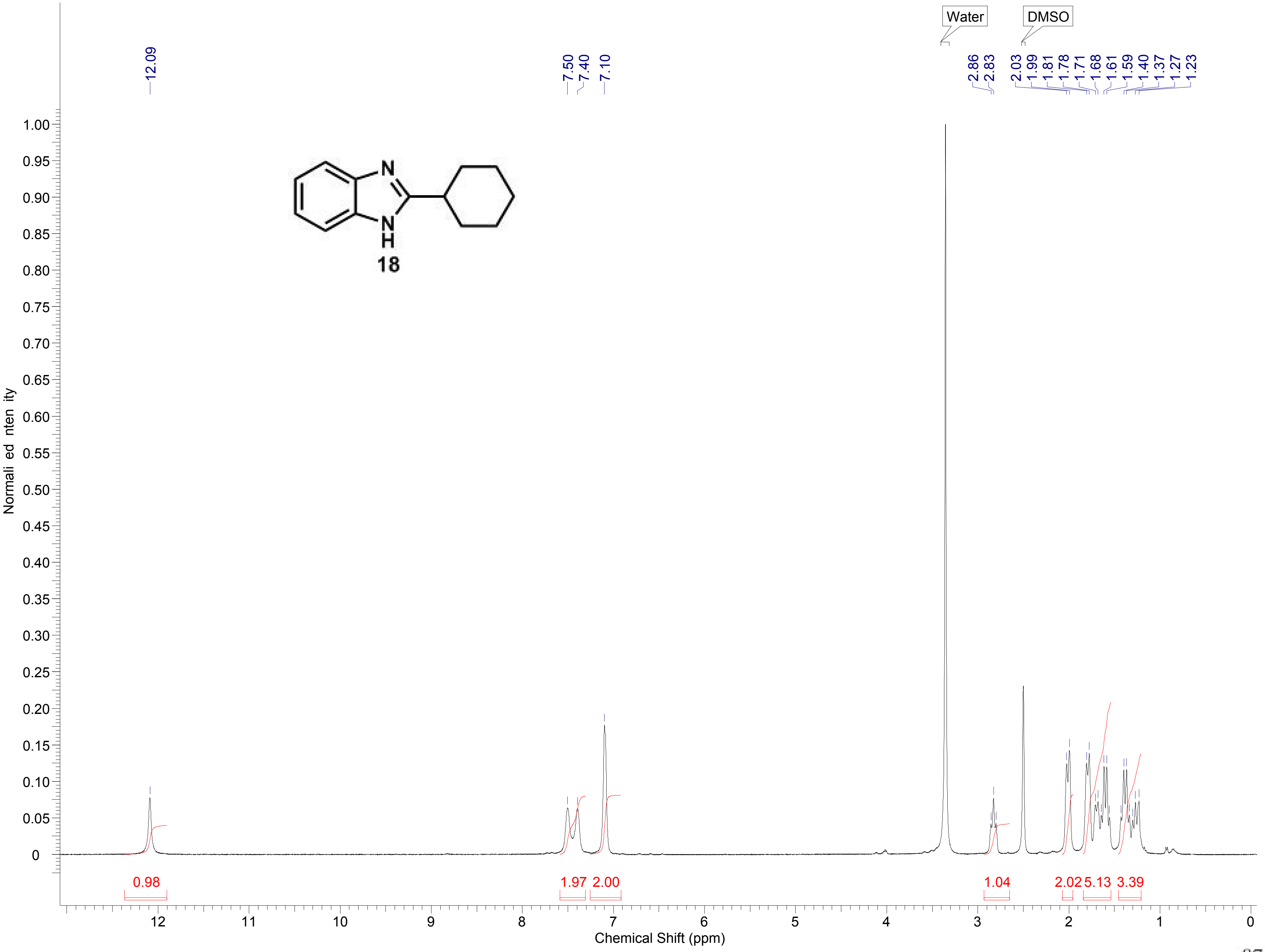
Water
DMSO
12.09
7.50
7.40
7.10
2.86
2.83
2.03
1.99
1.81
1.78
1.71
1.68
1.61
1.59
1.40
1.37
1.27
1.23
N
N
H
18
Normalized Intensity
0.98
1.97
2.00
1.04
2.02
5.13
3.39
Chemical Shift (ppm)

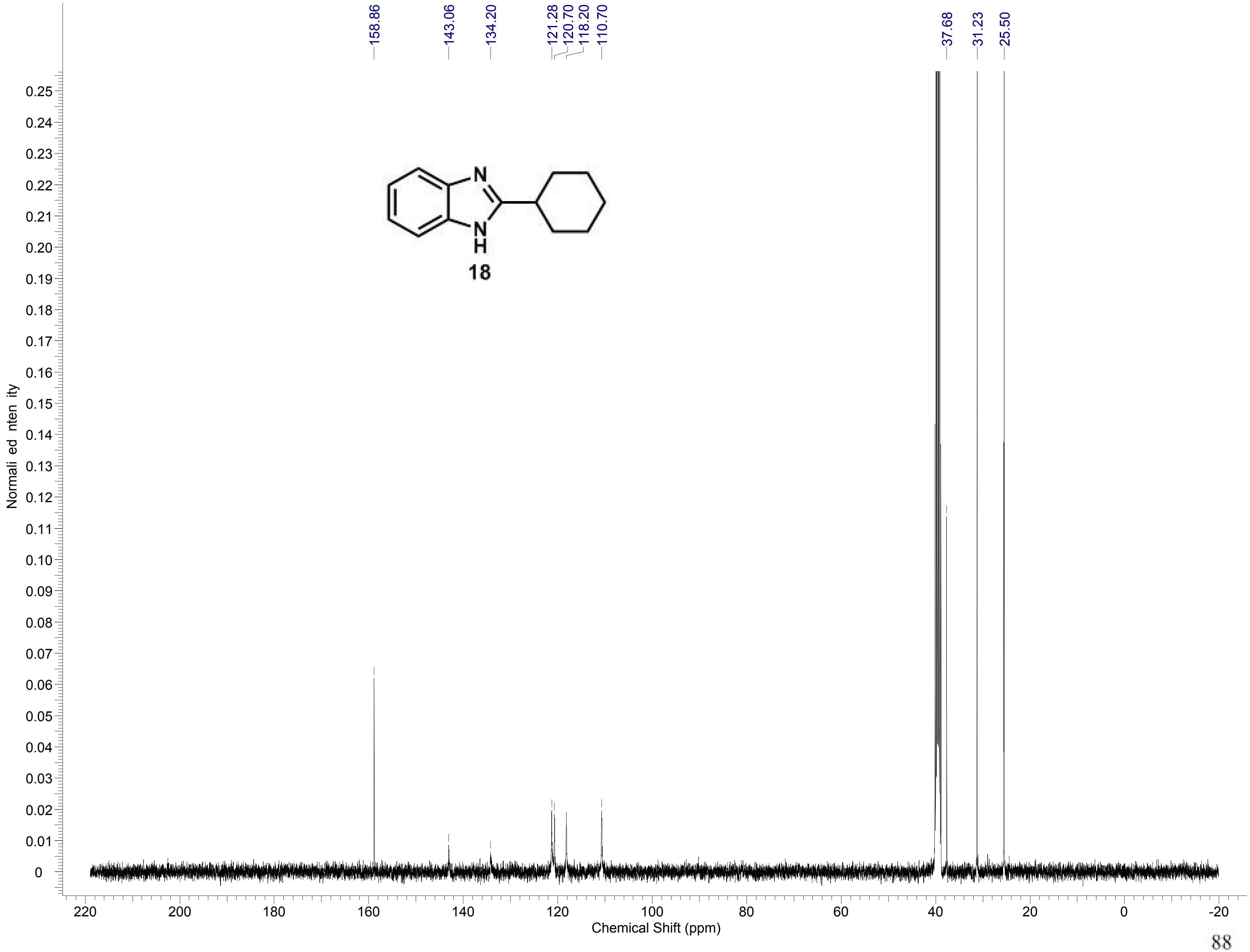

158.86
143.06
134.20
121.28
120.70
118.20
110.70
37.68
31.23
25.50
N
N
H
18
Normali ed nten ity
Chemical Shift (ppm)

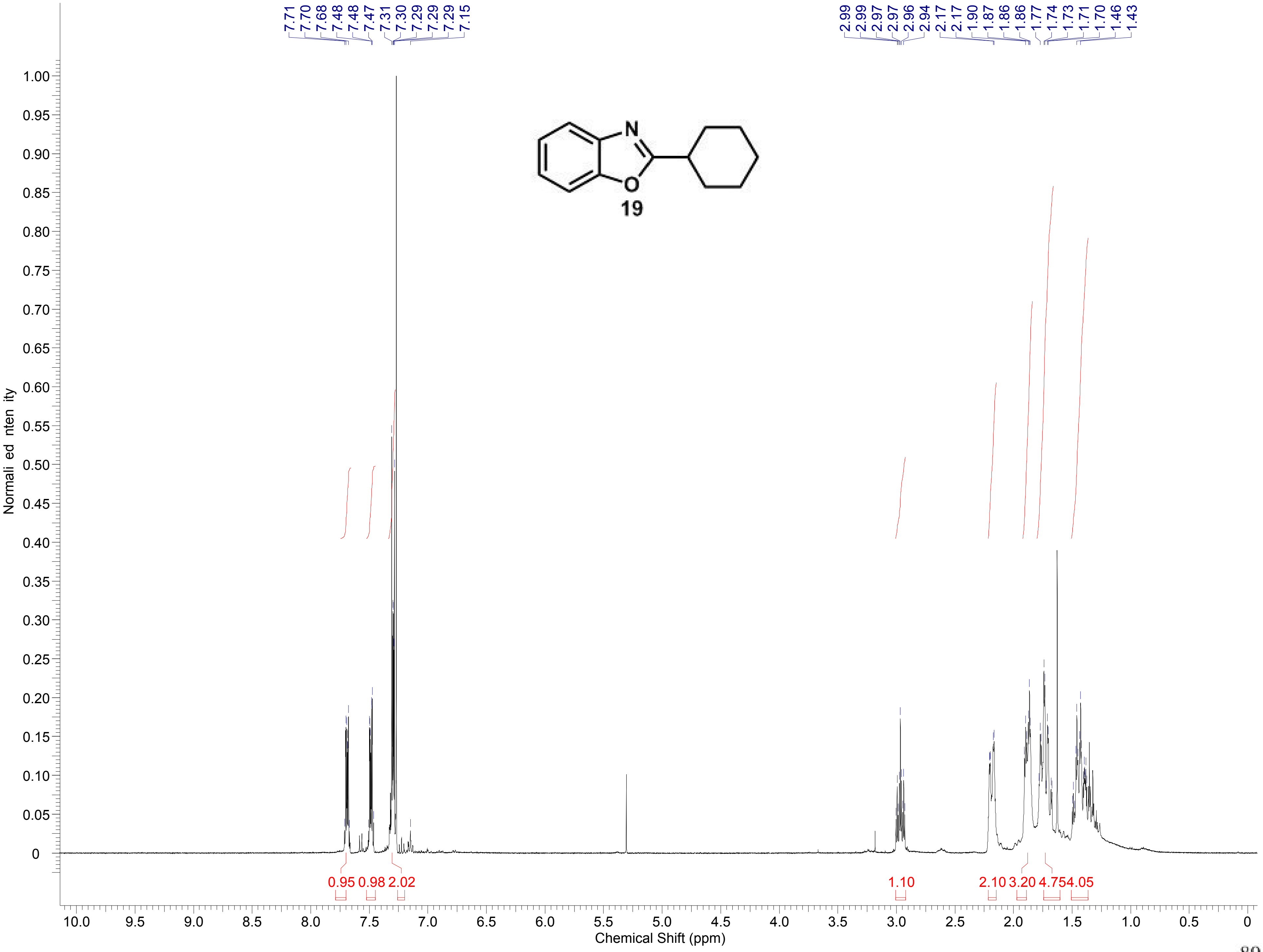
7.71 7.70 7.68 7.48 7.48 7.47 7.31 7.30 7.29 7.29 7.29 7.15
2.99 2.99 2.97 2.97 2.96 2.94 2.17 2.17 1.90 1.87 1.86 1.86 1.77 1.74 1.73 1.71 1.70 1.46 1.43
N
O
19
0.95 0.98 2.02 1.10 2.10 3.20 4.75 4.05
Normalized Intensity
Chemical Shift (ppm)

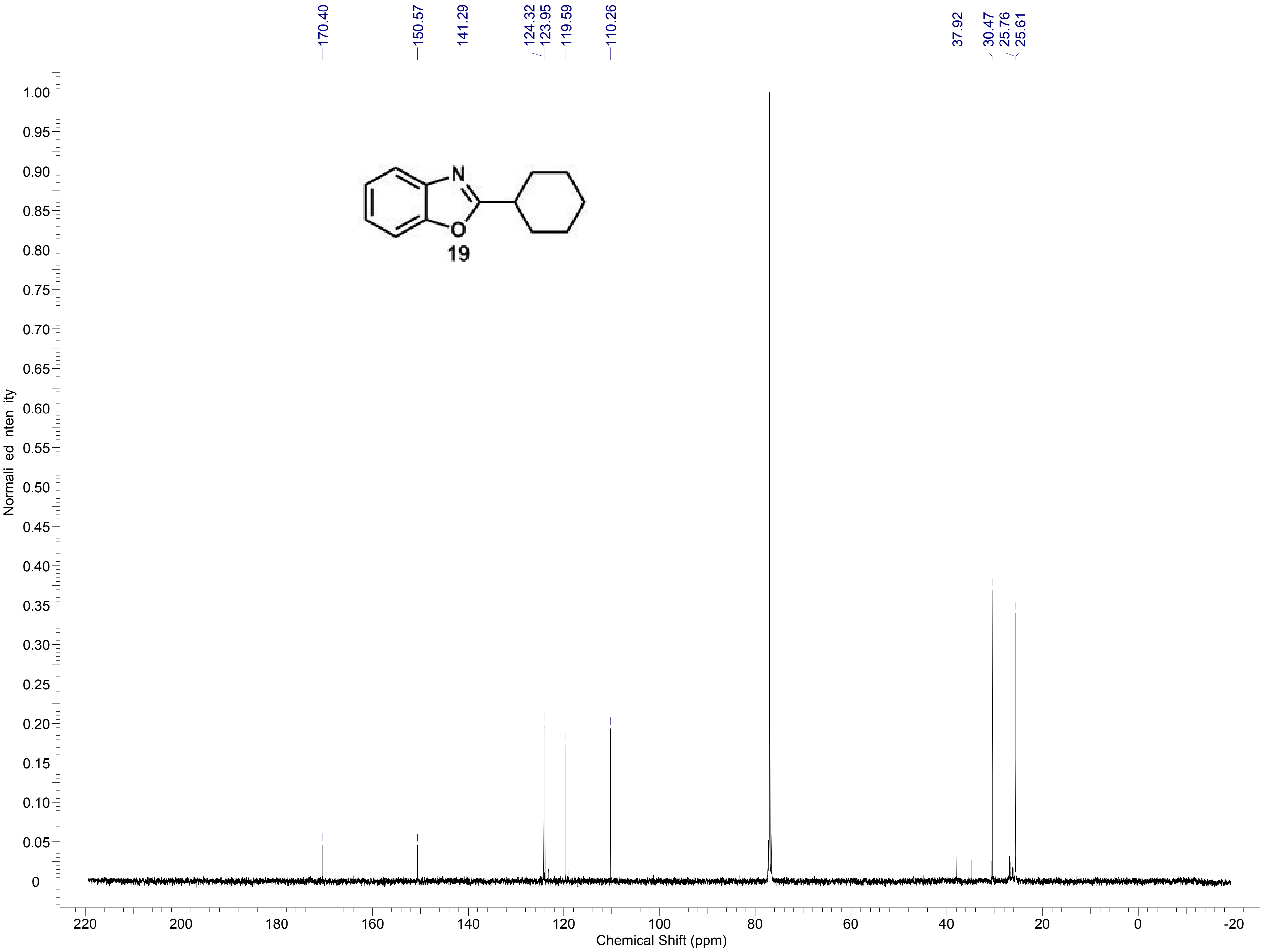
170.40
150.57
141.29
124.32
123.95
119.59
110.26
37.92
30.47
25.76
25.61
N
O
19
Normalized Intensity
Chemical Shift (ppm)

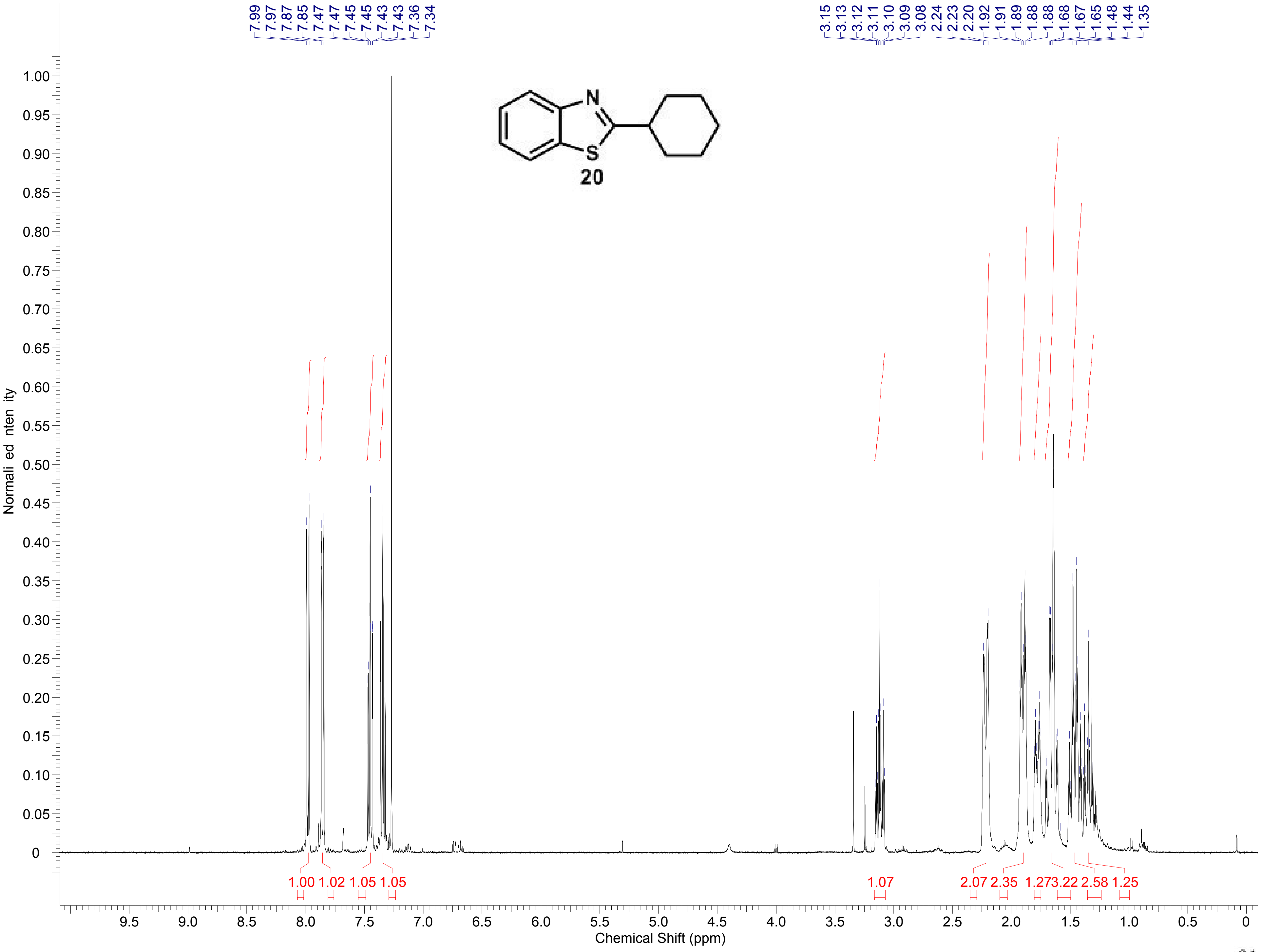
7.99
7.97
7.87
7.85
7.47
7.47
7.45
7.45
7.43
7.43
7.36
7.34
3.15
3.13
3.12
3.11
3.10
3.09
3.08
2.24
2.23
2.20
1.92
1.91
1.89
1.88
1.88
1.68
1.67
1.65
1.48
1.44
1.35
N
S
20
1.00
1.02
1.05
1.05
1.07
2.07
2.35
1.27
3.22
2.58
1.25
Normali ed nten ity
Chemical Shift (ppm)

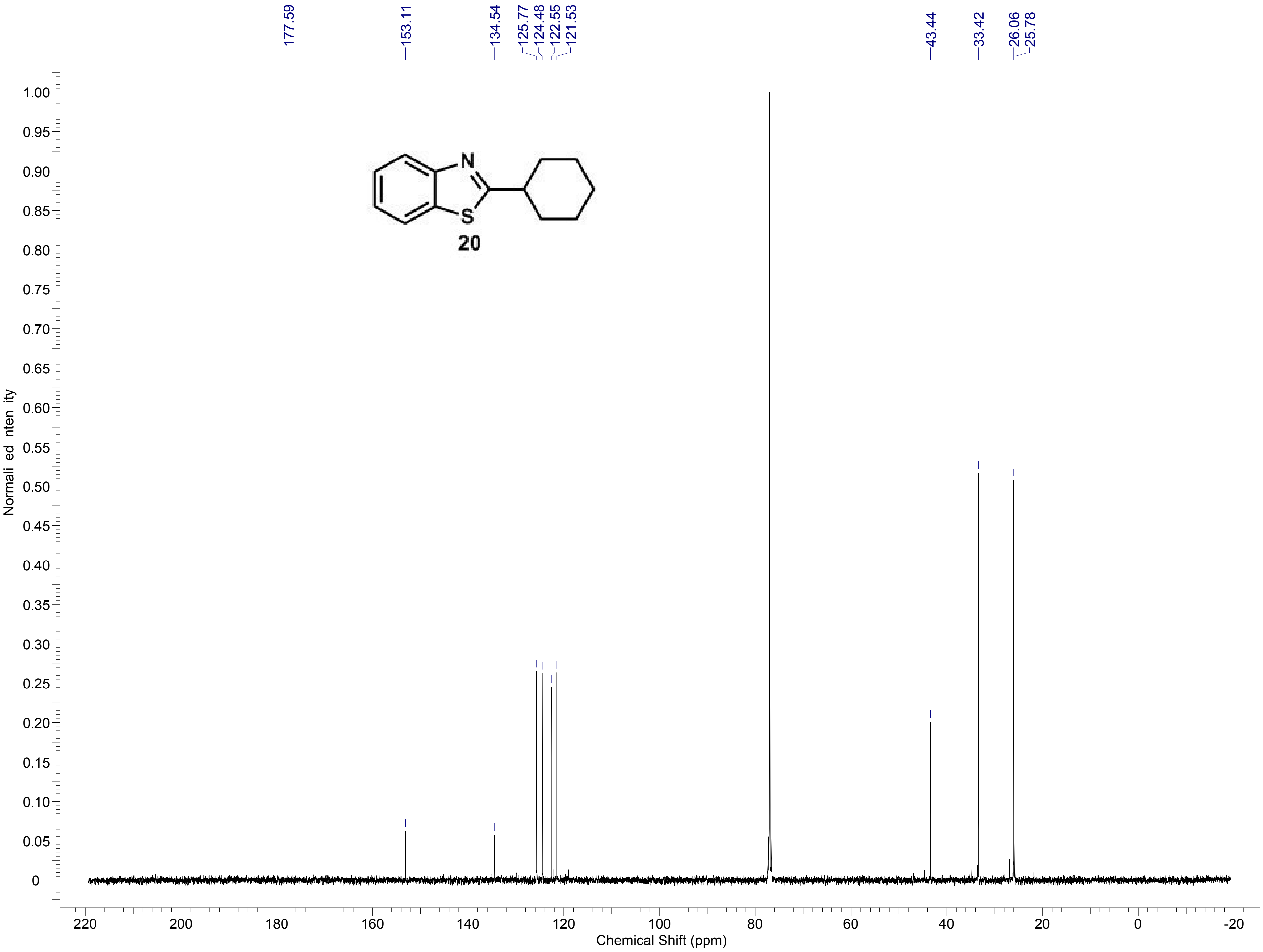
177.59
153.11
134.54
125.77
124.48
122.55
121.53
43.44
33.42
26.06
25.78
N
S
20
Normali ed nten ity
1.00
0.95
0.90
0.85
0.80
0.75
0.70
0.65
0.60
0.55
0.50
0.45
0.40
0.35
0.30
0.25
0.20
0.15
0.10
0.05
0
220
200
180
160
140
120
100
80
60
40
20
0
-20
Chemical Shift (ppm)

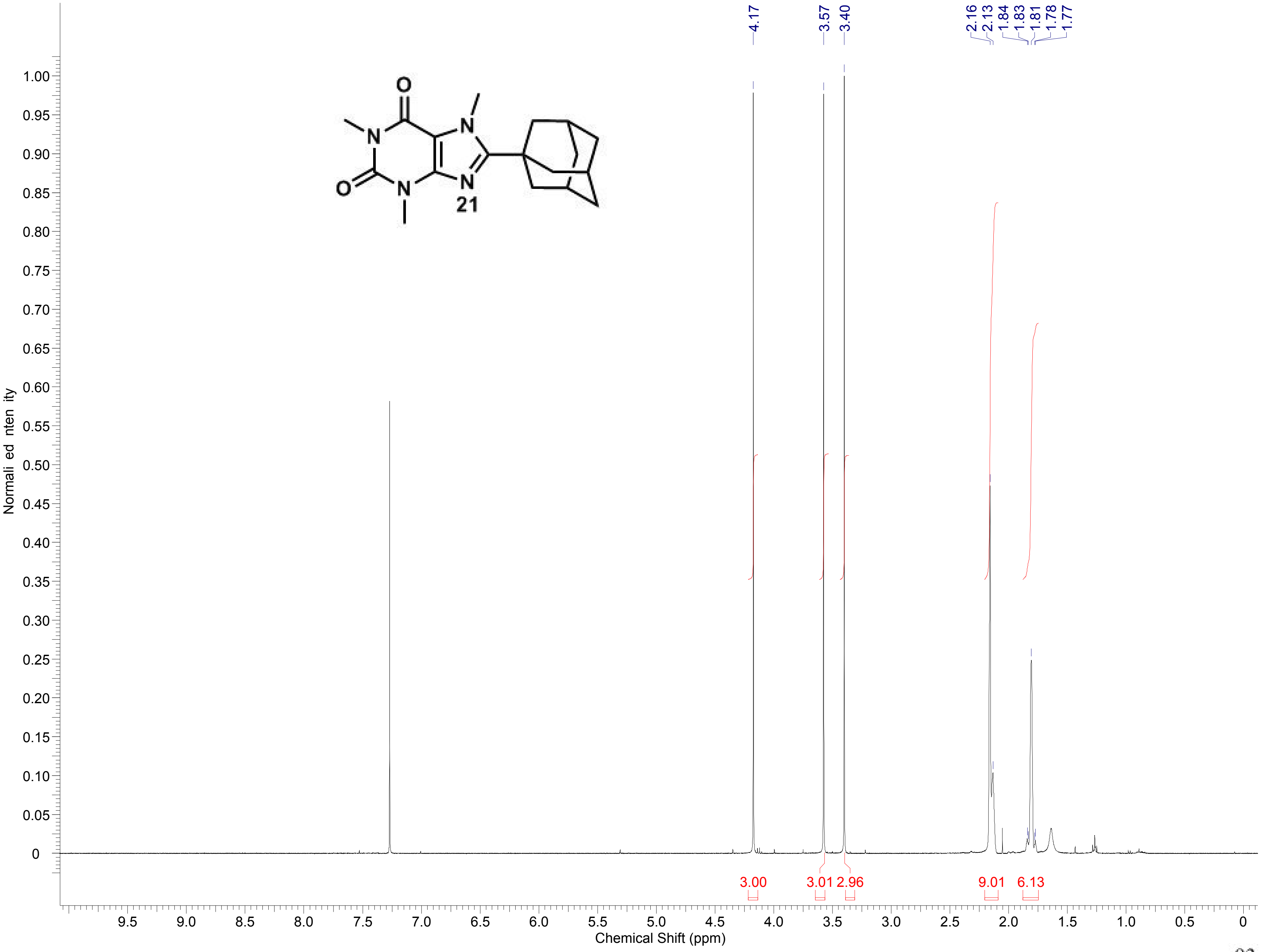
4.17
3.57
3.40
2.16
2.13
1.84
1.83
1.81
1.78
1.77
21
3.00
3.01
2.96
9.01
6.13
Normalized Intensity
Chemical Shift (ppm)

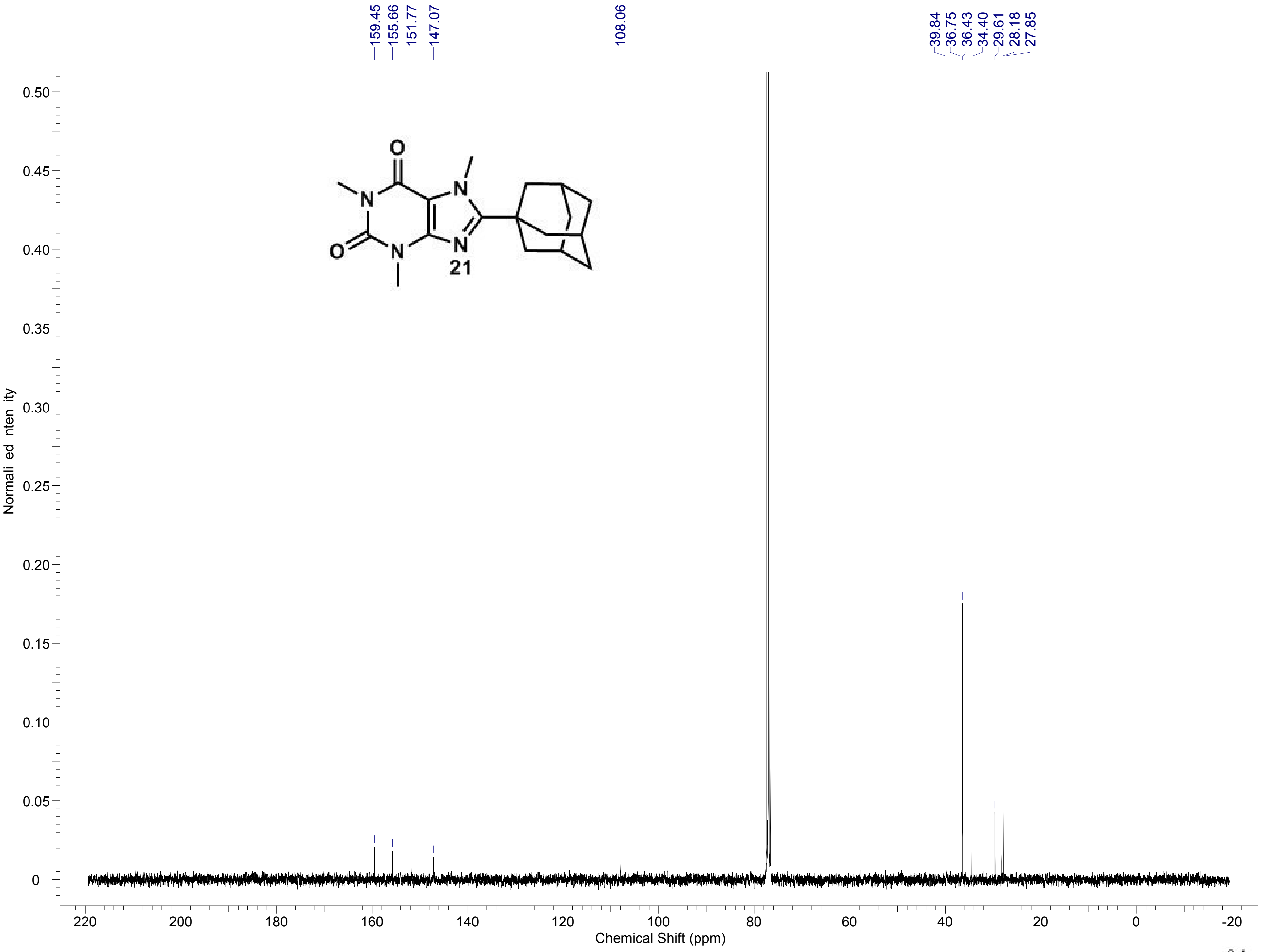
159.45
155.66
151.77
147.07
108.06
39.84
36.75
36.43
34.40
29.61
28.18
27.85
21
Chemical Shift (ppm)

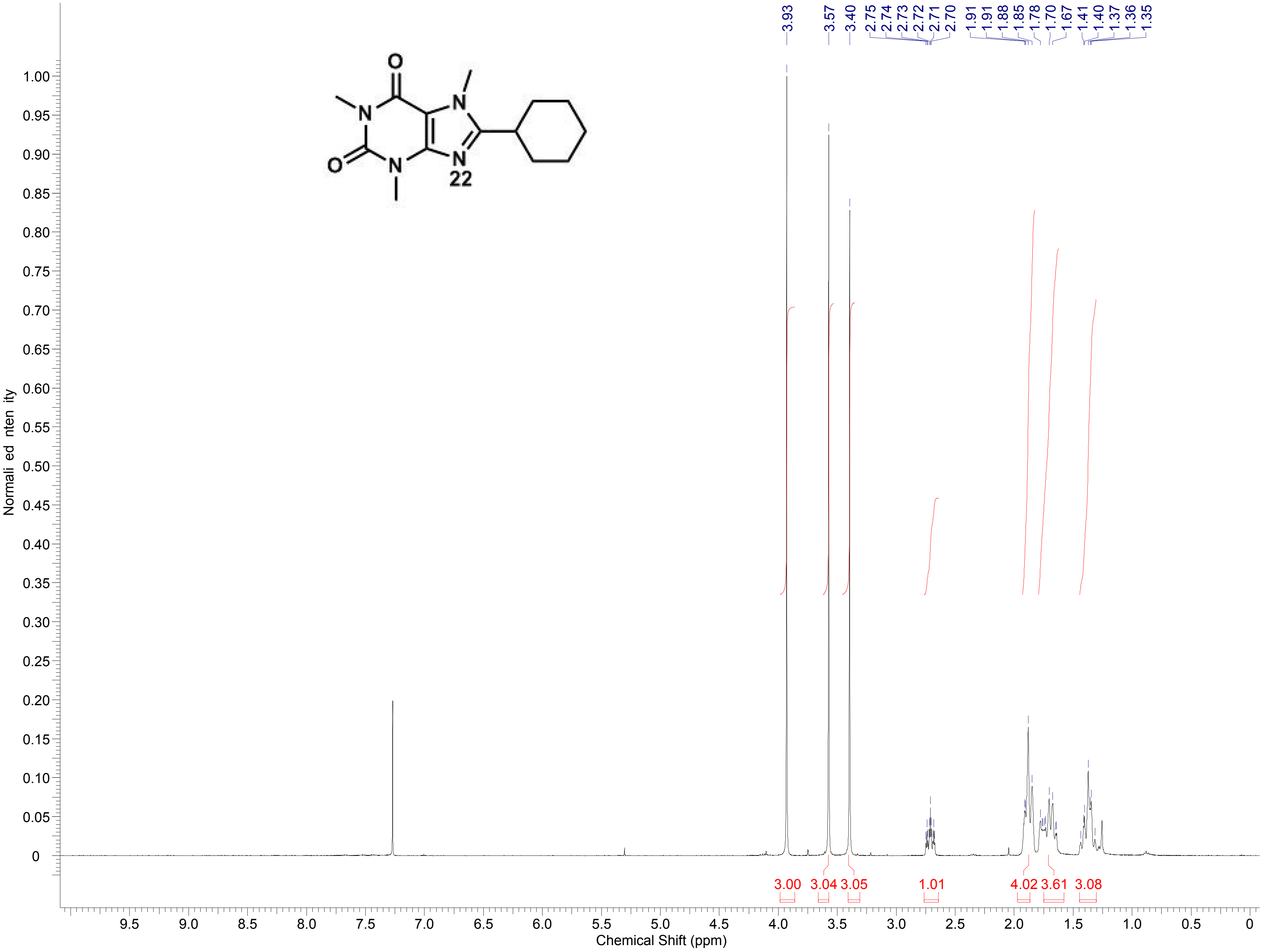

3.93
3.57
3.40
2.75
2.74
2.73
2.72
2.71
2.70
1.91
1.91
1.88
1.85
1.78
1.70
1.67
1.41
1.40
1.37
1.36
1.35
22
3.00
3.04
3.05
1.01
4.02
3.61
3.08
Normali ed nten ity
Chemical Shift (ppm)

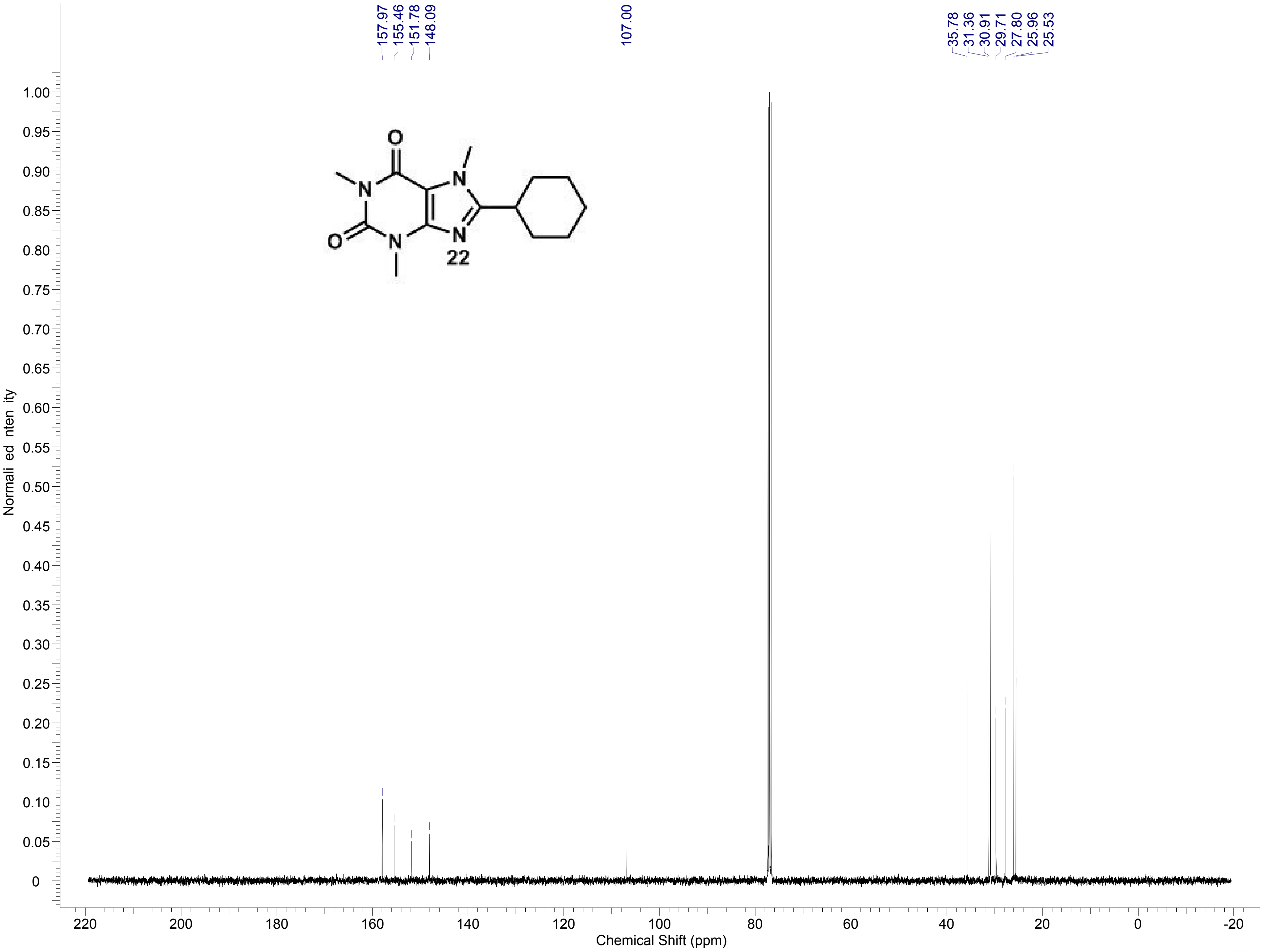
157.97
155.46
151.78
148.09
107.00
35.78
31.36
30.91
29.71
27.80
25.96
25.53
22
Normali ed nten ity
Chemical Shift (ppm)

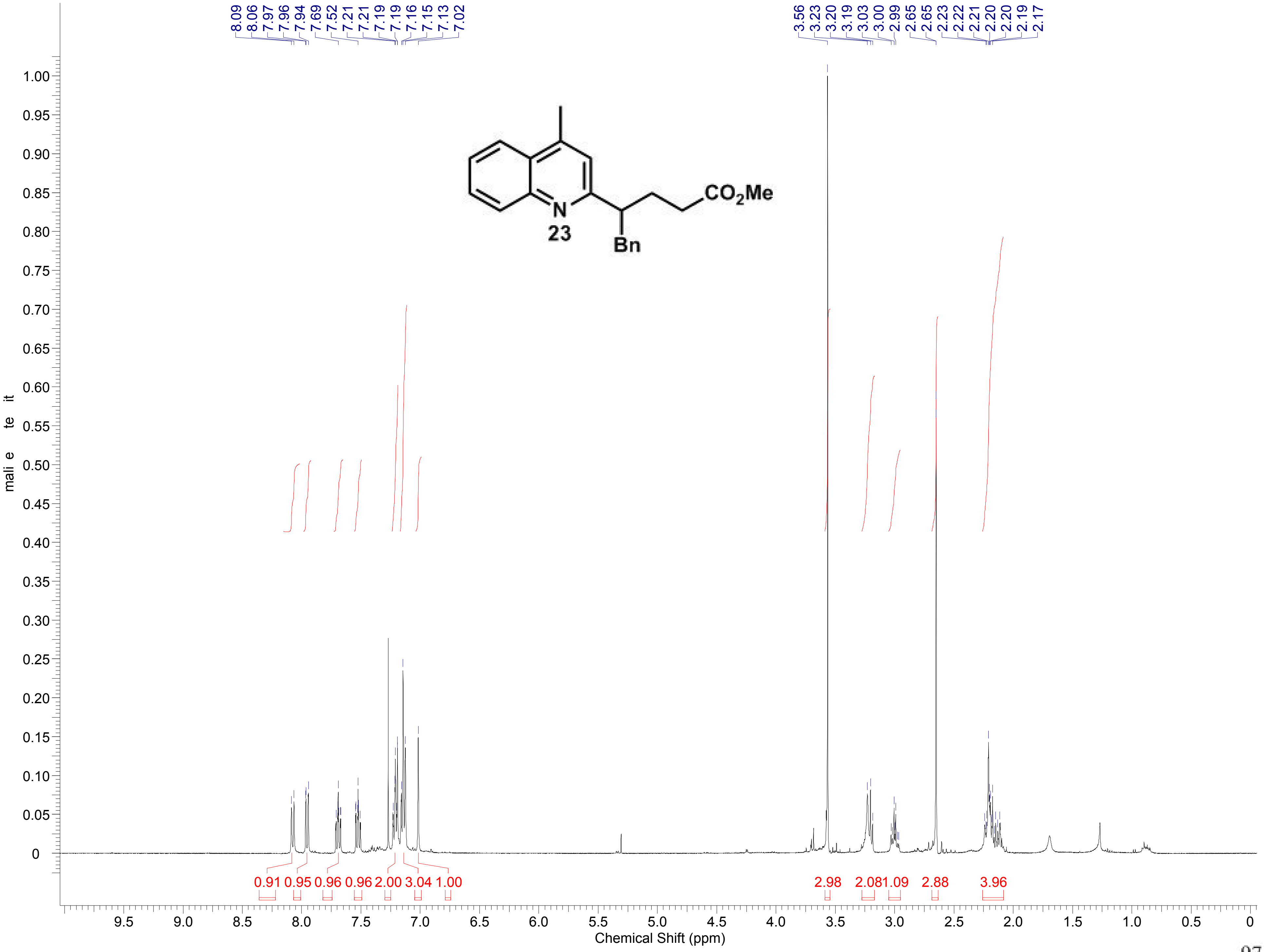

8.09 8.06 7.97 7.96 7.94 7.69 7.52 7.21 7.21 7.19 7.19 7.16 7.15 7.13 7.02
3.56 3.23 3.20 3.19 3.03 3.00 2.99 2.65 2.65 2.23 2.22 2.21 2.20 2.20 2.19 2.17
CO_2Me
N
23
Bn
0.91 0.95 0.96 0.96 2.00 3.04 1.00
2.98 2.08 1.09 2.88 3.96
Chemical Shift (ppm)

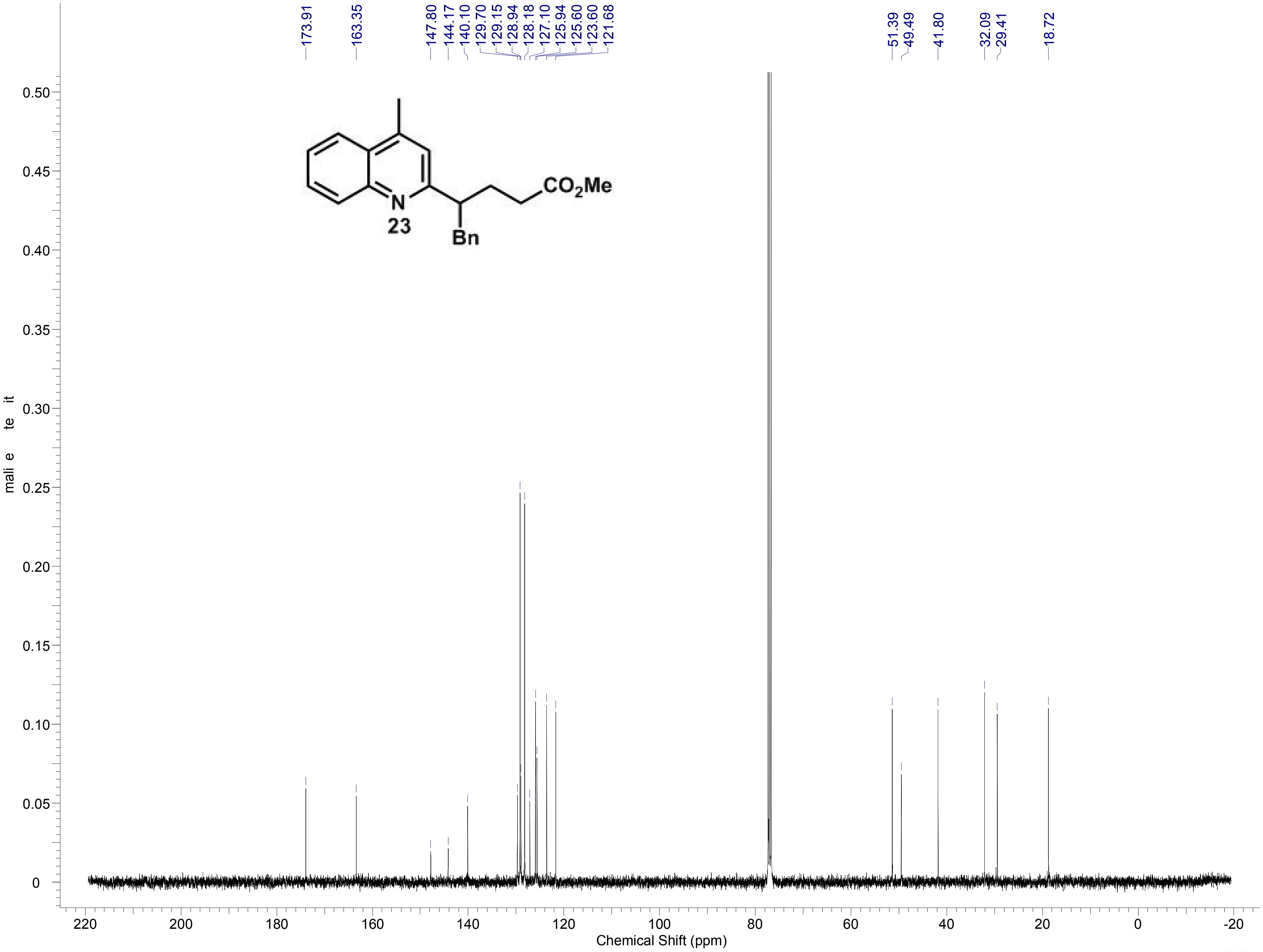
173.91
163.35
147.80
144.17
140.10
129.70
129.15
128.94
128.18
127.10
125.94
125.60
123.60
121.68
51.39
49.49
41.80
32.09
29.41
18.72
CO_2Me
N
23
Bn
mali e te it
Chemical Shift (ppm)

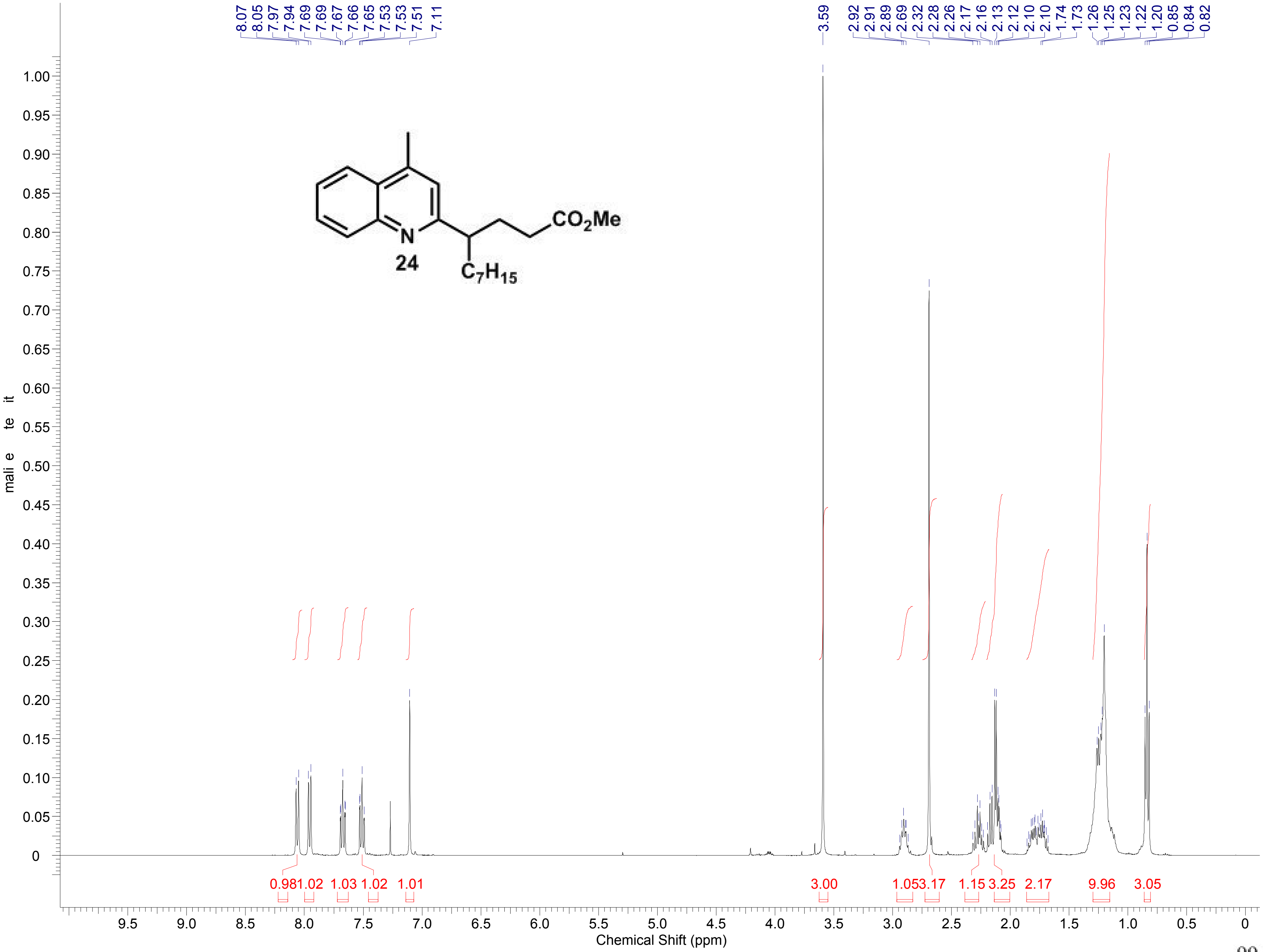
8.07 8.05 7.97 7.94 7.69 7.69 7.67 7.66 7.65 7.53 7.53 7.51 7.11
3.59
2.92 2.91 2.89 2.69 2.32 2.28 2.26 2.17 2.16 2.13 2.12 2.10 2.10 1.74 1.73 1.26 1.25 1.23 1.22 1.20 0.85 0.84 0.82
N
CO2Me
24
C7H15
0.98 1.02 1.03 1.02 1.01
3.00
1.05 3.17 1.15 3.25 2.17 9.96 3.05
Chemical Shift (ppm)

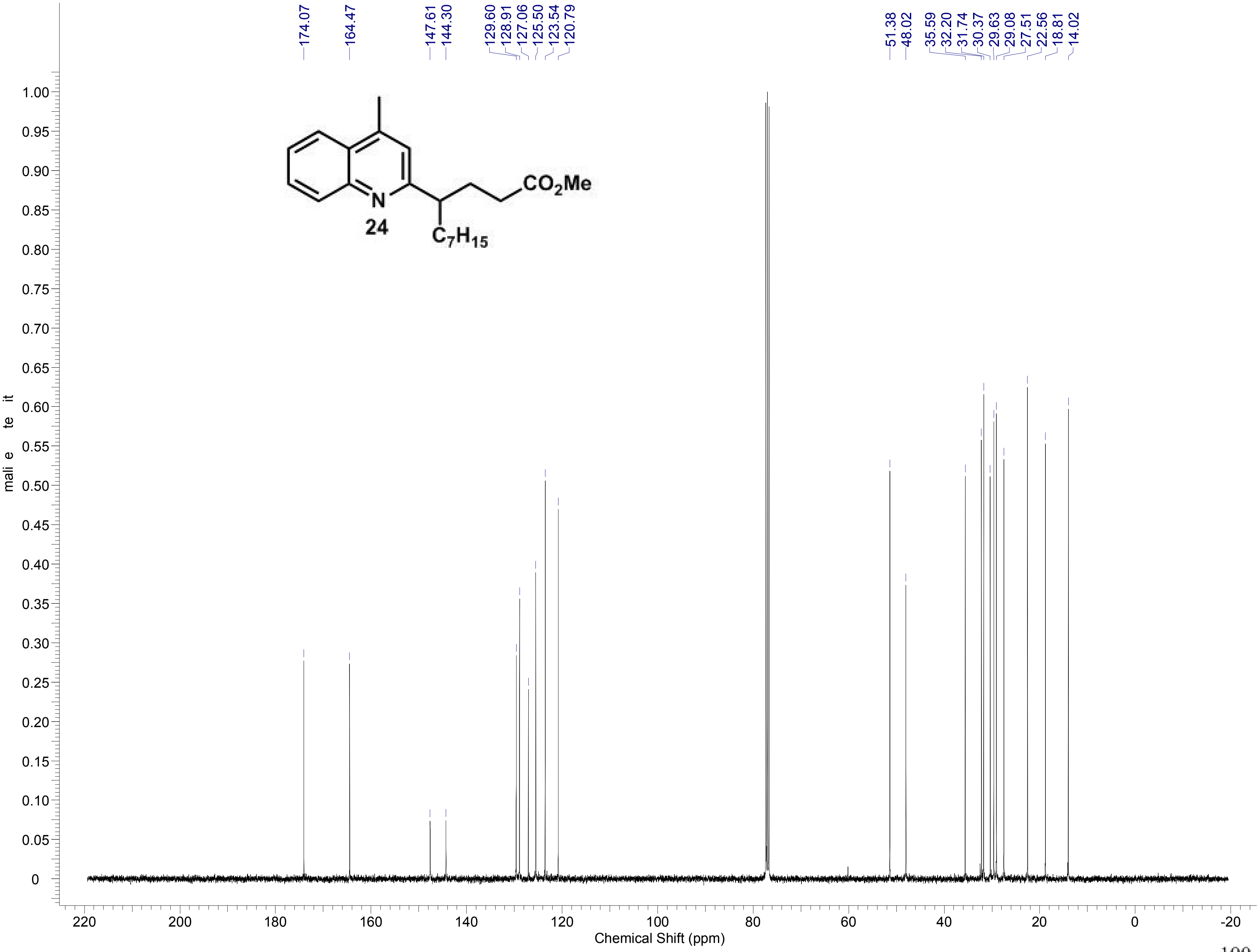

174.07
164.47
147.61
144.30
129.60
128.91
127.06
125.50
123.54
120.79
51.38
48.02
35.59
32.20
31.74
30.37
29.63
29.08
27.51
22.56
18.81
14.02
CO_2Me
N
24
C_7H_{15}
mali e te it
Chemical Shift (ppm)

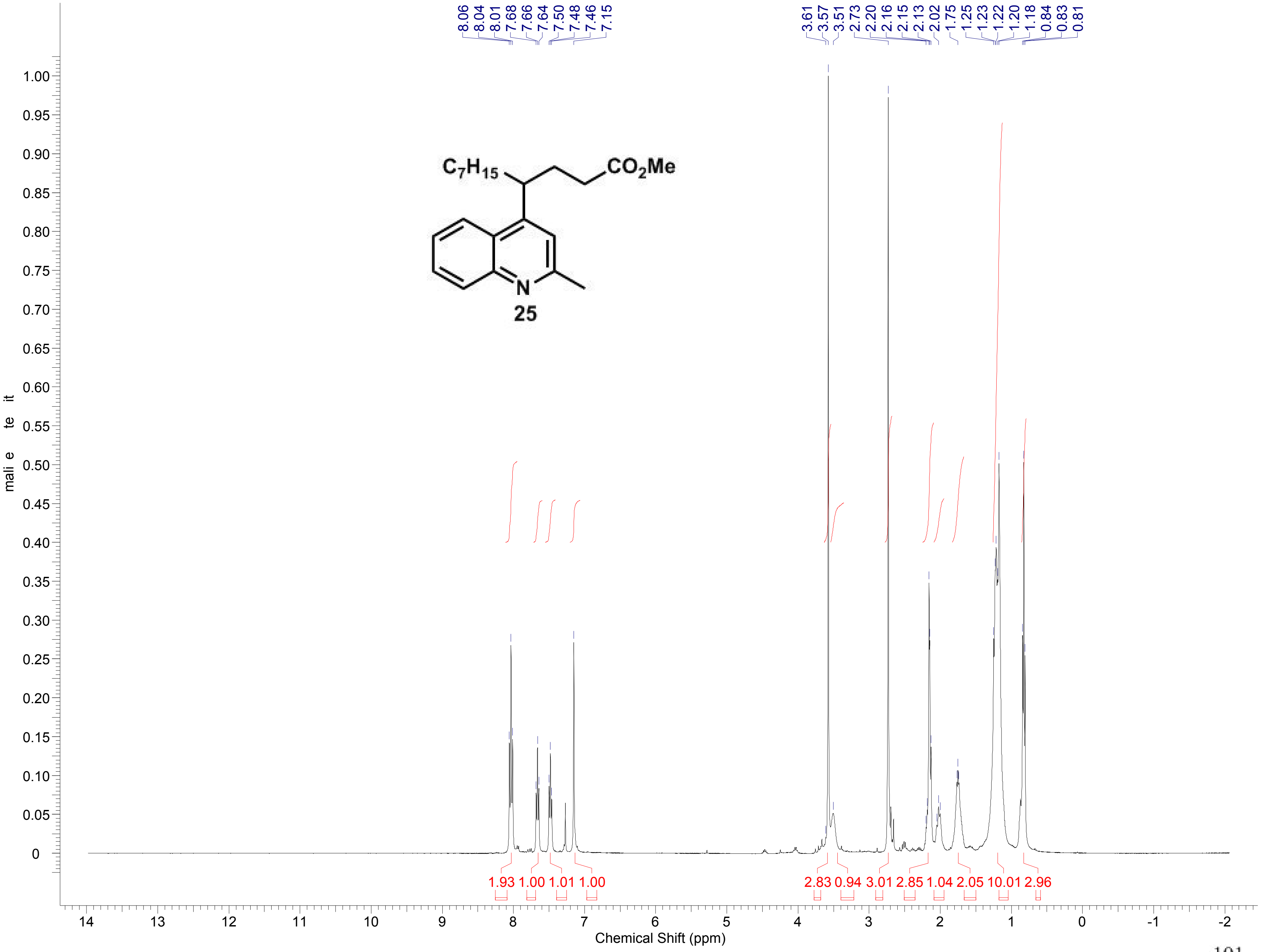

C7H15
CO2Me
N
25
8.06 8.04 8.01 7.68 7.66 7.64 7.50 7.48 7.46 7.15
3.61 3.57 3.51 2.73 2.20 2.16 2.15 2.13 2.02 1.75 1.25 1.23 1.22 1.20 1.18 0.84 0.83 0.81
1.93 1.00 1.01 1.00
2.83 0.94 3.01 2.85 1.04 2.05 10.01 2.96
Chemical Shift (ppm)

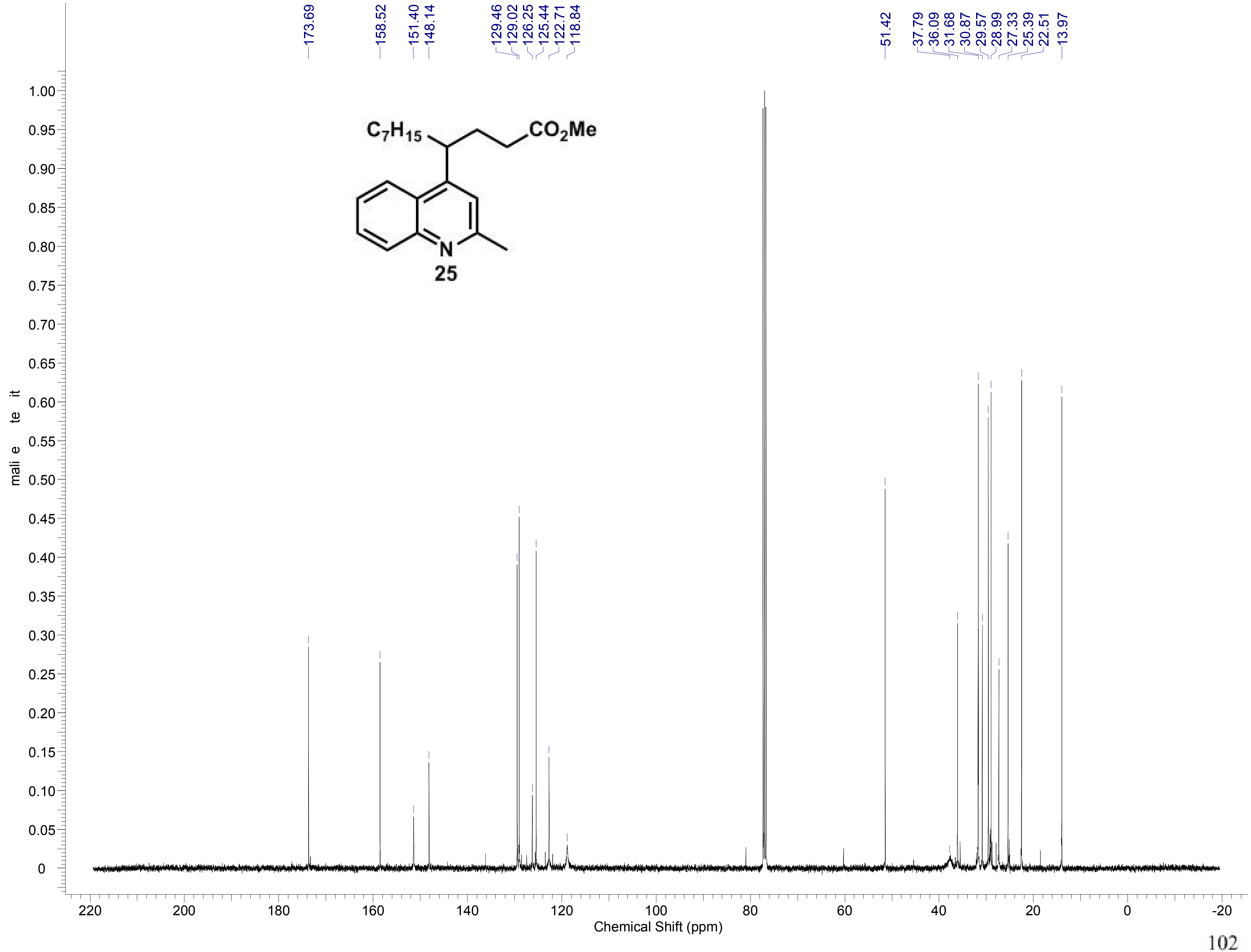
173.69
158.52
151.40
148.14
129.46
129.02
126.25
125.44
122.71
118.84
51.42
37.79
36.09
31.68
30.87
29.57
28.99
27.33
25.39
22.51
13.97
C_7H_{15}
CO_2Me
N
25
Chemical Shift (ppm)

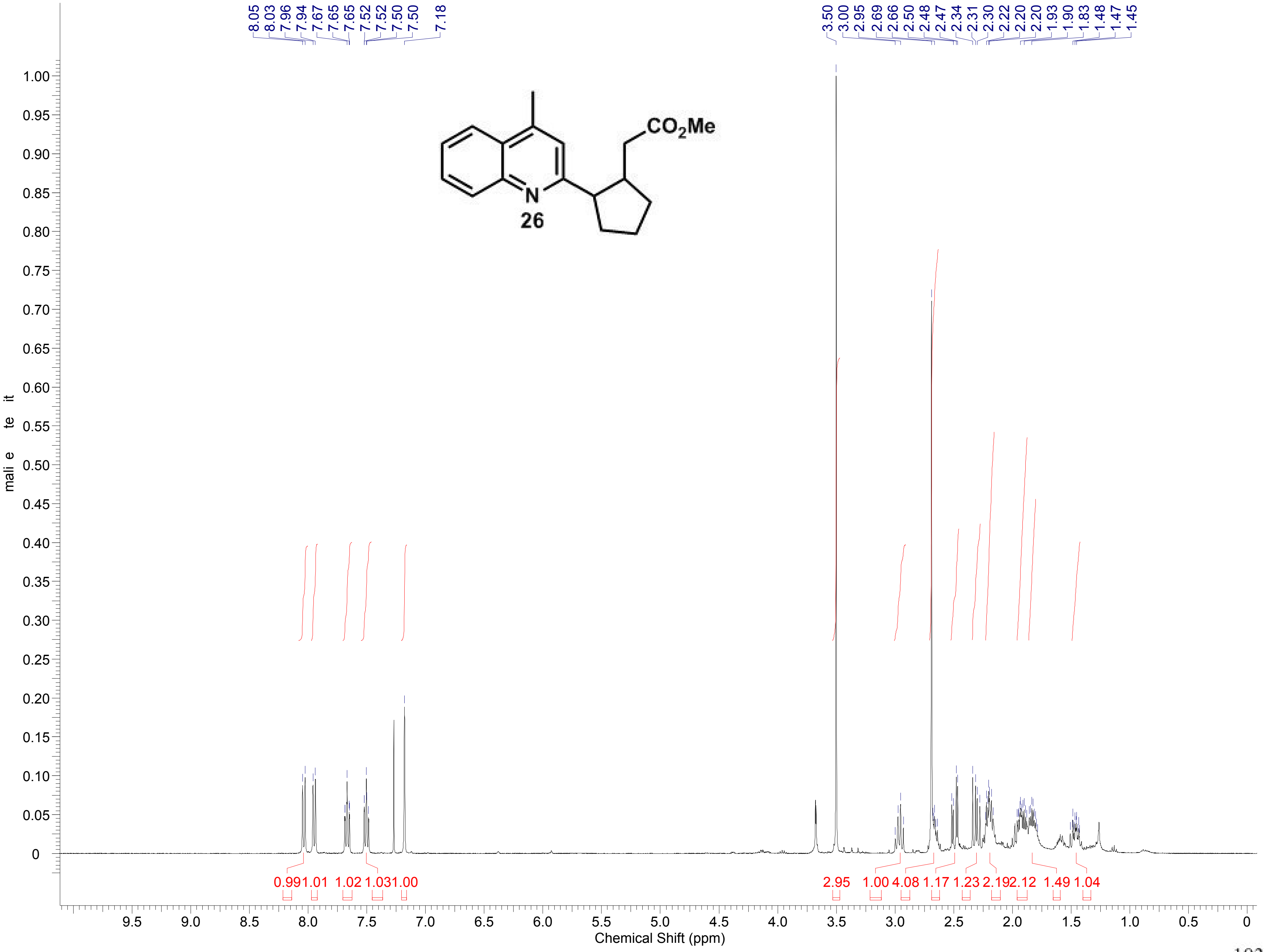

8.05 8.03 7.96 7.94 7.67 7.65 7.65 7.52 7.52 7.50 7.50 7.18
3.50 3.00 2.95 2.69 2.66 2.50 2.48 2.47 2.34 2.31 2.30 2.22 2.20 2.20 1.93 1.90 1.83 1.48 1.47 1.45
CO2Me
N
26
0.99 1.01 1.02 1.03 1.00
2.95 1.00 4.08 1.17 1.23 2.19 2.12 1.49 1.04
Chemical Shift (ppm)

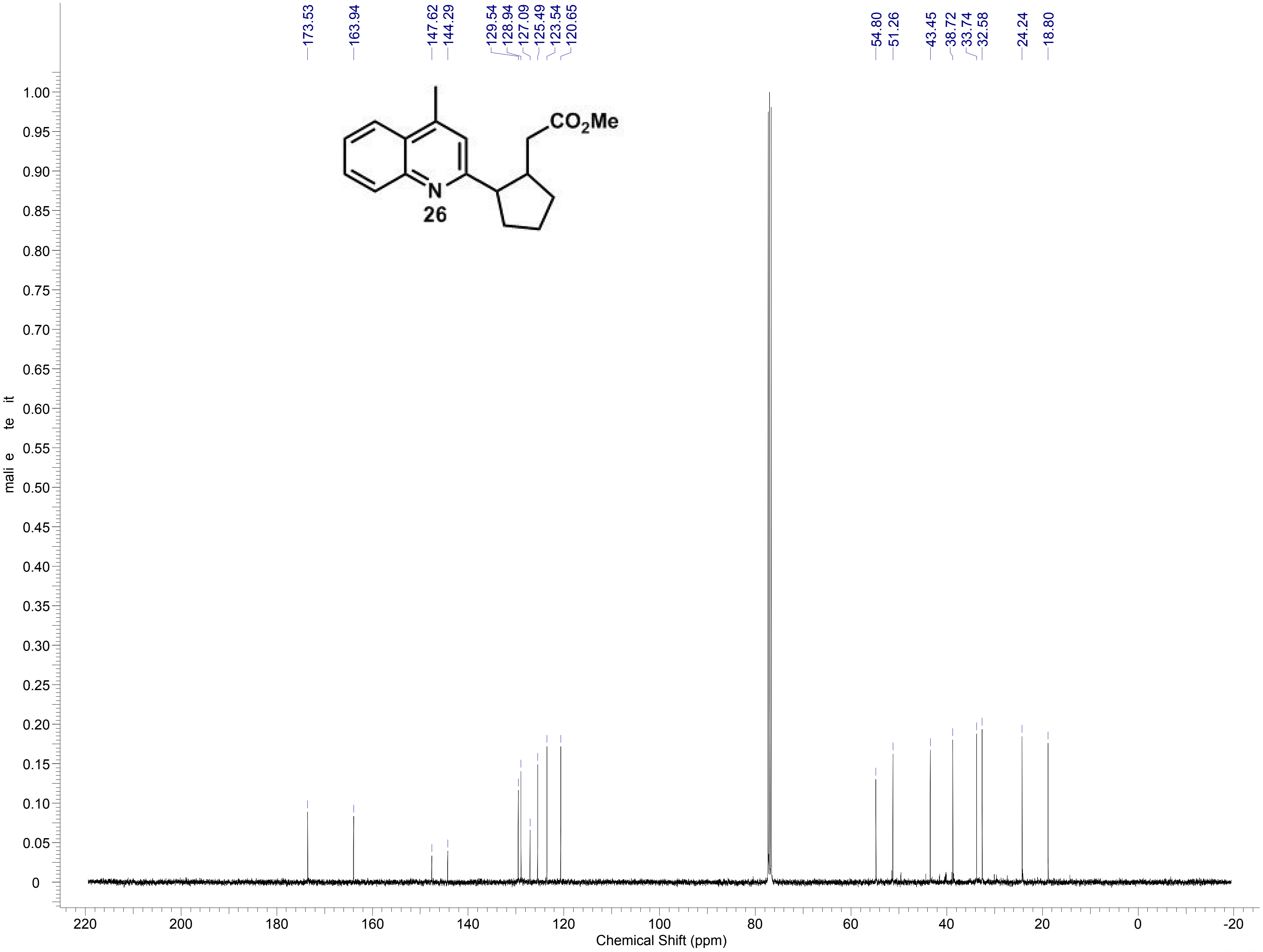
173.53
163.94
147.62
144.29
129.54
128.94
127.09
125.49
123.54
120.65
54.80
51.26
43.45
38.72
33.74
32.58
24.24
18.80
CO2Me
N
26
Chemical Shift (ppm)

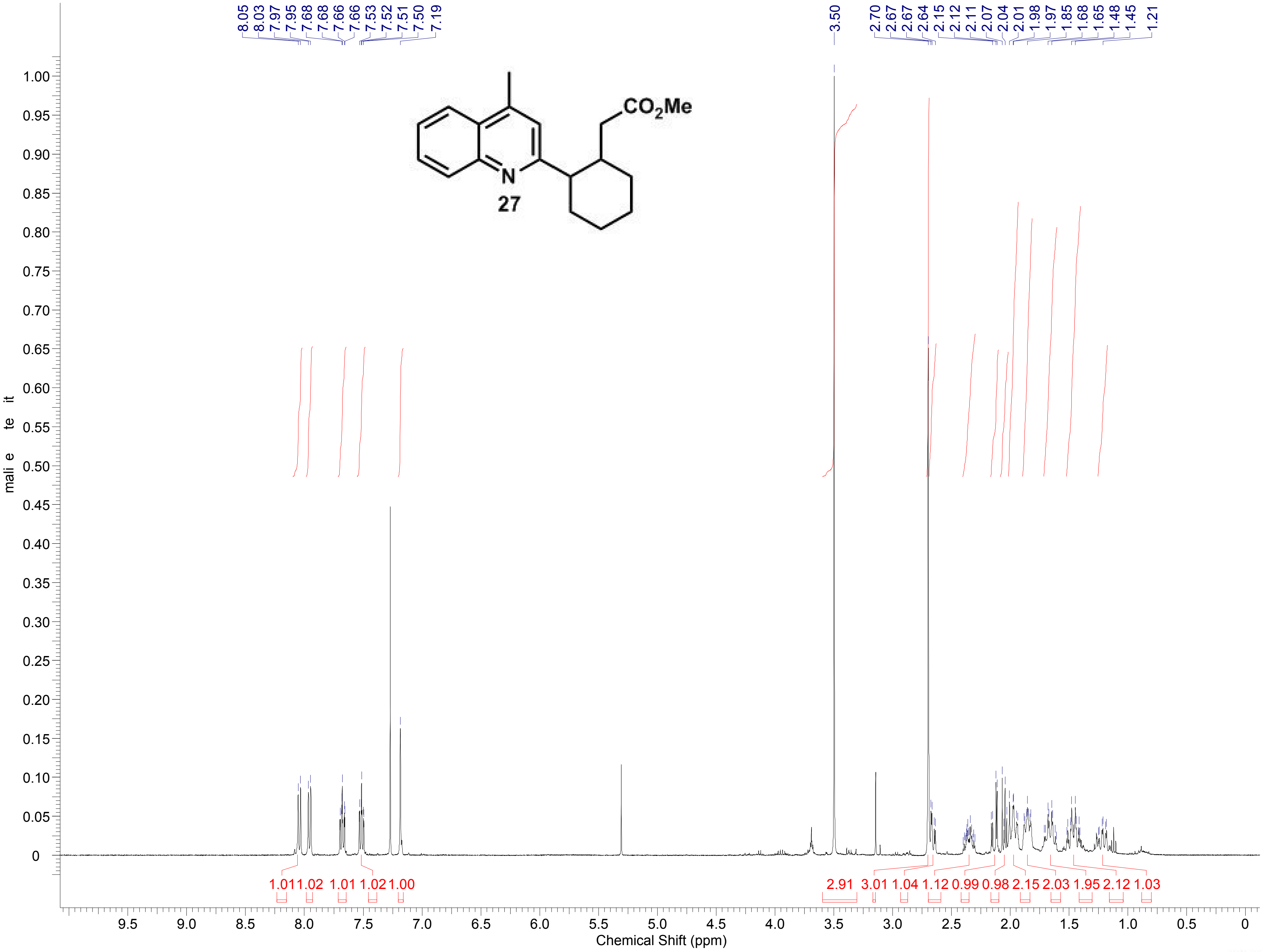

8.05 8.03 7.97 7.95 7.68 7.68 7.66 7.66 7.53 7.52 7.51 7.50 7.19
3.50
2.70 2.67 2.67 2.64 2.15 2.12 2.11 2.07 2.04 2.01 1.98 1.97 1.85 1.68 1.65 1.48 1.45 1.21
CO2Me
N
27
1.01 1.02 1.01 1.02 1.00
2.91 3.01 1.04 1.12 0.99 0.98 2.15 2.03 1.95 2.12 1.03
Chemical Shift (ppm)

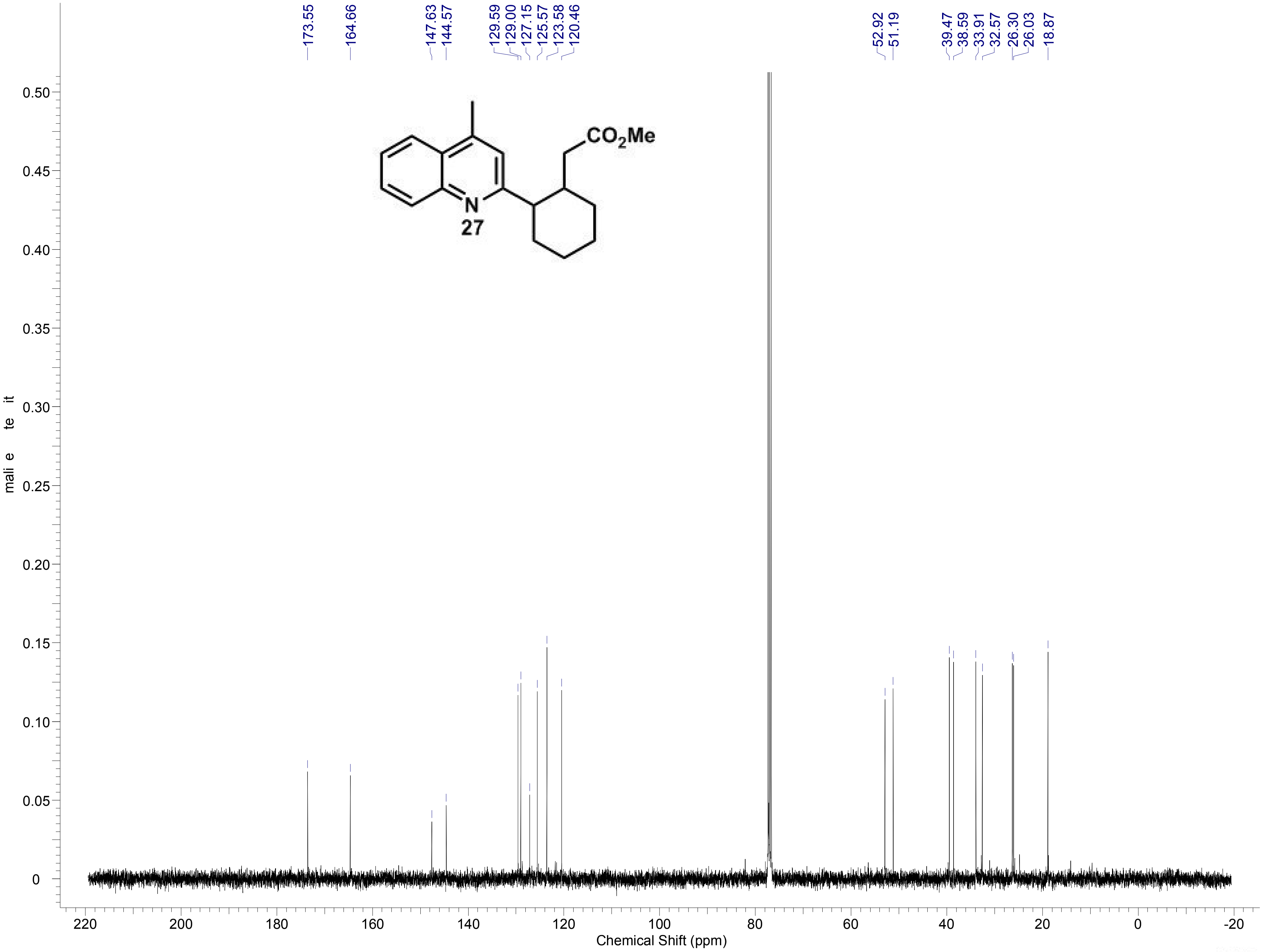
173.55
164.66
147.63
144.57
129.59
129.00
127.15
125.57
123.58
120.46
52.92
51.19
39.47
38.59
33.91
32.57
26.30
26.03
18.87
CO_2Me
N
27
mali e te it
0.50
0.45
0.40
0.35
0.30
0.25
0.20
0.15
0.10
0.05
0
220
200
180
160
140
120
100
80
60
40
20
0
-20
Chemical Shift (ppm)

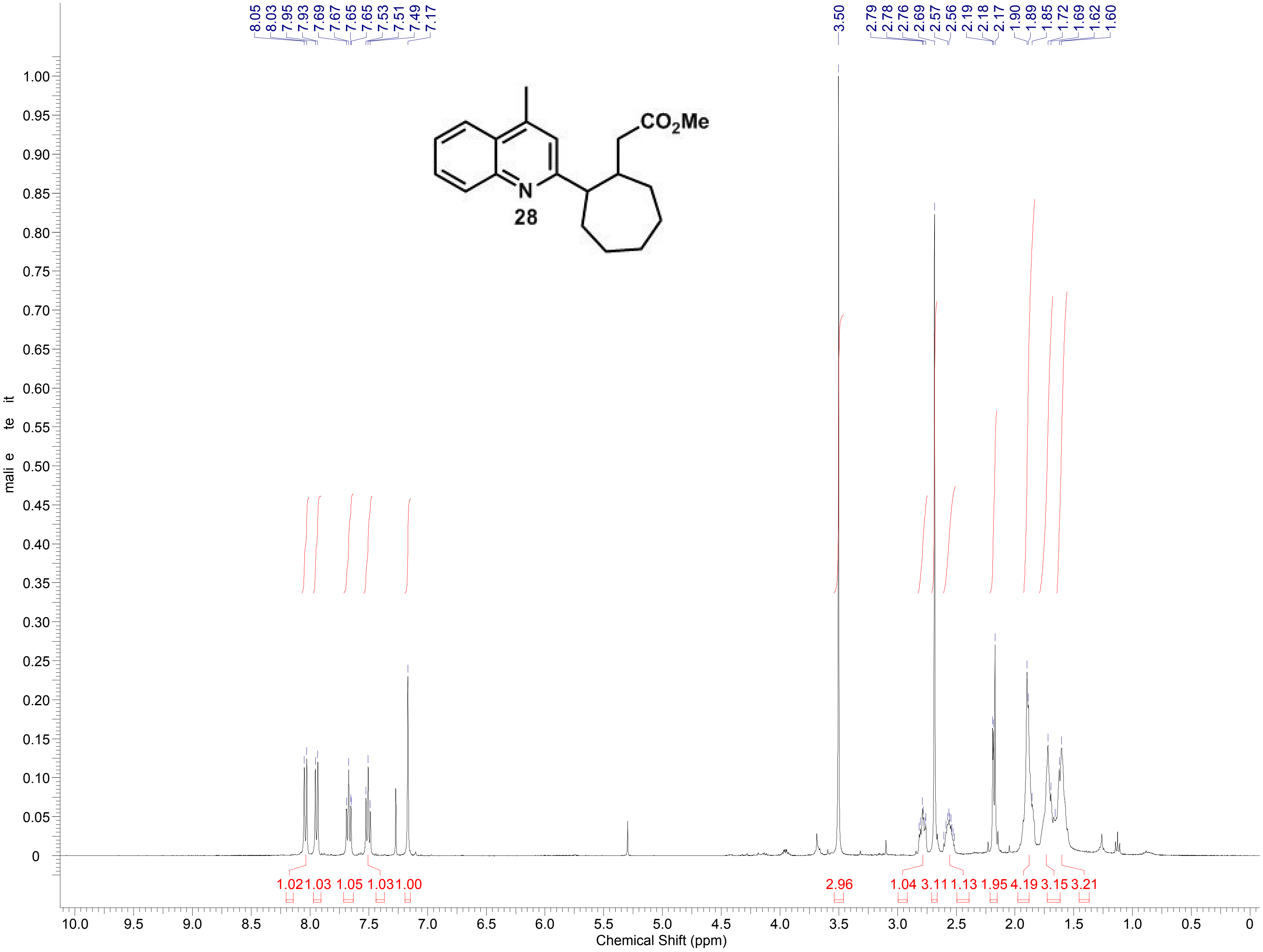

8.05 8.03 7.95 7.93 7.69 7.67 7.65 7.65 7.53 7.51 7.49 7.17
3.50
2.79 2.78 2.76 2.69 2.57 2.56 2.19 2.18 2.17 1.90 1.89 1.85 1.72 1.69 1.62 1.60
CO_2Me
N
28
1.02 1.03 1.05 1.03 1.00
2.96
1.04 3.11 1.13 1.95 4.19 3.15 3.21
Chemical Shift (ppm)

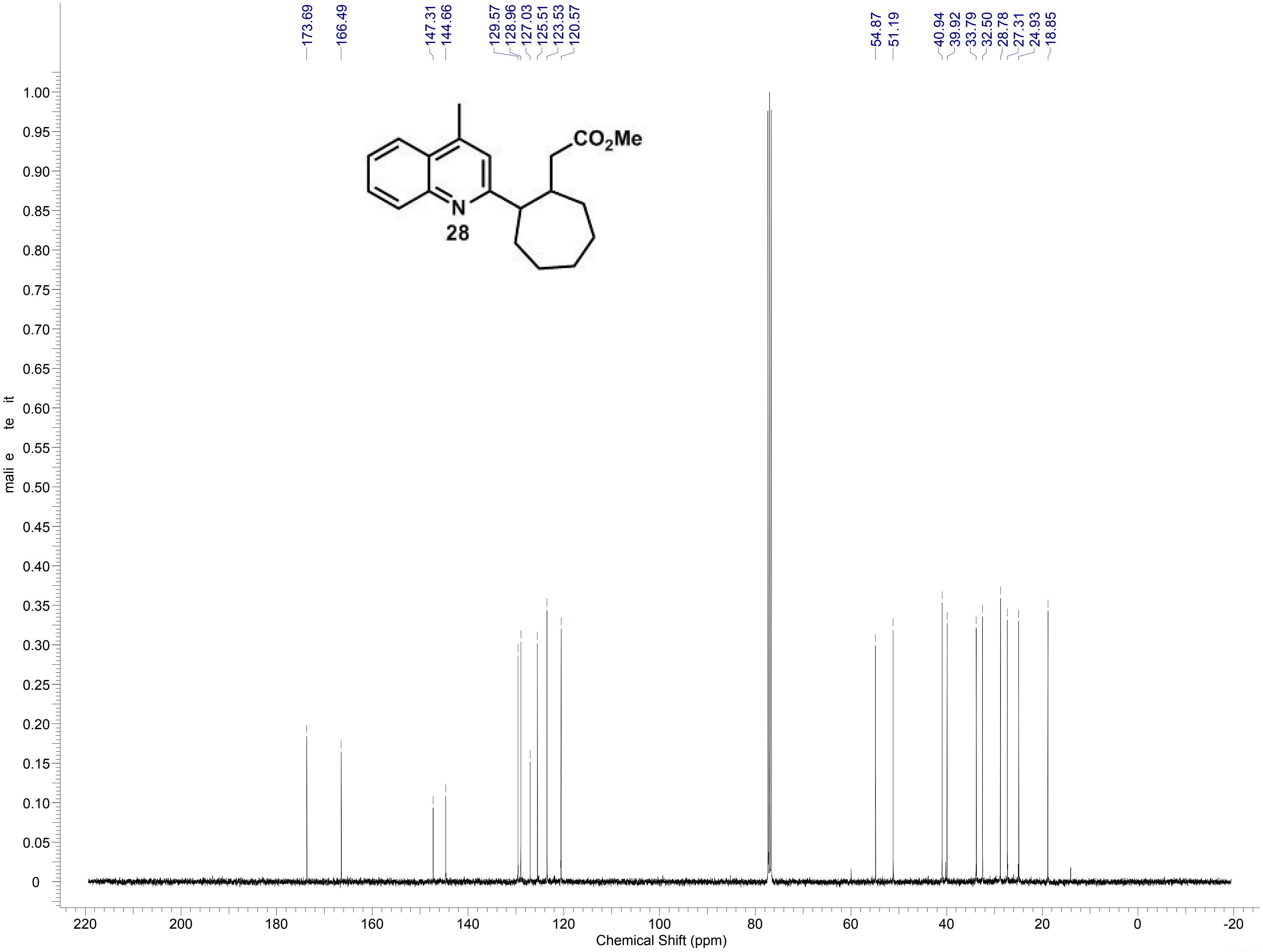
173.69
166.49
147.31
144.66
129.57
128.96
127.03
125.51
123.53
120.57
54.87
51.19
40.94
39.92
33.79
32.50
28.78
27.31
24.93
18.85
CO2Me
N
28
Chemical Shift (ppm)

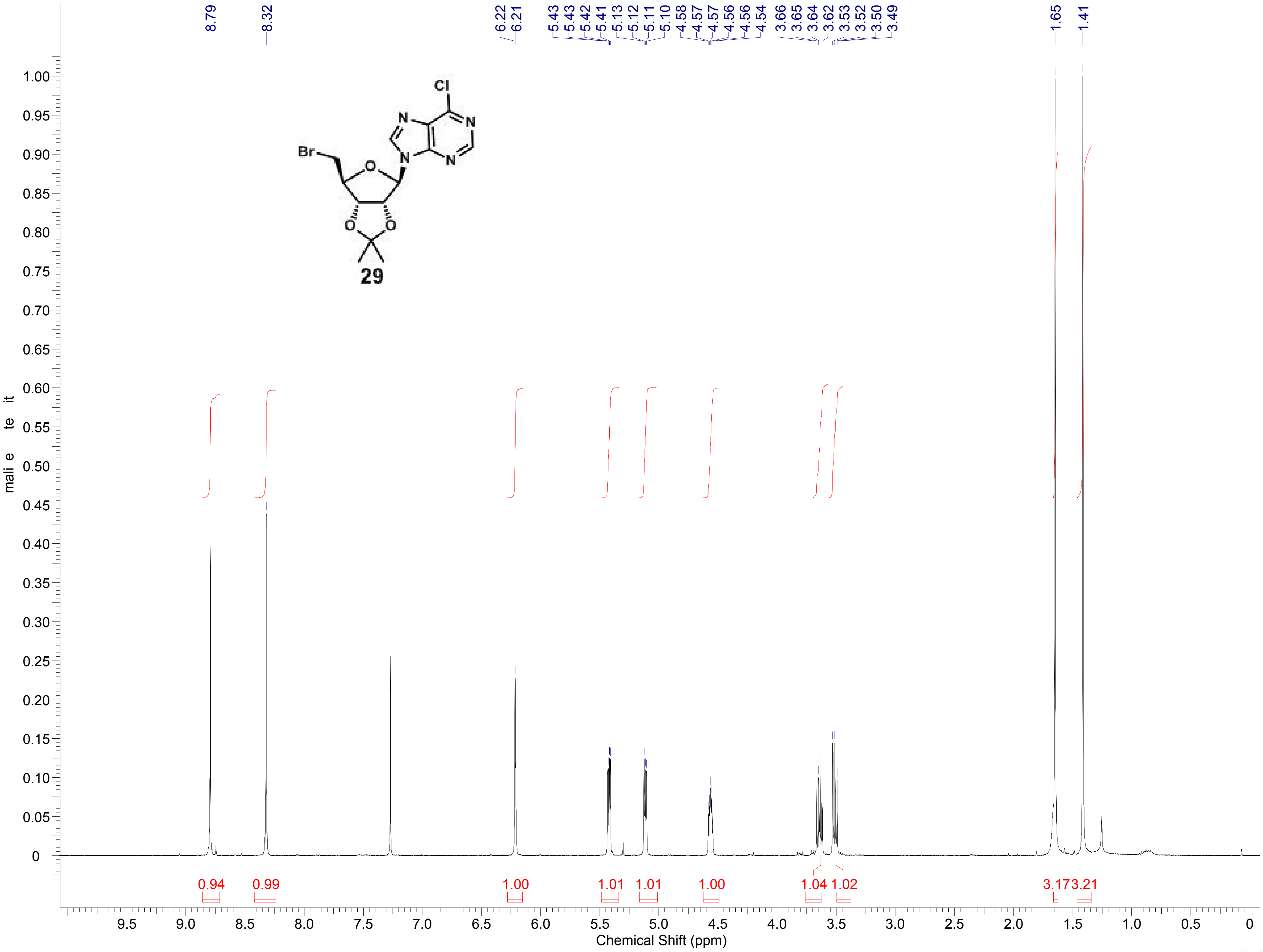
8.79
8.32
6.22
6.21
5.43
5.43
5.42
5.41
5.13
5.12
5.11
5.10
4.58
4.57
4.57
4.56
4.56
4.54
3.66
3.65
3.64
3.62
3.53
3.52
3.50
3.49
1.65
1.41
Cl
N
N
N
N
Br
O
O
O
29
0.94
0.99
1.00
1.01
1.01
1.00
1.04
1.02
3.17
3.21
Chemical Shift (ppm)

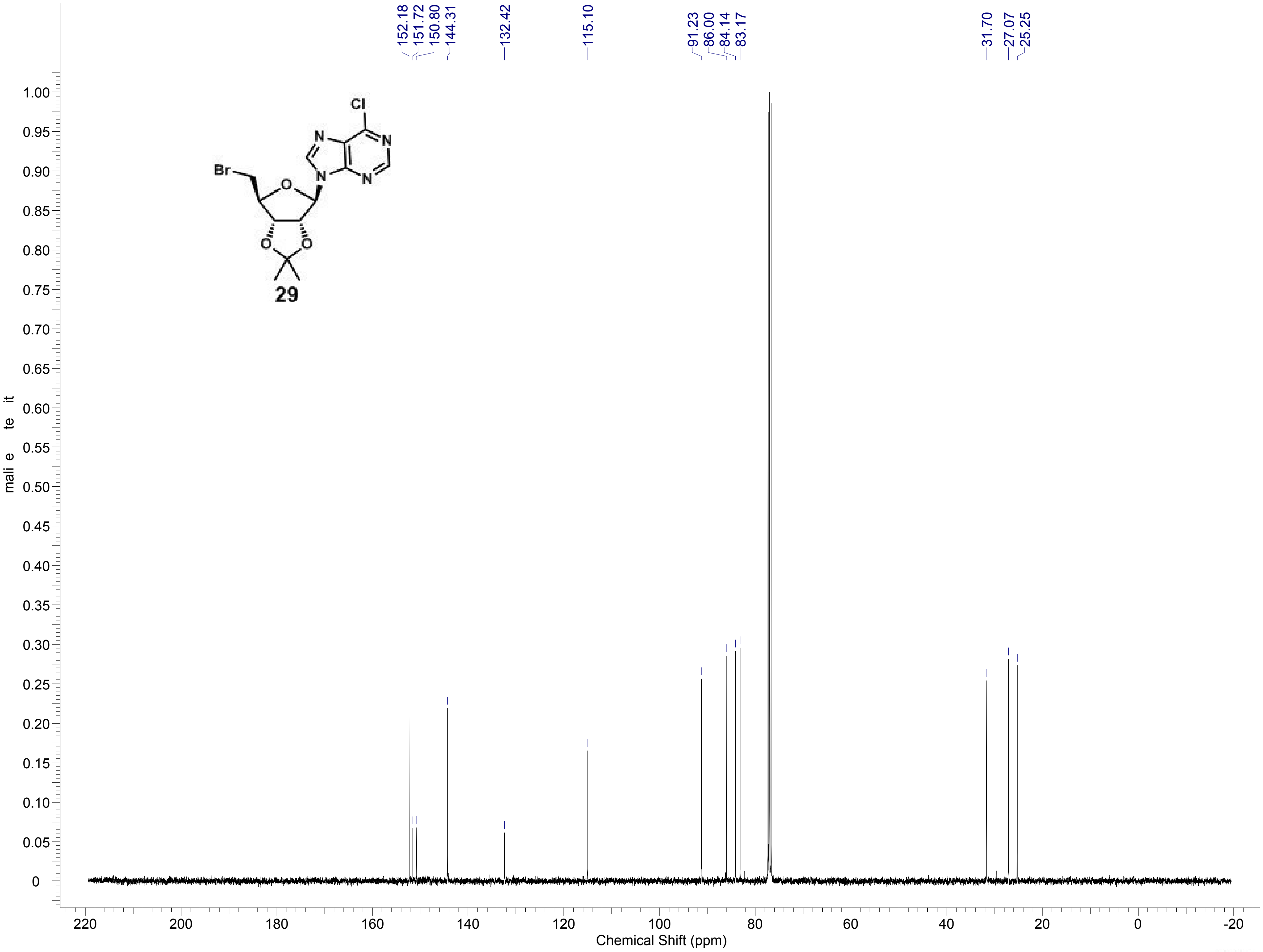

152.18
151.72
150.80
144.31
132.42
115.10
91.23
86.00
84.14
83.17
31.70
27.07
25.25
Cl
N
N
N
N
Br
O
O
O
29
1.00
0.95
0.90
0.85
0.80
0.75
0.70
0.65
0.60
0.55
0.50
0.45
0.40
0.35
0.30
0.25
0.20
0.15
0.10
0.05
0
220
200
180
160
140
120
100
80
60
40
20
0
-20
Chemical Shift (ppm)

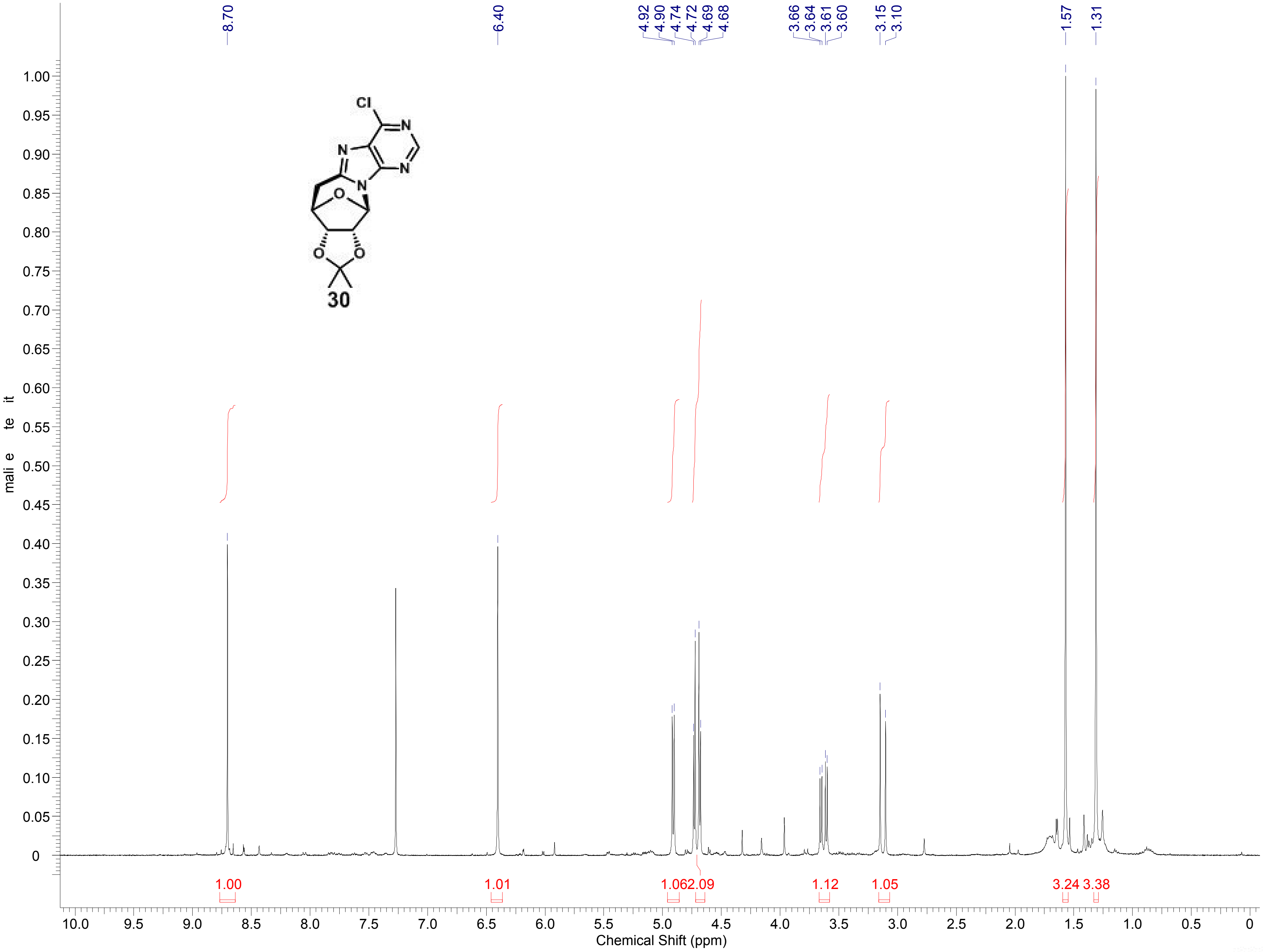
8.70
6.40
4.92 4.90 4.74 4.72 4.69 4.68
3.66 3.64 3.61 3.60
3.15 3.10
1.57
1.31
Cl
N
O
30
1.00
1.01
1.06 2.09
1.12
1.05
3.24 3.38
Chemical Shift (ppm)

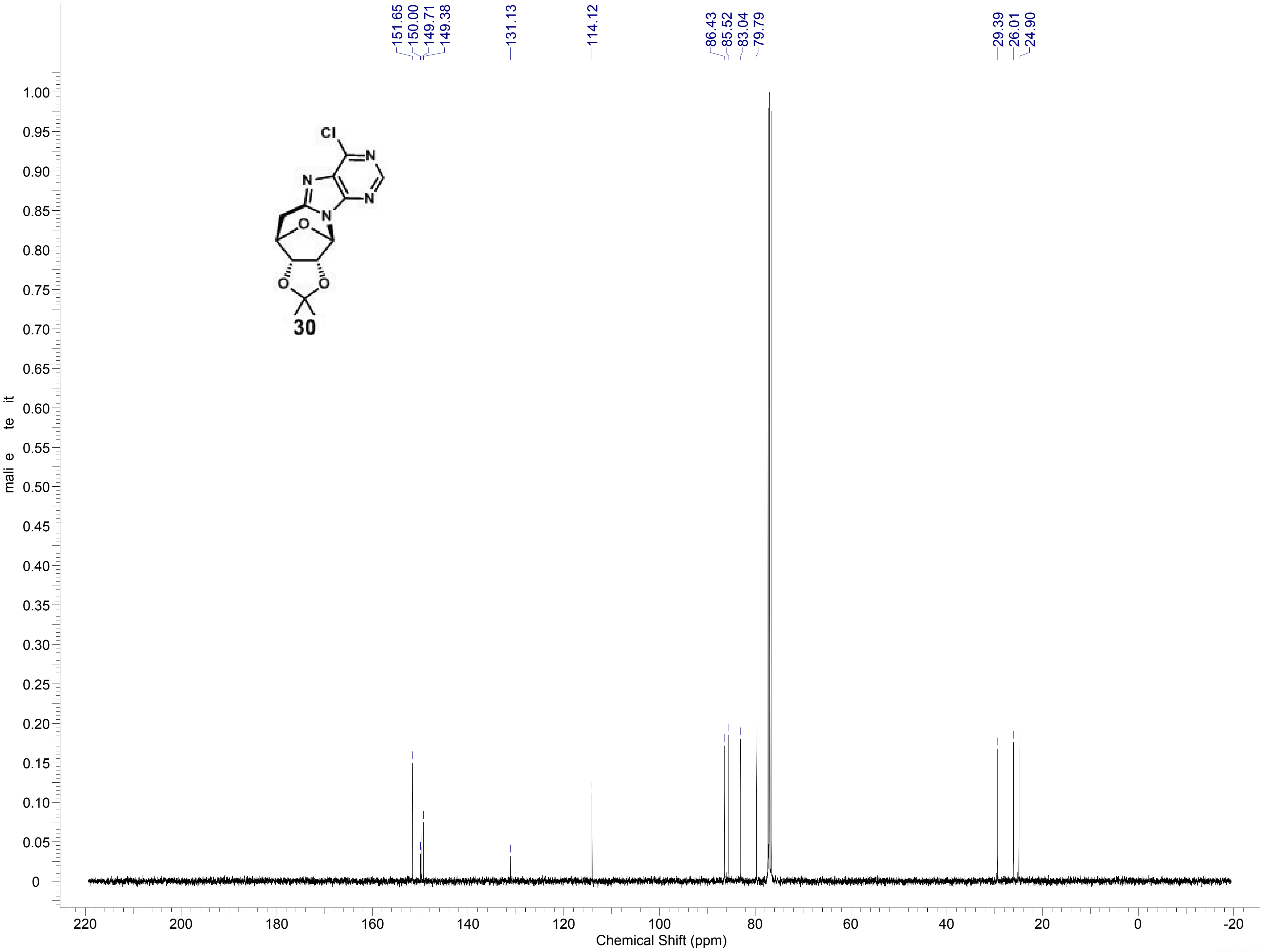
151.65
150.00
149.71
149.38
131.13
114.12
86.43
85.52
83.04
79.79
29.39
26.01
24.90
Cl
N
N
N
N
O
O
O
30
mali e te it
Chemical Shift (ppm)

6. X-Ray Diffraction Data

Crystal growth

Colorless block crystals suitable for X-ray analysis were grown from a Hex:DCM solution.

X-ray measurements

The X-ray diffraction data was collected on Bruker Kappa Apex II CCD diffractometer in the ω and φ-scan modes using Mo Kα (λ = 0.71073 Å) radiation. Images were indexed, integrated and scaled using the APEX II software package. The data was corrected for absorption using SADABS program. The structure was solved by direct methods in $P2_1$ space group and refined by full matrix least-squares method using SHELXTL and WinGX programs. All non-hydrogen atoms were refined anisotropically. The hydrogen atoms were placed at calculated positions and refined using the riding model. The crystal data, data collection and the refinement parameters are presented in Table S4. Figures were made using the Mercury program.

CCDC 1434820 contains the supplementary crystallographic data for this paper. The data can be obtained free of charge from The Cambridge Crystallographic Data Centre via www.ccdc.cam.ac.uk/getstructures.

Table S3. Crystal data and structure refinement parameters.

Identification code	shelx	
Empirical formula	C13 H13 Cl N4 O3	
Formula weight	308.72	
Temperature	200(2) K	
Wavelength	0.71073 Å	
Crystal system	Monoclinic	
Space group	P 21	
Unit cell dimensions	a = 7.7686(8) Å	a= 90°.
	b = 6.4525(6) Å	b= 91.2380(10)°.
	c = 13.9164(13) Å	g = 90°.
Volume	697.42(12) Å^{3}	
Z	2	
Density (calculated)	1.470 Mg/m^{3}	
Absorption coefficient	0.290 mm^{-1}	
F(000)	320	
Crystal size	0.773 x 0.636 x 0.544 mm^{3}	
Theta range for data collection	2.622 to 27.878°.	
Index ranges	-10<=h<=10, -8<=k<=8, -18<=l<=17	
Reflections collected	7028	
Independent reflections	3259 [R(int) = 0.0116]	
Completeness to theta = 25.242°	99.6 %	
Refinement method	Full-matrix least-squares on F^{2}	
Data / restraints / parameters	3259 / 1 / 192	
Goodness-of-fit on F^{2}	1.033	
Final R indices [I>2sigma(I)]	R1 = 0.0278, wR2 = 0.0704	
R indices (all data)	R1 = 0.0293, wR2 = 0.0720	
Absolute structure parameter	-0.290(17)	
Extinction coefficient	n/a	
Largest diff. peak and hole	0.186 and -0.182 e.Å^{-3}	
